# Characteristics of Hepatitis B virus integration and mechanism of inducing chromosome translocation

**DOI:** 10.1038/s41525-023-00355-y

**Published:** 2023-06-02

**Authors:** Xiaofang Cui, Yiyan Li, Hanshi Xu, Yuhui Sun, Shulong Jiang, Weiyang Li

**Affiliations:** 1 Jining Medical University, Jining, Shandong China; 2 School of Biological Science, Jining Medical University, Rizhao, Shandong China; 3 College of Life Sciences, University of Chinese Academy of Sciences, Beijing, China; 4 Institute of Microbiology, Chinese Academy of Sciences, 100101 Beijing, China; 5 BGI-Shenzhen, 518083 Shenzhen, China; 6 Clinical Medical Laboratory Center, Jining First People’s Hospital, Shandong First Medical University, Jining, China

**Keywords:** Cancer genomics, Tumour virus infections

## Abstract

Hepatitis B virus (HBV) integration is closely associated with the onset and progression of tumors. This study utilized the DNA of 27 liver cancer samples for high-throughput Viral Integration Detection (HIVID), with the overarching goal of detecting HBV integration. KEGG pathway analysis of breakpoints was performed using the ClusterProfiler software. The breakpoints were annotated using the latest ANNOVAR software. We identified 775 integration sites and detected two new hotspot genes for virus integration, *N4BP1* and *WASHP*, along with 331 new genes. Furthermore, we conducted a comprehensive analysis to determine the critical impact pathways of virus integration by combining our findings with the results of three major global studies on HBV integration. Meanwhile, we found common characteristics of virus integration hotspots among different ethnic groups. To specify the direct impact of virus integration on genomic instability, we explained the causes of inversion and the frequent occurrence of translocation due to HBV integration. This study detected a series of hotspot integration genes and specified common characteristics of critical hotspot integration genes. These hotspot genes are universal across different ethnic groups, providing an effective target for better research on the pathogenic mechanism. We also demonstrated more comprehensive key pathways affected by HBV integration and elucidated the mechanism for inversion and frequent translocation events due to virus integration. Apart from the great significance of the rule of HBV integration, the current study also provides valuable insights into the mechanism of virus integration.

## Introduction

Hepatitis B virus (HBV) integration has been frequently detected in liver cancer. With the advancement of high-throughput sequencing technology, significant progress has been made in understanding virus integration. First, Sung et al. found that there were HBV integration hotspots in cancer tissues by sequencing the whole genome of 88 tissue samples of liver cancer patients from Southeast Asia, and the hotspot genes were *TERT* (*n* = 18), *MLL4* (*n* = 9), and *CCNE1* (*n* = 4)^1^. Later, new high-throughput Viral Integration Detection (HIVID) methods significantly promoted the research on virus integration^2^. For example, Zhao et al. identified a series of HBV integration hotspots in liver cancer, such as *TERT* (101), *KMT2B* (31), *CCNE1* (7), and *CCNA2* (8), by utilizing the HIVID technology to detect 426 liver cancer samples from China^3^. In addition, they ascertained that virus integration is directly associated with clinical prognosis. Péneau *et al*. found that the gene hotspots of virus integration were *TERT* (*n* = 48), *CCNE1* (*n* = 4), and *KMT2B* (*n* = 3) by carrying out virus integration detection on 177 liver cancer samples from France^4^. Over the years, the research on HBV integration has gradually increased. HBV integration is known to cause cancer in at least three aspects: (1) HBV integration leads to abnormal gene expression at, before, and behind the virus integration site; (2) Viral protein is produced after the virus is integrated within an appropriate site; (3) Virus integration disturbs normal chromosome structure and chromatin accessibility, leading to the instability of many forms of genomes^5,6^. Thus, it is evident that virus integration induces hepatocellular diseases by disturbing the normal transcription and translation of hepatocytes.

Although a series of new hotspots of virus integration have been identified from the research on HBV integration, the data on HBV integration sites obtained by high-throughput technology is still minimal. Therefore, new HBV integration sites, especially new integration hotspots, need further exploration. Moreover, the key pathways affected by HBV integration and genomic instability due to virus integration remain unclear.

This study used 27 tumor samples for HIVID, which resulted in identifying 775 integration sites and detecting two new hotspot genes for virus integration, *N4BP1* and *WASHP*, and 331 new genes. Furthermore, we combined our results with three significant global studies on HBV integration to determine the pathways and functions related to virus integration sites. Results revealed that the essential hotspot genes of virus integration have common characteristics across ethnic groups. The study also found that the viral genome is integrated into the forward and reverse directions and frequently leads to events such as inversion in the genome. Consequently, we clarified the possible mechanism for the occurrence of inversion and the reason for frequent translocation events caused by HBV integration. Overall, this study detected new integration genes, hotspot genes, and more comprehensive critical pathways associated with the HBV integration sites. Our findings provide valuable insights into the mechanisms underlying inversion and translocation events due to virus integration. This study not only plays a positive role in the clinical application of virus integration but also effectively promotes elucidating the mechanism behind virus integration.

## Results

### Hotspot genes of HBV integration

**Fig. 1:**
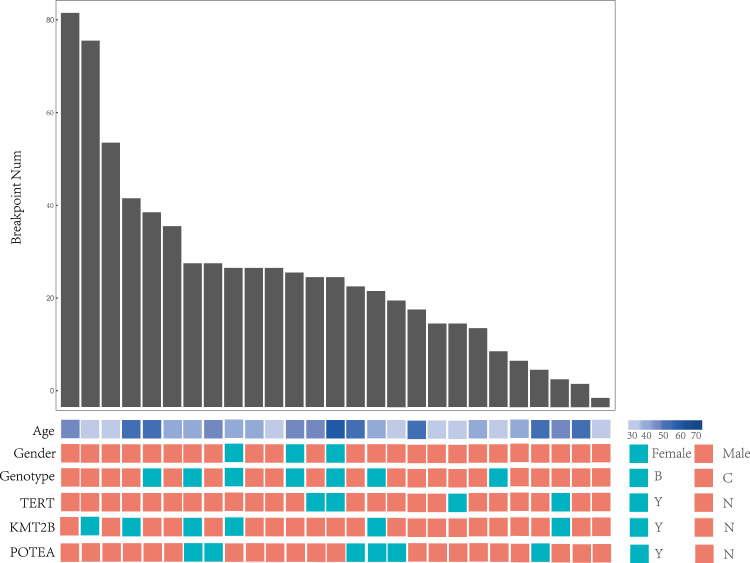
Clinical annotation of HBV integration breakpoints in the human genome of 27 HCC patients. All the panels are aligned with vertical tracks representing the 27 individuals.

**Fig. 2:**
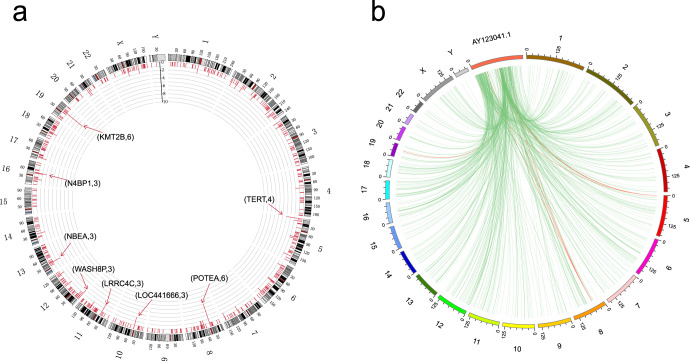
Distribution of HBV integration breakpoints within the human genomes of 27 samples. **a** Distribution of integration breakpoints across the human genome of 27 samples. Each bar represents the sampling frequency of HBV integration breakpoints at a particular locus within the human genome (hg19). Histogram axis units represent the number of samples. Some loci with a high frequency of integration are denoted. **b** HBV integration within the Human genome of 27 HCC samples. Each line represents HBV integration. One end means HBV genome breakpoints, and the other represents the human genome breakpoints. Red line represents the breakpoints in hotspot genes (TERT, KMT2B, and POTEA).

**Fig. 3:**
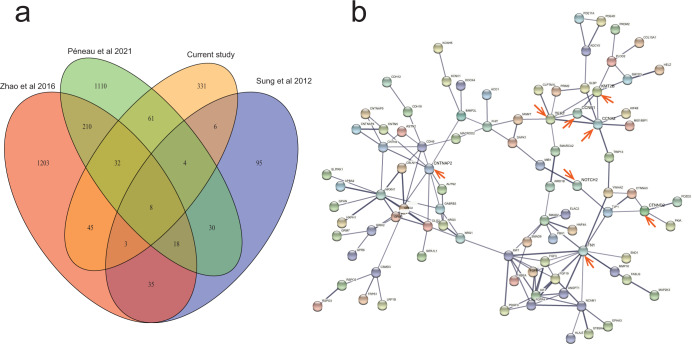
Venn diagram and the PPI network. **a** Venn diagram showing the genes from four studies, **b** The PPI network of recurrent genes.

The average number of integrated breakpoints in the 27 samples was 28.7. Results revealed four integrated samples in the *TERT* gene and six in *KMT2B*. We found that the hotspot genes of virus integration were *TERT* (4), *KMT2B* (6), and *POTEA* (6) (Figs. 1 and 2a, and Supplementary Table 1). There were two regions in the HBV genome where hotspots were easy to break: One was the 0–1000 bp region, and the other was the 1600–2000 bp region (Fig. 2b). After comparing the integrated hotspot genes with other studies^1,3,4^, we identified eight co-integrated hotspot genes (*TERT, KMT2B, EMBP1, LOC441666, MTRNR2L1, ANKRD26P1, RSPO2*, and *GABRB3*; Fig. 3a and Supplementary Table 2). This study discovered a total of 331 genes. Among them, the hotspot genes were *N4BP1* and *WASH8P*, and the viral integration of these genes occurred in three samples (Supplementary Table 1). It was also found that the key virus integration genes interacted with other genes (Fig. 3b).

### The pathway and network affected by HBV integration events

**Fig. 4:**
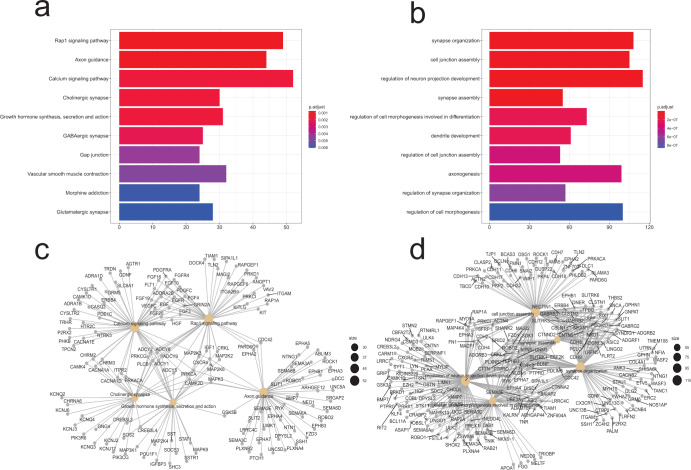
The enrichments of HBV integration breakpoints in KEGG pathways of HCC patients. **a** The histogram of HBV integration breakpoints in KEGG pathways. The vertical and horizontal axes illustrate the name of the pathway and the count of the enriched genes, respectively. The color indicates statistical significance, with red indicating a smaller *P*-value. **b** The histogram of HBV integration breakpoints in GO enrichment analysis. The vertical and horizontal axes illustrate the name of gene ontology terms and the count of the enriched genes, respectively. The color indicates statistical significance, with red indicating a smaller *P*-value. **c** The network map of the pathways and their HBV integration-related genes. The Gray node in the figure represents a gene; the Orange node represents the pathway; the circle size represents gene numbers in the pathway; the color line between the nodes represents the connection of pathways and genes. **d** The network map of gene ontology terms and their HBV integration-related genes. The Orange node represents the gene ontology term; the circle size represents gene numbers; the color line between the nodes represents the connection of the gene ontology term and genes.

Pathway analysis of all integrated genes from the four studies revealed that the significant pathways were the Rap1 signaling pathway, Axon guidance, Calcium signaling pathway, Cholinergic synapse, Growth hormone synthesis, secretion and action, and GABAergic synapse (Fig. 4a and Supplementary Table 3). In addition, the significant GO pathways were synapse organization, cell junction assembly, the regulation of neuron projection development, synapse assembly, and the regulation of cell morphogenesis involved in differentiation (Fig. 4b and Supplementary Table 4). Furthermore, the influencing genes of top 5 pathways in the KEGG and GO analysis were shown through network map (Fig. 4c, d).

### The characteristics and structure of HBV integration events

Among the 775 integration sites, 388 viruses were connected with the human genome in a forward direction and 387 in a reverse direction (Supplementary Table 5), depicting the universality of forward and reverse connection. In addition, we obtained two complete virus integration structures through long-fragment sequencing and analyzed their structures in detail (Supplementary Tables 6 and 7). The breakpoints of cancer and adjacent tissues were shown (Supplementary Tables 5 and 8).

## Discussion

This study detected the breakpoints of cancer tissues and adjacent tissues in 27 HCC patients. It was found that the intersection of the breakpoints between cancer and adjacent tissues was 3 (Supplementary Tables 5 and 8). At the same time, the intersection of the breakpoints-related genes between them was 38 (Supplementary Fig. 1). Therefore, in terms of breakpoints and gene levels, there are very few intersections between cancer and adjacent tissues, and given that the project mainly focused on the virus-integrated sites in the cancer tissues, we carried out a comprehensive analysis of downstream functional characterization using virus integrated sites in the cancer tissues. The virus integration hotspots were identified as *TERT* and *KMT2B*^*3*^. This study not only discovered two new integration genes, *N4BP1* and *WASHP* (both integrated three times), but also found that the sample integration frequencies of the *TERT* gene were 23.7% (101/426), 20.5% (18/88), 27.1% (48/177), and 14.8% (4/27), respectively, after comparing with previous studies. In addition, we found that the integration frequencies of *KMT2B* were 7.3% (31/426), 10.2% (9/88), 1.7% (3/177), and 22.2% (6/27), respectively^1,3,4^. This indicated significant differences among different studies, and the integration frequency of the *KMT2B* gene in Asia was much higher than that in Europe. Moreover, a comparison between the Asian and European populations revealed that there were a series of common hotspot genes (*TERT, KMT2B, EMBP1, LOC441666, MTRNR2L1, ANKRD26P1, RSPO2*, and *GABRB3*), indicating that there were some common characteristics of virus integration among the different ethnic groups. This suggests that the common virus integration events have certain regularity in enhancing tumor evolution, but the hypothesis requires further study. In addition, we found that *N4BP1* enables mRNA binding activity, ribonuclease activity, and ubiquitin-binding activity. It is also involved in the cellular response to UV and negative regulation of viral genome replication^7^. It acts as a restriction factor against some viruses, such as HIV-1: restricts HIV-1 replication by binding to HIV-1 mRNAs and mediating their degradation via its ribonuclease activity^8^. *WASH8P* (WAS Protein Family Homolog 8) is a pseudogene. Although little research has been done on this gene, it was recently been linked to rectal cancer^9^. Considering the limited knowledge of *N4BP1* and *WASH8P* genes and virus integration information, this study could not elaborate on how virus integration affects the two genes.

Herein, it was found that the regions of the HBV genome where breakpoints easily appear are the 0–1 K region and 1.6–2.0 K region. The regions before and after 1.8 K are the linear ends of the HBV genome. Moreover, the virus integration of linear double-stranded HBV genome is frequent^10^. The integration in the 0–1 K region could be due to abnormal rupture or secondary recombination during integration^11^. After conducting a comprehensive pathway analysis of all the integration genes in the four existing studies, we found that the virus integration sites were significantly enriched within the Rap1 signaling pathway, Axon guidance, Calcium signaling pathway, Cholinergic synapse, GABAergic synapse, Glutamatergic synapse, and other pathways. In addition, GO analysis demonstrated that the virus integration sites have specific effects on synapse organization, cell junction assembly, regulation of neuron projection development, and other pathways. Interestingly, the virus integration enriches neural transmission and key synaptic pathways. Thus, we speculated that in addition to the above three reasons which can lead to the tumor, the virus integration event may cause cancer by inducing the abnormality of neural transmission and the transmitter system. Current studies also indicate that neurotransmitters and growth factors scattered in the peripheral nervous system can trigger many cancers under experimental conditions, including pancreatic, gastric, colon, prostate, breast, oral, and skin^12,13^. Meanwhile, researchers have observed that the signal transduction among sympathetic nerve, parasympathetic nerve, and malignant cells in the tumor microenvironment usually regulates the onset or metastasis of cancer via the neurotransmitter-dependent signal transduction cascades. Studies have also revealed that in tumors, axonogenesis is promoted by the feedforward mechanism due to enhanced adrenergic or cholinergic signal transduction^12,14^. Therefore, virus integration events likely cause tumor onset and progression through interaction with neural signal pathways. Since virus integration events occur earlier than tumors^6^, they may affect relevant neural pathways during the early stage of tumors. Therefore, there is a need to explore the correlation between the onset and progression of early tumors with abnormal mental symptoms, including anxiety and depression, and psychosomatic symptoms.

**Fig. 5:**
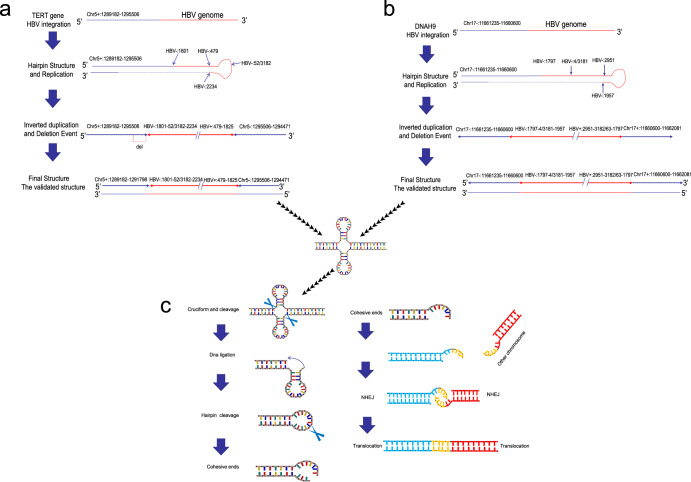
The mechanism behind HBV integration-induced inversion occurrence and translocation. **a** The mechanism behind HBV integration in *TERT*, **b** HBV integration in *DNAH9*. **c** The mechanism of induced inversion translocation.

This study found that the same direction connection often accompanies the connection site between the HBV genome and the human genome (++, −−) or reverse connection (+−, −+) during HBV integration. We identified 775 points and found that the same direction connection accounted for 388/775. In contrast, the reverse connection accounted for 387/775, indicating that insertion of virus integration happens in various directions. The proportion of reverse connection cases shows that virus integration leads to the frequent reverse connection of genomes. The reverse connection at both endpoints after virus integration indicates similar inversion events. However, although this phenomenon has been observed in many studies on HBV integration models^4,15^, the mechanism and impact behind this inversion event have not yet been elucidated. Thus, we speculate that the internal mechanism (Fig. 5a, b and Supplementary Tables 6 and 7) of the virus causing inversion is by developing a hairpin structure to replicate virus integration through an in-depth analysis of HBV integration on two typical sites. Furthermore, this inversion event is highly prone to form a cross structure which is easy to break and create a bare end^16,17^. When this end contacts the distal chromosome or other viruses to integrate and develop a bare end, it can cause translocation through non-homologous recombination (Fig. 5c). The above structural changes will lead to a high-degree structural variation of the human genome structure, resulting in a higher level of instability of the chromosome structure^17^. Therefore, HBV virus integration can lead to abnormal expression of integrated genes and the abnormal production of viral proteins. Moreover, it can induce the generation of tumors by directly causing a higher degree of genomic instability through the inversion and translocation events.

Conclusion: This study identified a series of new virus integration genes and expanded the latest knowledge of virus integration hotspots in the field of HBV integration. There is an extensive background of random integration and evident hotspot genes, such as *TERT* and *KMT2B*. We found that these hotspot genes are universal across different ethnic groups, providing an effective target for better research on the pathogenic mechanism and treatment of liver cancer through virus integration. Virus integration is widely believed to cause changes in genome structure, genomic instability, and abnormal expression of tumor suppressor genes, oncogenes, and viral genes. This study established that virus integration could directly cause inversion and translocation at the genomic structural level and found that virus integration can be closely associated with nerve axons and crucial synapses. Although the mechanism of virus integration affecting nerve axons and synaptic pathways requires further study, the close relationship with the nervous system has been gradually uncovered. Li et al. also observed that genes shared by HBV and HPV integration are enriched in nerve axons and essential synapses. These phenomena indicate that virus integration is closely linked with nervous system abnormalities during the carcinogenic process^11^. This study confirmed the commonness of virus integration hotspots among different ethnic groups and discovered new hotspot genes, describing the close relationship between virus integration and the nervous system. Moreover, the study explained the mechanism of inversion and translocation due to HBV integration. Overall, the research achievements will be significant for the clinical application of HBV integration and for studying carcinogenic mechanisms and drug targets.

## Methods

Twenty-seven HBV-positive hepatocellular carcinoma (HCC) samples were obtained from Jining Medical University. Supplementary Table 9 describes the data production process.

All the procedures performed in this study involving human participants followed the ethical standards of the institutional research committee based on the 1964 Helsinki Declaration and the later amendments or comparable ethical standards. The study was approved by the Ethics Review Committee of Jining Medical University. Written informed consent was obtained from each participant.

### HBV capture experiment

Genomic DNA was extracted from all samples using the Tiangen kit following the manufacturer’s instructions. The capture probes were designed from the DNA sequences of eight genotypes of HBV and synthesized by MyGenostics. The extracted DNA (1 ug) from each sample was sheared into fragments of 150–200 bp length using Covaris M220 (Covaris Inc., Woburn, MA). The fragments were then purified, end blunted, ‘A’ tailed, and adapter ligated to obtain a DNA library. Next, the hybridization process was carried according to MyGenostics GenCap Target Enrichment Protocol (GenCap Enrichment, MyGenostics, USA), followed by PE150 DNA sequencing (Illumina Inc., San Diego, CA).

### Detection of HBV integration sites

HBV integration sites were detected using the HIVID method^2^. First, low-quality, duplicate reads and adapter-contaminated reads are filtered out to obtain the clean data. Then, clean reads were mapped on to human (NCBI build 37, HG19) and HBV genomes by Burrows-Wheeler Aligner (BWA). Paired-end reads could be perfectly mapped to the human or HBV reference genome were removed. The remaining reads were used to reconstruct fragment based on the overlap of paired-end reads. Subsequently, the paired-end assembled reads were mapped onto human and HBV genomes using BWA^18^. The junction position (Breakpoints) of the human and HBV sequences was detected out in the paired-end assembled reads. Finally, the breakpoints of HBV integration were obtained. The breakpoints with total support read ≥ 5 were retained.

### Pathway analysis

KEGG pathway enrichment analysis of the integration breakpoints was performed using the Clusterprofiler software^19^, an intelligent bioinformatic tool for statistical and network analysis. The significance threshold for altered biological processes/pathways was set at a corrected hypergeometric *P*-value of 0.05^20,21^. The protein-protein interaction (PPI) networks were constructed based on the data from the STRING (https://cn.string-db.org/) database. The breakpoints were annotated using the latest ANNOVAR in hg19 coordinates^22^. The integrated viral genome has been considered a strong cis-activator of the flanking genes, and the cis-acting enhancers influence their target genes over long distances. Thus, genes near the breakpoints in the intergenic region were included in determining the affected gene in HBV-integrated samples^23,24^.

## Supplementary information

supplementary information

## Data Availability

The data used to support the findings of this study are available in the Supplementary Tables and deposited in the NCBI (BioProject accession number: PRJNA939923).
