# Supplementary material for: Characteristics of Hepatitis B virus integration and mechanism of inducing chromosome translocation
Source: NPJ Genom Med. 2023 Jun 2;8:11. doi: 10.1038/s41525-023-00355-y (PMC10238498; doi:10.1038/s41525-023-00355-y)
Supplement: Supplementary file 1 — supplementary information [file 41525_2023_355_MOESM1_ESM.pdf]

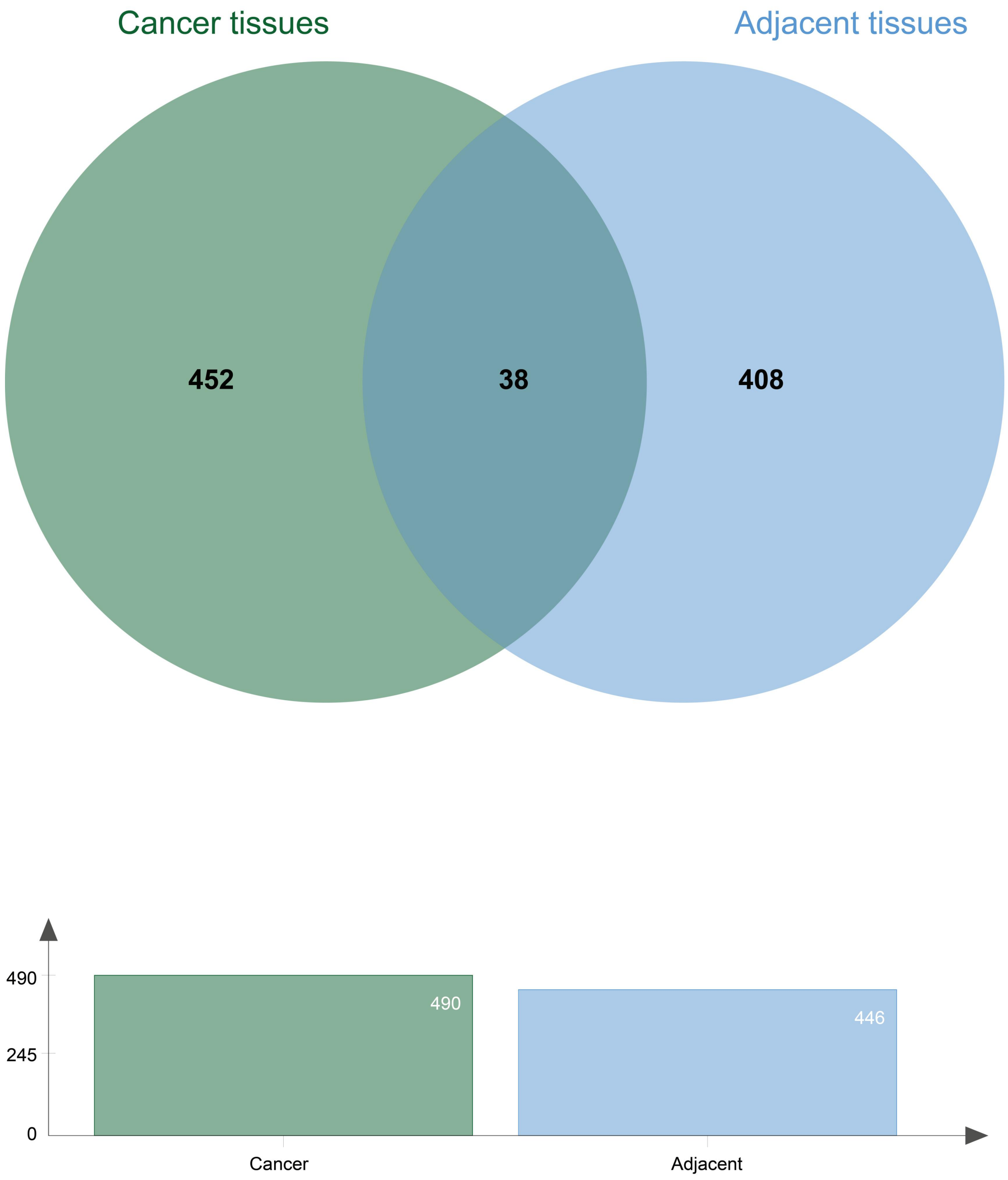


Supplementary Figure1

Supplementary Table 1 Data production

| ID | Sample | Raw_data_bases(Mb) | Clean_data_bases (Mb) | Aligned_bases (Mb) | Aligned | Effective bases on HBV | Fraction of effective bases on HBV | Duplication | Genotype |
|---|---|---|---|---|---|---|---|---|---|
| 1T | 20C211239_R303_CapNGS | 1989.11 | 1785.82 | 1391.94 | 77.94% | 72048080 | 5.18% | 32.42% | C |
| 2T | 20C211240_R303_CapNGS | 1872.22 | 1676.11 | 493.36 | 29.43% | 6943505 | 1.41% | 10.52% | C |
| 3T | 20C211241_R303_CapNGS | 1473.85 | 1320.24 | 506.66 | 38.38% | 16591558 | 3.27% | 14.73% | C |
| 4T | 20C211242_R303_CapNGS | 1757.81 | 1557.65 | 1094.25 | 70.25% | 37480082 | 3.43% | 30.54% | B |
| 5T | 20C211243_R303_CapNGS | 2154.14 | 1881.54 | 1849.73 | 98.31% | 173648233 | 9.39% | 39.83% | C |
| 6T | 20C211244_R303_CapNGS | 1913.49 | 1723.88 | 996.4 | 57.80% | 25715788 | 2.58% | 23.64% | C |
| 7T | 20C211245_R303_CapNGS | 1665.02 | 1485.56 | 57.93 | 3.90% | 1166868 | 2.01% | 1.79% | C |
| 8T | 20C211246_R303_CapNGS | 1736.46 | 1560.65 | 1454.22 | 93.18% | 42646249 | 2.93% | 41.74% | C |
| 9T | 20C211247_R303_CapNGS | 1500.32 | 1361.83 | 467.19 | 34.31% | 5379232 | 1.15% | 14.17% | C |
| 10T | 20C211248_R303_CapNGS | 1380.87 | 1205.36 | 1123.87 | 93.24% | 44004868 | 3.92% | 40.71% | B |
| 11T | 20C211249_R303_CapNGS | 1714.83 | 1546.89 | 1203.56 | 77.81% | 52494883 | 4.36% | 32.81% | B |
| 12T | 20C211250_R303_CapNGS | 1141.78 | 1022.34 | 802.28 | 78.47% | 79118944 | 9.86% | 31.03% | C |
| 13T | 20C211251_R303_CapNGS | 686.9 | 592.19 | 571.1 | 96.44% | 68826551 | 12.05% | 36.80% | C |
| 14T | 20C211252_R303_CapNGS | 1920.95 | 1747.38 | 1410.53 | 80.72% | 131825537 | 9.35% | 31.54% | C |
| 15T | 20C211253_R303_CapNGS | 2438.48 | 2211.71 | 2075.08 | 93.82% | 158845782 | 7.65% | 38.63% | C |
| 16T | 20C211254_R303_CapNGS | 1336.9 | 1190.66 | 1072.63 | 90.09% | 94846552 | 8.84% | 33.75% | C |
| 17T | 20C211255_R303_CapNGS | 1554.73 | 1425.99 | 961.36 | 67.42% | 32937946 | 3.43% | 28.86% | C |
| 18T | 20C211256_R303_CapNGS | 1195.85 | 1074.78 | 698.81 | 65.02% | 38440691 | 5.50% | 28.12% | C |
| 19T | 20C211257_R303_CapNGS | 1995.93 | 1771.62 | 1615.99 | 91.22% | 95360349 | 5.90% | 37.17% | C |
| 20T | 20C211258_R303_CapNGS | 1782.05 | 1628.45 | 1144.55 | 70.29% | 66850104 | 5.84% | 25.42% | B |
| 21T | 20C211259_R303_CapNGS | 1490.32 | 1347.5 | 1300.47 | 96.51% | 46552062 | 3.58% | 43.34% | B |
| 22T | 20C211260_R303_CapNGS | 729.83 | 657.88 | 559.42 | 85.03% | 50287929 | 8.99% | 29.82% | C |
| 23T | 20C211261_R303_CapNGS | 2350.57 | 2131.56 | 1621.36 | 76.06% | 71807653 | 4.43% | 31.10% | B |
| 24T | 20C211263_R303_CapNGS | 1795.96 | 1588.61 | 1408.3 | 88.65% | 62501098 | 4.44% | 37.81% | B |
| 25T | 20C211264_R303_CapNGS | 1811.94 | 1635.8 | 1564.25 | 95.63% | 212278179 | 13.57% | 35.69% | C |
| 26T | 20C211265_R303_CapNGS | 1597.71 | 1425.21 | 821.33 | 57.63% | 39433968 | 4.80% | 21.69% | C |
| 27T | 20C211266_R303_CapNGS | 1570.45 | 1403.56 | 1257.69 | 89.61% | 70937231 | 5.64% | 40.32% | C |

Supplementary Table 2 Gene frequency

| Gene | Frequency |
|---|---|
| POTEA | 6 |
| KMT2B | 6 |
| TERT | 4 |
| LRRC4C | 3 |
| LOC44166€ | 3 |
| N4BP1 | 3 |
| NBEA | 3 |
| WASH8P | 3 |
| ETV6 | 2 |
| KDM2B | 2 |
| SGCD | 2 |
| EDIL3 | 2 |
| MIR4790 | 2 |
| LINC0168З | 2 |
| CDH2 | 2 |
| SASH1 | 2 |
| SRD5A2 | 2 |
| IL7 | 2 |
| FN1 | 2 |
| ZNF292 | 2 |
| EMBP1 | 2 |
| MT-TS1 | 2 |
| MYH10 | 2 |
| ANO2 | 2 |
| KIFAP3 | 2 |
| UBE2E2-AS | 2 |
| DPP10 | 2 |
| CTNND2 | 2 |
| KLHL13 | 2 |
| MIR4268 | 2 |
| CEP162 | 2 |
| PTPRB | 2 |
| PTCSC3 | 2 |
| CHIC1 | 2 |
| LINC0108ε | 2 |
| EZH2 | 2 |
| PREX2 | 2 |
| FAM117B | 2 |
| GRM5 | 2 |
| ANKRD30BF | 2 |
| ANKRD26P1 | 2 |
| LINC0110ε | 2 |
| HNF4G | 2 |
| CCDC85A | 2 |
| LHFPL2 | 2 |
| ZNF804B | 2 |
| LINC0029Ç | 2 |
| CDR2 | 2 |

Supplementary Table 3 Venn data

| | Zhao et al 2016 | Sung et al 2012 | Peneau, C et al 2021 | Current study | ..count.. |
|---|---|---|---|---|---|
| 1 | TRUE | TRUE | TRUE | TRUE | 8 |
| 2 | FALSE | TRUE | TRUE | TRUE | 4 |
| 3 | TRUE | FALSE | TRUE | TRUE | 32 |
| 4 | FALSE | FALSE | TRUE | TRUE | 61 |
| 5 | TRUE | TRUE | FALSE | TRUE | 3 |
| 6 | FALSE | TRUE | FALSE | TRUE | 6 |
| 7 | TRUE | FALSE | FALSE | TRUE | 45 |
| 8 | FALSE | FALSE | FALSE | TRUE | 331 |
| 9 | TRUE | TRUE | TRUE | FALSE | 18 |
| 10 | FALSE | TRUE | TRUE | FALSE | 30 |
| 11 | TRUE | FALSE | TRUE | FALSE | 210 |
| 12 | FALSE | FALSE | TRUE | FALSE | 1110 |
| 13 | TRUE | TRUE | FALSE | FALSE | 35 |
| 14 | FALSE | TRUE | FALSE | FALSE | 95 |
| 15 | TRUE | FALSE | FALSE | FALSE | 1203 |

Supplementary Table 4 kegg pathway

| ID | Description | GeneRatio | BgRatio | pvalue | p.adjust | qvalue | Count |
|---|---|---|---|---|---|---|---|
| hsa04015 | Rap1 signaling pathway | 49/988 | 210/8105 | 3.94E-06 | 0.000757 | 0.00062 | 49 |
| hsa04360 | Axon guidance | 44/988 | 182/8105 | 4.60E-06 | 0.000757 | 0.00062 | 44 |
| hsa04020 | Calcium signaling pathway | 52/988 | 240/8105 | 1.92E-05 | 0.001636 | 0.00134 | 52 |
| hsa04725 | Cholinergic synapse | 30/988 | 113/8105 | 2.26E-05 | 0.001636 | 0.00134 | 30 |
| hsa04935 | Growth hormone synthesis, secretion and action | 31/988 | 119/8105 | 2.49E-05 | 0.001636 | 0.00134 | 31 |
| hsa04727 | GABAergic synapse | 25/988 | 89/8105 | 3.87E-05 | 0.00212 | 0.001737 | 25 |
| hsa04540 | Gap junction | 24/988 | 88/8105 | 9.21E-05 | 0.004328 | 0.003545 | 24 |
| hsa04270 | Vascular smooth muscle contraction | 32/988 | 134/8105 | 0.000116 | 0.004757 | 0.003897 | 32 |
| hsa05032 | Morphine addiction | 24/988 | 91/8105 | 0.000163 | 0.005953 | 0.004876 | 24 |
| hsa04724 | Glutamatergic synapse | 28/988 | 114/8105 | 0.000183 | 0.006019 | 0.00493 | 28 |
| hsa04151 | PI3K-Akt signaling pathway | 66/988 | 354/8105 | 0.000221 | 0.006611 | 0.005415 | 66 |
| hsa04010 | MAPK signaling pathway | 56/988 | 294/8105 | 0.000377 | 0.010323 | 0.008455 | 56 |
| hsa04912 | GnRH signaling pathway | 23/988 | 93/8105 | 0.000604 | 0.015286 | 0.01252 | 23 |
| hsa04810 | Regulation of actin cytoskeleton | 43/988 | 218/8105 | 0.000842 | 0.018864 | 0.015451 | 43 |
| hsa04916 | Melanogenesis | 24/988 | 101/8105 | 0.00086 | 0.018864 | 0.015451 | 24 |
| hsa04726 | Serotonergic synapse | 26/988 | 115/8105 | 0.00118 | 0.024267 | 0.019876 | 26 |
| hsa04921 | Oxytocin signaling pathway | 32/988 | 154/8105 | 0.001559 | 0.029281 | 0.023983 | 32 |
| hsa04072 | Phospholipase D signaling pathway | 31/988 | 148/8105 | 0.001602 | 0.029281 | 0.023983 | 31 |
| hsa04934 | Cushing syndrome | 32/988 | 155/8105 | 0.001741 | 0.030147 | 0.024693 | 32 |
| hsa05218 | Melanoma | 18/988 | 72/8105 | 0.002012 | 0.03309 | 0.027103 | 18 |
| hsa04152 | AMPK signaling pathway | 26/988 | 120/8105 | 0.002251 | 0.03527 | 0.028889 | 26 |
| hsa04310 | Wnt signaling pathway | 33/988 | 166/8105 | 0.002839 | 0.041127 | 0.033686 | 33 |
| hsa04911 | Insulin secretion | 20/988 | 86/8105 | 0.002952 | 0.041127 | 0.033686 | 20 |
| hsa04750 | Inflammatory mediator regulation of TRP channels | 22/988 | 98/8105 | 0.003 | 0.041127 | 0.033686 | 22 |
| hsa04723 | Retrograde endocannabinoid signaling | 30/988 | 148/8105 | 0.003192 | 0.042003 | 0.034404 | 30 |
| hsa04927 | Cortisol synthesis and secretion | 16/988 | 65/8105 | 0.004127 | 0.05222 | 0.042772 | 16 |
| hsa04728 | Dopaminergic synapse | 27/988 | 132/8105 | 0.004394 | 0.053543 | 0.043856 | 27 |
| hsa04144 | Endocytosis | 45/988 | 252/8105 | 0.005002 | 0.058776 | 0.048142 | 45 |
| hsa04024 | cAMP signaling pathway | 40/988 | 219/8105 | 0.005317 | 0.060321 | 0.049407 | 40 |
| hsa04713 | Circadian entrainment | 21/988 | 97/8105 | 0.005766 | 0.061427 | 0.050313 | 21 |
| hsa00534 | Glycosaminoglycan biosynthesis - heparan sulfate / heparin | 8/988 | 24/8105 | 0.005788 | 0.061427 | 0.050313 | 8 |
| hsa04510 | Focal adhesion | 37/988 | 201/8105 | 0.006308 | 0.064856 | 0.053122 | 37 |
| hsa05226 | Gastric cancer | 29/988 | 149/8105 | 0.006738 | 0.067179 | 0.055025 | 29 |
| hsa00310 | Lysine degradation | 15/988 | 63/8105 | 0.007483 | 0.070415 | 0.057675 | 15 |
| hsa04918 | Thyroid hormone synthesis | 17/988 | 75/8105 | 0.007705 | 0.070415 | 0.057675 | 17 |
| hsa05214 | Glioma | 17/988 | 75/8105 | 0.007705 | 0.070415 | 0.057675 | 17 |
| hsa04722 | Neurotrophin signaling pathway | 24/988 | 119/8105 | 0.008364 | 0.074371 | 0.060915 | 24 |
| hsa04971 | Gastric acid secretion | 17/988 | 76/8105 | 0.008831 | 0.076457 | 0.062624 | 17 |
| hsa05165 | Human papillomavirus infection | 55/988 | 331/8105 | 0.009533 | 0.08042 | 0.06587 | 55 |

Supplementary Table 5 GO analysis

| ID | Description | GeneRatic | BgRatio | pvalue | p.adjust | qvalue | Count |
|---|---|---|---|---|---|---|---|
| GO:0050808 | synapse organization | 108/2119 | 408/18670 | 1.08E-17 | 6.52E-14 | 5.82E-14 | 108 |
| GO:0034329 | cell junction assembly | 105/2119 | 409/18670 | 3.09E-16 | 9.34E-13 | 8.33E-13 | 105 |
| GO:0010975 | regulation of neuron projection development | 115/2119 | 499/18670 | 4.77E-14 | 9.63E-11 | 8.59E-11 | 115 |
| GO:0007416 | synapse assembly | 55/2119 | 177/18670 | 1.22E-12 | 1.85E-09 | 1.65E-09 | 55 |
| GO:0010769 | regulation of cell morphogenesis involved in differentiation | 73/2119 | 301/18670 | 1.86E-10 | 2.25E-07 | 2.01E-07 | 73 |
| GO:0016358 | dendrite development | 61/2119 | 233/18670 | 2.27E-10 | 2.29E-07 | 2.04E-07 | 61 |
| GO:1901888 | regulation of cell junction assembly | 53/2119 | 192/18670 | 4.33E-10 | 3.70E-07 | 3.30E-07 | 53 |
| GO:0007409 | axonogenesis | 99/2119 | 468/18670 | 4.89E-10 | 3.70E-07 | 3.30E-07 | 99 |
| GO:0050807 | regulation of synapse organization | 57/2119 | 218/18670 | 9.24E-10 | 6.21E-07 | 5.54E-07 | 57 |
| GO:0022604 | regulation of cell morphogenesis | 100/2119 | 484/18670 | 1.52E-09 | 8.58E-07 | 7.65E-07 | 100 |
| GO:0098742 | cell-cell adhesion via plasma-membrane adhesion molecules | 66/2119 | 273/18670 | 1.58E-09 | 8.58E-07 | 7.65E-07 | 66 |
| GO:0050803 | regulation of synapse structure or activity | 58/2119 | 227/18670 | 1.70E-09 | 8.58E-07 | 7.65E-07 | 58 |
| GO:0051963 | regulation of synapse assembly | 35/2119 | 106/18670 | 2.45E-09 | 1.14E-06 | 1.02E-06 | 35 |
| GO:0007411 | axon guidance | 64/2119 | 276/18670 | 1.58E-08 | 6.84E-06 | 6.10E-06 | 64 |
| GO:0060560 | developmental growth involved in morphogenesis | 57/2119 | 235/18670 | 1.78E-08 | 6.84E-06 | 6.10E-06 | 57 |
| GO:0097485 | neuron projection guidance | 64/2119 | 277/18670 | 1.84E-08 | 6.84E-06 | 6.10E-06 | 64 |
| GO:0050769 | positive regulation of neurogenesis | 95/2119 | 474/18670 | 1.92E-08 | 6.84E-06 | 6.10E-06 | 95 |
| GO:0016049 | cell growth | 96/2119 | 484/18670 | 2.76E-08 | 9.28E-06 | 8.27E-06 | 96 |
| GO:0042391 | regulation of membrane potential | 88/2119 | 434/18670 | 3.67E-08 | 1.17E-05 | 1.04E-05 | 88 |
| GO:0050804 | modulation of chemical synaptic transmission | 88/2119 | 436/18670 | 4.57E-08 | 1.38E-05 | 1.23E-05 | 88 |
| GO:0099177 | regulation of trans-synaptic signaling | 88/2119 | 437/18670 | 5.10E-08 | 1.47E-05 | 1.31E-05 | 88 |
| GO:0007156 | homophilic cell adhesion via plasma membrane adhesion molecules | 44/2119 | 168/18670 | 7.01E-08 | 1.93E-05 | 1.72E-05 | 44 |
| GO:0031346 | positive regulation of cell projection organization | 79/2119 | 383/18670 | 8.58E-08 | 2.26E-05 | 2.01E-05 | 79 |
| GO:0048588 | developmental cell growth | 55/2119 | 234/18670 | 9.84E-08 | 2.48E-05 | 2.21E-05 | 55 |
| GO:0050773 | regulation of dendrite development | 40/2119 | 149/18670 | 1.32E-07 | 3.14E-05 | 2.80E-05 | 40 |
| GO:0050770 | regulation of axonogenesis | 46/2119 | 183/18670 | 1.35E-07 | 3.14E-05 | 2.80E-05 | 46 |
| GO:0051965 | positive regulation of synapse assembly | 24/2119 | 68/18670 | 1.88E-07 | 4.21E-05 | 3.76E-05 | 24 |
| GO:0050919 | negative chemotaxis | 19/2119 | 46/18670 | 2.04E-07 | 4.42E-05 | 3.94E-05 | 19 |
| GO:0099173 | postsynapse organization | 42/2119 | 163/18670 | 2.25E-07 | 4.69E-05 | 4.18E-05 | 42 |
| GO:0048638 | regulation of developmental growth | 72/2119 | 347/18670 | 2.50E-07 | 5.04E-05 | 4.49E-05 | 72 |
| GO:0001558 | regulation of cell growth | 82/2119 | 416/18670 | 3.70E-07 | 7.21E-05 | 6.43E-05 | 82 |

| | | | | | | | |
|---|---|---|---|---|---|---|---|
| GO:0099003 | vesicle-mediated transport in synapse | 50/2119 | 213/18670 | 3.85E-07 | 7.21E-05 | 6.43E-05 | 50 |
| GO:0090596 | sensory organ morphogenesis | 57/2119 | 256/18670 | 4.05E-07 | 7.21E-05 | 6.43E-05 | 57 |
| GO:0010976 | positive regulation of neuron projection | 61/2119 | 281/18670 | 4.08E-07 | 7.21E-05 | 6.43E-05 | 61 |
| GO:0045666 | positive regulation of neuron differentiation | 75/2119 | 371/18670 | 4.17E-07 | 7.21E-05 | 6.43E-05 | 75 |
| GO:0001655 | urogenital system development | 68/2119 | 330/18670 | 6.95E-07 | 0.00012 | 0.0001 | 68 |
| GO:0048813 | dendrite morphogenesis | 37/2119 | 143/18670 | 1.03E-06 | 0.00017 | 0.00015 | 37 |
| GO:0007265 | Ras protein signal transduction | 85/2119 | 448/18670 | 1.20E-06 | 0.00019 | 0.00017 | 85 |
| GO:0014033 | neural crest cell differentiation | 27/2119 | 90/18670 | 1.38E-06 | 0.00021 | 0.00019 | 27 |
| GO:0048864 | stem cell development | 26/2119 | 85/18670 | 1.43E-06 | 0.00021 | 0.00019 | 26 |
| GO:1990138 | neuron projection extension | 41/2119 | 168/18670 | 1.45E-06 | 0.00021 | 0.00019 | 41 |
| GO:1901890 | positive regulation of cell junction assembly | 29/2119 | 101/18670 | 1.55E-06 | 0.00022 | 0.0002 | 29 |
| GO:0060562 | epithelial tube morphogenesis | 65/2119 | 322/18670 | 2.54E-06 | 0.00036 | 0.00032 | 65 |
| GO:0044331 | cell-cell adhesion mediated by cadherin | 17/2119 | 44/18670 | 2.71E-06 | 0.00037 | 0.00033 | 17 |
| GO:0007215 | glutamate receptor signaling pathway | 28/2119 | 100/18670 | 4.00E-06 | 0.00053 | 0.00048 | 28 |
| GO:0007605 | sensory perception of | 36/2119 | 145/18670 | 4.06E-06 | 0.00053 | 0.00048 | 36 |
| GO:0099504 | synaptic vesicle cycle | 44/2119 | 193/18670 | 4.28E-06 | 0.00055 | 0.00049 | 44 |
| GO:0050768 | negative regulation of neurogenesis | 60/2119 | 295/18670 | 4.75E-06 | 0.0006 | 0.00053 | 60 |
| GO:0051961 | negative regulation of nervous system | 63/2119 | 316/18670 | 5.50E-06 | 0.00067 | 0.00059 | 63 |
| GO:0034332 | adherens junction organization | 21/2119 | 65/18670 | 5.50E-06 | 0.00067 | 0.00059 | 21 |
| GO:0001763 | morphogenesis of a branching structure | 44/2119 | 196/18670 | 6.53E-06 | 0.00076 | 0.00068 | 44 |
| GO:2001222 | regulation of neuron migration | 16/2119 | 42/18670 | 6.60E-06 | 0.00076 | 0.00068 | 16 |
| GO:0014032 | neural crest cell | 24/2119 | 81/18670 | 6.63E-06 | 0.00076 | 0.00068 | 24 |
| GO:0051056 | regulation of small GTPase mediated signal | 66/2119 | 338/18670 | 6.87E-06 | 0.00077 | 0.00069 | 66 |
| GO:0048568 | embryonic organ development | 79/2119 | 428/18670 | 8.21E-06 | 0.0009 | 0.00081 | 79 |
| GO:0008038 | neuron recognition | 17/2119 | 48/18670 | 1.08E-05 | 0.00114 | 0.00102 | 17 |
| GO:0016339 | calcium-dependent cell-cell adhesion via plasma membrane cell adhesion molecules | 17/2119 | 48/18670 | 1.08E-05 | 0.00114 | 0.00102 | 17 |
| GO:0001764 | neuron migration | 37/2119 | 157/18670 | 1.09E-05 | 0.00114 | 0.00102 | 37 |

Supplementary Table 6 Breakpoints of cancer

| ID | Sample | Viral_Transcript | Host_Gene | Total support- | Chromosom | Position 1 | Strand 1 | Chromosom | Position | St | CytoBand |
|---|---|---|---|---|---|---|---|---|---|---|---|
| 1T | 20C211239_R303_CapNGS | HBVCgp4_PREC/C | ARHGAP44//intronic | 5 | chr17 | 12694870 | + | AY123041 | 2026 | + | 17p12 |
| 1T | 20C211239_R303_CapNGS | HBVCgp3_X//upstream | PFKFB3//intronic | 5 | chr10 | 6287802 | + | AY123041 | 350 | - | 10p15.1 |
| 1T | 20C211239_R303_CapNGS | HBVCgp3_X//upstream | HSD17B7(dist=17082),CCDC190(dist=24397)//intergenic | 5 | chr1 | 162799690 | + | AY123041 | 505 | - | 1q23.3 |
| 1T | 20C211239_R303_CapNGS | HBVCgp4_PREC/C | FAM230B//ncRNA_intronic | 6 | chr22 | 21534373 | + | AY123041 | 1908 | - | 22q11.21 |
| 1T | 20C211239_R303_CapNGS | HBVCgp3_X//upstream | FAF1//intronic | 6 | chr1 | 50943763 | + | AY123041 | 369 | - | 1p32.3 |
| 1T | 20C211239_R303_CapNGS | HBVCgp3_X//upstream | MCF2L2//intronic | 7 | chr3 | 183015429 | + | AY123041 | 274 | + | 3q27.1 |
| 1T | 20C211239_R303_CapNGS | HBVCgp4_PREC/C | LOC102724050//ncRNA_intronic | 8 | chr12 | 54810749 | + | AY123041 | 1958 | + | 12q13.13 |
| 1T | 20C211239_R303_CapNGS | HBVCgp4_PREC/C//downstream | MSC-AS1//ncRNA_intronic | 10 | chr8 | 72951569 | - | AY123041 | 2817 | + | 8q13.3 |
| 1T | 20C211239_R303_CapNGS | HBVCgp3_X//upstream | CCDC85A(dist=186738),VRK2(dist=1334739)//intergenic | 20 | chr2 | 56800047 | + | AY123041 | 359 | - | 2p16.1 |
| 1T | 20C211239_R303_CapNGS | HBVCgp4_PREC/C | FAM124B(dist=30167),CUL3(dist=37876)//intergenic | 22 | chr2 | 225296991 | + | AY123041 | 2066 | + | 2q36.2 |
| 1T | 20C211239_R303_CapNGS | HBVCgp4_PREC/C | CRADD//intronic | 48 | chr12 | 94207073 | - | AY123041 | 1976 | + | 12q22 |
| 1T | 20C211239_R303_CapNGS | HBVCgp3_X//upstream | NBEA//intronic | 81 | chr13 | 35590942 | - | AY123041 | 668 | + | 13q13.3 |
| 1T | 20C211239_R303_CapNGS | HBVCgp3_X//upstream | NBEA//intronic | 81 | chr13 | 35590984 | - | AY123041 | 668 | + | 13q13.3 |
| 1T | 20C211239_R303_CapNGS | HBVCgp3_X//upstream | NBEA//intronic | 82 | chr13 | 35590988 | - | AY123041 | 668 | + | 13q13.3 |
| 1T | 20C211239_R303_CapNGS | HBVCgp4_PREC/C | LINC01680(dist=50998),RGS18(dist=878966)//intergenic | 108 | chr1 | 191248626 | + | AY123041 | 2003 | - | 1q31.2 |
| 1T | 20C211239_R303_CapNGS | HBVCgp4_PREC/C | INTS4//intronic | 112 | chr11 | 77629517 | + | AY123041 | 1883 | + | 11q14.1 |
| 1T | 20C211239_R303_CapNGS | HBVCgp3_X//upstream | LINC02264(dist=56611),NDST3(dist=107927)//intergenic | 150 | chr4 | 118847714 | + | AY123041 | 296 | + | 4q26 |
| 2T | 20C211240_R303_CapNGS | HBVCgp4_PREC/C | MIR4426(dist=219892),CDH8(dist=371593)//intergenic | 8 | chr16 | 61309562 | + | AY123041 | 1915 | - | 16q21 |
| 2T | 20C211240_R303_CapNGS | HBVCgp3_X//upstream | LINC02186(dist=38970),MRTFB(dist=11015)//intergenic | 10 | chr16 | 14153904 | + | AY123041 | 533 | - | 16p13.12 |
| 2T | 20C211240_R303_CapNGS | HBVCgp3_X//upstream | LINC00692(dist=344905),LRRC3B(dist=404206)//intergenic | 79 | chr3 | 26260091 | + | AY123041 | 274 | + | 3p24.2 |
| 2T | 20C211240_R303_CapNGS | HBVCgp3_X//upstream | RNF14(dist=1593),GNPDA1(dist=8786)//intergenic | 84 | chr5 | 141371450 | + | AY123041 | 505 | + | 5q31.3 |
| 2T | 20C211240_R303_CapNGS | HBVCgp2_PRES1/PRES2/S;HBVCgp1_P;HBVCgp3_X;HBVCgp4_PREC/C//upstream;downstream | ASTN2//intronic | 261 | chr9 | 119570616 | - | AY123041 | 2482 | + | 9q33.1 |
| 3T | 20C211241_R303_CapNGS | HBVCgp3_X//upstream | DNMT3L//intronic | 5 | chr21 | 45677333 | + | AY123041 | 633 | + | 21q22.3 |
| 3T | 20C211241_R303_CapNGS | HBVCgp3_X//upstream | MIR5708(dist=19175),ZBTB10(dist=224945)//intergenic | 6 | chr8 | 81172883 | + | AY123041 | 633 | + | 8q21.13 |
| 3T | 20C211241_R303_CapNGS | HBVCgp3_X//upstream | RAB9BP1(dist=216379),LINC01950(dist=1498720)//intergenic | 6 | chr5 | 104652178 | + | AY123041 | 633 | + | 5q21.3 |
| 3T | 20C211241_R303_CapNGS | HBVCgp3_X;HBVCgp4_PREC/C//upstream | TBC1D22A//intronic | 6 | chr22 | 47448433 | + | AY123041 | 1111 | + | 22q13.31 |
| 3T | 20C211241_R303_CapNGS | HBVCgp4_PREC/C | NARF(dist=8754),FOXK2(dist=20441)//intergenic | 6 | chr17 | 80457167 | + | AY123041 | 2103 | + | 17q25.3 |
| 3T | 20C211241_R303_CapNGS | HBVCgp3_X//upstream | MIR891A(dist=220573),CXorf51B(dist=561326)//intergenic | 8 | chrX | 145329963 | + | AY123041 | 633 | + | Xq27.3 |
| 3T | 20C211241_R303_CapNGS | HBVCgp3_X//upstream | CTNNBL1//intronic | 8 | chr20 | 36415993 | + | AY123041 | 633 | - | 20q11.23 |

| | | | | | | | | | | | |
|---|---|---|---|---|---|---|---|---|---|---|---|
| 3T | 20C211241_R303_CapNGS | HBVCgp3_X//upstream | BPY2(dist=55982),DAZ4(dist=67910)//intergenic | 9 | chrY | 25207592 | + | AY123041 | 638 | + | Yq11.223 |
| 3T | 20C211241_R303_CapNGS | HBVCgp2_PRES1/PRES2/S;HBVCgp1_P;HBVCgp3_X;HBVCgp4_PREC/C//upstream;downstream | UNC5D(dist=862517),KCNU1(dist=125307)//intergenic | 9 | chr8 | 36516585 | - | AY123041 | 2349 | + | 8p11.23 |
| 3T | 20C211241_R303_CapNGS | HBVCgp3_X//upstream | DIS3L2//intronic | 12 | chr2 | 232916291 | + | AY123041 | 638 | + | 2q37.1 |
| 3T | 20C211241_R303_CapNGS | HBVCgp3_X//upstream | ZNF804A(dist=114224),LOC105373782(dist=111239)//intergenic | 13 | chr2 | 185918442 | + | AY123041 | 638 | - | 2q32.1 |
| 3T | 20C211241_R303_CapNGS | HBVCgp3_X;HBVCgp4_PREC/C//upstream | AIG1//intronic | 14 | chr6 | 143507292 | + | AY123041 | 729 | + | 6q24.2 |
| 3T | 20C211241_R303_CapNGS | HBVCgp3_X//upstream | STXBP6//intronic | 14 | chr14 | 25351354 | + | AY123041 | 633 | + | 14q12 |
| 3T | 20C211241_R303_CapNGS | HBVCgp3_X//upstream | TPTE2P1(dist=102645),PABPC3(dist=25024)//intergenic | 15 | chr13 | 25645252 | + | AY123041 | 638 | + | 13q12.13 |
| 3T | 20C211241_R303_CapNGS | HBVCgp4_PREC/C | MAP1LC3B2(dist=78099),C12orf49(dist=55479)//intergenic | 15 | chr12 | 117092497 | + | AY123041 | 1879 | + | 12q24.22 |
| 3T | 20C211241_R303_CapNGS | HBVCgp3_X//upstream | SLC25A51P1(dist=735950),LOC102723883(dist=1529917)//intergenic | 17 | chr6 | 67235326 | + | AY123041 | 638 | + | 6q12 |
| 3T | 20C211241_R303_CapNGS | HBVCgp3_X//upstream | LINC01493(dist=1248404),LRRC4C(dist=210548)//intergenic | 17 | chr11 | 39925203 | + | AY123041 | 638 | - | 11p12 |
| 3T | 20C211241_R303_CapNGS | HBVCgp3_X//upstream | HNRNPC//intronic | 19 | chr14 | 21728127 | + | AY123041 | 638 | + | 14q11.2 |
| 3T | 20C211241_R303_CapNGS | HBVCgp3_X//upstream | KCNAB1//intronic | 20 | chr3 | 155882836 | + | AY123041 | 638 | + | 3q25.31 |
| 3T | 20C211241_R303_CapNGS | HBVCgp3_X//upstream | C1orf87(dist=331462),LINC01748(dist=110484)//intergenic | 22 | chr1 | 60870904 | + | AY123041 | 638 | - | 1p32.1 |
| 3T | 20C211241_R303_CapNGS | HBVCgp3_X//upstream | MAP2K4(dist=149500),LINC00670(dist=256642)//intergenic | 25 | chr17 | 12196643 | + | AY123041 | 638 | + | 17p12 |
| 3T | 20C211241_R303_CapNGS | HBVCgp3_X//upstream | SGK1//intronic | 26 | chr6 | 134540966 | + | AY123041 | 638 | + | 6q23.2 |
| 3T | 20C211241_R303_CapNGS | HBVCgp3_X//upstream | TMTC2(dist=937732),SLC6A15(dist=786894)//intergenic | 45 | chr12 | 84466376 | + | AY123041 | 638 | - | 12q21.31 |
| 3T | 20C211241_R303_CapNGS | HBVCgp3_X//upstream | ETV6//intronic | 53 | chr12 | 11994785 | + | AY123041 | 638 | + | 12p13.2 |
| 3T | 20C211241_R303_CapNGS | NONE(dist=NONE),HBVCgp3_X(dist=1080)//intergenic | RFX3-AS1(dist=131348),GLIS3(dist=1023)//intergenic | 85 | chr9 | 3823104 | - | AY123041 | 168 | + | 9p24.2 |
| 3T | 20C211241_R303_CapNGS | HBVCgp4_PREC/C//downstream | DLC1//intronic | 198 | chr8 | 13008835 | + | AY123041 | 2754 | - | 8p22 |
| 3T | 20C211241_R303_CapNGS | HBVCgp4_PREC/C | NONE(dist=NONE),LOC441666(dist=229785)//intergenic | 2227 | chr10 | 42597529 | - | AY123041 | 1800 | + | 10q11.21 |
| 3T | 20C211241_R303_CapNGS | HBVCgp4_PREC/C | NONE(dist=NONE),LOC441666(dist=229782)//intergenic | 2281 | chr10 | 42597532 | - | AY123041 | 1800 | + | 10q11.21 |
| 3T | 20C211241_R303_CapNGS | HBVCgp3_X | LRP12(dist=692178),ZFPM2(dist=37236)//intergenic | 3491 | chr8 | 106293430 | - | AY123041 | 1386 | + | 8q23.1 |
| 3T | 20C211241_R303_CapNGS | HBVCgp3_X | LRP12(dist=692177),ZFPM2(dist=37237)//intergenic | 3557 | chr8 | 106293429 | + | AY123041 | 1386 | - | 8q23.1 |
| 4T | 20C211242_R303_CapNGS | HBVBgp2_S | LOC105375787(dist=17483),LINC01300(dist=66249)//intergenic | 5 | chr8 | 142284399 | + | AF100309 | 803 | + | 8q24.3 |
| 4T | 20C211242_R303_CapNGS | HBVBgp3_X;HBVBgp4_C;HBVBgp2_S//upstream;downstream | CSNK1E;TPTEP2-CSNK1E//intronic | 5 | chr22 | 38704983 | + | AF100309 | 1218 | + | 22q13.1 |
| 4T | 20C211242_R303_CapNGS | HBVBgp3_X;HBVBgp4_C//downstream | MRPS18A(dist=9332),VEGFA(dist=73065)//intergenic | 6 | chr6 | 43664881 | + | AF100309 | 2752 | + | 6p21.1 |

| | | | | | | | | | | | |
|---|---|---|---|---|---|---|---|---|---|---|---|
| 4T | 20C211242_R303_CapNGS | HBVBgp3_X | MIR4790(dist=399209),GRM7-AS3(dist=982896)//intergenic | 6 | chr3 | 5691149 | + | AF100309 | 1616 | + | 3p26.1 |
| 4T | 20C211242_R303_CapNGS | HBVBgp2_S | MS4A13(dist=8468),LINC00301(dist=64565)//intergenic | 6 | chr11 | 60318659 | + | AF100309 | 786 | + | 11q12.2 |
| 4T | 20C211242_R303_CapNGS | HBVBgp3_X;HBVBgp4_C;HBVBgp2_S//upstream;downstream | MIR548XHG(dist=798977),LINC01683(dist=334486)//intergenic | 7 | chr21 | 20931107 | + | AF100309 | 963 | + | 21q21.1 |
| 4T | 20C211242_R303_CapNGS | HBVBgp3_X;HBVBgp4_C//downstream | NONE(dist=NONE),SNORD3P3(dist=15319)//intergenic | 7 | chr14 | 85722957 | + | AF100309 | 2782 | - | 14q31.3 |
| 4T | 20C211242_R303_CapNGS | HBVBgp4_C | CSMD1//intronic | 9 | chr8 | 3957094 | + | AF100309 | 2196 | - | 8p23.2 |
| 4T | 20C211242_R303_CapNGS | HBVBgp3_X;HBVBgp4_C;HBVBgp2_S//upstream;downstream | LINC00564(dist=861183),SLITRK1(dist=1789039)//intergenic | 10 | chr13 | 82662301 | + | AF100309 | 913 | + | 13q31.1 |
| 4T | 20C211242_R303_CapNGS | HBVBgp2_S | KCNH8//intronic | 11 | chr3 | 19412421 | + | AF100309 | 642 | - | 3p24.3 |
| 4T | 20C211242_R303_CapNGS | HBVBgp3_X;HBVBgp4_C//downstream | PPP1R37//upstream | 13 | chr19 | 45596208 | + | AF100309 | 2774 | + | 19q13.32 |
| 4T | 20C211242_R303_CapNGS | HBVBgp3_X;HBVBgp4_C//downstream | ANP32A//upstream | 18 | chr15 | 69113447 | + | AF100309 | 2821 | + | 15q23 |
| 4T | 20C211242_R303_CapNGS | HBVBgp3_X | SPAG6(dist=3492),LOC100499489(dist=14323)//intergenic | 25 | chr10 | 22710031 | - | AF100309 | 1439 | + | 10p12.2 |
| 4T | 20C211242_R303_CapNGS | HBVBgp3_X;HBVBgp4_C;HBVBgp2_S//upstream;downstream | TRDN//intronic | 35 | chr6 | 123846346 | + | AF100309 | 1054 | + | 6q22.31 |
| 4T | 20C211242_R303_CapNGS | HBVBgp3_X | CDH2(dist=373501),MIR302F(dist=1748281)//intergenic | 40 | chr18 | 26130595 | + | AF100309 | 1684 | - | 18q12.1 |
| 4T | 20C211242_R303_CapNGS | HBVBgp4_C//downstream | IGF1(dist=248461),LINC00485(dist=80259)//intergenic | 49 | chr12 | 103122802 | + | AF100309 | 3059 | - | 12q23.2 |
| 4T | 20C211242_R303_CapNGS | HBVBgp3_X | PRH1-PRR4//ncRNA_intronic | 50 | chr12 | 11036615 | + | AF100309 | 1418 | + | 12p13.2 |
| 4T | 20C211242_R303_CapNGS | HBVBgp4_C | FAM50A//intronic | 66 | chrX | 153676544 | + | AF100309 | 2185 | + | Xq28 |
| 4T | 20C211242_R303_CapNGS | HBVBgp4_C | SULT1E1(dist=32164),CSN1S1(dist=38759)//intergenic | 79 | chr4 | 70758027 | + | AF100309 | 2169 | + | 4q13.3 |
| 4T | 20C211242_R303_CapNGS | HBVBgp2_S | NRG1//intronic | 80 | chr8 | 32589256 | - | AF100309 | 644 | + | 8p12 |
| 4T | 20C211242_R303_CapNGS | HBVBgp3_X | FFAR4;RBP4//downstream | 34583 | chr10 | 95350667 | - | AF100309 | 1582 | + | 10q23.33 |
| 4T | 20C211242_R303_CapNGS | HBVBgp3_X | FFAR4;RBP4//downstream | 34583 | chr10 | 95350671 | - | AF100309 | 1582 | + | 10q23.33 |
| 4T | 20C211242_R303_CapNGS | HBVBgp3_X | FFAR4;RBP4//downstream | 34585 | chr10 | 95350673 | - | AF100309 | 1582 | + | 10q23.33 |
| 4T | 20C211242_R303_CapNGS | HBVBgp3_X | TBC1D22A(dist=131627),LOC339685(dist=38352)//intergenic | 49338 | chr22 | 47702969 | + | AF100309 | 1785 | + | 22q13.31 |
| 4T | 20C211242_R303_CapNGS | HBVBgp4_C | TBC1D22A(dist=131321),LOC339685(dist=38658)//intergenic | 49339 | chr22 | 47702663 | + | AF100309 | 2162 | + | 22q13.31 |
| 4T | 20C211242_R303_CapNGS | HBVBgp4_C | TBC1D22A(dist=131316),LOC339685(dist=38663)//intergenic | 49340 | chr22 | 47702658 | + | AF100309 | 2166 | + | 22q13.31 |
| 4T | 20C211242_R303_CapNGS | HBVBgp4_C | TBC1D22A(dist=131308),LOC339685(dist=38671)//intergenic | 49340 | chr22 | 47702650 | + | AF100309 | 2166 | + | 22q13.31 |
| 4T | 20C211242_R303_CapNGS | HBVBgp4_C | TBC1D22A(dist=131322),LOC339685(dist=38657)//intergenic | 50127 | chr22 | 47702664 | + | AF100309 | 2165 | + | 22q13.31 |
| 4T | 20C211242_R303_CapNGS | HBVBgp3_X | TBC1D22A(dist=131622),LOC339685(dist=38357)//intergenic | 50315 | chr22 | 47702964 | + | AF100309 | 1778 | + | 22q13.31 |

| | | | | | | | | | | | |
|---|---|---|---|---|---|---|---|---|---|---|---|
| 5T | 20C211243_R303_CapNGS | HBVCgp2_PRES1/PRES2/S;HBVCgp1_P;HBVCgp3_X;HBVCgp4_PREC/C//upstream;downstream | COL14A1//intronic | 5 | chr8 | 121355490 | - | AY123041 | 2328 | + | 8q24.12 |
| 5T | 20C211243_R303_CapNGS | HBVCgp3_X//upstream | KCNH7(dist=27471),FIGN(dist=736374)//intergenic | 6 | chr2 | 163722748 | + | AY123041 | 272 | + | 2q24.3 |
| 5T | 20C211243_R303_CapNGS | HBVCgp2_PRES1/PRES2/S;HBVCgp1_P;HBVCgp3_X;HBVCgp4_PREC/C//upstream;downstream | VPS33B//intronic | 6 | chr15 | 91559393 | + | AY123041 | 2366 | + | 15q26.1 |
| 5T | 20C211243_R303_CapNGS | HBVCgp3_X//upstream | MKI67(dist=50609),LINC01163(dist=108918)//intergenic | 8 | chr10 | 129975296 | + | AY123041 | 457 | - | 10q26.2 |
| 5T | 20C211243_R303_CapNGS | HBVCgp2_PRES1/PRES2/S;HBVCgp1_P;HBVCgp3_X;HBVCgp4_PREC/C//upstream;downstream | RBFOX2//intronic | 12 | chr22 | 36253565 | + | AY123041 | 2397 | + | 22q12.3 |
| 5T | 20C211243_R303_CapNGS | HBVCgp3_X//upstream | MIR4682(dist=54871),RPL21(dist=341196)//intergenic | 14 | chr10 | 121772975 | + | AY123041 | 319 | - | 10q26.12 |
| 5T | 20C211243_R303_CapNGS | NONE(dist=NONE),HBVCgp3_X(dist=1231)//intergenic | KCNJ2(dist=70528),CASC17(dist=847204)//intergenic | 16 | chr17 | 68246711 | + | AY123041 | 17 | + | 17q24.3 |
| 5T | 20C211243_R303_CapNGS | HBVCgp3_X//upstream | EML1//intronic | 17 | chr14 | 100318409 | + | AY123041 | 256 | - | 14q32.2 |
| 5T | 20C211243_R303_CapNGS | HBVCgp2_PRES1/PRES2/S;HBVCgp1_P;HBVCgp3_X;HBVCgp4_PREC/C//upstream;downstream | KDM2B//intronic | 19 | chr12 | 121984795 | + | AY123041 | 2386 | + | 12q24.31 |
| 5T | 20C211243_R303_CapNGS | HBVCgp3_X;HBVCgp4_PREC/C | KIF4B(dist=1171016),SGCD(dist=185057)//intergenic | 20 | chr5 | 155568710 | + | AY123041 | 1700 | + | 5q33.2 |
| 5T | 20C211243_R303_CapNGS | NONE(dist=NONE),HBVCgp3_X(dist=1151)//intergenic | B3GNT2//intronic | 21 | chr2 | 62440524 | + | AY123041 | 97 | - | 2p15 |
| 5T | 20C211243_R303_CapNGS | HBVCgp3_X//upstream | MYO5B//intronic | 26 | chr18 | 47433833 | + | AY123041 | 461 | - | 18q21.1 |
| 5T | 20C211243_R303_CapNGS | HBVCgp2_PRES1/PRES2/S;HBVCgp1_P;HBVCgp3_X;HBVCgp4_PREC/C//upstream;downstream | NCAM1//intronic | 28 | chr11 | 113026139 | + | AY123041 | 2494 | - | 11q23.2 |
| 5T | 20C211243_R303_CapNGS | HBVCgp2_PRES1/PRES2/S;HBVCgp1_P;HBVCgp3_X;HBVCgp4_PREC/C//upstream;downstream | LINC01493(dist=1063330),LRRC4C(dist=395622)//intergenic | 30 | chr11 | 39740129 | - | AY123041 | 2328 | + | 11p12 |
| 5T | 20C211243_R303_CapNGS | HBVCgp2_PRES1/PRES2/S;HBVCgp1_P;HBVCgp3_X;HBVCgp4_PREC/C//upstream;downstream | SPATA33(dist=7048),CDK10(dist=9183)//intergenic | 38 | chr16 | 89743914 | + | AY123041 | 2494 | - | 16q24.3 |
| 5T | 20C211243_R303_CapNGS | HBVCgp2_PRES1/PRES2/S;HBVCgp1_P;HBVCgp3_X;HBVCgp4_PREC/C//upstream;downstream | SYAP1//intronic | 45 | chrX | 16766113 | + | AY123041 | 2494 | - | Xp22.2 |
| 5T | 20C211243_R303_CapNGS | HBVCgp3_X;HBVCgp4_PREC/C | ADGRG4(dist=21939),BRS3(dist=49118)//intergenic | 75 | chrX | 135520988 | + | AY123041 | 1698 | - | Xq26.3 |

| | | | | | | | | | | | |
|---|---|---|---|---|---|---|---|---|---|---|---|
| 5T | 20C211243_R303_CapNGS | HBVCgp4_PREC/C | UBE2NL(dist=1113085),SPANXN1(dist=247581)//intergenic | 97 | chrX | 144081442 | + | AY123041 | 1765 | + | Xq27.3 |
| 5T | 20C211243_R303_CapNGS | HBVCgp2_PRES1/PRES2/S;HBVCgp1_P;HBVCgp3_X;HBVCgp4_PREC/C//upstream;downstream | ZNF658B(dist=119971),GLIDR(dist=218999)//intergenic | 124 | chr9 | 41729515 | + | AY123041 | 2494 | - | 9p12 |
| 5T | 20C211243_R303_CapNGS | HBVCgp2_PRES1/PRES2/S;HBVCgp1_P;HBVCgp3_X;HBVCgp4_PREC/C//upstream;downstream | LOC642929(dist=327402),FAM74A7(dist=135487)//intergenic | 127 | chr9 | 43472886 | + | AY123041 | 2494 | + | 9p12 |
| 5T | 20C211243_R303_CapNGS | HBVCgp2_PRES1/PRES2/S;HBVCgp1_P;HBVCgp3_X;HBVCgp4_PREC/C//upstream;downstream | DEPDC4//intronic | 151 | chr12 | 100641095 | + | AY123041 | 2494 | + | 12q23.1 |
| 5T | 20C211243_R303_CapNGS | HBVCgp2_PRES1/PRES2/S;HBVCgp1_P;HBVCgp3_X;HBVCgp4_PREC/C//upstream;downstream | EDIL3(dist=376015),NBPF22P(dist=1521549)//intergenic | 166 | chr5 | 84056713 | + | AY123041 | 2494 | + | 5q14.3 |
| 5T | 20C211243_R303_CapNGS | HBVCgp3_X | POTEA(dist=604063),NONE(dist=NONE)//intergenic | 5750 | chr8 | 43822393 | + | AY123041 | 1682 | - | 8p11.1 |
| 6T | 20C211244_R303_CapNGS | HBVCgp3_X//upstream | QRFPR//intronic | 5 | chr4 | 122269811 | - | AY123041 | 420 | + | 4q27 |
| 6T | 20C211244_R303_CapNGS | NONE(dist=NONE),HBVCgp3_X(dist=1109)//intergenic | CNTNAP3//intronic | 7 | chr9 | 39134146 | - | AY123041 | 139 | + | 9p13.1 |
| 6T | 20C211244_R303_CapNGS | HBVCgp3_X//upstream | METTL21C(dist=13534),CCDC168(dist=21312)//intergenic | 7 | chr13 | 103360405 | + | AY123041 | 545 | - | 13q33.1 |
| 6T | 20C211244_R303_CapNGS | HBVCgp2_PRES1/PRES2/S;HBVCgp1_P;HBVCgp3_X;HBVCgp4_PREC/C//upstream;downstream | KDM2B//intronic | 63 | chr12 | 121984790 | + | AY123041 | 2386 | + | 12q24.31 |
| 6T | 20C211244_R303_CapNGS | HBVCgp3_X | POTEA(dist=604062),NONE(dist=NONE)//intergenic | 67 | chr8 | 43822390 | - | AY123041 | 1682 | + | 8p11.1 |
| 6T | 20C211244_R303_CapNGS | HBVCgp3_X | NEDD4L//downstream | 229 | chr18 | 56068977 | + | AY123041 | 1680 | - | 18q21.31 |
| 6T | 20C211244_R303_CapNGS | HBVCgp3_X | POTEA(dist=609671),NONE(dist=NONE)//intergenic | 876 | chr8 | 43827999 | + | AY123041 | 1682 | - | 8p11.1 |
| 6T | 20C211244_R303_CapNGS | HBVCgp3_X | POTEA(dist=604063),NONE(dist=NONE)//intergenic | 1675 | chr8 | 43822391 | + | AY123041 | 1682 | - | 8p11.1 |
| 7T | 20C211245_R303_CapNGS | HBVCgp3_X;HBVCgp4_PREC/C | ZNF292//intronic | 985 | chr6 | 87941031 | + | AY123041 | 1697 | + | 6q14.3 |
| 7T | 20C211245_R303_CapNGS | HBVCgp3_X | EMBP1(dist=171168),NONE(dist=NONE)//intergenic | 5355 | chr1 | 121484854 | + | AY123041 | 1633 | + | 1p11.2 |
| 8T | 20C211246_R303_CapNGS | HBVCgp2_PRES1/PRES2/S;HBVCgp1_P;HBVCgp3_X;HBVCgp4_PREC/C//upstream;downstream | LOC100130964//ncRNA_intronic | 6 | chr8 | 39435864 | + | AY123041 | 2437 | - | 8p11.22 |
| 8T | 20C211246_R303_CapNGS | HBVCgp4_PREC/C | SASH1//intronic | 7 | chr6 | 148799836 | + | AY123041 | 1860 | + | 6q24.3 |
| 8T | 20C211246_R303_CapNGS | HBVCgp3_X;HBVCgp4_PREC/C//upstream | RALGPS2//intronic | 7 | chr1 | 178725851 | + | AY123041 | 709 | - | 1q25.2 |
| 8T | 20C211246_R303_CapNGS | HBVCgp3_X//upstream | XDH(dist=78899),SRD5A2(dist=31043)//intergenic | 8 | chr2 | 31716507 | - | AY123041 | 363 | + | 2p23.1 |

| | | | | | | | | | | | |
|---|---|---|---|---|---|---|---|---|---|---|---|
| 8T | 20C211246_R303_CapNGS | HBVCgp4_PREC/C | MIR548X2(dist=111976),MIR4704(dist=139845)//intergenic | 11 | chr13 | 66652537 | + | AY123041 | 2082 | - | 13q21.32 |
| 8T | 20C211246_R303_CapNGS | NONE(dist=NONE),HBVCgp3_X(dist=1038)//intergenic | MSI2//intronic | 12 | chr17 | 55350477 | + | AY123041 | 210 | + | 17q22 |
| 8T | 20C211246_R303_CapNGS | HBVCgp3_X;HBVCgp4_PREC/C//upstream | LINC01671(dist=36516),PDE9A(dist=2010)//intergenic | 56 | chr21 | 44071721 | + | AY123041 | 1012 | + | 21q22.3 |
| 8T | 20C211246_R303_CapNGS | HBVCgp4_PREC/C | IL7//intronic | 59 | chr8 | 79680850 | + | AY123041 | 2252 | + | 8q21.12 |
| 8T | 20C211246_R303_CapNGS | HBVCgp4_PREC/C | C7orf66(dist=43952),EIF3IP1(dist=1030688)//intergenic | 83 | chr7 | 108568596 | + | AY123041 | 2292 | + | 7q31.1 |
| 8T | 20C211246_R303_CapNGS | HBVCgp3_X;HBVCgp4_PREC/C//upstream | POTEA(dist=604147),NONE(dist=NONE)//intergenic | 173 | chr8 | 43822475 | + | AY123041 | 822 | + | 8p11.1 |
| 8T | 20C211246_R303_CapNGS | HBVCgp4_PREC/C | LINC00910//ncRNA_intronic | 212 | chr17 | 41453198 | + | AY123041 | 1885 | - | 17q21.31 |
| 8T | 20C211246_R303_CapNGS | HBVCgp3_X//upstream | LOC102724708//ncRNA_intronic | 281 | chr8 | 68391159 | + | AY123041 | 356 | + | 8q13.2 |
| 8T | 20C211246_R303_CapNGS | HBVCgp3_X | MAP3K7(dist=31493),MIR4643(dist=903094)//intergenic | 300 | chr6 | 91328284 | + | AY123041 | 1402 | + | 6q15 |
| 8T | 20C211246_R303_CapNGS | HBVCgp3_X//upstream | ZNF831//downstream | 334 | chr20 | 57834728 | - | AY123041 | 476 | + | 20q13.32 |
| 8T | 20C211246_R303_CapNGS | HBVCgp3_X;HBVCgp4_PREC/C | FN1//exonic//FN1:NM_001306129:exon19:c.2817,FN1:NM_001306130:exon19:c.2817,FN1:NM_001306131:exon19:c.2817,FN1:NM_001306132:exon19:c.2817,FN1:NM_001365517:exon19:c.2817,FN1:NM_001365518:exon19:c.2817,FN1:NM_001365519:exon19:c.2817,FN1:NM_001365520:exon19:c.2817,FN1:NM_001365521:exon19:c.2817,FN1:NM_001365522:exon19:c.2817,FN1:NM_001365523:exon19:c.2817,FN1:NM_001365524:exon19:c.2817,FN1:NM_002026:exon19:c.2817,FN1:NM_212474:exon19:c.2817,FN1:NM_212476:exon19:c.2817,FN1:NM_212478:exon19:c.2817,FN1:NM_212482:exon19:c.2817 | 384 | chr2 | 216271130 | - | AY123041 | 1696 | + | 2q35 |
| 8T | 20C211246_R303_CapNGS | HBVCgp2_PRES1/PRES2/S;HBVCgp3_X;HBVCgp4_PREC/C//upstream;downstream | KIT(dist=130860),KDR(dist=206907)//intergenic | 796 | chr4 | 55737741 | - | AY123041 | 2602 | + | 4q12 |
| 8T | 20C211246_R303_CapNGS | HBVCgp3_X;HBVCgp4_PREC/C//upstream | RSPO3//intronic | 4028 | chr6 | 127441231 | + | AY123041 | 934 | + | 6q22.33 |
| 8T | 20C211246_R303_CapNGS | HBVCgp3_X;HBVCgp4_PREC/C//upstream | RSPO3//intronic | 4106 | chr6 | 127441229 | + | AY123041 | 934 | + | 6q22.33 |
| 8T | 20C211246_R303_CapNGS | HBVCgp4_PREC/C | LINC01309(dist=1265596),DAOA-AS1(dist=765775)//intergenic | 4150 | chr13 | 105345631 | - | AY123041 | 2030 | + | 13q33.2 |
| 8T | 20C211246_R303_CapNGS | HBVCgp4_PREC/C | LINC01309(dist=1265597),DAOA-AS1(dist=765774)//intergenic | 4253 | chr13 | 105345632 | - | AY123041 | 2030 | + | 13q33.2 |
| 8T | 20C211246_R303_CapNGS | HBVCgp3_X;HBVCgp4_PREC/C//upstream | MAPK3(dist=22445),CORO1A(dist=37656)//intergenic | 4390 | chr16 | 30157075 | + | AY123041 | 866 | + | 16p11.2 |
| 8T | 20C211246_R303_CapNGS | HBVCgp3_X | LINC02073(dist=36323),CA10(dist=148188)//intergenic | 5562 | chr17 | 49559486 | - | AY123041 | 1351 | + | 17q21.33 |
| 8T | 20C211246_R303_CapNGS | HBVCgp3_X//upstream | ITPR2//intronic | 8929 | chr12 | 26604939 | + | AY123041 | 266 | - | 12p11.23 |
| 8T | 20C211246_R303_CapNGS | HBVCgp4_PREC/C | LINC02303(dist=48968),LINC00871(dist=299589)//intergenic | 14266 | chr14 | 46233773 | + | AY123041 | 2074 | + | 14q21.2 |

| | | | | | | | | | | | |
|---|---|---|---|---|---|---|---|---|---|---|---|
| 8T | 20C211246_R303_CapNGS | HBVCgp4_PREC/C | LINC02303(dist=48964),LINC00871(dist=299593)//intergenic | 14266 | chr14 | 46233769 | + | AY123041 | 2074 | + | 14q21.2 |
| 8T | 20C211246_R303_CapNGS | HBVCgp4_PREC/C | LINC02303(dist=48969),LINC00871(dist=299588)//intergenic | 14412 | chr14 | 46233774 | + | AY123041 | 2074 | + | 14q21.2 |
| 8T | 20C211246_R303_CapNGS | HBVCgp3_X;HBVCgp4_PREC/C | ZNF292//intronic | 60682 | chr6 | 87941044 | + | AY123041 | 1692 | + | 6q14.3 |
| 8T | 20C211246_R303_CapNGS | HBVCgp3_X;HBVCgp4_PREC/C | ZNF292//intronic | 60683 | chr6 | 87941034 | + | AY123041 | 1693 | + | 6q14.3 |
| 8T | 20C211246_R303_CapNGS | HBVCgp3_X;HBVCgp4_PREC/C | ZNF292//intronic | 60683 | chr6 | 87941043 | + | AY123041 | 1692 | + | 6q14.3 |
| 8T | 20C211246_R303_CapNGS | HBVCgp3_X;HBVCgp4_PREC/C | ZNF292//intronic | 61017 | chr6 | 87941012 | + | AY123041 | 1710 | + | 6q14.3 |
| 8T | 20C211246_R303_CapNGS | HBVCgp3_X;HBVCgp4_PREC/C | ZNF292//intronic | 61163 | chr6 | 87941036 | + | AY123041 | 1692 | + | 6q14.3 |
| 9T | 20C211247_R303_CapNGS | HBVCgp3_X//upstream | NONE(dist=NONE),LOC441666(dist=299333)//intergenic | 5 | chr10 | 42527981 | + | AY123041 | 426 | + | 10q11.21 |
| 9T | 20C211247_R303_CapNGS | NONE(dist=NONE),HBVCgp3_X(dist=1199)//intergenic | ALX3//intronic | 13 | chr1 | 110605179 | - | AY123041 | 49 | + | 1p13.3 |
| 9T | 20C211247_R303_CapNGS | HBVCgp3_X | TERT//upstream | 27 | chr5 | 1295626 | + | AY123041 | 1501 | + | 5p15.33 |
| 9T | 20C211247_R303_CapNGS | HBVCgp4_PREC/C | KMT2B//exonic//KMT2B:NM_014727:exon3:c.2421 | 112 | chr19 | 36212670 | + | AY123041 | 1808 | + | 19q13.12 |
| 9T | 20C211247_R303_CapNGS | HBVCgp3_X;HBVCgp4_PREC/C | FN1//intronic | 186 | chr2 | 216254696 | + | AY123041 | 1696 | - | 2q35 |
| 9T | 20C211247_R303_CapNGS | HBVCgp3_X | FN1//exonic//FN1:NM_001306129:exon20:c.3073,FN1:NM_001306130:exon20:c.3073,FN1:NM_001306131:exon20:c.3073,FN1:NM_001306132:exon20:c.3073,FN1:NM_001365517:exon20:c.3073,FN1:NM_001365518:exon20:c.3073,FN1:NM_001365519:exon20:c.3073,FN1:NM_001365520:exon20:c.3073,FN1:NM_001365521:exon20:c.3073,FN1:NM_001365522:exon20:c.3073,FN1:NM_001365523:exon20:c.3073,FN1:NM_001365524:exon20:c.3073,FN1:NM_002026:exon20:c.3073,FN1:NM_212474:exon20:c.3073,FN1:NM_212476:exon20:c.3073,FN1:NM_212478:exon20:c.3073,FN1:NM_212482:exon20:c.3073 | 1317 | chr2 | 216269292 | - | AY123041 | 1681 | + | 2q35 |
| 10T | 20C211248_R303_CapNGS | HBVBgp3_X | TMEM43//UTR3 | 5 | chr3 | 14185035 | - | AF100309 | 1499 | + | 3p25.1 |
| 10T | 20C211248_R303_CapNGS | HBVBgp3_X;HBVBgp4_C;HBVBgp2_S//upstream;downstream | ABCC1//intronic | 5 | chr16 | 16154338 | + | AF100309 | 978 | - | 16p13.11 |
| 10T | 20C211248_R303_CapNGS | HBVBgp1_P;HBVBgp3_X;HBVBgp4_C;HBVBgp2_S//upstream;downstream | LOC105377975//ncRNA_intronic | 6 | chr6 | 120163277 | + | AF100309 | 1312 | + | 6q22.31 |
| 10T | 20C211248_R303_CapNGS | HBVBgp3_X | ELOVL5(dist=39072),GCLC(dist=109091)//intergenic | 6 | chr6 | 53253049 | + | AF100309 | 1708 | + | 6p12.1 |
| 10T | 20C211248_R303_CapNGS | HBVBgp4_C | ARPP21(dist=451058),STAC(dist=134933)//intergenic | 6 | chr3 | 36287046 | + | AF100309 | 2365 | - | 3p22.3 |
| 10T | 20C211248_R303_CapNGS | HBVBgp2_S | GRM7-AS3//ncRNA_intronic | 7 | chr3 | 6711433 | + | AF100309 | 774 | + | 3p26.1 |

| | | | | | | | | | | | |
|---|---|---|---|---|---|---|---|---|---|---|---|
| 10T | 20C211248_R303_CapNGS | HBVBgp2_S | GPR6(dist=1528),WASF1(dist=117571)//intergenic | 15 | chr6 | 110303451 | - | AF100309 | 288 | + | 6q21 |
| 10T | 20C211248_R303_CapNGS | HBVBgp2_S | CMAHP(dist=53434),LOC101928663(dist=53092)//intergenic | 17 | chr6 | 25192054 | - | AF100309 | 418 | + | 6p22.3 |
| 10T | 20C211248_R303_CapNGS | HBVBgp2_S//upstream | MT-TS1(dist=8046),NONE(dist=NONE)//intergenic | 88 | chrM | 15561 | + | AF100309 | 126 | + | |
| 10T | 20C211248_R303_CapNGS | HBVBgp2_S | HNF1A//intronic | 95 | chr12 | 121418255 | + | AF100309 | 554 | - | 12q24.31 |
| 10T | 20C211248_R303_CapNGS | HBVBgp1_P;HBVBgp4_C;HBVBgp3_X//upstream;downstream | NAMPT(dist=218425),CCDC71L(dist=150953)//intergenic | 138 | chr7 | 106143854 | - | AF100309 | 1901 | + | 7q22.3 |
| 10T | 20C211248_R303_CapNGS | HBVBgp2_S | LINC02353(dist=646426),LOC101928622(dist=896693)//intergenic | 153 | chr4 | 33001268 | + | AF100309 | 402 | - | 4p15.1 |
| 10T | 20C211248_R303_CapNGS | HBVBgp3_X | LINC01163(dist=256688),LINC02667(dist=338419)//intergenic | 212 | chr10 | 130372678 | - | AF100309 | 1470 | + | 10q26.2 |
| 10T | 20C211248_R303_CapNGS | HBVBgp3_X | TMEM64(dist=88031),NECAB1(dist=57661)//intergenic | 591 | chr8 | 91746342 | + | AF100309 | 1699 | + | 8q21.3 |
| 10T | 20C211248_R303_CapNGS | HBVBgp3_X | TMEM64(dist=88032),NECAB1(dist=57660)//intergenic | 6925 | chr8 | 91746343 | + | AF100309 | 1694 | + | 8q21.3 |
| 10T | 20C211248_R303_CapNGS | HBVBgp3_X | TMEM64(dist=88036),NECAB1(dist=57656)//intergenic | 7132 | chr8 | 91746347 | + | AF100309 | 1699 | + | 8q21.3 |
| 10T | 20C211248_R303_CapNGS | HBVBgp3_X | TMEM64(dist=88034),NECAB1(dist=57658)//intergenic | 7132 | chr8 | 91746345 | + | AF100309 | 1699 | + | 8q21.3 |
| 10T | 20C211248_R303_CapNGS | HBVBgp3_X | TMEM64(dist=88033),NECAB1(dist=57659)//intergenic | 7133 | chr8 | 91746344 | + | AF100309 | 1699 | + | 8q21.3 |
| 10T | 20C211248_R303_CapNGS | HBVBgp1_P;HBVBgp3_X;HBVBgp4_C//downstream | ST8SIA4(dist=617755),SLCO4C1(dist=712951)//intergenic | 14090 | chr5 | 100856741 | + | AF100309 | 2455 | - | 5q21.1 |
| 10T | 20C211248_R303_CapNGS | HBVBgp1_P;HBVBgp3_X;HBVBgp4_C//downstream | ST8SIA4(dist=617759),SLCO4C1(dist=712947)//intergenic | 14099 | chr5 | 100856745 | + | AF100309 | 2457 | - | 5q21.1 |
| 10T | 20C211248_R303_CapNGS | HBVBgp3_X | SGCD//intronic | 15765 | chr5 | 156008349 | - | AF100309 | 1746 | + | 5q33.3 |
| 10T | 20C211248_R303_CapNGS | HBVBgp3_X | SGCD//intronic | 15765 | chr5 | 156008351 | - | AF100309 | 1746 | + | 5q33.3 |
| 10T | 20C211248_R303_CapNGS | HBVBgp3_X | SGCD//intronic | 15766 | chr5 | 156008347 | - | AF100309 | 1746 | + | 5q33.3 |
| 10T | 20C211248_R303_CapNGS | HBVBgp4_C | NDUFS8//downstream | 27919 | chr11 | 67804899 | + | AF100309 | 2194 | + | 11q13.2 |
| 10T | 20C211248_R303_CapNGS | HBVBgp4_C | NDUFS8//downstream | 27919 | chr11 | 67804906 | + | AF100309 | 2194 | + | 11q13.2 |
| 10T | 20C211248_R303_CapNGS | HBVBgp4_C | NDUFS8//downstream | 27920 | chr11 | 67804912 | + | AF100309 | 2196 | + | 11q13.2 |
| 10T | 20C211248_R303_CapNGS | HBVBgp4_C | NDUFS8//downstream | 27920 | chr11 | 67804908 | + | AF100309 | 2192 | + | 11q13.2 |
| 10T | 20C211248_R303_CapNGS | HBVBgp4_C | NDUFS8//downstream | 27921 | chr11 | 67804895 | + | AF100309 | 2192 | + | 11q13.2 |
| 10T | 20C211248_R303_CapNGS | HBVBgp3_X | NDUFS8//downstream | 27922 | chr11 | 67804966 | + | AF100309 | 1775 | + | 11q13.2 |
| 10T | 20C211248_R303_CapNGS | HBVBgp4_C | NDUFS8//downstream | 27927 | chr11 | 67804896 | + | AF100309 | 2194 | + | 11q13.2 |
| 10T | 20C211248_R303_CapNGS | HBVBgp3_X | NDUFS8//downstream | 28522 | chr11 | 67804961 | + | AF100309 | 1775 | + | 11q13.2 |
| 10T | 20C211248_R303_CapNGS | HBVBgp4_C | NDUFS8//downstream | 28583 | chr11 | 67804913 | + | AF100309 | 2192 | + | 11q13.2 |
| 10T | 20C211248_R303_CapNGS | HBVBgp3_X | LYPLA2//intronic | 45731 | chr1 | 24120665 | + | AF100309 | 1834 | + | 1p36.11 |
| 10T | 20C211248_R303_CapNGS | HBVBgp3_X | LYPLA2//intronic | 45731 | chr1 | 24120667 | + | AF100309 | 1834 | + | 1p36.11 |
| 10T | 20C211248_R303_CapNGS | HBVBgp2_S | LYPLA2//exonic//LYPLA2:NM_007260:exon8:c.447 | 45734 | chr1 | 24120791 | + | AF100309 | 740 | + | 1p36.11 |
| 10T | 20C211248_R303_CapNGS | HBVBgp2_S | LYPLA2//intronic | 45735 | chr1 | 24120703 | + | AF100309 | 738 | + | 1p36.11 |
| 10T | 20C211248_R303_CapNGS | HBVBgp2_S | LYPLA2//intronic | 45735 | chr1 | 24120705 | + | AF100309 | 738 | + | 1p36.11 |
| 10T | 20C211248_R303_CapNGS | HBVBgp3_X | LYPLA2//intronic | 46079 | chr1 | 24120666 | + | AF100309 | 1834 | + | 1p36.11 |
| 10T | 20C211248_R303_CapNGS | HBVBgp2_S | LYPLA2//intronic | 46518 | chr1 | 24120702 | + | AF100309 | 733 | + | 1p36.11 |

| | | | | | | | | | | | |
|---|---|---|---|---|---|---|---|---|---|---|---|
| 10T | 20C211248_R303_CapNGS | HBVBgp3_X | LYPLA2//intronic | 46754 | chr1 | 24120656 | + | AF100309 | 1834 | + | 1p36.11 |
| 10T | 20C211248_R303_CapNGS | HBVBgp3_X | LYPLA2//intronic | 46754 | chr1 | 24120664 | + | AF100309 | 1834 | + | 1p36.11 |
| 10T | 20C211248_R303_CapNGS | HBVBgp3_X | LYPLA2//intronic | 46755 | chr1 | 24120663 | + | AF100309 | 1834 | + | 1p36.11 |
| 11T | 20C211249_R303_CapNGS | HBVBgp1_P;HBVBgp3_X;HBVBgp4_C//downstream | IL9//downstream | 5 | chr5 | 135227831 | - | AF100309 | 2528 | + | 5q31.1 |
| 11T | 20C211249_R303_CapNGS | HBVBgp4_C//downstream | LINC02130(dist=62470),PARN(dist=2965)//intergenic | 8 | chr16 | 14526593 | + | AF100309 | 3065 | - | 16p13.12 |
| 11T | 20C211249_R303_CapNGS | HBVBgp4_C | MAP2K6//intronic | 12 | chr17 | 67486185 | + | AF100309 | 1957 | - | 17q24.3 |
| 11T | 20C211249_R303_CapNGS | HBVBgp4_C | KCNJ3//intronic | 18 | chr2 | 155697188 | + | AF100309 | 1924 | + | 2q24.1 |
| 11T | 20C211249_R303_CapNGS | HBVBgp2_S | NAP1L1(dist=72634),LNCOG(dist=102564)//intergenic | 30 | chr12 | 76551099 | + | AF100309 | 741 | - | 12q21.2 |
| 11T | 20C211249_R303_CapNGS | HBVBgp4_C//downstream | VIP(dist=189807),FBXO5(dist=20970)//intergenic | 68 | chr6 | 153270702 | + | AF100309 | 2881 | + | 6q25.2 |
| 11T | 20C211249_R303_CapNGS | HBVBgp2_S | DSC1(dist=122005),DSG1(dist=33299)//intergenic | 81 | chr18 | 28864824 | + | AF100309 | 580 | - | 18q12.1 |
| 11T | 20C211249_R303_CapNGS | HBVBgp3_X | POTEA(dist=299576),NONE(dist=NONE)//intergenic | 46009 | chr8 | 43517904 | + | AF100309 | 1565 | - | 8p11.1 |
| 11T | 20C211249_R303_CapNGS | HBVBgp3_X | POTEA(dist=299575),NONE(dist=NONE)//intergenic | 46009 | chr8 | 43517903 | + | AF100309 | 1565 | - | 8p11.1 |
| 11T | 20C211249_R303_CapNGS | HBVBgp3_X | POTEA(dist=299573),NONE(dist=NONE)//intergenic | 46009 | chr8 | 43517901 | + | AF100309 | 1565 | - | 8p11.1 |
| 11T | 20C211249_R303_CapNGS | HBVBgp3_X | POTEA(dist=299579),NONE(dist=NONE)//intergenic | 46011 | chr8 | 43517907 | + | AF100309 | 1566 | - | 8p11.1 |
| 11T | 20C211249_R303_CapNGS | HBVBgp3_X | POTEA(dist=299574),NONE(dist=NONE)//intergenic | 47079 | chr8 | 43517902 | + | AF100309 | 1565 | - | 8p11.1 |
| 11T | 20C211249_R303_CapNGS | HBVBgp3_X | POTEA(dist=299583),NONE(dist=NONE)//intergenic | 47531 | chr8 | 43517911 | - | AF100309 | 1566 | + | 8p11.1 |
| 11T | 20C211249_R303_CapNGS | HBVBgp3_X | POTEA(dist=299580),NONE(dist=NONE)//intergenic | 47531 | chr8 | 43517908 | - | AF100309 | 1566 | + | 8p11.1 |
| 11T | 20C211249_R303_CapNGS | HBVBgp3_X | POTEA(dist=299578),NONE(dist=NONE)//intergenic | 47531 | chr8 | 43517906 | - | AF100309 | 1566 | + | 8p11.1 |
| 11T | 20C211249_R303_CapNGS | HBVBgp3_X | TRIML1(dist=198115),LINC01060(dist=109723)//intergenic | 48728 | chr4 | 189267009 | + | AF100309 | 1822 | + | 4q35.2 |
| 11T | 20C211249_R303_CapNGS | HBVBgp3_X | TRIML1(dist=198113),LINC01060(dist=109725)//intergenic | 48728 | chr4 | 189267007 | + | AF100309 | 1822 | + | 4q35.2 |
| 11T | 20C211249_R303_CapNGS | HBVBgp3_X | TRIML1(dist=198112),LINC01060(dist=109726)//intergenic | 48731 | chr4 | 189267006 | + | AF100309 | 1822 | + | 4q35.2 |
| 11T | 20C211249_R303_CapNGS | HBVBgp4_C//downstream | KMT2B//exonic//KMT2B:NM_014727:exon3:c.1510 | 75519 | chr19 | 36211759 | + | AF100309 | 3002 | + | 19q13.12 |
| 11T | 20C211249_R303_CapNGS | HBVBgp4_C//downstream | KMT2B//exonic//KMT2B:NM_014727:exon3:c.1505 | 75523 | chr19 | 36211754 | + | AF100309 | 3002 | + | 19q13.12 |
| 11T | 20C211249_R303_CapNGS | HBVBgp4_C//downstream | KMT2B//exonic//KMT2B:NM_014727:exon3:c.1509 | 76869 | chr19 | 36211758 | + | AF100309 | 3002 | + | 19q13.12 |
| 11T | 20C211249_R303_CapNGS | HBVBgp3_X | LINC01630(dist=338134),DCC(dist=439594)//intergenic | 139161 | chr18 | 49426973 | + | AF100309 | 1823 | + | 18q21.2 |
| 11T | 20C211249_R303_CapNGS | HBVBgp3_X | LINC01630(dist=338133),DCC(dist=439595)//intergenic | 139161 | chr18 | 49426972 | + | AF100309 | 1823 | + | 18q21.2 |
| 11T | 20C211249_R303_CapNGS | HBVBgp3_X | LINC01630(dist=338131),DCC(dist=439597)//intergenic | 139162 | chr18 | 49426970 | + | AF100309 | 1823 | + | 18q21.2 |

| | | | | | | | | | | | |
|---|---|---|---|---|---|---|---|---|---|---|---|
| 11T | 20C211249_R303_CapNGS | HBVBgp3_X | LINC01630(dist=338699),DCC(dist=439029)//intergenic | 139162 | chr18 | 49427538 | + | AF100309 | 1802 | + | 18q21.2 |
| 11T | 20C211249_R303_CapNGS | HBVBgp3_X | LINC01630(dist=338697),DCC(dist=439031)//intergenic | 139163 | chr18 | 49427536 | + | AF100309 | 1802 | + | 18q21.2 |
| 11T | 20C211249_R303_CapNGS | HBVBgp3_X | LINC01630(dist=338703),DCC(dist=439025)//intergenic | 139164 | chr18 | 49427542 | + | AF100309 | 1809 | + | 18q21.2 |
| 11T | 20C211249_R303_CapNGS | HBVBgp3_X | LINC01630(dist=338696),DCC(dist=439032)//intergenic | 139171 | chr18 | 49427535 | + | AF100309 | 1802 | + | 18q21.2 |
| 11T | 20C211249_R303_CapNGS | HBVBgp3_X | LINC01630(dist=338135),DCC(dist=439593)//intergenic | 140433 | chr18 | 49426974 | + | AF100309 | 1823 | + | 18q21.2 |
| 11T | 20C211249_R303_CapNGS | HBVBgp3_X | LINC01630(dist=338694),DCC(dist=439034)//intergenic | 141120 | chr18 | 49427533 | + | AF100309 | 1802 | + | 18q21.2 |
| 11T | 20C211249_R303_CapNGS | HBVBgp3_X | LINC01630(dist=338695),DCC(dist=439033)//intergenic | 141386 | chr18 | 49427534 | + | AF100309 | 1812 | + | 18q21.2 |
| 12T | 20C211250_R303_CapNGS | HBVCgp3_X//upstream | ATP9B(dist=1646),NFATC1(dist=16002)//intergenic | 5 | chr18 | 77139928 | + | AY123041 | 349 | + | 18q23 |
| 12T | 20C211250_R303_CapNGS | HBVCgp3_X//upstream | ZNF652//intronic | 5 | chr17 | 47427060 | + | AY123041 | 328 | - | 17q21.33 |
| 12T | 20C211250_R303_CapNGS | HBVCgp3_X//upstream | ITGA2B//downstream | 5 | chr17 | 42448935 | + | AY123041 | 301 | - | 17q21.31 |
| 12T | 20C211250_R303_CapNGS | HBVCgp3_X//upstream | GRTP1-AS1//ncRNA_intronic | 5 | chr13 | 114007908 | - | AY123041 | 276 | + | 13q34 |
| 12T | 20C211250_R303_CapNGS | HBVCgp4_PREC/C | PTPRB//intronic | 5 | chr12 | 71024629 | + | AY123041 | 1795 | - | 12q15 |
| 12T | 20C211250_R303_CapNGS | HBVCgp4_PREC/C | LINC01493(dist=1031752),LRRC4C(dist=427200)//intergenic | 5 | chr11 | 39708551 | + | AY123041 | 1899 | - | 11p12 |
| 12T | 20C211250_R303_CapNGS | HBVCgp3_X//upstream | ZBTB10(dist=35815),ZNF704(dist=66371)//intergenic | 6 | chr8 | 81474315 | + | AY123041 | 346 | + | 8q21.13 |
| 12T | 20C211250_R303_CapNGS | HBVCgp3_X | C8orf44-SGK3;SGK3//intronic | 6 | chr8 | 67715412 | + | AY123041 | 1549 | + | 8q13.1 |
| 12T | 20C211250_R303_CapNGS | HBVCgp4_PREC/C | CALN1//intronic | 6 | chr7 | 71702808 | + | AY123041 | 2183 | + | 7q11.22 |
| 12T | 20C211250_R303_CapNGS | HBVCgp4_PREC/C | KHDRBS2(dist=531175),LGSN(dist=458549)//intergenic | 6 | chr6 | 63527307 | + | AY123041 | 1795 | + | 6q12 |
| 12T | 20C211250_R303_CapNGS | HBVCgp4_PREC/C//downstream | CASC15//ncRNA_intronic | 6 | chr6 | 21954333 | + | AY123041 | 3086 | - | 6p22.3 |
| 12T | 20C211250_R303_CapNGS | HBVCgp4_PREC/C | CEP162//intronic | 6 | chr6 | 84915708 | + | AY123041 | 1795 | - | 6q14.3 |
| 12T | 20C211250_R303_CapNGS | HBVCgp3_X | GPBP1(dist=199623),ACTBL2(dist=15716)//intergenic | 6 | chr5 | 56760129 | - | AY123041 | 1418 | + | 5q11.2 |
| 12T | 20C211250_R303_CapNGS | HBVCgp4_PREC/C | ZNF662//UTR3 | 6 | chr3 | 42958054 | + | AY123041 | 1791 | - | 3p22.1 |
| 12T | 20C211250_R303_CapNGS | HBVCgp4_PREC/C | N4BP1(dist=168114),CBLN1(dist=499624)//intergenic | 6 | chr16 | 48812205 | + | AY123041 | 2229 | + | 16q12.1 |
| 12T | 20C211250_R303_CapNGS | HBVCgp4_PREC/C | CD180(dist=369756),LINC02242(dist=65711)//intergenic | 7 | chr5 | 66862383 | + | AY123041 | 1795 | + | 5q13.1 |
| 12T | 20C211250_R303_CapNGS | HBVCgp4_PREC/C | BEX5(dist=31944),TCP11X2(dist=27387)//intergenic | 8 | chrX | 101442893 | + | AY123041 | 1795 | + | Xq22.1 |
| 12T | 20C211250_R303_CapNGS | HBVCgp3_X//upstream | CWH43(dist=40627),NONE(dist=NONE)//intergenic | 8 | chr4 | 49104722 | + | AY123041 | 403 | + | 4p11 |
| 12T | 20C211250_R303_CapNGS | HBVCgp4_PREC/C | LINC00609;PTCSC3//ncRNA_intronic | 8 | chr14 | 36627204 | + | AY123041 | 1795 | + | 14q13.3 |
| 12T | 20C211250_R303_CapNGS | HBVCgp4_PREC/C | ARL5B(dist=362033),MALRD1(dist=5129)//intergenic | 8 | chr10 | 19332601 | + | AY123041 | 2175 | + | 10p12.31 |
| 12T | 20C211250_R303_CapNGS | NONE(dist=NONE),HBVCgp3_X(dist=1049)//intergenic | LINC01091//ncRNA_intronic | 9 | chr4 | 124701973 | + | AY123041 | 199 | + | 4q28.1 |
| 12T | 20C211250_R303_CapNGS | HBVCgp4_PREC/C | OR52H1(dist=29607),OR52B6(dist=5747)//intergenic | 9 | chr11 | 5596360 | + | AY123041 | 2133 | - | 11p15.4 |

| | | | | | | | | | | | |
|---|---|---|---|---|---|---|---|---|---|---|---|
| 12T | 20C211250_R303_CapNGS | HBVCgp2_PRES1/PRES2/S;HBVCgp1_P;HBVCgp3_X;HBVCgp4_PREC/C//upstream;downstream | TENM2//intronic | 10 | chr5 | 167041272 | - | AY123041 | 2386 | + | 5q34 |
| 12T | 20C211250_R303_CapNGS | HBVCgp4_PREC/C | DNAJC6//intronic | 11 | chr1 | 65815094 | + | AY123041 | 2218 | + | 1p31.3 |
| 12T | 20C211250_R303_CapNGS | HBVCgp3_X//upstream | ZNF385D//intronic | 13 | chr3 | 21692671 | + | AY123041 | 295 | + | 3p24.3 |
| 12T | 20C211250_R303_CapNGS | HBVCgp4_PREC/C | CHIC1//intronic | 14 | chrX | 72819910 | + | AY123041 | 1795 | + | Xq13.2 |
| 12T | 20C211250_R303_CapNGS | NONE(dist=NONE),HBVCgp3_X(dist=1167)//intergenic | MT-RNR2(dist=3189),MT-TS1(dist=1026)//intergenic | 14 | chrM | 6419 | + | AY123041 | 81 | - | |
| 12T | 20C211250_R303_CapNGS | HBVCgp4_PREC/C | DYNAP(dist=61574),RAB27B(dist=167410)//intergenic | 15 | chr18 | 52328298 | - | AY123041 | 1937 | + | 18q21.2 |
| 12T | 20C211250_R303_CapNGS | HBVCgp4_PREC/C | LOC401478//ncRNA_intronic | 20 | chr8 | 138922600 | + | AY123041 | 1959 | - | 8q24.23 |
| 12T | 20C211250_R303_CapNGS | HBVCgp2_PRES1/PRES2/S;HBVCgp1_P;HBVCgp3_X;HBVCgp4_PREC/C//upstream;downstream | KCNV1(dist=767985),LINC01608(dist=193934)//intergenic | 21 | chr8 | 111755985 | + | AY123041 | 2336 | - | 8q23.2 |
| 12T | 20C211250_R303_CapNGS | HBVCgp3_X//upstream | EEA1//exonic//EEA1:NM_003566:exon21:c.2856 | 25 | chr12 | 93192779 | + | AY123041 | 357 | + | 12q22 |
| 12T | 20C211250_R303_CapNGS | NONE(dist=NONE),HBVCgp3_X(dist=1025)//intergenic | CASZ1//intronic | 25 | chr1 | 10714977 | + | AY123041 | 223 | + | 1p36.22 |
| 12T | 20C211250_R303_CapNGS | HBVCgp4_PREC/C//downstream | LINC01692(dist=304929),LINC00158(dist=23148)//intergenic | 26 | chr21 | 26734985 | + | AY123041 | 3010 | + | 21q21.2 |
| 12T | 20C211250_R303_CapNGS | HBVCgp4_PREC/C | KMT2B//exonic//KMT2B:NM_014727:exon3:c.2421 | 31 | chr19 | 36212679 | + | AY123041 | 1808 | + | 19q13.12 |
| 12T | 20C211250_R303_CapNGS | HBVCgp4_PREC/C | LINC01088//ncRNA_intronic | 32 | chr4 | 79899323 | + | AY123041 | 1800 | + | 4q21.21 |
| 12T | 20C211250_R303_CapNGS | HBVCgp3_X//upstream | ZNF627(dist=19679),HNRNPA1P10(dist=26896)//intergenic | 33 | chr19 | 11749655 | + | AY123041 | 300 | + | 19p13.2 |
| 12T | 20C211250_R303_CapNGS | HBVCgp4_PREC/C | PDZRN4(dist=160292),LINC02400(dist=29152)//intergenic | 38 | chr12 | 42128839 | + | AY123041 | 1795 | + | 12q12 |
| 12T | 20C211250_R303_CapNGS | HBVCgp4_PREC/C | KIFAP3//intronic | 41 | chr1 | 170029544 | + | AY123041 | 1990 | + | 1q24.2 |
| 12T | 20C211250_R303_CapNGS | HBVCgp3_X//upstream | IL7//intronic | 44 | chr8 | 79684266 | + | AY123041 | 470 | - | 8q21.12 |
| 12T | 20C211250_R303_CapNGS | HBVCgp4_PREC/C | PREX2//intronic | 71 | chr8 | 68906821 | + | AY123041 | 1732 | - | 8q13.2 |
| 12T | 20C211250_R303_CapNGS | HBVCgp3_X//upstream | EZH2(dist=23353),RNY5(dist=33844)//intergenic | 96 | chr7 | 148604736 | + | AY123041 | 561 | + | 7q36.1 |
| 12T | 20C211250_R303_CapNGS | HBVCgp3_X;HBVCgp4_PREC/C | SAMD8(dist=7275),VDAC2(dist=20756)//intergenic | 59826 | chr10 | 76949156 | + | AY123041 | 1711 | + | 10q22.2 |
| 12T | 20C211250_R303_CapNGS | HBVCgp3_X;HBVCgp4_PREC/C | SAMD8(dist=7266),VDAC2(dist=20765)//intergenic | 59826 | chr10 | 76949147 | + | AY123041 | 1711 | + | 10q22.2 |
| 12T | 20C211250_R303_CapNGS | HBVCgp4_PREC/C | SAMD8(dist=7270),VDAC2(dist=20761)//intergenic | 59828 | chr10 | 76949151 | + | AY123041 | 1723 | + | 10q22.2 |
| 12T | 20C211250_R303_CapNGS | HBVCgp3_X;HBVCgp4_PREC/C | SAMD8(dist=7273),VDAC2(dist=20758)//intergenic | 59874 | chr10 | 76949154 | + | AY123041 | 1711 | + | 10q22.2 |
| 13T | 20C211251_R303_CapNGS | HBVCgp3_X//upstream | LINC01804(dist=99068),MYCNUT(dist=102368)//intergenic | 5 | chr2 | 15958153 | + | AY123041 | 396 | + | 2p24.3 |
| 13T | 20C211251_R303_CapNGS | HBVCgp4_PREC/C | N4BP1(dist=168114),CBLN1(dist=499624)//intergenic | 6 | chr16 | 48812209 | + | AY123041 | 2229 | + | 16q12.1 |
| 13T | 20C211251_R303_CapNGS | HBVCgp4_PREC/C | TGIF2LY(dist=771431),MIR9985(dist=254648)//intergenic | 7 | chrY | 4219513 | + | AY123041 | 1796 | - | Yp11.2 |

| | | | | | | | | | | | |
|---|---|---|---|---|---|---|---|---|---|---|---|
| 13T | 20C211251_R303_CapNGS | HBVCgp3_X//upstream | ANGPT1(dist=105489),RSPO2(dist=295909)//intergenic | 8 | chr8 | 108615635 | + | AY123041 | 403 | + | 8q23.1 |
| 13T | 20C211251_R303_CapNGS | HBVCgp4_PREC/C | OGFOD3//intronic | 10 | chr17 | 80364119 | + | AY123041 | 2310 | - | 17q25.3 |
| 13T | 20C211251_R303_CapNGS | HBVCgp3_X | MYH10//intronic | 10 | chr17 | 8399007 | - | AY123041 | 1364 | + | 17p13.1 |
| 13T | 20C211251_R303_CapNGS | HBVCgp3_X//upstream | RSPO2//downstream | 11 | chr8 | 108910569 | + | AY123041 | 482 | - | 8q23.1 |
| 13T | 20C211251_R303_CapNGS | NONE(dist=NONE),HBVCgp3_X(dist=1000)//intergenic | ZBTB49(dist=59039),NSG1(dist=5434)//intergenic | 11 | chr4 | 4382549 | + | AY123041 | 248 | + | 4p16.3 |
| 13T | 20C211251_R303_CapNGS | HBVCgp4_PREC/C | LOC105370829(dist=5183),RSL24D1(dist=83450)//intergenic | 12 | chr15 | 55389554 | + | AY123041 | 1796 | + | 15q21.3 |
| 13T | 20C211251_R303_CapNGS | HBVCgp4_PREC/C | STPG2//intronic | 13 | chr4 | 98703499 | + | AY123041 | 1796 | - | 4q22.3 |
| 13T | 20C211251_R303_CapNGS | HBVCgp4_PREC/C | LOC642484//ncRNA_intronic | 13 | chr18 | 53822588 | + | AY123041 | 1795 | - | 18q21.31 |
| 13T | 20C211251_R303_CapNGS | HBVCgp4_PREC/C//downstream | ANO2//intronic | 13 | chr12 | 5874706 | + | AY123041 | 2749 | + | 12p13.31 |
| 13T | 20C211251_R303_CapNGS | HBVCgp4_PREC/C | KIFAP3//intronic | 13 | chr1 | 170029543 | + | AY123041 | 1991 | + | 1q24.2 |
| 13T | 20C211251_R303_CapNGS | HBVCgp4_PREC/C | LINC01645(dist=330468),LINC01741(dist=3597)//intergenic | 13 | chr1 | 177666062 | + | AY123041 | 1795 | - | 1q25.2 |
| 13T | 20C211251_R303_CapNGS | HBVCgp4_PREC/C | LINC00972(dist=765710),GRM3(dist=388861)//intergenic | 14 | chr7 | 85884364 | + | AY123041 | 1796 | - | 7q21.11 |
| 13T | 20C211251_R303_CapNGS | HBVCgp4_PREC/C | AGPAT4//intronic | 14 | chr6 | 161595418 | + | AY123041 | 1796 | + | 6q26 |
| 13T | 20C211251_R303_CapNGS | HBVCgp4_PREC/C | ZNF385D-AS2(dist=875524),UBE2E2-AS1(dist=339717)//intergenic | 15 | chr3 | 22896844 | + | AY123041 | 1796 | + | 3p24.3 |
| 13T | 20C211251_R303_CapNGS | HBVCgp4_PREC/C | LINGO2//intronic | 16 | chr9 | 29009449 | + | AY123041 | 1796 | - | 9p21.1 |
| 13T | 20C211251_R303_CapNGS | HBVCgp4_PREC/C | TMEM163//intronic | 16 | chr2 | 135354423 | + | AY123041 | 1795 | + | 2q21.3 |
| 13T | 20C211251_R303_CapNGS | HBVCgp4_PREC/C | HACD4//intronic | 17 | chr9 | 21021649 | + | AY123041 | 1796 | - | 9p21.3 |
| 13T | 20C211251_R303_CapNGS | HBVCgp4_PREC/C | ELAVL2(dist=431165),IZUMO3(dist=261186)//intergenic | 17 | chr9 | 24281764 | + | AY123041 | 1795 | - | 9p21.3 |
| 13T | 20C211251_R303_CapNGS | HBVCgp4_PREC/C | LINC02737(dist=345992),NONE(dist=NONE)//intergenic | 17 | chr11 | 96593906 | + | AY123041 | 1795 | + | 11q21 |
| 13T | 20C211251_R303_CapNGS | HBVCgp4_PREC/C | MAGEC1(dist=214756),MAGEC2(dist=78214)//intergenic | 18 | chrX | 141211914 | + | AY123041 | 1795 | - | Xq27.2 |
| 13T | 20C211251_R303_CapNGS | HBVCgp4_PREC/C | LINC02339(dist=377848),LINC00358(dist=197731)//intergenic | 21 | chr13 | 62379927 | + | AY123041 | 1795 | - | 13q21.31 |
| 13T | 20C211251_R303_CapNGS | HBVCgp4_PREC/C | LINC02501(dist=125587),LINC02506(dist=312971)//intergenic | 22 | chr4 | 31686030 | + | AY123041 | 1795 | + | 4p15.1 |
| 13T | 20C211251_R303_CapNGS | HBVCgp4_PREC/C | KIAA1217//intronic | 22 | chr10 | 24676754 | + | AY123041 | 1795 | - | 10p12.1 |
| 13T | 20C211251_R303_CapNGS | HBVCgp4_PREC/C | DPP10//intronic | 24 | chr2 | 116088742 | + | AY123041 | 1795 | + | 2q14.1 |
| 13T | 20C211251_R303_CapNGS | HBVCgp4_PREC/C | PELI2(dist=157299),LOC101927690(dist=55508)//intergenic | 25 | chr14 | 56925541 | + | AY123041 | 1796 | - | 14q22.3 |
| 13T | 20C211251_R303_CapNGS | HBVCgp4_PREC/C | MIR4790(dist=455705),GRM7-AS3(dist=926400)//intergenic | 26 | chr3 | 5747645 | + | AY123041 | 1795 | + | 3p26.1 |
| 13T | 20C211251_R303_CapNGS | HBVCgp4_PREC/C | DPYD//intronic | 26 | chr1 | 97865244 | + | AY123041 | 1795 | + | 1p21.3 |
| 13T | 20C211251_R303_CapNGS | HBVCgp4_PREC/C | NFE2L3//intronic | 27 | chr7 | 26205009 | + | AY123041 | 1796 | + | 7p15.2 |
| 13T | 20C211251_R303_CapNGS | HBVCgp4_PREC/C | SPATA8(dist=481703),LINC02254(dist=103053)//intergenic | 27 | chr15 | 97810548 | + | AY123041 | 1795 | + | 15q26.2 |
| 13T | 20C211251_R303_CapNGS | HBVCgp4_PREC/C | CTNND2//intronic | 28 | chr5 | 11740072 | + | AY123041 | 1795 | - | 5p15.2 |
| 13T | 20C211251_R303_CapNGS | HBVCgp4_PREC/C | LOC100126447(dist=686509),KLHL13(dist=393439)//intergenic | 29 | chrX | 116638337 | + | AY123041 | 1795 | - | Xq24 |
| 13T | 20C211251_R303_CapNGS | HBVCgp4_PREC/C | URI1(dist=169785),ZNF536(dist=185987)//intergenic | 30 | chr19 | 30677304 | + | AY123041 | 1795 | + | 19q12 |

| | | | | | | | | | | | |
|---|---|---|---|---|---|---|---|---|---|---|---|
| 13T | 20C211251_R303_CapNGS | HBVCgp4_PREC/C | SPECC1//intronic | 30 | chr17 | 20200074 | + | AY123041 | 1795 | + | 17p11.2 |
| 13T | 20C211251_R303_CapNGS | HBVCgp4_PREC/C | SCFD2//intronic | 34 | chr4 | 53900912 | + | AY123041 | 1795 | - | 4q12 |
| 13T | 20C211251_R303_CapNGS | HBVCgp4_PREC/C | SYT10(dist=299039),ALG10(dist=283638)//intergenic | 34 | chr12 | 33891793 | + | AY123041 | 1795 | - | 12p11.1 |
| 13T | 20C211251_R303_CapNGS | HBVCgp4_PREC/C | MIR5011(dist=8544),DSEL(dist=416357)//intergenic | 35 | chr18 | 64757467 | + | AY123041 | 1795 | - | 18q22.1 |
| 13T | 20C211251_R303_CapNGS | HBVCgp4_PREC/C | C3orf67(dist=568196),LOC339902(dist=19068)//intergenic | 36 | chr3 | 59603954 | + | AY123041 | 1795 | - | 3p14.2 |
| 13T | 20C211251_R303_CapNGS | HBVCgp4_PREC/C | BRINP3//intronic | 39 | chr1 | 190223643 | + | AY123041 | 1795 | + | 1q31.1 |
| 13T | 20C211251_R303_CapNGS | HBVCgp4_PREC/C | XDH(dist=79263),SRD5A2(dist=30679)//intergenic | 45 | chr2 | 31716871 | + | AY123041 | 1795 | - | 2p23.1 |
| 13T | 20C211251_R303_CapNGS | HBVCgp4_PREC/C | DERA//intronic | 45 | chr12 | 16098404 | + | AY123041 | 1795 | + | 12p12.3 |
| 13T | 20C211251_R303_CapNGS | HBVCgp4_PREC/C | PDE4D//intronic | 47 | chr5 | 58985813 | + | AY123041 | 1800 | - | 5q12.1 |
| 13T | 20C211251_R303_CapNGS | HBVCgp4_PREC/C | SKAP2(dist=139599),HOXA1(dist=88806)//intergenic | 52 | chr7 | 27043808 | + | AY123041 | 1795 | + | 7p15.2 |
| 13T | 20C211251_R303_CapNGS | HBVCgp4_PREC/C | LINC00326(dist=12815),EYA4(dist=121963)//intergenic | 55 | chr6 | 133440532 | + | AY123041 | 1795 | + | 6q23.2 |
| 13T | 20C211251_R303_CapNGS | HBVCgp3_X;HBVCgp4_PREC/C | MIR4268(dist=294708),EPHA4(dist=1216753)//intergenic | 61 | chr2 | 221065992 | - | AY123041 | 1695 | + | 2q35 |
| 13T | 20C211251_R303_CapNGS | HBVCgp4_PREC/C | HS3ST4(dist=272577),C16orf82(dist=656665)//intergenic | 64 | chr16 | 26421583 | + | AY123041 | 1795 | + | 16p12.1 |
| 13T | 20C211251_R303_CapNGS | HBVCgp4_PREC/C//downstream | TTC27//intronic | 73 | chr2 | 32917423 | - | AY123041 | 2814 | + | 2p22.3 |
| 13T | 20C211251_R303_CapNGS | HBVCgp4_PREC/C | LINC02172(dist=311320),LINC00616(dist=112673)//intergenic | 80 | chr4 | 138835904 | + | AY123041 | 1795 | + | 4q28.3 |
| 13T | 20C211251_R303_CapNGS | HBVCgp4_PREC/C | LINC02347(dist=167450),LOC100996671(dist=12711)//intergenic | 80 | chr12 | 127124781 | + | AY123041 | 1795 | + | 12q24.32 |
| 13T | 20C211251_R303_CapNGS | HBVCgp4_PREC/C | CEP162//intronic | 81 | chr6 | 84915710 | + | AY123041 | 1795 | - | 6q14.3 |
| 13T | 20C211251_R303_CapNGS | HBVCgp4_PREC/C | PTPRB//intronic | 82 | chr12 | 71024623 | + | AY123041 | 1795 | - | 12q15 |
| 13T | 20C211251_R303_CapNGS | HBVCgp4_PREC/C | PTPRB//intronic | 84 | chr12 | 71024634 | + | AY123041 | 1795 | - | 12q15 |
| 13T | 20C211251_R303_CapNGS | HBVCgp4_PREC/C | CEP162//intronic | 85 | chr6 | 84915712 | + | AY123041 | 1799 | - | 6q14.3 |
| 13T | 20C211251_R303_CapNGS | HBVCgp4_PREC/C | PTPRB//intronic | 85 | chr12 | 71024633 | + | AY123041 | 1795 | - | 12q15 |
| 13T | 20C211251_R303_CapNGS | HBVCgp4_PREC/C | ADAM2//intronic | 92 | chr8 | 39669719 | + | AY123041 | 1796 | - | 8p11.22 |
| 13T | 20C211251_R303_CapNGS | HBVCgp4_PREC/C | ZNF835(dist=31175),ZIM2-AS1(dist=62390)//intergenic | 108 | chr19 | 57214300 | + | AY123041 | 1795 | + | 19q13.43 |
| 13T | 20C211251_R303_CapNGS | HBVCgp4_PREC/C | GABRB3//intronic | 118 | chr15 | 26863082 | + | AY123041 | 1795 | - | 15q12 |
| 13T | 20C211251_R303_CapNGS | HBVCgp4_PREC/C | TDGF1P3(dist=27043),CHRDL1(dist=123792)//intergenic | 125 | chrX | 109793292 | + | AY123041 | 1795 | - | Xq23 |
| 13T | 20C211251_R303_CapNGS | HBVCgp4_PREC/C | MIR3974(dist=354789),RERGL(dist=52686)//intergenic | 137 | chr12 | 18181117 | + | AY123041 | 1795 | - | 12p12.3 |
| 13T | 20C211251_R303_CapNGS | HBVCgp4_PREC/C | LINC00609;PTCSC3//ncRNA_intronic | 140 | chr14 | 36627201 | + | AY123041 | 1795 | + | 14q13.3 |
| 13T | 20C211251_R303_CapNGS | HBVCgp4_PREC/C | CCNYL2//ncRNA_intronic | 140 | chr10 | 42935115 | + | AY123041 | 1795 | - | 10q11.21 |
| 13T | 20C211251_R303_CapNGS | HBVCgp4_PREC/C | IPO11(dist=128205),HTR1A(dist=1203254)//intergenic | 146 | chr5 | 62052621 | + | AY123041 | 1795 | - | 5q12.1 |
| 13T | 20C211251_R303_CapNGS | HBVCgp4_PREC/C | C3orf67-AS1//ncRNA_intronic | 161 | chr3 | 58920919 | + | AY123041 | 1795 | - | 3p14.2 |
| 13T | 20C211251_R303_CapNGS | HBVCgp4_PREC/C | RICTOR//intronic | 165 | chr5 | 38988885 | + | AY123041 | 1795 | - | 5p13.1 |
| 13T | 20C211251_R303_CapNGS | HBVCgp4_PREC/C | ZPLD1(dist=298033),MIR548AB(dist=746159)//intergenic | 177 | chr3 | 102496718 | + | AY123041 | 1795 | - | 3q12.3 |
| 13T | 20C211251_R303_CapNGS | HBVCgp4_PREC/C | CHIC1//intronic | 226 | chrX | 72819911 | + | AY123041 | 1795 | + | Xq13.2 |

| | | | | | | | | | | | |
|---|---|---|---|---|---|---|---|---|---|---|---|
| 13T | 20C211251_R303_CapNGS | HBVCgp4_PREC/C | KIFAP3//intronic | 1501 | chr1 | 170029542 | + | AY123041 | 1990 | + | 1q24.2 |
| 13T | 20C211251_R303_CapNGS | HBVCgp4_PREC/C | KIFAP3//intronic | 1504 | chr1 | 170029540 | + | AY123041 | 1990 | + | 1q24.2 |
| 13T | 20C211251_R303_CapNGS | HBVCgp4_PREC/C | KIFAP3//intronic | 1506 | chr1 | 170029541 | + | AY123041 | 1990 | + | 1q24.2 |
| 13T | 20C211251_R303_CapNGS | HBVCgp4_PREC/C | KIFAP3//intronic | 1509 | chr1 | 170029539 | + | AY123041 | 1990 | + | 1q24.2 |
| 13T | 20C211251_R303_CapNGS | HBVCgp4_PREC/C | LINC01088//ncRNA_intronic | 2841 | chr4 | 79899325 | + | AY123041 | 1797 | + | 4q21.21 |
| 13T | 20C211251_R303_CapNGS | HBVCgp3_X//upstream | EZH2(dist=23351),RNY5(dist=33846)//intergenic | 4800 | chr7 | 148604734 | + | AY123041 | 560 | + | 7q36.1 |
| 13T | 20C211251_R303_CapNGS | HBVCgp4_PREC/C | PREX2//intronic | 5170 | chr8 | 68906824 | + | AY123041 | 1732 | - | 8q13.2 |
| 13T | 20C211251_R303_CapNGS | HBVCgp4_PREC/C | PREX2//intronic | 5172 | chr8 | 68906826 | + | AY123041 | 1732 | - | 8q13.2 |
| 13T | 20C211251_R303_CapNGS | HBVCgp3_X//upstream | EZH2(dist=23353),RNY5(dist=33844)//intergenic | 5285 | chr7 | 148604732 | + | AY123041 | 556 | + | 7q36.1 |
| 13T | 20C211251_R303_CapNGS | HBVCgp3_X//upstream | EZH2(dist=23362),RNY5(dist=33835)//intergenic | 5495 | chr7 | 148604745 | + | AY123041 | 561 | + | 7q36.1 |
| 13T | 20C211251_R303_CapNGS | HBVCgp3_X//upstream | EZH2(dist=23357),RNY5(dist=33840)//intergenic | 5496 | chr7 | 148604740 | + | AY123041 | 561 | + | 7q36.1 |
| 13T | 20C211251_R303_CapNGS | HBVCgp3_X//upstream | EZH2(dist=23356),RNY5(dist=33841)//intergenic | 5496 | chr7 | 148604739 | + | AY123041 | 561 | + | 7q36.1 |
| 13T | 20C211251_R303_CapNGS | HBVCgp3_X//upstream | EZH2(dist=23355),RNY5(dist=33842)//intergenic | 5496 | chr7 | 148604738 | + | AY123041 | 561 | + | 7q36.1 |
| 13T | 20C211251_R303_CapNGS | HBVCgp4_PREC/C | PREX2//intronic | 5632 | chr8 | 68906829 | + | AY123041 | 1732 | - | 8q13.2 |
| 13T | 20C211251_R303_CapNGS | HBVCgp4_PREC/C | PREX2//intronic | 6057 | chr8 | 68906822 | - | AY123041 | 1732 | + | 8q13.2 |
| 13T | 20C211251_R303_CapNGS | HBVCgp4_PREC/C | PREX2//intronic | 6058 | chr8 | 68906823 | - | AY123041 | 1732 | + | 8q13.2 |
| 13T | 20C211251_R303_CapNGS | HBVCgp3_X | NDRG4//intronic | 9254 | chr16 | 58513258 | + | AY123041 | 1631 | - | 16q21 |
| 14T | 20C211252_R303_CapNGS | HBVCgp3_X;HBVCgp4_PREC/C | NLRP2B(dist=32787),ZXDA(dist=192384)//intergenic | 5 | chrX | 57739480 | + | AY123041 | 1703 | + | Xp11.21 |
| 14T | 20C211252_R303_CapNGS | NONE(dist=NONE),HBVCgp3_X(dist=1172)//intergenic | KLF8//intronic | 5 | chrX | 55965372 | + | AY123041 | 76 | - | Xp11.21 |
| 14T | 20C211252_R303_CapNGS | HBVCgp3_X;HBVCgp4_PREC/C | RAP2C-AS1//ncRNA_intronic | 5 | chrX | 131420105 | + | AY123041 | 1703 | - | Xq26.2 |
| 14T | 20C211252_R303_CapNGS | HBVCgp3_X;HBVCgp4_PREC/C | NONE(dist=NONE),SPIN4(dist=775752)//intergenic | 5 | chrX | 61791355 | + | AY123041 | 1703 | - | Xq11.1 |
| 14T | 20C211252_R303_CapNGS | HBVCgp3_X;HBVCgp4_PREC/C | MIR325HG(dist=276153),FGF16(dist=198537)//intergenic | 5 | chrX | 76511110 | + | AY123041 | 1701 | - | Xq21.1 |
| 14T | 20C211252_R303_CapNGS | NONE(dist=NONE),HBVCgp3_X(dist=1011)//intergenic | H3-5(dist=61848),LINC02422(dist=22823)//intergenic | 5 | chr12 | 32007080 | + | AY123041 | 237 | + | 12p11.21 |
| 14T | 20C211252_R303_CapNGS | HBVCgp3_X;HBVCgp4_PREC/C | CNTN5//intronic | 5 | chr11 | 99450166 | + | AY123041 | 1703 | - | 11q22.1 |
| 14T | 20C211252_R303_CapNGS | HBVCgp3_X//upstream | LINC01509//ncRNA_intronic | 6 | chr9 | 110183480 | + | AY123041 | 657 | + | 9q31.2 |
| 14T | 20C211252_R303_CapNGS | HBVCgp2_PRES1/PRES2/S;HBVCgp1_P;HBVCgp3_X;HBVCgp4_PREC/C//upstream;downstream | PI15//intronic | 6 | chr8 | 75757759 | + | AY123041 | 2362 | + | 8q21.11 |
| 14T | 20C211252_R303_CapNGS | HBVCgp3_X//upstream | LINC02208//ncRNA_intronic | 6 | chr5 | 117868871 | + | AY123041 | 258 | - | 5q23.1 |
| 14T | 20C211252_R303_CapNGS | HBVCgp3_X | CDH12//intronic | 6 | chr5 | 22654053 | + | AY123041 | 1672 | - | 5p14.3 |
| 14T | 20C211252_R303_CapNGS | HBVCgp4_PREC/C | UQCC1(dist=5513),GDF5-AS1(dist=15369)//intergenic | 6 | chr20 | 34005458 | + | AY123041 | 2296 | + | 20q11.22 |
| 14T | 20C211252_R303_CapNGS | HBVCgp3_X//upstream | PMS1(dist=49614),MSTN(dist=128457)//intergenic | 6 | chr2 | 190791969 | + | AY123041 | 356 | - | 2q32.2 |

| | | | | | | | | | | | |
|---|---|---|---|---|---|---|---|---|---|---|---|
| 14T | 20C211252_R303_CapNGS | HBVCgp3_X//upstream | AXL//intronic | 6 | chr19 | 41733635 | + | AY123041 | 267 | + | 19q13.2 |
| 14T | 20C211252_R303_CapNGS | HBVCgp3_X;HBVCgp4_PREC/C//upstream | BIN2//intronic | 6 | chr12 | 51716731 | + | AY123041 | 696 | + | 12q13.13 |
| 14T | 20C211252_R303_CapNGS | HBVCgp3_X;HBVCgp4_PREC/C | TLL1(dist=248162),SPOCK3(dist=380765)//intergenic | 7 | chr4 | 167273771 | + | AY123041 | 1700 | + | 4q32.3 |
| 14T | 20C211252_R303_CapNGS | HBVCgp3_X;HBVCgp4_PREC/C | LOC100126447(dist=961282),KLHL13(dist=118666)//intergenic | 8 | chrX | 116913110 | + | AY123041 | 1703 | - | Xq24 |
| 14T | 20C211252_R303_CapNGS | HBVCgp4_PREC/C | LINC01904(dist=315678),LINC00470(dist=24640)//intergenic | 8 | chr18 | 1243672 | + | AY123041 | 2053 | - | 18p11.32 |
| 14T | 20C211252_R303_CapNGS | HBVCgp4_PREC/C | CLCN5(dist=5875),AKAP4(dist=85653)//intergenic | 9 | chrX | 49869767 | + | AY123041 | 1846 | + | Xp11.22 |
| 14T | 20C211252_R303_CapNGS | HBVCgp3_X;HBVCgp4_PREC/C | GBA3(dist=68187),PPARGC1A(dist=904262)//intergenic | 9 | chr4 | 22889382 | + | AY123041 | 1703 | - | 4p15.2 |
| 14T | 20C211252_R303_CapNGS | HBVCgp4_PREC/C | IFT140//intronic | 9 | chr16 | 1619170 | + | AY123041 | 1827 | + | 16p13.3 |
| 14T | 20C211252_R303_CapNGS | HBVCgp3_X//upstream | CFTR-AS1//ncRNA_intronic | 10 | chr7 | 117188313 | + | AY123041 | 419 | - | 7q31.2 |
| 14T | 20C211252_R303_CapNGS | HBVCgp3_X//upstream | GOT1(dist=26971),LINC01475(dist=68755)//intergenic | 10 | chr10 | 101217352 | - | AY123041 | 462 | + | 10q24.2 |
| 14T | 20C211252_R303_CapNGS | HBVCgp3_X;HBVCgp4_PREC/C | SNX19(dist=26412),NTM(dist=427716)//intergenic | 11 | chr11 | 130812794 | + | AY123041 | 1703 | - | 11q25 |
| 14T | 20C211252_R303_CapNGS | HBVCgp3_X;HBVCgp4_PREC/C//upstream | GRM5-AS1//ncRNA_exonic// | 12 | chr11 | 88241568 | + | AY123041 | 736 | + | 11q14.2 |
| 14T | 20C211252_R303_CapNGS | HBVCgp3_X;HBVCgp4_PREC/C | FHIT//intronic | 14 | chr3 | 60428611 | + | AY123041 | 1706 | - | 3p14.2 |
| 14T | 20C211252_R303_CapNGS | HBVCgp4_PREC/C | POU6F2//intronic | 15 | chr7 | 39333945 | + | AY123041 | 1786 | + | 7p14.1 |
| 14T | 20C211252_R303_CapNGS | HBVCgp3_X//upstream | GJA1(dist=801703),HSF2(dist=148120)//intergenic | 16 | chr6 | 122572576 | + | AY123041 | 491 | + | 6q22.31 |
| 14T | 20C211252_R303_CapNGS | HBVCgp3_X;HBVCgp4_PREC/C | BMPR2(dist=39346),FAM117B(dist=27874)//intergenic | 16 | chr2 | 203471818 | + | AY123041 | 1703 | - | 2q33.2 |
| 14T | 20C211252_R303_CapNGS | HBVCgp3_X;HBVCgp4_PREC/C | LOC284395//ncRNA_intronic | 16 | chr19 | 29843774 | + | AY123041 | 1701 | + | 19q12 |
| 14T | 20C211252_R303_CapNGS | HBVCgp3_X//upstream | TUNAR(dist=46649),C14orf132(dist=67104)//intergenic | 16 | chr14 | 96438557 | + | AY123041 | 364 | - | 14q32.2 |
| 14T | 20C211252_R303_CapNGS | HBVCgp3_X//upstream | GRM5//intronic | 16 | chr11 | 88340176 | + | AY123041 | 586 | + | 11q14.3 |
| 14T | 20C211252_R303_CapNGS | HBVCgp4_PREC/C | RAB13(dist=3075),RPS27(dist=1341)//intergenic | 16 | chr1 | 153961890 | + | AY123041 | 1826 | + | 1q21.3 |
| 14T | 20C211252_R303_CapNGS | HBVCgp3_X;HBVCgp4_PREC/C | ANKRD30BP2(dist=29936),MIR3156-3(dist=258198)//intergenic | 17 | chr21 | 14520507 | + | AY123041 | 1704 | + | 21q11.2 |
| 14T | 20C211252_R303_CapNGS | HBVCgp3_X;HBVCgp4_PREC/C | DPP10(dist=1921845),DDX18(dist=47068)//intergenic | 17 | chr2 | 118525199 | + | AY123041 | 1703 | - | 2q14.1 |
| 14T | 20C211252_R303_CapNGS | HBVCgp4_PREC/C | ADGRA3(dist=132248),GBA3(dist=44600)//intergenic | 18 | chr4 | 22649937 | + | AY123041 | 2206 | - | 4p15.2 |
| 14T | 20C211252_R303_CapNGS | HBVCgp3_X;HBVCgp4_PREC/C | CYSLTR1(dist=162296),RTL3(dist=166081)//intergenic | 19 | chrX | 77745484 | + | AY123041 | 1703 | - | Xq21.1 |
| 14T | 20C211252_R303_CapNGS | HBVCgp4_PREC/C | FGFR4(dist=10174),NSD1(dist=24761)//intergenic | 21 | chr5 | 176535319 | + | AY123041 | 1825 | - | 5q35.2 |
| 14T | 20C211252_R303_CapNGS | HBVCgp4_PREC/C | SBNO2(dist=20787),STK11(dist=10723)//intergenic | 23 | chr19 | 1195054 | + | AY123041 | 1825 | + | 19p13.3 |
| 14T | 20C211252_R303_CapNGS | HBVCgp3_X//upstream | DLX4//UTR3 | 24 | chr17 | 48051908 | + | AY123041 | 376 | - | 17q21.33 |
| 14T | 20C211252_R303_CapNGS | HBVCgp4_PREC/C | GRID1//intronic | 25 | chr10 | 87496755 | + | AY123041 | 2161 | - | 10q23.1 |
| 14T | 20C211252_R303_CapNGS | HBVCgp4_PREC/C | TBC1D32//intronic | 26 | chr6 | 121596684 | + | AY123041 | 1856 | - | 6q22.31 |

| | | | | | | | | | | | |
|---|---|---|---|---|---|---|---|---|---|---|---|
| 14T | 20C211252_R303_CapNGS | HBVCgp3_X;HBVCgp4_PREC/C | APLF(dist=35094),PROKR1(dist=28332)//intergenic | 26 | chr2 | 68842388 | + | AY123041 | 1703 | - | 2p13.3 |
| 14T | 20C211252_R303_CapNGS | HBVCgp3_X;HBVCgp4_PREC/C | SRD5A1P1(dist=79119),F9(dist=2644)//intergenic | 27 | chrX | 138610251 | + | AY123041 | 1703 | + | Xq27.1 |
| 14T | 20C211252_R303_CapNGS | HBVCgp3_X;HBVCgp4_PREC/C | LINC02465//ncRNA_intronic | 30 | chr4 | 130800672 | + | AY123041 | 1703 | - | 4q28.2 |
| 14T | 20C211252_R303_CapNGS | HBVCgp3_X;HBVCgp4_PREC/C | MIR3974(dist=64458),RERGL(dist=343017)//intergenic | 34 | chr12 | 17890786 | + | AY123041 | 1703 | + | 12p12.3 |
| 14T | 20C211252_R303_CapNGS | HBVCgp3_X;HBVCgp4_PREC/C | DUSP26(dist=459216),LINC01288(dist=724599)//intergenic | 44 | chr8 | 33916840 | + | AY123041 | 1703 | + | 8p12 |
| 14T | 20C211252_R303_CapNGS | HBVCgp4_PREC/C | FAM83B//intronic | 49 | chr6 | 54765808 | + | AY123041 | 2210 | - | 6p12.1 |
| 14T | 20C211252_R303_CapNGS | HBVCgp3_X//upstream | MIR4318(dist=1538556),MIR924HG(dist=11154)//intergenic | 53 | chr18 | 36775734 | + | AY123041 | 429 | + | 18q12.2 |
| 14T | 20C211252_R303_CapNGS | NONE(dist=NONE),HBVCgp3_X(dist=1178)//intergenic | CRAT37(dist=72576),SLCO3A1(dist=286282)//intergenic | 69 | chr15 | 92110656 | + | AY123041 | 70 | - | 15q26.1 |
| 14T | 20C211252_R303_CapNGS | HBVCgp3_X;HBVCgp4_PREC/C | LINC01790(dist=465760),SLC39A10(dist=429763)//intergenic | 72 | chr2 | 196091919 | + | AY123041 | 1706 | + | 2q32.3 |
| 14T | 20C211252_R303_CapNGS | HBVCgp3_X;HBVCgp4_PREC/C | LINC02041(dist=133304),SST(dist=85659)//intergenic | 95 | chr3 | 187301041 | + | AY123041 | 1703 | - | 3q27.3 |
| 14T | 20C211252_R303_CapNGS | HBVCgp4_PREC/C | AHDC1//intronic | 96 | chr1 | 27919000 | + | AY123041 | 1812 | + | 1p36.11 |
| 14T | 20C211252_R303_CapNGS | HBVCgp4_PREC/C | KCNIP4(dist=290449),LOC100505912(dist=88163)//intergenic | 174 | chr4 | 22240823 | + | AY123041 | 1728 | + | 4p15.2 |
| 14T | 20C211252_R303_CapNGS | HBVCgp4_PREC/C | ADPGK-AS1(dist=140022),NEO1(dist=114242)//intergenic | 403 | chr15 | 73230562 | + | AY123041 | 1827 | + | 15q24.1 |
| 14T | 20C211252_R303_CapNGS | HBVCgp3_X;HBVCgp4_PREC/C | LINC00364(dist=1446569),LINC00550(dist=34721)//intergenic | 717 | chr13 | 69400695 | + | AY123041 | 1704 | + | 13q21.33 |
| 14T | 20C211252_R303_CapNGS | HBVCgp3_X;HBVCgp4_PREC/C | KLF8//intronic | 6527 | chrX | 56200136 | + | AY123041 | 1700 | - | Xp11.21 |
| 14T | 20C211252_R303_CapNGS | NONE(dist=NONE),HBVCgp3_X(dist=1055)//intergenic | N4BP1(dist=213833),CBLN1(dist=453905)//intergenic | 11428 | chr16 | 48857924 | + | AY123041 | 193 | + | 16q12.1 |
| 14T | 20C211252_R303_CapNGS | NONE(dist=NONE),HBVCgp3_X(dist=1055)//intergenic | N4BP1(dist=213831),CBLN1(dist=453907)//intergenic | 11428 | chr16 | 48857922 | + | AY123041 | 193 | + | 16q12.1 |
| 14T | 20C211252_R303_CapNGS | NONE(dist=NONE),HBVCgp3_X(dist=1055)//intergenic | N4BP1(dist=213832),CBLN1(dist=453906)//intergenic | 12189 | chr16 | 48857923 | + | AY123041 | 193 | + | 16q12.1 |
| 14T | 20C211252_R303_CapNGS | HBVCgp4_PREC/C | N4BP1(dist=168114),CBLN1(dist=499624)//intergenic | 14180 | chr16 | 48812206 | + | AY123041 | 2226 | + | 16q12.1 |
| 14T | 20C211252_R303_CapNGS | HBVCgp3_X//upstream | ANO2//intronic | 19678 | chr12 | 5874718 | + | AY123041 | 336 | + | 12p13.31 |
| 14T | 20C211252_R303_CapNGS | HBVCgp3_X//upstream | ANO2//intronic | 19678 | chr12 | 5874719 | + | AY123041 | 336 | + | 12p13.31 |
| 14T | 20C211252_R303_CapNGS | HBVCgp3_X//upstream | ANO2//intronic | 19678 | chr12 | 5874722 | + | AY123041 | 336 | + | 12p13.31 |
| 14T | 20C211252_R303_CapNGS | HBVCgp3_X//upstream | ANO2//intronic | 19678 | chr12 | 5874720 | + | AY123041 | 336 | + | 12p13.31 |
| 14T | 20C211252_R303_CapNGS | HBVCgp3_X//upstream | ANO2//intronic | 19678 | chr12 | 5874721 | + | AY123041 | 336 | + | 12p13.31 |
| 14T | 20C211252_R303_CapNGS | HBVCgp3_X//upstream | ANO2//intronic | 19678 | chr12 | 5874717 | + | AY123041 | 336 | + | 12p13.31 |
| 14T | 20C211252_R303_CapNGS | HBVCgp4_PREC/C | ANO2//intronic | 22302 | chr12 | 5874709 | + | AY123041 | 2181 | + | 12p13.31 |
| 14T | 20C211252_R303_CapNGS | HBVCgp4_PREC/C | KMT2B//exonic//KMT2B:NM_014727:exon3:c.2419 | 33589 | chr19 | 36212668 | + | AY123041 | 1801 | + | 19q13.12 |

| | | | | | | | | | | | |
|---|---|---|---|---|---|---|---|---|---|---|---|
| 14T | 20C211252_R303_CapNGS | HBVCgp4_PREC/C | KMT2B//exonic//KMT2B:NM_014727:exon3:c.2428 | 33591 | chr19 | 36212677 | + | AY123041 | 1808 | + | 19q13.12 |
| 14T | 20C211252_R303_CapNGS | HBVCgp4_PREC/C | KMT2B//exonic//KMT2B:NM_014727:exon3:c.2427 | 33591 | chr19 | 36212676 | + | AY123041 | 1808 | + | 19q13.12 |
| 14T | 20C211252_R303_CapNGS | HBVCgp4_PREC/C | KMT2B//exonic//KMT2B:NM_014727:exon3:c.2423 | 33591 | chr19 | 36212672 | + | AY123041 | 1801 | + | 19q13.12 |
| 14T | 20C211252_R303_CapNGS | HBVCgp4_PREC/C | KMT2B//exonic//KMT2B:NM_014727:exon3:c.2426 | 33595 | chr19 | 36212675 | + | AY123041 | 1793 | + | 19q13.12 |
| 14T | 20C211252_R303_CapNGS | HBVCgp4_PREC/C | KMT2B//exonic//KMT2B:NM_014727:exon3:c.2425 | 33619 | chr19 | 36212674 | + | AY123041 | 1804 | + | 19q13.12 |
| 14T | 20C211252_R303_CapNGS | HBVCgp4_PREC/C | KMT2B//exonic//KMT2B:NM_014727:exon3:c.2424 | 33634 | chr19 | 36212673 | + | AY123041 | 1793 | + | 19q13.12 |
| 14T | 20C211252_R303_CapNGS | HBVCgp4_PREC/C | KMT2B//exonic//KMT2B:NM_014727:exon3:c.2422 | 33741 | chr19 | 36212671 | + | AY123041 | 1807 | + | 19q13.12 |
| 14T | 20C211252_R303_CapNGS | HBVCgp4_PREC/C | KMT2B//exonic//KMT2B:NM_014727:exon3:c.2421 | 33959 | chr19 | 36212678 | + | AY123041 | 1793 | + | 19q13.12 |
| 14T | 20C211252_R303_CapNGS | HBVCgp3_X;HBVCgp4_PREC/C | MIR4268(dist=294709),EPHA4(dist=1216752)//intergenic | 40208 | chr2 | 221065995 | + | AY123041 | 1689 | - | 2q35 |
| 14T | 20C211252_R303_CapNGS | HBVCgp3_X;HBVCgp4_PREC/C | MIR4268(dist=294708),EPHA4(dist=1216753)//intergenic | 41581 | chr2 | 221065994 | + | AY123041 | 1689 | - | 2q35 |
| 15T | 20C211253_R303_CapNGS | HBVCgp4_PREC/C | HDX(dist=5157),UBE2DNL(dist=426539)//intergenic | 5 | chrX | 83762618 | + | AY123041 | 1850 | + | Xq21.1 |
| 15T | 20C211253_R303_CapNGS | HBVCgp2_PRES1/PRES2/S;HBVCgp3_X;HBVCgp4_PREC/C//upstream;downstream | LINC02462(dist=529601),LINC02485(dist=37980)//intergenic | 5 | chr4 | 135996260 | + | AY123041 | 2709 | + | 4q28.3 |
| 15T | 20C211253_R303_CapNGS | HBVCgp3_X | RIT2//intronic | 5 | chr18 | 40649017 | + | AY123041 | 1565 | + | 18q12.3 |
| 15T | 20C211253_R303_CapNGS | HBVCgp3_X;HBVCgp4_PREC/C//upstream | GS1-279B7.1(dist=135585),LINC01350(dist=87756)//intergenic | 5 | chr1 | 185439756 | + | AY123041 | 994 | - | 1q25.3 |
| 15T | 20C211253_R303_CapNGS | HBVCgp3_X | AUTS2//exonic//AUTS2:NM_001127231:exon18:c.3650,AUTS2:NM_015570:exon19:c.3722 | 6 | chr7 | 70255924 | + | AY123041 | 1504 | + | 7q11.22 |
| 15T | 20C211253_R303_CapNGS | HBVCgp4_PREC/C | COG6(dist=387115),LINC00332(dist=3028)//intergenic | 6 | chr13 | 40752918 | + | AY123041 | 2111 | - | 13q14.11 |
| 15T | 20C211253_R303_CapNGS | HBVCgp3_X//upstream | GAD2(dist=63143),APBB1IP(dist=70636)//intergenic | 6 | chr10 | 26656634 | + | AY123041 | 517 | - | 10p12.1 |
| 15T | 20C211253_R303_CapNGS | HBVCgp4_PREC/C | PSMB2//UTR3 | 6 | chr1 | 36066425 | + | AY123041 | 1983 | + | 1p34.3 |
| 15T | 20C211253_R303_CapNGS | HBVCgp3_X | FTX//ncRNA_intronic | 8 | chrX | 73333288 | + | AY123041 | 1260 | + | Xq13.2 |
| 15T | 20C211253_R303_CapNGS | HBVCgp3_X;HBVCgp4_PREC/C | EYA1(dist=283381),MSC(dist=10067)//intergenic | 8 | chr8 | 72743710 | + | AY123041 | 1689 | + | 8q13.3 |
| 15T | 20C211253_R303_CapNGS | HBVCgp4_PREC/C | LINC00299//ncRNA_intronic | 9 | chr2 | 8229772 | + | AY123041 | 2237 | + | 2p25.1 |
| 15T | 20C211253_R303_CapNGS | HBVCgp4_PREC/C | LINC02024(dist=9584),LOC105374060(dist=807902)//intergenic | 12 | chr3 | 117419356 | + | AY123041 | 2058 | - | 3q13.32 |
| 15T | 20C211253_R303_CapNGS | HBVCgp3_X//upstream | ZNF521(dist=460714),SS18(dist=203373)//intergenic | 12 | chr18 | 23392844 | + | AY123041 | 491 | - | 18q11.2 |
| 15T | 20C211253_R303_CapNGS | HBVCgp3_X | REN//exonic//REN:NM_000537:exon10:c.1187 | 12 | chr1 | 204124178 | - | AY123041 | 1560 | + | 1q32.1 |
| 15T | 20C211253_R303_CapNGS | HBVCgp4_PREC/C | DCAF1//intronic | 13 | chr3 | 51515806 | + | AY123041 | 2312 | + | 3p21.2 |
| 15T | 20C211253_R303_CapNGS | HBVCgp3_X//upstream | PLGRKT//intronic | 15 | chr9 | 5398706 | + | AY123041 | 484 | - | 9p24.1 |

| | | | | | | | | | | | |
|---|---|---|---|---|---|---|---|---|---|---|---|
| 15T | 20C211253_R303_CapNGS | HBVCgp4_PREC/C | CDH18//intronic | 17 | chr5 | 20112531 | - | AY123041 | 1839 | + | 5p14.3 |
| 15T | 20C211253_R303_CapNGS | HBVCgp4_PREC/C | ARHGEF10L//intronic | 21 | chr1 | 17881712 | + | AY123041 | 2285 | - | 1p36.13 |
| 15T | 20C211253_R303_CapNGS | HBVCgp4_PREC/C | ZNF385D-AS2(dist=614049),UBE2E2-AS1(dist=601192)//intergenic | 25 | chr3 | 22635369 | + | AY123041 | 1813 | - | 3p24.3 |
| 15T | 20C211253_R303_CapNGS | HBVCgp3_X | GRM5//intronic | 26 | chr11 | 88394583 | + | AY123041 | 1593 | - | 11q14.3 |
| 15T | 20C211253_R303_CapNGS | HBVCgp4_PREC/C | CAMK1D//intronic | 26 | chr10 | 12828440 | + | AY123041 | 2258 | - | 10p13 |
| 15T | 20C211253_R303_CapNGS | HBVCgp3_X | ARL17B//intronic | 27 | chr17 | 44359165 | + | AY123041 | 1519 | + | 17q21.31 |
| 15T | 20C211253_R303_CapNGS | HBVCgp2_PRES1/PRES2/S;HBVCgp1_P;HBVCgp3_X;HBVCgp4_PREC/C//upstream;downstream | MIR4289(dist=73783),C9orf47(dist=171175)//intergenic | 29 | chr9 | 91434603 | + | AY123041 | 2397 | - | 9q22.1 |
| 15T | 20C211253_R303_CapNGS | HBVCgp4_PREC/C | CDR2//intronic | 29 | chr16 | 22365843 | + | AY123041 | 1810 | + | 16p12.2 |
| 15T | 20C211253_R303_CapNGS | HBVCgp4_PREC/C | MBD3(dist=2407),UQCR11(dist=1897)//intergenic | 35 | chr19 | 1595271 | + | AY123041 | 2266 | + | 19p13.3 |
| 15T | 20C211253_R303_CapNGS | HBVCgp4_PREC/C | LINC00433(dist=77834),LINC00560(dist=3509)//intergenic | 35 | chr13 | 89275598 | - | AY123041 | 1718 | + | 13q31.2 |
| 15T | 20C211253_R303_CapNGS | HBVCgp2_PRES1/PRES2/S;HBVCgp1_P;HBVCgp3_X;HBVCgp4_PREC/C//upstream;downstream | LINC00680-GUSBP4(dist=488878),NONE(dist=NONE)//intergenic | 42 | chr6 | 58776602 | + | AY123041 | 2375 | - | 6p11.1 |
| 15T | 20C211253_R303_CapNGS | HBVCgp4_PREC/C | ZNF423(dist=163918),CNEP1R1(dist=3369)//intergenic | 47 | chr16 | 50055748 | + | AY123041 | 1826 | - | 16q12.1 |
| 15T | 20C211253_R303_CapNGS | HBVCgp4_PREC/C | NONE(dist=NONE),ANKRD30BP2(dist=50924)//intergenic | 64 | chr21 | 14359563 | + | AY123041 | 2123 | + | 21q11.2 |
| 15T | 20C211253_R303_CapNGS | HBVCgp3_X | ESRRG//intronic | 203 | chr1 | 216706530 | + | AY123041 | 1677 | - | 1q41 |
| 15T | 20C211253_R303_CapNGS | HBVCgp4_PREC/C | NDFIP2//intronic | 330 | chr13 | 80063910 | + | AY123041 | 1778 | + | 13q31.1 |
| 15T | 20C211253_R303_CapNGS | HBVCgp4_PREC/C | NDFIP2//intronic | 331 | chr13 | 80063912 | + | AY123041 | 1778 | + | 13q31.1 |
| 15T | 20C211253_R303_CapNGS | HBVCgp4_PREC/C | ZXDA(dist=628671),NONE(dist=NONE)//intergenic | 1580 | chrX | 58565738 | + | AY123041 | 2124 | + | Xp11.1 |
| 15T | 20C211253_R303_CapNGS | HBVCgp3_X | CSNK1A1L(dist=39880),LINC01048(dist=335920)//intergenic | 3380 | chr13 | 37719681 | + | AY123041 | 1476 | - | 13q13.3 |
| 15T | 20C211253_R303_CapNGS | HBVCgp3_X;HBVCgp4_PREC/C | PMAIP1(dist=310928),MC4R(dist=155830)//intergenic | 11697 | chr18 | 57882465 | + | AY123041 | 1690 | - | 18q21.32 |
| 15T | 20C211253_R303_CapNGS | HBVCgp3_X;HBVCgp4_PREC/C | PMAIP1(dist=310927),MC4R(dist=155831)//intergenic | 11726 | chr18 | 57882464 | - | AY123041 | 1691 | + | 18q21.32 |
| 15T | 20C211253_R303_CapNGS | HBVCgp3_X;HBVCgp4_PREC/C | PMAIP1(dist=310924),MC4R(dist=155834)//intergenic | 11726 | chr18 | 57882461 | - | AY123041 | 1691 | + | 18q21.32 |
| 15T | 20C211253_R303_CapNGS | HBVCgp3_X;HBVCgp4_PREC/C | PMAIP1(dist=310923),MC4R(dist=155835)//intergenic | 11726 | chr18 | 57882460 | - | AY123041 | 1691 | + | 18q21.32 |
| 15T | 20C211253_R303_CapNGS | HBVCgp4_PREC/C | PMAIP1(dist=310967),MC4R(dist=155791)//intergenic | 12047 | chr18 | 57882504 | + | AY123041 | 1863 | - | 18q21.32 |
| 16T | 20C211254_R303_CapNGS | HBVCgp3_X | PSD2-AS1(dist=20979),PSD2(dist=28606)//intergenic | 5 | chr5 | 139146787 | + | AY123041 | 1684 | + | 5q31.2 |
| 16T | 20C211254_R303_CapNGS | NONE(dist=NONE),HBVCgp3_X(dist=1085)//intergenic | LINC00299//ncRNA_intronic | 5 | chr2 | 8313059 | + | AY123041 | 163 | + | 2p25.1 |
| 16T | 20C211254_R303_CapNGS | HBVCgp3_X | PPP4R3A//intronic | 5 | chr14 | 91955460 | + | AY123041 | 1684 | + | 14q32.12 |
| 16T | 20C211254_R303_CapNGS | HBVCgp3_X | TMEM135//intronic | 5 | chr11 | 86851318 | + | AY123041 | 1685 | + | 11q14.2 |
| 16T | 20C211254_R303_CapNGS | HBVCgp3_X | SUCLG2//intronic | 6 | chr3 | 67686985 | + | AY123041 | 1684 | + | 3p14.1 |

| | | | | | | | | | | | |
|---|---|---|---|---|---|---|---|---|---|---|---|
| 16T | 20C211254_R303_CapNGS | HBVCgp3_X | FBN3//intronic | 6 | chr19 | 8164372 | + | AY123041 | 1679 | + | 19p13.2 |
| 16T | 20C211254_R303_CapNGS | HBVCgp3_X | DCDC1//intronic | 6 | chr11 | 30975771 | + | AY123041 | 1684 | + | 11p14.1 |
| 16T | 20C211254_R303_CapNGS | HBVCgp3_X | CCSER2//intronic | 8 | chr10 | 86196198 | + | AY123041 | 1685 | - | 10q23.1 |
| 16T | 20C211254_R303_CapNGS | HBVCgp3_X | PLPPR4(dist=124757),LINC01708(dist=38081)//intergenic | 8 | chr1 | 99899895 | + | AY123041 | 1684 | - | 1p21.2 |
| 16T | 20C211254_R303_CapNGS | NONE(dist=NONE),HBVCgp3_X(dist=1153)//intergenic | LINC01987(dist=85652),LINC01973(dist=64790)//intergenic | 9 | chr17 | 75810293 | + | AY123041 | 95 | + | 17q25.3 |
| 16T | 20C211254_R303_CapNGS | HBVCgp3_X | KRTAP5-9(dist=3353),KRTAP5-10(dist=12603)//intergenic | 13 | chr11 | 71264006 | + | AY123041 | 1684 | + | 11q13.4 |
| 16T | 20C211254_R303_CapNGS | HBVCgp3_X | ZNF843(dist=7223),ARMC5(dist=7921)//intergenic | 18 | chr16 | 31461704 | + | AY123041 | 1684 | - | 16p11.2 |
| 16T | 20C211254_R303_CapNGS | HBVCgp3_X | FAM189A1//intronic | 18 | chr15 | 29771205 | + | AY123041 | 1684 | - | 15q13.1 |
| 16T | 20C211254_R303_CapNGS | HBVCgp3_X | FAM117B//intronic | 19 | chr2 | 203531577 | + | AY123041 | 1684 | + | 2q33.2 |
| 16T | 20C211254_R303_CapNGS | HBVCgp3_X | DIAPH2(dist=929614),PCDH19(dist=1757033)//intergenic | 22 | chrX | 97789609 | + | AY123041 | 1684 | - | Xq21.33 |
| 16T | 20C211254_R303_CapNGS | HBVCgp3_X | SMARCB1//intronic | 29 | chr22 | 24157528 | + | AY123041 | 1684 | - | 22q11.23 |
| 16T | 20C211254_R303_CapNGS | HBVCgp3_X | DDI2//intronic | 87 | chr1 | 15957547 | + | AY123041 | 1684 | + | 1p36.21 |
| 16T | 20C211254_R303_CapNGS | HBVCgp3_X | SDK1//intronic | 88 | chr7 | 4062615 | + | AY123041 | 1684 | + | 7p22.2 |
| 16T | 20C211254_R303_CapNGS | HBVCgp3_X | SDK1(dist=404668),FOXK1(dist=8627)//intergenic | 327 | chr7 | 4713299 | + | AY123041 | 1684 | - | 7p22.1 |
| 16T | 20C211254_R303_CapNGS | HBVCgp3_X | FBN3//intronic | 536 | chr19 | 8164373 | + | AY123041 | 1684 | + | 19p13.2 |
| 16T | 20C211254_R303_CapNGS | HBVCgp3_X | SDK1(dist=404667),FOXK1(dist=8628)//intergenic | 633 | chr7 | 4713298 | + | AY123041 | 1684 | - | 7p22.1 |
| 16T | 20C211254_R303_CapNGS | HBVCgp3_X | DTNB(dist=20282),ASXL2(dist=39797)//intergenic | 664 | chr2 | 25916825 | + | AY123041 | 1684 | - | 2p23.3 |
| 16T | 20C211254_R303_CapNGS | HBVCgp4_PREC/C | CDR2//intronic | 8318 | chr16 | 22365848 | + | AY123041 | 1804 | + | 16p12.2 |
| 16T | 20C211254_R303_CapNGS | HBVCgp4_PREC/C | CDR2//intronic | 8321 | chr16 | 22365844 | + | AY123041 | 1801 | + | 16p12.2 |
| 16T | 20C211254_R303_CapNGS | HBVCgp4_PREC/C | CDR2//intronic | 8331 | chr16 | 22365847 | + | AY123041 | 1805 | + | 16p12.2 |
| 16T | 20C211254_R303_CapNGS | HBVCgp4_PREC/C | CDR2//intronic | 8334 | chr16 | 22365846 | + | AY123041 | 1801 | + | 16p12.2 |
| 16T | 20C211254_R303_CapNGS | NONE(dist=NONE),HBVCgp3_X(dist=1150)//intergenic | AIDA//intronic | 13451 | chr1 | 222884859 | + | AY123041 | 98 | - | 1q41 |
| 16T | 20C211254_R303_CapNGS | HBVCgp3_X | DISC1FP1(dist=276600),FAT3(dist=1033380)//intergenic | 13736 | chr11 | 90924820 | + | AY123041 | 1309 | - | 11q14.3 |
| 16T | 20C211254_R303_CapNGS | HBVCgp4_PREC/C | ADCY5//intronic | 23818 | chr3 | 123045761 | + | AY123041 | 1888 | - | 3q21.1 |
| 16T | 20C211254_R303_CapNGS | HBVCgp4_PREC/C | ADCY5//intronic | 24794 | chr3 | 123045757 | + | AY123041 | 1888 | - | 3q21.1 |
| 17T | 20C211255_R303_CapNGS | HBVCgp4_PREC/C//downstream | ANKRD31(dist=26175),HMGCR(dist=74115)//intergenic | 5 | chr5 | 74558878 | - | AY123041 | 3038 | + | 5q13.3 |
| 17T | 20C211255_R303_CapNGS | HBVCgp4_PREC/C//downstream | CCDC85A(dist=445041),VRK2(dist=1076436)//intergenic | 115 | chr2 | 57058350 | + | AY123041 | 2939 | + | 2p16.1 |
| 17T | 20C211255_R303_CapNGS | HBVCgp3_X | MYH10//intronic | 28203 | chr17 | 8399009 | + | AY123041 | 1360 | - | 17p13.1 |
| 17T | 20C211255_R303_CapNGS | HBVCgp3_X;HBVCgp4_PREC/C//upstream | WDR45B//UTR3 | 58993 | chr17 | 80573785 | - | AY123041 | 851 | + | 17q25.3 |
| 17T | 20C211255_R303_CapNGS | HBVCgp3_X;HBVCgp4_PREC/C//upstream | WDR45B//UTR3 | 58993 | chr17 | 80573781 | - | AY123041 | 851 | + | 17q25.3 |
| 17T | 20C211255_R303_CapNGS | HBVCgp3_X;HBVCgp4_PREC/C//upstream | WDR45B//UTR3 | 58993 | chr17 | 80573783 | - | AY123041 | 851 | + | 17q25.3 |
| 17T | 20C211255_R303_CapNGS | HBVCgp3_X;HBVCgp4_PREC/C//upstream | WDR45B//UTR3 | 58997 | chr17 | 80573787 | + | AY123041 | 850 | - | 17q25.3 |

| | | | | | | | | | | | |
|---|---|---|---|---|---|---|---|---|---|---|---|
| 17T | 20C211255_R303_CapNGS | HBVCgp3_X;HBVCgp4_PREC/C//upstream | WDR45B//UTR3 | 59820 | chr17 | 80573782 | + | AY123041 | 850 | - | 17q25.3 |
| 17T | 20C211255_R303_CapNGS | HBVCgp3_X | MIEF2//intronic | 75062 | chr17 | 18165337 | + | AY123041 | 1674 | + | 17p11.2 |
| 17T | 20C211255_R303_CapNGS | HBVCgp3_X | MIEF2//intronic | 75063 | chr17 | 18165336 | + | AY123041 | 1674 | + | 17p11.2 |
| 18T | 20C211256_R303_CapNGS | HBVCgp3_X | CNTNAP2//intronic | 7 | chr7 | 147465233 | + | AY123041 | 1418 | - | 7q35 |
| 18T | 20C211256_R303_CapNGS | HBVCgp3_X//upstream | DHX35//intronic | 7 | chr20 | 37657813 | + | AY123041 | 403 | + | 20q12 |
| 18T | 20C211256_R303_CapNGS | NONE(dist=NONE),HBVCgp3_X(dist=1063)//intergenic | MIR8054(dist=9139),LUZP2(dist=1068724)//intergenic | 11 | chr11 | 23449875 | + | AY123041 | 185 | - | 11p14.3 |
| 18T | 20C211256_R303_CapNGS | HBVCgp3_X;HBVCgp4_PREC/C//upstream | LOC101927066//ncRNA_intronic | 24 | chr8 | 98219078 | + | AY123041 | 727 | - | 8q22.1 |
| 18T | 20C211256_R303_CapNGS | HBVCgp3_X//upstream | RBM14//exonic//RBM14:NM_006328:exon2:c.1236 | 37 | chr11 | 66392583 | + | AY123041 | 296 | - | 11q13.2 |
| 18T | 20C211256_R303_CapNGS | HBVCgp3_X//upstream | NBEA//intronic | 60 | chr13 | 35936765 | + | AY123041 | 522 | - | 13q13.3 |
| 18T | 20C211256_R303_CapNGS | HBVCgp3_X//upstream | CNTN1(dist=40236),PDZRN4(dist=75669)//intergenic | 139 | chr12 | 41506453 | - | AY123041 | 549 | + | 12q12 |
| 18T | 20C211256_R303_CapNGS | HBVCgp3_X;HBVCgp4_PREC/C | TERT//UTR5 | 145 | chr5 | 1295176 | + | AY123041 | 1698 | - | 5p15.33 |
| 18T | 20C211256_R303_CapNGS | NONE(dist=NONE),HBVCgp3_X(dist=1009)//intergenic | LINC01108(dist=91644),JARID2(dist=868971)//intergenic | 213 | chr6 | 14377329 | + | AY123041 | 239 | - | 6p23 |
| 18T | 20C211256_R303_CapNGS | NONE(dist=NONE),HBVCgp3_X(dist=1247)//intergenic | TERT//intronic | 68580 | chr5 | 1270872 | + | AY123041 | 1 | + | 5p15.33 |
| 18T | 20C211256_R303_CapNGS | NONE(dist=NONE),HBVCgp3_X(dist=1247)//intergenic | TERT//intronic | 68580 | chr5 | 1270871 | + | AY123041 | 1 | + | 5p15.33 |
| 18T | 20C211256_R303_CapNGS | HBVCgp3_X | TERT//intronic | 68580 | chr5 | 1270897 | + | AY123041 | 1659 | + | 5p15.33 |
| 18T | 20C211256_R303_CapNGS | HBVCgp3_X | TERT//intronic | 68580 | chr5 | 1270902 | + | AY123041 | 1659 | + | 5p15.33 |
| 18T | 20C211256_R303_CapNGS | HBVCgp3_X | TERT//intronic | 68581 | chr5 | 1270899 | + | AY123041 | 1659 | + | 5p15.33 |
| 18T | 20C211256_R303_CapNGS | HBVCgp3_X | TERT//intronic | 68582 | chr5 | 1270900 | + | AY123041 | 1659 | + | 5p15.33 |
| 18T | 20C211256_R303_CapNGS | HBVCgp3_X | TERT//intronic | 68584 | chr5 | 1270898 | + | AY123041 | 1661 | + | 5p15.33 |
| 18T | 20C211256_R303_CapNGS | NONE(dist=NONE),HBVCgp3_X(dist=1247)//intergenic | TERT//intronic | 68590 | chr5 | 1270874 | + | AY123041 | 1 | + | 5p15.33 |
| 18T | 20C211256_R303_CapNGS | NONE(dist=NONE),HBVCgp3_X(dist=1247)//intergenic | TERT//intronic | 70555 | chr5 | 1270876 | + | AY123041 | 1 | + | 5p15.33 |
| 19T | 20C211257_R303_CapNGS | HBVCgp4_PREC/C | ZNF804B(dist=253062),STEAP2-AS1(dist=290763)//intergenic | 6 | chr7 | 89220904 | + | AY123041 | 1745 | + | 7q21.13 |
| 19T | 20C211257_R303_CapNGS | HBVCgp2_PRES1/PRES2/S;HBVCgp3_X;HBVCgp4_PREC/C//upstream;downstream | GPHN//intronic | 6 | chr14 | 67348630 | + | AY123041 | 2695 | - | 14q23.3 |
| 19T | 20C211257_R303_CapNGS | HBVCgp2_PRES1/PRES2/S;HBVCgp3_X;HBVCgp4_PREC/C//upstream;downstream | LINC02520//ncRNA_intronic | 10 | chr6 | 37499033 | + | AY123041 | 2678 | + | 6p21.2 |
| 19T | 20C211257_R303_CapNGS | HBVCgp3_X;HBVCgp4_PREC/C | TMEFF2(dist=422721),PCGEM1(dist=132191)//intergenic | 116 | chr2 | 193482380 | + | AY123041 | 1700 | - | 2q32.3 |

| | | | | | | | | | | | |
|---|---|---|---|---|---|---|---|---|---|---|---|
| 19T | 20C211257_R303_CapNGS | HBVCgp3_X;HBVCgp4_PREC/C//upstream | TMEM161B-AS1//ncRNA_intronic | 235 | chr5 | 87649030 | + | AY123041 | 737 | + | 5q14.3 |
| 19T | 20C211257_R303_CapNGS | HBVCgp3_X;HBVCgp4_PREC/C | DBET(dist=55147),NONE(dist=NONE)//intergenic | 639 | chr4 | 191044166 | - | AY123041 | 1702 | + | 4q35.2 |
| 19T | 20C211257_R303_CapNGS | HBVCgp3_X;HBVCgp4_PREC/C | DBET(dist=55142),NONE(dist=NONE)//intergenic | 649 | chr4 | 191044161 | - | AY123041 | 1704 | + | 4q35.2 |
| 19T | 20C211257_R303_CapNGS | HBVCgp3_X;HBVCgp4_PREC/C | DBET(dist=55148),NONE(dist=NONE)//intergenic | 651 | chr4 | 191044167 | + | AY123041 | 1701 | - | 4q35.2 |
| 19T | 20C211257_R303_CapNGS | HBVCgp3_X;HBVCgp4_PREC/C | DBET(dist=55160),NONE(dist=NONE)//intergenic | 655 | chr4 | 191044179 | + | AY123041 | 1704 | - | 4q35.2 |
| 19T | 20C211257_R303_CapNGS | HBVCgp2_PRES1/PRES2/S;HBVCgp1_P;HBVCgp3_X;HBVCgp4_PREC/C//upstream;downstream | NONE(dist=NONE),ANKRD26P1(dist=113789)//intergenic | 8014 | chr16 | 46389460 | + | AY123041 | 2368 | - | 16q11.2 |
| 19T | 20C211257_R303_CapNGS | HBVCgp2_PRES1/PRES2/S;HBVCgp1_P;HBVCgp3_X;HBVCgp4_PREC/C//upstream;downstream | NONE(dist=NONE),ANKRD26P1(dist=113780)//intergenic | 8325 | chr16 | 46389469 | + | AY123041 | 2368 | - | 16q11.2 |
| 19T | 20C211257_R303_CapNGS | HBVCgp2_PRES1/PRES2/S;HBVCgp1_P;HBVCgp3_X;HBVCgp4_PREC/C//upstream;downstream | NONE(dist=NONE),ANKRD26P1(dist=113782)//intergenic | 8398 | chr16 | 46389467 | - | AY123041 | 2371 | + | 16q11.2 |
| 19T | 20C211257_R303_CapNGS | HBVCgp3_X;HBVCgp4_PREC/C | FAM157A;FAM157B//ncRNA_intronic | 9067 | chr3 | 197900290 | + | AY123041 | 1711 | - | 3q29 |
| 19T | 20C211257_R303_CapNGS | HBVCgp3_X;HBVCgp4_PREC/C | WASH8P(dist=4186),IQSEC3(dist=80484)//intergenic | 12741 | chr12 | 95449 | - | AY123041 | 1701 | + | 12p13.33 |
| 19T | 20C211257_R303_CapNGS | HBVCgp3_X;HBVCgp4_PREC/C | WASH8P(dist=4194),IQSEC3(dist=80476)//intergenic | 12758 | chr12 | 95457 | + | AY123041 | 1701 | - | 12p13.33 |
| 19T | 20C211257_R303_CapNGS | HBVCgp3_X;HBVCgp4_PREC/C | WASH8P(dist=4187),IQSEC3(dist=80483)//intergenic | 12765 | chr12 | 95450 | + | AY123041 | 1700 | - | 12p13.33 |
| 19T | 20C211257_R303_CapNGS | HBVCgp3_X;HBVCgp4_PREC/C | WASH8P(dist=4191),IQSEC3(dist=80479)//intergenic | 12767 | chr12 | 95454 | + | AY123041 | 1700 | - | 12p13.33 |
| 19T | 20C211257_R303_CapNGS | HBVCgp3_X;HBVCgp4_PREC/C | WASH8P(dist=4179),IQSEC3(dist=80491)//intergenic | 12767 | chr12 | 95442 | + | AY123041 | 1700 | - | 12p13.33 |
| 19T | 20C211257_R303_CapNGS | HBVCgp3_X;HBVCgp4_PREC/C | WASH8P(dist=4182),IQSEC3(dist=80488)//intergenic | 12841 | chr12 | 95445 | + | AY123041 | 1700 | - | 12p13.33 |
| 19T | 20C211257_R303_CapNGS | HBVCgp3_X;HBVCgp4_PREC/C | WASH8P(dist=4170),IQSEC3(dist=80500)//intergenic | 12848 | chr12 | 95433 | + | AY123041 | 1704 | - | 12p13.33 |
| 19T | 20C211257_R303_CapNGS | HBVCgp3_X;HBVCgp4_PREC/C | WASH8P(dist=4176),IQSEC3(dist=80494)//intergenic | 12867 | chr12 | 95439 | + | AY123041 | 1700 | - | 12p13.33 |
| 20T | 20C211258_R303_CapNGS | HBVBgp3_X | SAMD5(dist=421892),SASH1(dist=280415)//intergenic | 5 | chr6 | 148313049 | - | AF100309 | 1836 | + | 6q24.3 |
| 20T | 20C211258_R303_CapNGS | HBVBgp3_X;HBVBgp4_C;HBVBgp2_S//upstream;downstream | LOC105373394(dist=242886),LINC01249(dist=410052)//intergenic | 6 | chr2 | 4265756 | - | AF100309 | 1249 | + | 2p25.3 |
| 20T | 20C211258_R303_CapNGS | HBVBgp3_X | TERT//upstream | 9 | chr5 | 1295933 | - | AF100309 | 1795 | + | 5p15.33 |
| 20T | 20C211258_R303_CapNGS | HBVBgp3_X | TERT//upstream | 9 | chr5 | 1295939 | - | AF100309 | 1795 | + | 5p15.33 |
| 20T | 20C211258_R303_CapNGS | HBVBgp3_X | SLC66A1L(dist=327383),SHOX2(dist=167396)//intergenic | 10 | chr3 | 157646404 | + | AF100309 | 1506 | + | 3q25.32 |
| 20T | 20C211258_R303_CapNGS | HBVBgp3_X | TERT//upstream | 11 | chr5 | 1295936 | - | AF100309 | 1795 | + | 5p15.33 |
| 20T | 20C211258_R303_CapNGS | HBVBgp3_X | TERT//upstream | 11 | chr5 | 1295931 | - | AF100309 | 1795 | + | 5p15.33 |

| | | | | | | | | | | | |
|---|---|---|---|---|---|---|---|---|---|---|---|
| 20T | 20C211258_R303_CapNGS | HBVBgp3_X | SLC66A1L(dist=327378),SHOX2(dist=167401)//intergenic | 11 | chr3 | 157646399 | + | AF100309 | 1506 | + | 3q25.32 |
| 20T | 20C211258_R303_CapNGS | HBVBgp3_X | TERT//upstream | 12 | chr5 | 1295932 | - | AF100309 | 1795 | + | 5p15.33 |
| 20T | 20C211258_R303_CapNGS | HBVBgp3_X | TERT//upstream | 12 | chr5 | 1295935 | - | AF100309 | 1795 | + | 5p15.33 |
| 20T | 20C211258_R303_CapNGS | HBVBgp3_X | TERT//upstream | 14 | chr5 | 1295934 | - | AF100309 | 1795 | + | 5p15.33 |
| 20T | 20C211258_R303_CapNGS | HBVBgp3_X | TERT//upstream | 16 | chr5 | 1295937 | - | AF100309 | 1795 | + | 5p15.33 |
| 20T | 20C211258_R303_CapNGS | HBVBgp3_X | TERT//upstream | 17 | chr5 | 1295938 | - | AF100309 | 1795 | + | 5p15.33 |
| 20T | 20C211258_R303_CapNGS | HBVBgp3_X | TERT//upstream | 21 | chr5 | 1295940 | - | AF100309 | 1795 | + | 5p15.33 |
| 20T | 20C211258_R303_CapNGS | HBVBgp3_X | TRMT1L//intronic | 23 | chr1 | 185096278 | - | AF100309 | 1705 | + | 1q25.3 |
| 20T | 20C211258_R303_CapNGS | HBVBgp3_X | TRMT1L//intronic | 24 | chr1 | 185096274 | - | AF100309 | 1705 | + | 1q25.3 |
| 20T | 20C211258_R303_CapNGS | HBVBgp3_X | TRMT1L//intronic | 24 | chr1 | 185096281 | - | AF100309 | 1705 | + | 1q25.3 |
| 20T | 20C211258_R303_CapNGS | HBVBgp3_X | TRMT1L//intronic | 25 | chr1 | 185096276 | - | AF100309 | 1705 | + | 1q25.3 |
| 20T | 20C211258_R303_CapNGS | HBVBgp3_X | TRMT1L//intronic | 27 | chr1 | 185096277 | - | AF100309 | 1705 | + | 1q25.3 |
| 20T | 20C211258_R303_CapNGS | HBVBgp3_X | TRMT1L//intronic | 33 | chr1 | 185096279 | - | AF100309 | 1705 | + | 1q25.3 |
| 20T | 20C211258_R303_CapNGS | HBVBgp3_X;HBVBgp4_C;HBVBgp2_S//upstream;downstream | TERT(dist=3674),MIR4457(dist=10568)//intergenic | 5744 | chr5 | 1298857 | - | AF100309 | 1075 | + | 5p15.33 |
| 20T | 20C211258_R303_CapNGS | HBVBgp3_X;HBVBgp4_C;HBVBgp2_S//upstream;downstream | TERT(dist=3667),MIR4457(dist=10575)//intergenic | 5744 | chr5 | 1298850 | - | AF100309 | 1075 | + | 5p15.33 |
| 20T | 20C211258_R303_CapNGS | HBVBgp3_X;HBVBgp4_C;HBVBgp2_S//upstream;downstream | TERT(dist=3661),MIR4457(dist=10581)//intergenic | 5746 | chr5 | 1298844 | - | AF100309 | 1075 | + | 5p15.33 |
| 20T | 20C211258_R303_CapNGS | HBVBgp3_X;HBVBgp4_C;HBVBgp2_S//upstream;downstream | TERT(dist=3664),MIR4457(dist=10578)//intergenic | 5748 | chr5 | 1298847 | - | AF100309 | 1075 | + | 5p15.33 |
| 20T | 20C211258_R303_CapNGS | HBVBgp3_X;HBVBgp4_C;HBVBgp2_S//upstream;downstream | TERT(dist=3662),MIR4457(dist=10580)//intergenic | 5748 | chr5 | 1298845 | - | AF100309 | 1075 | + | 5p15.33 |
| 20T | 20C211258_R303_CapNGS | HBVBgp3_X;HBVBgp4_C;HBVBgp2_S//upstream;downstream | TERT(dist=3660),MIR4457(dist=10582)//intergenic | 5748 | chr5 | 1298843 | - | AF100309 | 1075 | + | 5p15.33 |
| 20T | 20C211258_R303_CapNGS | HBVBgp3_X;HBVBgp4_C;HBVBgp2_S//upstream;downstream | TERT(dist=3655),MIR4457(dist=10587)//intergenic | 5748 | chr5 | 1298838 | - | AF100309 | 1075 | + | 5p15.33 |
| 20T | 20C211258_R303_CapNGS | HBVBgp3_X;HBVBgp4_C;HBVBgp2_S//upstream;downstream | TERT(dist=3652),MIR4457(dist=10590)//intergenic | 5755 | chr5 | 1298835 | - | AF100309 | 1075 | + | 5p15.33 |
| 21T | 20C211259_R303_CapNGS | HBVBgp3_X;HBVBgp4_C;HBVBgp2_S//upstream;downstream | POU1F1(dist=114829),HTR1F(dist=401293)//intergenic | 5 | chr3 | 87440563 | + | AF100309 | 1141 | - | 3p11.2 |
| 21T | 20C211259_R303_CapNGS | HBVBgp2_S | PDHX(dist=7193),LOC100507144(dist=129329)//intergenic | 6 | chr11 | 35024868 | + | AF100309 | 712 | + | 11p13 |
| 21T | 20C211259_R303_CapNGS | HBVBgp3_X;HBVBgp2_S//upstream;downstream | ACTN2(dist=10043),MTR(dist=20611)//intergenic | 6 | chr1 | 236937970 | + | AF100309 | 863 | + | 1q43 |
| 21T | 20C211259_R303_CapNGS | HBVBgp2_S | CCDC82//intronic | 11 | chr11 | 96091493 | + | AF100309 | 396 | + | 11q21 |
| 21T | 20C211259_R303_CapNGS | HBVBgp1_P;HBVBgp4_C;HBVBgp3_X//upstream;downstream | LINC00544(dist=149028),LINC00365(dist=3662)//intergenic | 16 | chr13 | 30673653 | - | AF100309 | 1875 | + | 13q12.3 |

| | | | | | | | | | | | |
|---|---|---|---|---|---|---|---|---|---|---|---|
| 21T | 20C211259_R303_CapNGS | HBVBgp1_P;HBVBgp3_X;HBVBgp4_C//downstream | LINC01170//ncRNA_intronic | 20 | chr5 | 123725983 | + | AF100309 | 2466 | - | 5q23.2 |
| 21T | 20C211259_R303_CapNGS | HBVBgp4_C | IKZF1//UTR3 | 34 | chr7 | 50437098 | + | AF100309 | 2240 | + | 7p12.2 |
| 21T | 20C211259_R303_CapNGS | HBVBgp2_S | SLITRK1(dist=16005),LINC00333(dist=242123)//intergenic | 34 | chr13 | 84472614 | + | AF100309 | 583 | + | 13q31.1 |
| 21T | 20C211259_R303_CapNGS | HBVBgp2_S | SPANXN2(dist=160232),UBE2NL(dist=3179)//intergenic | 61 | chrX | 142963994 | + | AF100309 | 837 | + | Xq27.3 |
| 21T | 20C211259_R303_CapNGS | HBVBgp2_S | LINC02267//ncRNA_intronic | 88 | chr4 | 97643192 | + | AF100309 | 782 | - | 4q22.3 |
| 21T | 20C211259_R303_CapNGS | HBVBgp3_X | UNC5D(dist=144447),KCNU1(dist=843377)//intergenic | 47346 | chr8 | 35798515 | + | AF100309 | 1686 | + | 8p12 |
| 21T | 20C211259_R303_CapNGS | HBVBgp3_X | UNC5D(dist=144445),KCNU1(dist=843379)//intergenic | 47346 | chr8 | 35798513 | + | AF100309 | 1686 | + | 8p12 |
| 22T | 20C211260_R303_CapNGS | NONE(dist=NONE),HBVCgp3_X(dist=1155)//intergenic | SOX2-OT//ncRNA_intronic | 9 | chr3 | 181386960 | + | AY123041 | 93 | + | 3q26.33 |
| 22T | 20C211260_R303_CapNGS | HBVCgp4_PREC/C | LRRC52-AS1(dist=23040),MGST3(dist=26039)//intergenic | 25 | chr1 | 165574432 | + | AY123041 | 1815 | + | 1q24.1 |
| 22T | 20C211260_R303_CapNGS | HBVCgp3_X//upstream | KCNJ2(dist=856904),CASC17(dist=60828)//intergenic | 70 | chr17 | 69033087 | - | AY123041 | 252 | + | 17q24.3 |
| 22T | 20C211260_R303_CapNGS | HBVCgp4_PREC/C | KDM4C(dist=246269),DMAC1(dist=374583)//intergenic | 15208 | chr9 | 7421917 | + | AY123041 | 1792 | - | 9p24.1 |
| 22T | 20C211260_R303_CapNGS | HBVCgp3_X//upstream | MTRNR2L1(dist=49286),NONE(dist=NONE)//intergenic | 16146 | chr17 | 22073277 | + | AY123041 | 385 | + | 17p11.2 |
| 22T | 20C211260_R303_CapNGS | HBVCgp3_X//upstream | MTRNR2L1(dist=49294),NONE(dist=NONE)//intergenic | 16234 | chr17 | 22073285 | + | AY123041 | 391 | + | 17p11.2 |
| 22T | 20C211260_R303_CapNGS | HBVCgp3_X//upstream | MTRNR2L1(dist=49291),NONE(dist=NONE)//intergenic | 16235 | chr17 | 22073282 | + | AY123041 | 391 | + | 17p11.2 |
| 22T | 20C211260_R303_CapNGS | HBVCgp3_X//upstream | MTRNR2L1(dist=49287),NONE(dist=NONE)//intergenic | 16235 | chr17 | 22073278 | + | AY123041 | 391 | + | 17p11.2 |
| 22T | 20C211260_R303_CapNGS | HBVCgp3_X//upstream | MTRNR2L1(dist=49285),NONE(dist=NONE)//intergenic | 16235 | chr17 | 22073276 | + | AY123041 | 391 | + | 17p11.2 |
| 22T | 20C211260_R303_CapNGS | HBVCgp4_PREC/C | OTUD7B(dist=52688),VPS45(dist=4076)//intergenic | 20503 | chr1 | 150035363 | - | AY123041 | 1726 | + | 1q21.2 |
| 22T | 20C211260_R303_CapNGS | HBVCgp4_PREC/C | OTUD7B(dist=52685),VPS45(dist=4079)//intergenic | 20503 | chr1 | 150035360 | - | AY123041 | 1726 | + | 1q21.2 |
| 22T | 20C211260_R303_CapNGS | HBVCgp4_PREC/C | OTUD7B(dist=52681),VPS45(dist=4083)//intergenic | 20505 | chr1 | 150035356 | + | AY123041 | 1726 | - | 1q21.2 |
| 22T | 20C211260_R303_CapNGS | HBVCgp4_PREC/C | OTUD7B(dist=52687),VPS45(dist=4077)//intergenic | 21462 | chr1 | 150035362 | - | AY123041 | 1723 | + | 1q21.2 |
| 22T | 20C211260_R303_CapNGS | HBVCgp4_PREC/C | OTUD7B(dist=52686),VPS45(dist=4078)//intergenic | 21462 | chr1 | 150035361 | - | AY123041 | 1723 | + | 1q21.2 |
| 22T | 20C211260_R303_CapNGS | HBVCgp4_PREC/C | OTUD7B(dist=52683),VPS45(dist=4081)//intergenic | 21462 | chr1 | 150035358 | - | AY123041 | 1723 | + | 1q21.2 |
| 22T | 20C211260_R303_CapNGS | HBVCgp4_PREC/C | OTUD7B(dist=52684),VPS45(dist=4080)//intergenic | 21463 | chr1 | 150035359 | - | AY123041 | 1723 | + | 1q21.2 |
| 22T | 20C211260_R303_CapNGS | HBVCgp4_PREC/C | OTUD7B(dist=52682),VPS45(dist=4082)//intergenic | 21466 | chr1 | 150035357 | + | AY123041 | 1723 | - | 1q21.2 |
| 22T | 20C211260_R303_CapNGS | HBVCgp4_PREC/C | OTUD7B(dist=52679),VPS45(dist=4085)//intergenic | 21645 | chr1 | 150035354 | + | AY123041 | 1724 | - | 1q21.2 |

| | | | | | | | | | | | |
|---|---|---|---|---|---|---|---|---|---|---|---|
| 23T | 20C211261_R303_CapNGS | HBVBgp2_S | INHBB(dist=48323),LINC01101(dist=64209)//intergenic | 5 | chr2 | 121157702 | + | AF100309 | 265 | - | 2q14.2 |
| 23T | 20C211261_R303_CapNGS | HBVBgp3_X | LINC01915//ncRNA_intronic | 6 | chr18 | 22228612 | + | AF100309 | 1786 | + | 18q11.2 |
| 23T | 20C211261_R303_CapNGS | HBVBgp4_C | SEL1L3(dist=16925),SMIM20(dist=33701)//intergenic | 8 | chr4 | 25882113 | - | AF100309 | 2237 | + | 4p15.2 |
| 23T | 20C211261_R303_CapNGS | HBVBgp4_C//downstream | LINC01255(dist=88182),SLC35G4(dist=14393)//intergenic | 14 | chr18 | 11595164 | + | AF100309 | 3049 | - | 18p11.21 |
| 23T | 20C211261_R303_CapNGS | HBVBgp3_X;HBVBgp4_C;HBVBgp2_S//upstream;downstream | MNS1(dist=35009),ZNF280D(dist=130030)//intergenic | 93 | chr15 | 56792344 | + | AF100309 | 1249 | - | 15q21.3 |
| 23T | 20C211261_R303_CapNGS | HBVBgp2_S | EDIL3//intronic | 34592 | chr5 | 83665702 | + | AF100309 | 161 | + | 5q14.3 |
| 23T | 20C211261_R303_CapNGS | HBVBgp3_X | KMT2B//intronic | 76445 | chr19 | 36212866 | + | AF100309 | 1646 | + | 19q13.12 |
| 23T | 20C211261_R303_CapNGS | HBVBgp3_X | KMT2B//intronic | 76445 | chr19 | 36212867 | + | AF100309 | 1652 | + | 19q13.12 |
| 23T | 20C211261_R303_CapNGS | HBVBgp1_P;HBVBgp3_X;HBVBgp4_C//downstream | KMT2B//intronic | 76448 | chr19 | 36212893 | + | AF100309 | 2459 | + | 19q13.12 |
| 23T | 20C211261_R303_CapNGS | HBVBgp1_P;HBVBgp3_X;HBVBgp4_C//downstream | KMT2B//intronic | 76473 | chr19 | 36212907 | + | AF100309 | 2465 | + | 19q13.12 |
| 23T | 20C211261_R303_CapNGS | HBVBgp1_P;HBVBgp3_X;HBVBgp4_C//downstream | KMT2B//intronic | 76490 | chr19 | 36212906 | + | AF100309 | 2459 | + | 19q13.12 |
| 23T | 20C211261_R303_CapNGS | HBVBgp1_P;HBVBgp3_X;HBVBgp4_C//downstream | KMT2B//intronic | 77165 | chr19 | 36212894 | + | AF100309 | 2459 | + | 19q13.12 |
| 23T | 20C211261_R303_CapNGS | HBVBgp3_X | KMT2B//intronic | 77203 | chr19 | 36212869 | + | AF100309 | 1646 | + | 19q13.12 |
| 23T | 20C211261_R303_CapNGS | HBVBgp1_P;HBVBgp3_X;HBVBgp4_C//downstream | KMT2B//intronic | 79271 | chr19 | 36212897 | + | AF100309 | 2470 | + | 19q13.12 |
| 23T | 20C211261_R303_CapNGS | HBVBgp1_P;HBVBgp3_X;HBVBgp4_C//downstream | KMT2B//intronic | 79271 | chr19 | 36212912 | + | AF100309 | 2470 | + | 19q13.12 |
| 23T | 20C211261_R303_CapNGS | HBVBgp1_P;HBVBgp3_X;HBVBgp4_C//downstream | KMT2B//intronic | 79271 | chr19 | 36212913 | + | AF100309 | 2470 | + | 19q13.12 |
| 23T | 20C211261_R303_CapNGS | HBVBgp1_P;HBVBgp3_X;HBVBgp4_C//downstream | KMT2B//intronic | 79272 | chr19 | 36212905 | + | AF100309 | 2470 | + | 19q13.12 |
| 23T | 20C211261_R303_CapNGS | HBVBgp1_P;HBVBgp3_X;HBVBgp4_C//downstream | KMT2B//intronic | 79287 | chr19 | 36212909 | + | AF100309 | 2470 | + | 19q13.12 |
| 23T | 20C211261_R303_CapNGS | HBVBgp1_P;HBVBgp3_X;HBVBgp4_C//downstream | KMT2B//intronic | 79296 | chr19 | 36212908 | + | AF100309 | 2470 | + | 19q13.12 |
| 23T | 20C211261_R303_CapNGS | HBVBgp3_X;HBVBgp4_C;HBVBgp2_S//upstream;downstream | PRR20B(dist=438297),PCDH17(dist=23229)//intergenic | 113848 | chr13 | 58182649 | - | AF100309 | 1146 | + | 13q21.1 |
| 23T | 20C211261_R303_CapNGS | HBVBgp3_X;HBVBgp4_C;HBVBgp2_S//upstream;downstream | PRR20B(dist=438294),PCDH17(dist=23232)//intergenic | 113849 | chr13 | 58182646 | - | AF100309 | 1146 | + | 13q21.1 |
| 23T | 20C211261_R303_CapNGS | HBVBgp3_X;HBVBgp4_C;HBVBgp2_S//upstream;downstream | PRR20B(dist=438308),PCDH17(dist=23218)//intergenic | 113851 | chr13 | 58182660 | - | AF100309 | 1146 | + | 13q21.1 |
| 23T | 20C211261_R303_CapNGS | HBVBgp3_X;HBVBgp4_C;HBVBgp2_S//upstream;downstream | PRR20B(dist=438295),PCDH17(dist=23231)//intergenic | 113851 | chr13 | 58182647 | - | AF100309 | 1146 | + | 13q21.1 |
| 23T | 20C211261_R303_CapNGS | HBVBgp3_X;HBVBgp4_C;HBVBgp2_S//upstream;downstream | PRR20B(dist=438304),PCDH17(dist=23222)//intergenic | 113853 | chr13 | 58182656 | - | AF100309 | 1146 | + | 13q21.1 |

| | | | | | | | | | | | |
|---|---|---|---|---|---|---|---|---|---|---|---|
| 23T | 20C211261_R303_CapNGS | HBVBgp1_P;HBVBgp4_C;HBVBgp3_X//upstream;downstream | PRR20B(dist=438311),PCDH17(dist=23215)//intergenic | 115170 | chr13 | 58182663 | + | AF100309 | 1883 | - | 13q21.1 |
| 23T | 20C211261_R303_CapNGS | HBVBgp1_P;HBVBgp4_C;HBVBgp3_X//upstream;downstream | PRR20B(dist=438310),PCDH17(dist=23216)//intergenic | 115171 | chr13 | 58182662 | + | AF100309 | 1883 | - | 13q21.1 |
| 23T | 20C211261_R303_CapNGS | HBVBgp2_S | LOC100505501(dist=428459),CA8(dist=633475)//intergenic | 137575 | chr8 | 60464496 | + | AF100309 | 330 | + | 8q12.1 |
| 23T | 20C211261_R303_CapNGS | HBVBgp2_S | LOC100505501(dist=428457),CA8(dist=633477)//intergenic | 137576 | chr8 | 60464494 | + | AF100309 | 321 | + | 8q12.1 |
| 23T | 20C211261_R303_CapNGS | HBVBgp2_S | LOC100505501(dist=428458),CA8(dist=633476)//intergenic | 137580 | chr8 | 60464495 | + | AF100309 | 321 | + | 8q12.1 |
| 23T | 20C211261_R303_CapNGS | HBVBgp2_S | LOC100505501(dist=428455),CA8(dist=633479)//intergenic | 139751 | chr8 | 60464492 | + | AF100309 | 321 | + | 8q12.1 |
| 24T | 20C211263_R303_CapNGS | HBVBgp3_X;HBVBgp4_C;HBVBgp2_S//upstream;downstream | LINC01173(dist=128264),SH3BP4(dist=105989)//intergenic | 6 | chr2 | 235754672 | + | AF100309 | 1089 | + | 2q37.2 |
| 24T | 20C211263_R303_CapNGS | HBVBgp2_S | ZRANB2-AS2//ncRNA_intronic | 7 | chr1 | 71670174 | + | AF100309 | 794 | + | 1p31.1 |
| 24T | 20C211263_R303_CapNGS | HBVBgp3_X | CTNNA3//intronic | 15 | chr10 | 67763356 | + | AF100309 | 1389 | + | 10q21.3 |
| 24T | 20C211263_R303_CapNGS | HBVBgp1_P;HBVBgp3_X;HBVBgp4_C//downstream | AOPEP//intronic | 23 | chr9 | 97616317 | + | AF100309 | 2585 | + | 9q22.32 |
| 24T | 20C211263_R303_CapNGS | HBVBgp2_S | SNHG5(dist=119892),HTR1E(dist=1138903)//intergenic | 43 | chr6 | 86508343 | + | AF100309 | 594 | + | 6q14.3 |
| 24T | 20C211263_R303_CapNGS | HBVBgp2_S | LINC01535//ncRNA_exonic// | 48 | chr19 | 37748035 | + | AF100309 | 243 | - | 19q13.12 |
| 24T | 20C211263_R303_CapNGS | HBVBgp3_X;HBVBgp4_C;HBVBgp2_S//upstream;downstream | LOC102724874(dist=9448),PKIA(dist=1030560)//intergenic | 53 | chr8 | 78398015 | + | AF100309 | 1137 | + | 8q21.12 |
| 24T | 20C211263_R303_CapNGS | HBVBgp3_X | CPA6(dist=89120),PREX2(dist=116566)//intergenic | 59 | chr8 | 68747715 | + | AF100309 | 1685 | - | 8q13.2 |
| 24T | 20C211263_R303_CapNGS | HBVBgp3_X | ATXN10//intronic | 80 | chr22 | 46224460 | + | AF100309 | 1680 | + | 22q13.31 |
| 24T | 20C211263_R303_CapNGS | HBVBgp3_X;HBVBgp4_C;HBVBgp2_S//upstream;downstream | NONE(dist=NONE),LOC441666(dist=439633)//intergenic | 110 | chr10 | 42387681 | + | AF100309 | 1142 | + | 10q11.21 |
| 24T | 20C211263_R303_CapNGS | HBVBgp3_X | LINC01993(dist=70800),SOCS3(dist=7485)//intergenic | 112 | chr17 | 76345373 | + | AF100309 | 1791 | + | 17q25.3 |
| 24T | 20C211263_R303_CapNGS | HBVBgp3_X | HNF4G//intronic | 176 | chr8 | 76382812 | - | AF100309 | 1580 | + | 8q21.11 |
| 24T | 20C211263_R303_CapNGS | HBVBgp4_C | POTEA(dist=599869),NONE(dist=NONE)//intergenic | 616 | chr8 | 43818197 | + | AF100309 | 2410 | - | 8p11.1 |
| 24T | 20C211263_R303_CapNGS | HBVBgp2_S | HS6ST3//intronic | 6042 | chr13 | 97131484 | + | AF100309 | 416 | + | 13q32.1 |
| 24T | 20C211263_R303_CapNGS | HBVBgp3_X;HBVBgp4_C;HBVBgp2_S//upstream;downstream | GRIK2(dist=848428),HACE1(dist=1809583)//intergenic | 13759 | chr6 | 103366386 | + | AF100309 | 925 | + | 6q16.3 |
| 24T | 20C211263_R303_CapNGS | HBVBgp3_X | KMT2B//intronic | 21854 | chr19 | 36212955 | + | AF100309 | 1635 | + | 19q13.12 |
| 24T | 20C211263_R303_CapNGS | HBVBgp4_C | KMT2B//intronic | 22080 | chr19 | 36212977 | + | AF100309 | 2100 | + | 19q13.12 |
| 24T | 20C211263_R303_CapNGS | HBVBgp3_X | KMT2B//intronic | 22182 | chr19 | 36212958 | + | AF100309 | 1635 | + | 19q13.12 |
| 24T | 20C211263_R303_CapNGS | HBVBgp3_X | KMT2B//intronic | 22905 | chr19 | 36212948 | + | AF100309 | 1637 | + | 19q13.12 |
| 24T | 20C211263_R303_CapNGS | HBVBgp3_X | KMT2B//intronic | 22905 | chr19 | 36212956 | + | AF100309 | 1637 | + | 19q13.12 |
| 24T | 20C211263_R303_CapNGS | HBVBgp3_X | HCG22(dist=4384),C6orf15(dist=46961)//intergenic | 51138 | chr6 | 31032039 | + | AF100309 | 1789 | - | 6p21.33 |

| | | | | | | | | | | | |
|---|---|---|---|---|---|---|---|---|---|---|---|
| 24T | 20C211263_R303_CapNGS | HBVBgp3_X | HCG22(dist=4391),C6orf15(dist=46954)//intergenic | 51139 | chr6 | 31032046 | + | AF100309 | 1789 | - | 6p21.33 |
| 24T | 20C211263_R303_CapNGS | HBVBgp3_X | HCG22(dist=4389),C6orf15(dist=46956)//intergenic | 51950 | chr6 | 31032044 | + | AF100309 | 1789 | - | 6p21.33 |
| 24T | 20C211263_R303_CapNGS | HBVBgp3_X | HCG22(dist=4387),C6orf15(dist=46958)//intergenic | 52209 | chr6 | 31032042 | - | AF100309 | 1791 | + | 6p21.33 |
| 24T | 20C211263_R303_CapNGS | HBVBgp3_X | HCG22(dist=4388),C6orf15(dist=46957)//intergenic | 52210 | chr6 | 31032043 | - | AF100309 | 1791 | + | 6p21.33 |
| 25T | 20C211264_R303_CapNGS | HBVCgp3_X//upstream | LOC101928730(dist=7774),LINC01043(dist=32693)//intergenic | 5 | chr13 | 112935088 | + | AY123041 | 517 | + | 13q34 |
| 25T | 20C211264_R303_CapNGS | HBVCgp3_X//upstream | SLCO3A1//intronic | 6 | chr15 | 92523529 | + | AY123041 | 253 | + | 15q26.1 |
| 25T | 20C211264_R303_CapNGS | HBVCgp3_X//upstream | SIPA1L1//intronic | 6 | chr14 | 71864278 | - | AY123041 | 514 | + | 14q24.2 |
| 25T | 20C211264_R303_CapNGS | NONE(dist=NONE),HBVCgp3_X(dist=1016)//intergenic | ABCA13//intronic | 7 | chr7 | 48236468 | + | AY123041 | 232 | + | 7p12.3 |
| 25T | 20C211264_R303_CapNGS | NONE(dist=NONE),HBVCgp3_X(dist=1056)//intergenic | CDKAL1//intronic | 7 | chr6 | 21001136 | + | AY123041 | 192 | + | 6p22.3 |
| 25T | 20C211264_R303_CapNGS | HBVCgp4_PREC/C | TMEM108//intronic | 7 | chr3 | 133061490 | + | AY123041 | 2276 | - | 3q22.1 |
| 25T | 20C211264_R303_CapNGS | HBVCgp3_X//upstream | DCUN1D5(dist=3953),DYNC2H1(dist=13313)//intergenic | 7 | chr11 | 102966842 | + | AY123041 | 286 | - | 11q22.3 |
| 25T | 20C211264_R303_CapNGS | NONE(dist=NONE),HBVCgp3_X(dist=1084)//intergenic | LINC02517(dist=33039),ACOX3(dist=9278)//intergenic | 8 | chr4 | 8358731 | + | AY123041 | 164 | + | 4p16.1 |
| 25T | 20C211264_R303_CapNGS | NONE(dist=NONE),HBVCgp3_X(dist=1115)//intergenic | PCDH15//intronic | 8 | chr10 | 57039439 | + | AY123041 | 133 | + | 10q21.1 |
| 25T | 20C211264_R303_CapNGS | HBVCgp3_X//upstream | TMEM177(dist=31332),PTPN4(dist=46472)//intergenic | 9 | chr2 | 120471026 | + | AY123041 | 572 | - | 2q14.2 |
| 25T | 20C211264_R303_CapNGS | HBVCgp4_PREC/C | OR10AD1(dist=106317),H1-7(dist=19371)//intergenic | 13 | chr12 | 48703392 | + | AY123041 | 1986 | + | 12q13.11 |
| 25T | 20C211264_R303_CapNGS | NONE(dist=NONE),HBVCgp3_X(dist=1230)//intergenic | RPS6KA4(dist=41274),LINC02724(dist=35568)//intergenic | 13 | chr11 | 64180961 | + | AY123041 | 18 | + | 11q13.1 |
| 25T | 20C211264_R303_CapNGS | HBVCgp3_X//upstream | MIR4454(dist=392007),NAP1L3(dist=454023)//intergenic | 15 | chrX | 92471909 | + | AY123041 | 303 | - | Xq21.32 |
| 25T | 20C211264_R303_CapNGS | HBVCgp4_PREC/C | PCCA//intronic | 17 | chr13 | 100817422 | + | AY123041 | 1881 | + | 13q32.3 |
| 25T | 20C211264_R303_CapNGS | HBVCgp3_X//upstream | NALT1(dist=20486),LINC01451(dist=41798)//intergenic | 23 | chr9 | 139464682 | + | AY123041 | 655 | + | 9q34.3 |
| 25T | 20C211264_R303_CapNGS | NONE(dist=NONE),HBVCgp3_X(dist=1009)//intergenic | CADM1(dist=45363),LOC101928985(dist=182167)//intergenic | 30 | chr11 | 115420496 | + | AY123041 | 239 | + | 11q23.3 |
| 25T | 20C211264_R303_CapNGS | HBVCgp3_X//upstream | NONE(dist=NONE),ANKRD26P1(dist=104532)//intergenic | 1000 | chr16 | 46398717 | + | AY123041 | 688 | + | 16q11.2 |
| 25T | 20C211264_R303_CapNGS | HBVCgp3_X//upstream | NONE(dist=NONE),ANKRD26P1(dist=110346)//intergenic | 1445 | chr16 | 46392903 | + | AY123041 | 688 | + | 16q11.2 |
| 25T | 20C211264_R303_CapNGS | NONE(dist=NONE),HBVCgp3_X(dist=1118)//intergenic | FRK//intronic | 5938 | chr6 | 116346702 | - | AY123041 | 130 | + | 6q22.1 |

| | | | | | | | | | | | |
|---|---|---|---|---|---|---|---|---|---|---|---|
| 25T | 20C211264_R303_CapNGS | NONE(dist=NONE),HBVCgp3_X(dist=1118)//intergenic | FRK//intronic | 5939 | chr6 | 116346700 | - | AY123041 | 130 | + | 6q22.1 |
| 25T | 20C211264_R303_CapNGS | NONE(dist=NONE),HBVCgp3_X(dist=1118)//intergenic | FRK//intronic | 5939 | chr6 | 116346701 | - | AY123041 | 130 | + | 6q22.1 |
| 25T | 20C211264_R303_CapNGS | HBVCgp3_X | TERT//upstream | 5978 | chr5 | 1295327 | - | AY123041 | 1674 | + | 5p15.33 |
| 25T | 20C211264_R303_CapNGS | NONE(dist=NONE),HBVCgp3_X(dist=1118)//intergenic | FRK//intronic | 6539 | chr6 | 116346699 | + | AY123041 | 130 | - | 6q22.1 |
| 25T | 20C211264_R303_CapNGS | HBVCgp3_X | FRK//intronic | 6762 | chr6 | 116347208 | + | AY123041 | 1678 | - | 6q22.1 |
| 25T | 20C211264_R303_CapNGS | HBVCgp3_X | TERT//upstream | 6777 | chr5 | 1295331 | - | AY123041 | 1674 | + | 5p15.33 |
| 25T | 20C211264_R303_CapNGS | HBVCgp3_X | FRK//intronic | 7499 | chr6 | 116347204 | + | AY123041 | 1678 | - | 6q22.1 |
| 25T | 20C211264_R303_CapNGS | HBVCgp4_PREC/C | DNAH9//intronic | 9290 | chr17 | 11747593 | + | AY123041 | 1892 | + | 17p12 |
| 25T | 20C211264_R303_CapNGS | HBVCgp4_PREC/C | DNAH9//intronic | 10477 | chr17 | 11747598 | + | AY123041 | 1892 | + | 17p12 |
| 26T | 20C211265_R303_CapNGS | NONE(dist=NONE),HBVCgp3_X(dist=1246)//intergenic | HNF4G(dist=15128),LINC01111(dist=824700)//intergenic | 5 | chr8 | 76494189 | - | AY123041 | 2 | + | 8q21.11 |
| 26T | 20C211265_R303_CapNGS | HBVCgp2_PRES1/PRES2/S;HBVCgp1_P;HBVCgp3_X;HBVCgp4_PREC/C//upstream;downstream | CD83(dist=73302),LINC01108(dist=69908)//intergenic | 5 | chr6 | 14210450 | - | AY123041 | 2397 | + | 6p23 |
| 26T | 20C211265_R303_CapNGS | HBVCgp4_PREC/C | LHFPL2//intronic | 5 | chr5 | 77804856 | - | AY123041 | 1755 | + | 5q14.1 |
| 26T | 20C211265_R303_CapNGS | HBVCgp3_X//upstream | ARRDC3-AS1(dist=121288),NR2F1-AS1(dist=1907242)//intergenic | 5 | chr5 | 90837820 | + | AY123041 | 260 | + | 5q14.3 |
| 26T | 20C211265_R303_CapNGS | HBVCgp2_PRES1/PRES2/S;HBVCgp1_P;HBVCgp3_X;HBVCgp4_PREC/C//upstream;downstream | TNIK//intronic | 5 | chr3 | 170796038 | + | AY123041 | 2419 | + | 3q26.2 |
| 26T | 20C211265_R303_CapNGS | HBVCgp3_X;HBVCgp4_PREC/C//upstream | GULP1//intronic | 5 | chr2 | 189166166 | + | AY123041 | 696 | - | 2q32.1 |
| 26T | 20C211265_R303_CapNGS | NONE(dist=NONE),HBVCgp3_X(dist=1198)//intergenic | CMC2//intronic | 5 | chr16 | 81011790 | + | AY123041 | 50 | + | 16q23.2 |
| 26T | 20C211265_R303_CapNGS | NONE(dist=NONE),HBVCgp3_X(dist=1188)//intergenic | LOC105369879(dist=113695),LINC02258(dist=322796)//intergenic | 5 | chr12 | 87839793 | - | AY123041 | 60 | + | 12q21.32 |
| 26T | 20C211265_R303_CapNGS | HBVCgp4_PREC/C | NEDD9//intronic | 6 | chr6 | 11302984 | + | AY123041 | 2311 | - | 6p24.2 |
| 26T | 20C211265_R303_CapNGS | NONE(dist=NONE),HBVCgp3_X(dist=1159)//intergenic | LINC01935(dist=1145437),LOC100287010(dist=248984)//intergenic | 6 | chr2 | 104746324 | + | AY123041 | 89 | - | 2q12.1 |
| 26T | 20C211265_R303_CapNGS | HBVCgp4_PREC/C | HOXD1(dist=51533),MTX2(dist=27002)//intergenic | 6 | chr2 | 177107168 | + | AY123041 | 2225 | + | 2q31.1 |
| 26T | 20C211265_R303_CapNGS | HBVCgp3_X | ZNF274//intronic | 6 | chr19 | 58713011 | - | AY123041 | 1521 | + | 19q13.43 |
| 26T | 20C211265_R303_CapNGS | HBVCgp4_PREC/C | MGAT4C(dist=190255),LOC105369879(dist=301484)//intergenic | 6 | chr12 | 87423032 | + | AY123041 | 1969 | + | 12q21.32 |
| 26T | 20C211265_R303_CapNGS | HBVCgp3_X;HBVCgp4_PREC/C//upstream | SOX1-OT//ncRNA_intronic | 7 | chr13 | 112717432 | + | AY123041 | 706 | - | 13q34 |
| 26T | 20C211265_R303_CapNGS | HBVCgp4_PREC/C | MED10(dist=66951),UBE2QL1(dist=3361)//intergenic | 8 | chr5 | 6445611 | - | AY123041 | 1735 | + | 5p15.31 |

| | | | | | | | | | | | |
|---|---|---|---|---|---|---|---|---|---|---|---|
| 26T | 20C211265_R303_CapNGS | HBVCgp3_X//upstream | ITGB5//intronic | 8 | chr3 | 124556887 | - | AY123041 | 385 | + | 3q21.2 |
| 26T | 20C211265_R303_CapNGS | HBVCgp3_X//upstream | DYRK4//intronic | 8 | chr12 | 4693279 | - | AY123041 | 610 | + | 12p13.32 |
| 26T | 20C211265_R303_CapNGS | HBVCgp3_X;HBVCgp4_PREC/C//upstream | EEF1G//intronic | 8 | chr11 | 62333943 | + | AY123041 | 743 | - | 11q12.3 |
| 26T | 20C211265_R303_CapNGS | HBVCgp4_PREC/C | LINC02578//ncRNA_intronic | 9 | chr9 | 121472487 | + | AY123041 | 2203 | + | 9q33.1 |
| 26T | 20C211265_R303_CapNGS | NONE(dist=NONE),HBVCgp3_X(dist=1137)//intergenic | FCHO2//intronic | 9 | chr5 | 72366649 | + | AY123041 | 111 | + | 5q13.2 |
| 26T | 20C211265_R303_CapNGS | HBVCgp4_PREC/C | BLOC1S4(dist=27819),KIAA0232(dist=37246)//intergenic | 9 | chr4 | 6747208 | + | AY123041 | 1835 | + | 4p16.1 |
| 26T | 20C211265_R303_CapNGS | HBVCgp4_PREC/C//downstream | PRKCI//intronic | 9 | chr3 | 170016701 | + | AY123041 | 3041 | - | 3q26.2 |
| 26T | 20C211265_R303_CapNGS | HBVCgp3_X | ANKRA2//intronic | 10 | chr5 | 72856275 | + | AY123041 | 1673 | - | 5q13.2 |
| 26T | 20C211265_R303_CapNGS | HBVCgp4_PREC/C | LINC00290(dist=53161),LINC02500(dist=48706)//intergenic | 10 | chr4 | 182133463 | + | AY123041 | 1816 | - | 4q34.3 |
| 26T | 20C211265_R303_CapNGS | HBVCgp4_PREC/C | EFCC1//intronic | 10 | chr3 | 128723989 | + | AY123041 | 1974 | - | 3q21.3 |
| 26T | 20C211265_R303_CapNGS | NONE(dist=NONE),HBVCgp3_X(dist=1054)//intergenic | PHOSPHO2-KLHL23(dist=7481),SSB(dist=39512)//intergenic | 10 | chr2 | 170615869 | + | AY123041 | 194 | + | 2q31.1 |
| 26T | 20C211265_R303_CapNGS | HBVCgp3_X//upstream | SETBP1(dist=80925),SLC14A2(dist=18528)//intergenic | 10 | chr18 | 42729400 | + | AY123041 | 338 | - | 18q12.3 |
| 26T | 20C211265_R303_CapNGS | HBVCgp3_X;HBVCgp4_PREC/C//upstream | CADPS2//intronic | 12 | chr7 | 122150502 | + | AY123041 | 754 | - | 7q31.32 |
| 26T | 20C211265_R303_CapNGS | NONE(dist=NONE),HBVCgp3_X(dist=1056)//intergenic | NDRG2//intronic | 12 | chr14 | 21534428 | - | AY123041 | 192 | + | 14q11.2 |
| 26T | 20C211265_R303_CapNGS | HBVCgp4_PREC/C | TMEM67//intronic | 13 | chr8 | 94819703 | + | AY123041 | 1787 | + | 8q22.1 |
| 26T | 20C211265_R303_CapNGS | NONE(dist=NONE),HBVCgp3_X(dist=1046)//intergenic | LOC389895(dist=310386),SOX3(dist=99696)//intergenic | 17 | chrX | 139485456 | + | AY123041 | 202 | + | Xq27.1 |
| 26T | 20C211265_R303_CapNGS | HBVCgp4_PREC/C | ZNF804B(dist=245305),STEAP2-AS1(dist=298520)//intergenic | 17 | chr7 | 89213147 | + | AY123041 | 1805 | - | 7q21.13 |
| 26T | 20C211265_R303_CapNGS | HBVCgp4_PREC/C | CRISP1//intronic | 18 | chr6 | 49808341 | - | AY123041 | 1921 | + | 6p12.3 |
| 26T | 20C211265_R303_CapNGS | HBVCgp3_X;HBVCgp4_PREC/C//upstream | CTNND2//intronic | 18 | chr5 | 11381627 | + | AY123041 | 735 | + | 5p15.2 |
| 26T | 20C211265_R303_CapNGS | HBVCgp4_PREC/C | RBL1//intronic | 22 | chr20 | 35693484 | + | AY123041 | 2027 | - | 20q11.23 |
| 26T | 20C211265_R303_CapNGS | HBVCgp3_X;HBVCgp4_PREC/C//upstream | WWTR1//intronic | 44 | chr3 | 149291921 | + | AY123041 | 1161 | + | 3q25.1 |
| 26T | 20C211265_R303_CapNGS | HBVCgp4_PREC/C | ERN1//intronic | 49 | chr17 | 62169283 | - | AY123041 | 2305 | + | 17q23.3 |
| 26T | 20C211265_R303_CapNGS | HBVCgp3_X;HBVCgp4_PREC/C//upstream | RHOJ//intronic | 54 | chr14 | 63720832 | + | AY123041 | 702 | + | 14q23.2 |
| 26T | 20C211265_R303_CapNGS | HBVCgp3_X;HBVCgp4_PREC/C//upstream | NBEA//intronic | 59 | chr13 | 36209806 | + | AY123041 | 708 | + | 13q13.3 |
| 26T | 20C211265_R303_CapNGS | HBVCgp3_X//upstream | KCTD16(dist=674637),PRELID2(dist=596021)//intergenic | 85 | chr5 | 144539886 | + | AY123041 | 512 | - | 5q32 |
| 26T | 20C211265_R303_CapNGS | HBVCgp4_PREC/C | FAM120B//intronic | 92 | chr6 | 170707584 | + | AY123041 | 2011 | - | 6q27 |
| 26T | 20C211265_R303_CapNGS | HBVCgp3_X//upstream | SND1//intronic | 94 | chr7 | 127477172 | + | AY123041 | 522 | + | 7q32.1 |
| 26T | 20C211265_R303_CapNGS | HBVCgp3_X//upstream | TRPM6//intronic | 103 | chr9 | 77385433 | + | AY123041 | 257 | + | 9q21.13 |
| 26T | 20C211265_R303_CapNGS | HBVCgp3_X//upstream | EMBP1(dist=171747),NONE(dist=NONE)//intergenic | 106 | chr1 | 121485433 | + | AY123041 | 260 | + | 1p11.2 |

| | | | | | | | | | | | |
|---|---|---|---|---|---|---|---|---|---|---|---|
| 26T | 20C211265_R303_CapNGS | HBVCgp2_PRES1/PRES2/S;HBVCgp1_P;HBVCgp3_X;HBVCgp4_PREC/C//upstream;downstream | ZNF804A//intronic | 109 | chr2 | 185738535 | + | AY123041 | 2327 | + | 2q32.1 |
| 26T | 20C211265_R303_CapNGS | HBVCgp3_X;HBVCgp4_PREC/C//upstream | DMXL2//intronic | 112 | chr15 | 51899979 | + | AY123041 | 694 | - | 15q21.2 |
| 26T | 20C211265_R303_CapNGS | HBVCgp4_PREC/C | LRRC63//intronic | 134 | chr13 | 46804962 | + | AY123041 | 2003 | + | 13q14.13 |
| 26T | 20C211265_R303_CapNGS | HBVCgp3_X//upstream | LOC102724084//ncRNA_intronic | 142 | chr16 | 80553781 | - | AY123041 | 519 | + | 16q23.2 |
| 26T | 20C211265_R303_CapNGS | HBVCgp3_X;HBVCgp4_PREC/C//upstream | DPP10//intronic | 159 | chr2 | 115399936 | + | AY123041 | 753 | - | 2q14.1 |
| 26T | 20C211265_R303_CapNGS | HBVCgp4_PREC/C | KIF1A//intronic | 174 | chr2 | 241726822 | + | AY123041 | 2145 | + | 2q37.3 |
| 26T | 20C211265_R303_CapNGS | HBVCgp4_PREC/C//downstream | MIR3668(dist=144754),MIR4465(dist=333734)//intergenic | 222 | chr6 | 140671217 | + | AY123041 | 2994 | - | 6q24.1 |
| 26T | 20C211265_R303_CapNGS | NONE(dist=NONE),HBVCgp3_X(dist=1159)//intergenic | NALCN//intronic | 224 | chr13 | 101886697 | + | AY123041 | 89 | + | 13q33.1 |
| 26T | 20C211265_R303_CapNGS | NONE(dist=NONE),HBVCgp3_X(dist=1122)//intergenic | LINC02712(dist=290982),LINC02098(dist=580751)//intergenic | 294 | chr11 | 127497910 | + | AY123041 | 126 | - | 11q24.2 |
| 26T | 20C211265_R303_CapNGS | HBVCgp4_PREC/C | OPCML//intronic | 306 | chr11 | 133063737 | + | AY123041 | 1715 | - | 11q25 |
| 26T | 20C211265_R303_CapNGS | NONE(dist=NONE),HBVCgp3_X(dist=1143)//intergenic | WASH8P(dist=4186),IQSEC3(dist=80484)//intergenic | 311 | chr12 | 95455 | + | AY123041 | 105 | + | 12p13.33 |
| 26T | 20C211265_R303_CapNGS | HBVCgp4_PREC/C | ZNF268//intronic | 1753 | chr12 | 133766589 | + | AY123041 | 1806 | + | 12q24.33 |
| 26T | 20C211265_R303_CapNGS | NONE(dist=NONE),HBVCgp3_X(dist=1037)//intergenic | EIF3L//UTR5 | 3884 | chr22 | 38245442 | + | AY123041 | 211 | + | 22q13.1 |
| 27T | 20C211266_R303_CapNGS | HBVCgp4_PREC/C | POTEA(dist=323391),NONE(dist=NONE)//intergenic | 5 | chr8 | 43541719 | + | AY123041 | 2268 | - | 8p11.1 |
| 27T | 20C211266_R303_CapNGS | HBVCgp4_PREC/C | LINC01935(dist=675816),LOC100287010(dist=718605)//intergenic | 5 | chr2 | 104276703 | - | AY123041 | 2010 | + | 2q12.1 |
| 27T | 20C211266_R303_CapNGS | HBVCgp2_PRES1/PRES2/S;HBVCgp3_X;HBVCgp4_PREC/C//upstream;downstream | LUZP6(dist=599923),CHRM2(dist=291369)//intergenic | 6 | chr7 | 136262030 | + | AY123041 | 2630 | - | 7q33 |
| 27T | 20C211266_R303_CapNGS | HBVCgp3_X//upstream | IKBKE//exonic//IKBKE:NM_001193321:exon4:c.30,IKBKE:NM_001193322:exon5:c.285,IKBKE:NM_014002:exon5:c.285 | 6 | chr1 | 206648264 | + | AY123041 | 653 | - | 1q32.1 |
| 27T | 20C211266_R303_CapNGS | HBVCgp3_X//upstream | RTN4RL2(dist=3741),SLC43A1(dist=3259)//intergenic | 7 | chr11 | 57248748 | + | AY123041 | 450 | - | 11q12.1 |
| 27T | 20C211266_R303_CapNGS | HBVCgp3_X;HBVCgp4_PREC/C//upstream | FBXO15//intronic | 8 | chr18 | 71806441 | + | AY123041 | 878 | + | 18q22.3 |
| 27T | 20C211266_R303_CapNGS | HBVCgp3_X//upstream | LHFPL2//intronic | 10 | chr5 | 77859343 | - | AY123041 | 515 | + | 5q14.1 |
| 27T | 20C211266_R303_CapNGS | HBVCgp3_X | MIR548XHG(dist=922944),LINC01683(dist=210519)//intergenic | 13 | chr21 | 21055074 | - | AY123041 | 1457 | + | 21q21.1 |
| 27T | 20C211266_R303_CapNGS | NONE(dist=NONE),HBVCgp3_X(dist=1129)//intergenic | ETV6//intronic | 18 | chr12 | 11939063 | + | AY123041 | 119 | - | 12p13.2 |
| 27T | 20C211266_R303_CapNGS | HBVCgp3_X//upstream | NBPF25P//ncRNA_intronic | 21 | chr1 | 148548189 | + | AY123041 | 346 | - | 1q21.2 |

| | | | | | | | | | | | |
|---|---|---|---|---|---|---|---|---|---|---|---|
| 27T | 20C211266_R303_CapNGS | HBVCgp2_PRES1/PRES2/S;HBVCgp3_X;HBVCgp4_PREC/C//upstream;downstream | CLEC4GP1(dist=18900),EVI5L(dist=20306)//intergenic | 22 | chr19 | 7874798 | + | AY123041 | 2625 | + | 19p13.2 |
| 27T | 20C211266_R303_CapNGS | HBVCgp2_PRES1/PRES2/S;HBVCgp3_X;HBVCgp4_PREC/C//upstream;downstream | BUB1B-PAK6;BUB1B//intronic | 22 | chr15 | 40512289 | + | AY123041 | 2520 | - | 15q15.1 |
| 27T | 20C211266_R303_CapNGS | HBVCgp2_PRES1/PRES2/S;HBVCgp1_P;HBVCgp3_X;HBVCgp4_PREC/C//upstream;downstream | C19orf18//intronic | 27 | chr19 | 58472107 | + | AY123041 | 2328 | + | 19q13.43 |
| 27T | 20C211266_R303_CapNGS | NONE(dist=NONE),HBVCgp3_X(dist=1233)//intergenic | C7orf65(dist=73590),LINC00525(dist=26238)//intergenic | 33 | chr7 | 47774836 | + | AY123041 | 15 | + | 7p12.3 |
| 27T | 20C211266_R303_CapNGS | HBVCgp2_PRES1/PRES2/S;HBVCgp1_P;HBVCgp3_X;HBVCgp4_PREC/C//upstream;downstream | LINC02241(dist=263690),GUSBP1(dist=258097)//intergenic | 37 | chr5 | 21201492 | + | AY123041 | 2412 | + | 5p14.3 |
| 27T | 20C211266_R303_CapNGS | HBVCgp3_X//upstream | EIF2S2(dist=120272),ASIP(dist=27850)//intergenic | 47 | chr20 | 32820321 | + | AY123041 | 314 | - | 20q11.22 |
| 27T | 20C211266_R303_CapNGS | HBVCgp4_PREC/C | LINC01934//ncRNA_intronic | 59 | chr2 | 182140585 | + | AY123041 | 2238 | + | 2q31.3 |
| 27T | 20C211266_R303_CapNGS | HBVCgp4_PREC/C | WASH8P(dist=4169),IQSEC3(dist=80501)//intergenic | 432 | chr12 | 95432 | + | AY123041 | 2238 | - | 12p13.33 |
| 27T | 20C211266_R303_CapNGS | HBVCgp4_PREC/C | WASH8P(dist=4185),IQSEC3(dist=80485)//intergenic | 481 | chr12 | 95448 | + | AY123041 | 2242 | - | 12p13.33 |
| 27T | 20C211266_R303_CapNGS | HBVCgp3_X//upstream | CDH2(dist=365382),MIR302F(dist=1756400)//intergenic | 12678 | chr18 | 26122476 | + | AY123041 | 548 | - | 18q12.1 |
| 27T | 20C211266_R303_CapNGS | HBVCgp3_X//upstream | CDH2(dist=365381),MIR302F(dist=1756401)//intergenic | 12768 | chr18 | 26122475 | - | AY123041 | 550 | + | 18q12.1 |
| 27T | 20C211266_R303_CapNGS | HBVCgp3_X//upstream | CDH2(dist=365380),MIR302F(dist=1756402)//intergenic | 12768 | chr18 | 26122474 | - | AY123041 | 550 | + | 18q12.1 |
| 27T | 20C211266_R303_CapNGS | HBVCgp4_PREC/C | CDH2(dist=365428),MIR302F(dist=1756354)//intergenic | 12808 | chr18 | 26122522 | - | AY123041 | 1778 | + | 18q12.1 |
| 27T | 20C211266_R303_CapNGS | HBVCgp4_PREC/C | CDH2(dist=365427),MIR302F(dist=1756355)//intergenic | 12808 | chr18 | 26122521 | - | AY123041 | 1778 | + | 18q12.1 |
| 27T | 20C211266_R303_CapNGS | HBVCgp4_PREC/C | CDH2(dist=365431),MIR302F(dist=1756351)//intergenic | 12809 | chr18 | 26122525 | - | AY123041 | 1778 | + | 18q12.1 |
| 27T | 20C211266_R303_CapNGS | HBVCgp4_PREC/C | CDH2(dist=365426),MIR302F(dist=1756356)//intergenic | 13153 | chr18 | 26122520 | + | AY123041 | 1778 | - | 18q12.1 |

Supplementary Table 7 Complete structure of HBV integration

| ID | Left-Chr | Left-breakpoint(start-end) | Right-Chr | Right-Breakpoint(start-end） | Annotation | HBV genome | Start | End | HBV genome | Start | End | HBV genome | Start | End | HBV genome | Start | End |
|---|---|---|---|---|---|---|---|---|---|---|---|---|---|---|---|---|---|
| A | chr5+ | 1289182-1291798 | chr5- | 1295506-1294471 | TERT | HBV- | 1801 | 52 | HBV- | 3182 | 2234 | HBV+ | 479 | 1825 | | | |
| B | chr17- | 11661235-11660600 | chr17+ | 11660600-11662081 | DNAH9 | HBV- | 1797 | 4 | HBV- | 3181 | 1957 | HBV+ | 2951 | 3182 | HBV+ | 63 | 1797 |

Supplementary Table 8 Data source

**Read ID A Sequence:**

ATGTACTTCGTTCAGTTACGTATTGCTTGGTACAGCGGCGGCCCGCACCTGGTAGGCGCAGCTG
GGGGCCACCAGCACAAGAGCGCGCAGCGTACCAGCGAGGTGACCAGCGTCGTCGCCCACGCG
GCGCAGCGGCAGCCCCCGCCCCGCTTTCCCCCGCAGTGTCACCGGTCACCGTATTGGGCGAAG
GTAGCTGCGCACGCTGGTGGTGAAGGCCTCGAGGGGCCCCCGCGAGTCCAATCCAGCAGCGC
GCGAAGCCGAAGGCCAGCACGTTCTTCGCTGCGCTGCACAGCCTCTGCAGCACTCGGGCCACC
AGCTCCCTTCCAGGCAGGACACCTGCGGAAGCGCCTGAGTCACTGCGCTGCTCTCCATGTCGCT
GGTTCCCCCCGGCCGCCCTCAACCCCAGCCGGACGGCGACCCCGGGAGGCCACCTGGCGGAA
GGAGGGCGGCGGGGGCGACGTGCATCCCAGGGCACACCAGGCACTGGGCCACCAGCGCGCG
GAAAGCCGCCGGGTCCGCGCTGCACCAGCCGCCAGCCCTGGGGCCCCAGGCGCCGCACGAG
GCGTGGCCAGCGGCAGCACCTCGCGGTAGTGGCTGCGCAGCAAGGAGCTTACGGCTCAGCAG
CGGGAGCGCGCGGCATCGCGGGGTGGCCGGGAGCCAGGGCTTCCCACGTGCGCAGCAGGAC
GCAGCGCTGCCTGAAACTCGCGCCGCGAGGAGGCGGGCCGCAGAAGGAAGGGGAGGGGCTG
GGAGGGCCCGGAGGCTGGGCCGGGGACCCGGGAGGGTCGGGACAGGGCGGGGTCCGCGCG
GAGGAGCGGAGCTGGAAGGTGAAGGAGCGGACGGGTGCCCGGGTCCCAGTCCCTCCGCCACG
TGGGGAGCGCGGTCCACGGAAAAATCTGTGCCCGCAGATCCACTGGGGCCGGCCTGGCCGAC
AGCGCAGCTGCTCCGGGCGGACCCGAAGTCGGGCGCGCTTCCCCGCCCGCGCGCCGCTCGCT
CCCAGGGTGCAGGACATGGCGAGGGGCCCCAAAGTTGCATAGTGCTGGTGAACAGACCATTTA
TGCCTACAGCCTCCTGGTACCAAAGATCGTGCCTGAGTCTCCTCCCCAACTCCTCCCAGTCCTTA
AACAAACAGTCTTTGAGTATGCCTCAAGGTCGGTCGTTGACATTGCTGCGAGTCCAAGAGTCCT
CTTATGTAAGACCTTGGGCAAGACCTGGTGGGCGTTCCCTGGTGGTCGCCATGCGACGTGCAG
AGGTGAAGCGAAGTGCACGGTCCGGCGAATGAGAAGGCACAGACGGGGAGACCCCGCGTAG
AAAGGAGTGCGCCCCGTGGTCGGCCCGAGACGGCAGATGAAGAAGGGGACGAAGTGAAGCC
CCAAACGGCCCCCGAGACGGGTCGTCCGCAGGATTCAGCGCCGACGGGACGTAGACATAAGA
CGTTCCGCGCAGGATCCAGTTGGCAGCACACCCTGGCAACCATGGAAAGGGTGTATTTCGAGA
GAGGACAACAGGTTGTCGGTTCCGATAGTTTCGCTCCAGACCGGCTGCGAGCAAAACAAGCTG
CTAGGAGTTCCGCAGTATGGATCGGTAGAGGAGCCACAAGGTTCCACGCATGCGGCGATGGCC
AATAGCCAAGCCCATCCAGTGGGGGTTGCGTCAGCTTTTAAACACTTGGCAGAGACCTGACCGT
TGCCGGGCAACGGGTAAGGTTCAGATGGCCCCGACACACAGAAAGGCCTTGTAAGTTGGCGAG
AAAGTGAAAGCCTGCTTGGGTGTATACATGCATATAAAGGCATTAAGGCAGGATAGCCACATTG
TGTAAAAGGGGCAGCAAAGCCCAGAAGACCCACAATTCTCGACATACTTTCCAATCAATAGGTC
TATTTACAGGCAGTTTTCGAAAACATTGCTTGATTTTTAGTACAATATGTTCTTGCGGTAAGTACC
CCAACATCCAATTACATATCCCATGGTAAAGGGAGTAGCCCCAACGTTTGGTTTTATTAGGGTTC
AAATGTATGCAAGACAAAAGAAAATTGGTAATAGAGGTAAAGGGACTCAAGATGTTGTACAGA
CTTGGCCCCCAATACCCTCATCCATATAACTGAAAGCCAGACAGTGGGGGGAAAGCCCTACGA
ACCACTGAACAAATGGCACTATTAAACTGAGCCAGGAGAAACGGACTGAGGCCCACTCCCATA
GGAATCTTGCAGCCAGGATGATGGGATGGGAATACAAGTGCAGTTTCAAAGTCGAAGGTTTTGT
ACAGCAACAAGAGGGAAACATAGAGGTTCCTTGAGCAGGAGTCGTGCAGGTCTTGCATGGTCC
CGTGCTGGTAGTTGATGTTCCTGGAAGAAGAGAAACTGTTCTTGAGTATTTGGTGTCTTTTGGAG
TGTGGATTCGCGCTCCTCCCTGCTTACAGACCACAAAAATGCTATCTTATCAACACTTCCGGAAA
CTACTGTTGAACGACGATGCAGGTCCCCTAGAAGGAGGGAGCTCCCTCGCCGCCTCGCAGACG
AGGTCTCAATGCCGCGTCGCAGAAGATCTCACTCTCGGGAATCTTAATGTTAGTATCCCTTGGA

CTCATAAGGTGGGAAACTTTTCTGGGCTTTGTTAACTACCGTACCTGTCTTTACAATCTGAGTGG
CAAACTCCCTCCTTTCCTCATTCATTTACGGAGGATTATTGTTAATAGTCGCAATATGTGGGCCCT
CTTACAGTTAATGAAAAAAATTAAAATTAATTATGCCTGTAGAGTTTCTATCTAACCTTACCAAAT
ATCTAGGCATTAAGCCATATTATCCTGAACATGCAGTTAATCATTACTTCAAAACTAGGCATTATT
TACATACTCTGTGGAAGGCGGGCATTCCTATATAAGAGAAACTACACACTGTGCCTTACCTCATT
TTGTTGGGTCCATATATTCTTGGGAACAAGAGCTACAGCATGGGAGGGTTGGTCTTCCAGACGA
CCTCGAAAGGCATGGGACAGATCTTTCTGTTCCCAATCCTCTAGGGATTCTTTCCCGATCACCAC
AGTTGGACCTGCGTTCCGGAGCCAACTCAAACAATCCAATTGGGACACAGCCTAGCAAGGATC
ACTGGCCAGAGGCAATCAGGTAGAACCCCAGGGAGCATTCGGGCCAGGGTTCACCCCACCAC
GGCAGTCTTTTGGGGTGGAGCCCTCAGGCTCAGGGCATATTGACAACAGTGCCAGTAGCACCT
CCTCCTGCCTCCACCAATCGGCAGTCAGAGACAGCCTACTCCCATCTCCTCTAAAAAAGAGACA
GTCATCCTCAGGCCATGCAGTGGGAACTCCACAACTCACCAAATACTTCCTGCTGGAGGTGGCT
CCAGTTCCCGGAACAATAGAAGCCACAATTCCGACTACTCTACACCATATCGTCCAATCTTCTCT
GAGGGACTGGGGACCCTGCAGGCACATGGAGAACACAACATCAGAGTTCCTAGGACCCTGCTC
GGGTTACAGGCGTTTCTTGAGTACGACAGAATCCTCACAATACCACAGAGTCTAGACTCATTAA
TTGGACTTCTCTAATTTCTAGGGAGCACCGCGTGTCTAGCCGTCCGCGGTCCCAACCTCAATCAT
ACTTAACCTCATGCATCCTCCAATTTGTCCTGGCTATCGCTGGATGTGTCTGCGGCACGTTTTATC
ATATTCCTCTTCATCCACTGCTATGCCTCATGTCTTCTTGTTGGTTCTTCTGGACACCAGGTATGTT
GCCCGTTTGTCCTGCAACAGGAACATCAACTACCAGCTGGGACCATGCAAAGCCTGCGACTCCT
GCTCAAAGGAGGAACACCTCTATGTTTCCCTCTTGTTGCTGTACCCAAACCTTCGGACAGGAAA
CTGCACTTGTATTCCCATCCCATCATCCTGGAGCTGCATAAGATTCTATGGGAATTGGGCCTCAG
TCCATTTCTCCTGGCTCAGTTTACTAGTGCCATTTGTTCAGTGGTTCGTAGACTTTCCCACTGTCT
GCCTTTCAGTTATATGTGGATGATGTGGTATTGGGGGCCAAGTCTGTACAACATCTTTAGGAGTC
CCTTTTTGCCTCTATTACCAATTTTCTTTTGTCTTTTGGAGTATACATTGAACCCTAATAAAACCAA
CGTTGGGGCTACTCCCTTAATTTCATGGGATATGTAATTGGATGTTGGGGTACTTTACCATAGAA
TATTGTATAAATCAACAATGTTTTTCTGAAAACTGCCTGTAAATGAACCTATTGATTGGAAAGTAT
ATCAGAGAATTGTGGGTCTTCTAGGCTTTTGCTGCCTTTTACACAATGTGGCTATCCTTAATGCCT
TTATATGCATGTATACAATCTAAGCAGGCTTTCACTTTCTCGCCAACTTACAAGGCCTTTCTGTGT
AAAAAGCTTAAGCTAATACGAACCCTTTACCCCGTTGCCCGGCAACGATGAGTCTATAAGTGTT
TTGACGCAACCCCACTGGATGGGGCTTGGCTATTGGCCATCGCCACATGCGTGGAACTTGTGGC
TCCTCTACCACCCTGCCGTCCCAGCACTGCGGAACTCCCTAGCAGCTTGTTTGCTCTTGGCAGTC
TGGGCAAAACTTATCAGGATCAACTAACTCTGTTTGTCCTCTCTCCAAGCTCACCTCCTTTCCATG
GCTGCTGGGGTGTGCTGCCAACTGGATCCTGCGCGGAACGTCCTTTGTCTACGTCCCGTCGGCG
CTTCCCAGATCACCGGGGCTTGACCGTCTCGGGGCCGTTGAGACTTCGTCCCCTTCTTCATCTGC
CGTTCCAGCCACGTCCACAGCGCCTCTTCACGCGGTCTCCCCGTCTGGCGCCTTCTCATCTGCC
GGACCGTGTGCGCTTCCTTCACCTCTGCGTCGCATGGCGACCACCGTGAACGCCCACCAGGTCT
TGCCCAAGGTCTTGTAAGAGGACTCTTGGATAACAACGGCAATGTCAACGACCGACCTTGAGA
ACTACTTCAAAGACTGTTTGTTTAAGGACTGGGAGGAGTTGGGGAGGGCTAGATTGGCCTAGTC
TTTGTACTAAGAGCTGTAGGCATAAATTGGTCGTCCCGGGTGTCCCTGTCCCGTGCAGAATTGA
GTGAGGCGCACGCCGTCCCGAGTGTCCCTGTCCCGTGCAGAAAATTCAGGCAGGCACTTATCC
GGGTGTCCCTGTCACGTGCAGGGTGAGTGAGGCGCAGGTCCGAGGTGTCCCTGTCACATTGCA
GGGTGAGTGAGGCATCCCAGGTGTCCTGTCACGTGCAGGGTGAGTGAGAGCACGGCCCCCCC
CCAGGTGTCTGTACGTGCAGGGTGGTGAGGGCGCGGCCCCGGGTGTCCCTGTCACGTGCAGGG
TGGTGAGCTTGATCCCCCGGGTGTCCCTGTCGTGCAGGGTGGTGAGGCGCCGTCCCCGAATTTC

CCTGTCCCGTGCAGGGTGGGGGGTGAGGCATGATCCCCGGGTGTCTCTGTCACCGTGCAGGGT
GGGTGGTGCTGCTCCCCACGAAGGTATTATGCGATGCAGGGTGGTGAGGCACGAAGCCCCTGG
GTGTCCTGTCACGTGCAGGGTGAGTGAGGCGCCGTCCCGGGTGTCCTGTCCGTGCAGGGTGAG
TGAGCGCCGTCCCCGGGTGTCTCTGTCACGTGCACGAGGGTGGTGAGGCGCAGAAATCCCCAG
GTGTCCCTGTCACGTGCAGGGTGGTGAGGCAGCCTGGGTGTCCCTGTCACGTGCAGGGTGAGT
GAGGCGCGGCCCCAGGTGTCTGTCACGTGCCGGGGGTGGTGAGCGCAGCCCCGGGTGTCCTG
TCGCATTCAGGTGGTGAGGCGCGGCCCCCGGTGTCCTGTCACGTGCAGGGTGAGTGAGGCGCG
GTCCCCGGAATTTGTCCCTGTCACGTGCAGGGTGAGTGAGCGCGGTCCCCCGGGTGGCTCCTGT
CACGTGCAGGGTGAGTGAGGCGCGGTCCCCAGGTGTCCCTGTCCGTGTGAGATTGGTGAGGCA
CTGTCTGGGTGTCTGTCACGTTCCGGGGTGAGTGAGGCGCGGTCCCCGGGTGTCCCTGTCACGT
GTGAGGTGAGTGAGGCACTGTCCCCCGGGTGTCCCTGTACGTGCAGGGTGGAGTGAGGCGCCG
TCCCCCGGGTGTCCCTGTCACGTGCAGGGTGATTGACGCGAAAGAGGCCCCGGGTGTCCTGTC
ACACGTGCAGGGTGAGTGAGGCATAGTCCCCGCCGTGGTCCTGTCACGTGCAGGGTGAGTGGG
GGCGCAAATCGTGTCCCTGTCACGTGTAGGGTGGTGAGGTGCCGTCCCAGGTCTATGCACGTGT
AGGGTGAGTGACTGCGCCAAATCCCCAGGTGTCAGCCGTGTAGGGTGAGTGAGAGGCACTAAT
CCCAGGTGTCCCTGTCACGTGTAGGGTGAGTGAGGCGTCCCAGGTGTCCCTGTCACGTGCAGG
GTGATTGTCATGAAGACCCCCGGGTGTCCCTGTCACGTGCCCAGGGTGAAGTGAAACGCCGTC
CCCGCGTGCTCCTGTCACGTGCAGGTGGTGAGTACCGTCCCGAGATTGACCCTGCCGCGTGTA
GGGTGAGTGAGGCGCCGTCCAGGTGTCCCTGTCACGTGTAGGGTGAGTGAGGTGCCGTCCCCA
GGTGTCCCTGTCACGTGCAGGGTGAGTGAGGCCGTCCCAGGTGTCCTGGCCGTTCAGGGTGAG
TGAGGGCGCGGTCCCCGAAGGTCCTGTGCACGTGTAGGGTGGAGTGAGGCACTGTCCCCCGA
GGGTGTCCCTGTCACGTGCAGGTGATTGACACCGAGGCCCCGAGGTGTCCCTGTCACGACTGC
AGGGTGAGTGAGGCGCCGTCCCCGCGTGTCCCTGTCACGTGCAGGGTGAGTGGCTCGTCCCCG
GGTGTCCCTGTCGTAGGGTGAGTGAGTGCCGTCCCCCGGGTGTCCCTGTCGTGGGGTGAGTGA
GCGCCGTCCCCAGAATTCTGTCACGTGTAGGGTGGTGAGGCGCAGTCCCCAGGTGTCCCTGTC
ACGTGTAGGGTGAGTGAGCACTGTCCCCAGGTGTCCCTGTCGTGCAGGGTGAGTGAGGCGCGG
TCCCCGGGTGTCCCTGTCGTGCCCAGGGTGAGTGGGCTGCGTCCCCGGGTGTCCCTGTCACGT
GCAGGGTGAGGTGGCGGCCCAATTGTCCCTGTCCGTGCAGGGTGAGTGAGGCCGCAGCCCCC
AGGGTGTCCTGTCACGTGCAGGGTGAGTGAGAAGCACCGTCCCAGGTGTCTCTGTCGCATTGC
AGGGTGAGTGAGGCGCGGGTCCCCAGGTGTCCTGTCATTGCAGGGTAGAGTGAGCACGGCCC
CCCGAGGTGTCCCTGTCCACGTGCAGAGGTGTAGGTATTTAGCGGCCCCAGGTGTCCCTGTCAC
GTGCAGGGTGGTGGTGGCTTGAGGTGTCCCTGTCGATGCAGAGGTGAGTGGCTGTGATCCCCA
AGGTGTCCCTGTCACGTGCAGGGTGAGTGAGTCCCAGACATTATCACATTACAGGGGTAGGTA
GTGTGATCCCGGGTGTCCCTGTCACATTCACAGGGGTGAAAGGTGAGGCTGGTCCCAAGGTGT
CCCTGTCCCGTGTAGGGTGAGGTGAGCACTGTCCCCCGGGTGTCCCTGTCACGTTCAGGGTGA
GTGAGGCGTTGGGTGTCCCTACATCACGTGTAGGGTGGAGTGAGGCACGTCCCCAGGTGTCTG
TCACGTGCAGAGGTGAGTGAGGCGCCGTCCCAGTGTCCCCTGTCACGTGCAGGGTGATTGTGC
GAGCCCCAGGTGTCCCTGTCACGTGCAGGAGTGAGTGGCGCCGTCCCAGCGTGTCCCTGTCAG
TGCCAAGGGTGATGAGGCGCCGTCCCGGGTGTCCTGTCACGTGTAGGGTGAGTGAGTGCCGTC
CCCGGGTGTCCTGTCACGTGTGGGGTGAGTGGTGCGCCGTCCCCGGGTGTCCCTGTCACGTTCA
GGGTGAGTGAGGCACGGCCCCGGGTGTCCCTGTCACGTGTAGGGTAGTGGTAGCGGTCCCGAG
GTGTCACTGTCACGTGTAGGGTGAGTGAGGCACTGTCCCCGGGTGTCCCTGTCACGTCAGGGTG
AGTGAGCGCGGCCCAGATTGTCCCTGTCATGTGCAGGGTGGTGAGGGCTTGGCCCCGGTGTCC
CTGTCACGTGTAGGGTGGTGGTAGCCCCAGGTGTCCCTGTCACGTGCAGGGTGAGTGAGGCGC

AGCCCCAGGTGTCCCTGTCACGTGCAGGGTGAGTGGCTTGCAGCCCCCGGGTGTCCACTGTCA
CGTGCCCAGGGTGAGTGGCGCAGCCCCAGGTGTCCCTGTCACGTGCAGGGTGAGTGAGGGGC
ACGGCCCCCGGTGTCCCTGTCACGTGTAGGGTGAGTGAGGCGCAGCCCCCGGGTGTCCCTGTC
ACGTGCAGTGGTGAGCTGTCCCCGGGTGTCCCTGTCACGTTCAGGGTGAGTGGCTGTGATCCCC
GGGTGTCCCTGTCACGTGTAGGGTGAATGAGGCGCCGTCCCCAGATTGTCCTGTCACGTGCAG
GGTGGTGAGCGCCGTCCCCACAGGTGTCCCTGTCACGTGCAGGGTGATTGACGAGGCCCCAGG
TGTCCTGTCCGCGTGCAGGGTGAGTGGGCGCCGTCCCCGCGTGTCCCTGTCACGTGCGGGGTG
GTGAGGCGCCGTCCCCGGGTGTCCCTGTCACGTGTAGAGGGTGAGTGAGGCGCCGTCCCCGG
GTGTCCCTGTCACGTGTAGGGTGAGTGAGGCGCCGTCTTTGGGTGTCCCTGTCACGTGTAGGGT
GAGTGAGGCGCCGTCCCGGGTGTCCCTGTCACGTGTAGGGTGAAGGTGAGGCGCCGTCCCGG
GTGTCCCTGTCACGTGCAGGGAGCAATGCGTAACTAA

**Read ID A-reverse complement:**

TTAGTTACGCATTGCTCCCTGCACGTGACAGGGACACCCGGGACGGCGCCTCACCTTCAC
CCTACACGTGACAGGGACACCCGGGACGGCGCCTCACTCACCCTACACGTGACAGGGACA
CCCAAAGACGGCGCCTCACTCACCCTACACGTGACAGGGACACCCGGGGACGGCGCCTCA
CTCACCCTCTACACGTGACAGGGACACCCGGGGACGGCGCCTCACCACCCCGCACGTGAC
AGGGACACGCGGGGACGGCGCCCACTCACCCTGCACGCGGACAGGACACCTGGGGCCTCG
TCAATCACCCTGCACGTGACAGGGACACCTGTGGGGACGGCGCTCACCACCCTGCACGTG
ACAGGACAATCTGGGGACGGCGCCTCATTCACCCTACACGTGACAGGGACACCCGGGGAT
CACAGCCACTCACCCTGAACGTGACAGGGACACCCGGGGACAGCTCACCACTGCACGTGA
CAGGGACACCCGGGGGCTGCGCCTCACTCACCCTACACGTGACAGGGACACCGGGGGCCG
TGCCCCTCACTCACCCTGCACGTGACAGGGACACCTGGGGCTGCGCCACTCACCCTGGGC
ACGTGACAGTGGACACCCGGGGGCTGCAAGCCACTCACCCTGCACGTGACAGGGACACCT
GGGGCTGCGCCTCACTCACCCTGCACGTGACAGGGACACCTGGGGCTACCACCACCCTAC
ACGTGACAGGGACACCGGGGCCAAGCCCTCACCACCCTGCACATGACAGGGACAATCTGG
GCCGCGCTCACTCACCCTGACGTGACAGGGACACCCGGGGACAGTGCCTCACTCACCCTA
CACGTGACAGTGACACCTCGGGACCGCTACCACTACCCTACACGTGACAGGGACACCCGG
GGCCGTGCCTCACTCACCCTGAACGTGACAGGGACACCCGGGGACGGCGCACCACTCACC
CCACACGTGACAGGACACCCGGGGACGGCACTCACTCACCCTACACGTGACAGGACACCC
GGGACGGCGCCTCATCACCCTTGGCACTGACAGGGACACGCTGGGACGGCGCCACTCACT
CCTGCACGTGACAGGGACACCTGGGGCTCGCACAATCACCCTGCACGTGACAGGGGACAC
TGGGACGGCGCCTCACTCACCTCTGCACGTGACAGACACCTGGGGACGTGCCTCACTCCA
CCCTACACGTGATGTAGGGACACCCAACGCCTCACTCACCCTGAACGTGACAGGGACACC
CGGGGGACAGTGCTCACCTCACCCTACACGGGACAGGGACACCTTGGGACCAGCCTCACC
TTTCACCCCTGTGAATGTGACAGGGACACCCGGGATCACACTACCTACCCCTGTAATGTG
ATAATGTCTGGGACTCACTCACCCTGCACGTGACAGGGACACCTTGGGGATCACAGCCAC
TCACCTCTGCATCGACAGGGACACCTCAAGCCACCACCACCCTGCACGTGACAGGGACAC
CTGGGGCCGCTAAATACCTACACCTCTGCACGTGGACAGGGACACCTCGGGGGGCCGTGC
TCACTCTACCCTGCAATGACAGGACACCTGGGGACCCGCGCCTCACTCACCCTGCAATGC
GACAGAGACACCTGGGACGGTGCTTCTCACTCACCCTGCACGTGACAGGACACCCTGGGG
GCTGCGGCCTCACTCACCCTGCACGGACAGGGACAATTGGGCCGCCACCTCACCCTGCAC
GTGACAGGGACACCCGGGGACGCAGCCCACTCACCCTGGGCACGACAGGGACACCCGGGG
ACCGCGCCTCACTCACCCTGCACGACAGGGACACCTGGGGACAGTGCTCACTCACCCTAC

ACGTGACAGGGACACCTGGGGACTGCGCCTCACCACCCTACACGTGACAGAATTCTGGGG
ACGGCGCTCACTCACCCCACGACAGGGACACCCGGGGGACGGCACTCACTCACCCTACGA
CAGGGACACCCGGGGACGAGCCACTCACCCTGCACGTGACAGGGACACGCGGGGACGGCG
CCTCACTCACCCTGCAGTCGTGACAGGGACACCTCGGGGCCTCGGTGTCAATCACCTGCA
CGTGACAGGGACACCCTCGGGGGACAGTGCCTCACTCCACCCTACACGTGCACAGGACCT
TCGGGGACCGCGCCCTCACTCACCCTGAACGGCCAGGACACCTGGGACGGCCTCACTCAC
CCTGCACGTGACAGGGACACCTGGGGACGGCACCTCACTCACCCTACACGTGACAGGGAC
ACCTGGACGGCGCCTCACTCACCCTACACGCGGCAGGGTCAATCTCGGGACGGTACTCAC
CACCTGCACGTGACAGGAGCACGCGGGGACGGCGTTTCACTTCACCCTGGGCACGTGACA
GGGACACCCGGGGGTCTTCATGACAATCACCCTGCACGTGACAGGGACACCTGGGACGCC
TCACTCACCCTACACGTGACAGGGACACCTGGGATTAGTGCCTCTCACTCACCCTACACG
GCTGACACCTGGGGATTTGGCGCAGTCACTCACCCTACACGTGCATAGACCTGGGACGGC
ACCTCACCACCCTACACGTGACAGGGACACGATTTGCGCCCCCACTCACCCTGCACGTGA
CAGGACCACGGCGGGGACTATGCCTCACTCACCCTGCACGTGTGACAGGACACCCGGGGC
CTCTTTCGCGTCAATCACCCTGCACGTGACAGGGACACCCGGGGGACGGCGCCTCACTCC
ACCCTGCACGTACAGGGACACCCGGGGGACAGTGCCTCACTCACCTCACACGTGACAGGG
ACACCCGGGGACCGCGCCTCACTCACCCCGGAACGTGACAGACACCCAGACAGTGCCTCA
CCAATCTCACACGGACAGGGACACCTGGGGACCGCGCCTCACTCACCCTGCACGTGACAG
GAGCCACCCGGGGGACCGCGCTCACTCACCCTGCACGTGACAGGGACAAATTCCGGGGAC
CGCGCCTCACTCACCCTGCACGTGACAGGACACCGGGGGCCGCGCCTCACCACCTGAATG
CGACAGGACACCCGGGGCTGCGCTCACCACCCCCGGCACGTGACAGACACCTGGGGCCGC
GCCTCACTCACCCTGCACGTGACAGGGACACCCAGGCTGCCTCACCACCCTGCACGTGAC
AGGGACACCTGGGGATTTCTGCGCCTCACCACCCTCGTGCACGTGACAGAGACACCCGGG
GACGGCGCTCACTCACCCTGCACGGACAGGACACCCGGGACGGCGCCTCACTCACCCTGC
ACGTGACAGGACACCCAGGGGCTTCGTGCCTCACCACCCTGCATCGCATAATACCTTCGT
GGGGAGCAGCACCACCCACCCTGCACGGTGACAGAGACACCCGGGGATCATGCCTCACCC
CCCACCCTGCACGGGACAGGGAAATTCGGGGACGGCGCCTCACCACCCTGCACGACAGGG
ACACCCGGGGGATCAAGCTCACCACCCTGCACGTGACAGGGACACCCGGGGCCGCGCCCT
CACCACCCTGCACGTACAGACACCTGGGGGGGGGCCGTGCTCTCACTCACCCTGCACGTG
ACAGGACACCTGGGATGCCTCACTCACCCTGCAATGTGACAGGGACACCTCGGACCTGCG
CCTCACTCACCCTGCACGTGACAGGGACACCCGGATAAGTGCCTGCCTGAATTTTCTGCA
CGGGACAGGGACACTCGGGACGGCGTGCGCCTCACTCAATTCTGCACGGGACAGGGACAC
CCGGGACGACCAATTTATGCCTACAGCTCTTAGTACAAAGACTAGGCCAATCTAGCCCTC
CCCAACTCCTCCCAGTCCTTAAACAAACAGTCTTTGAAGTAGTTCTCAAGGTCGGTCGTT
GACATTGCCGTTGTTATCCAAGAGTCCTCTTACAAGACCTTGGGCAAGACCTGGTGGGCG
TTCACGGTGGTCGCCATGCGACGCAGAGGTGAAGGAAGCGCACACGGTCCGGCAGATGAG
AAGGCGCCAGACGGGGAGACCGCGTGAAGAGGCGCTGTGGACGTGGCTGGAACGGCAGAT
GAAGAAGGGGACGAAGTCTCAACGGCCCCGAGACGGTCAAGCCCCGGTGATCTGGGAAGC
GCCGACGGGACGTAGACAAAGGACGTTCCGCGCAGGATCCAGTTGGCAGCACACCCCAGC
AGCCATGGAAAGGAGGTGAGCTTGGAGAGAGGACAAACAGAGTTAGTTGATCCTGATAAG
TTTTGCCCAGACTGCCAAGAGCAAACAAGCTGCTAGGGAGTTCCGCAGTGCTGGGACGGC
AGGGTGGTAGAGGAGCCACAAGTTCCACGCATGTGGCGATGGCCAATAGCCAAGCCCCAT
CCAGTGGGGTTGCGTCAAAACACTTATAGACTCATCGTTGCCGGGCAACGGGGTAAAGGG
TTCGTATTAGCTTAAGCTTTTTACACAGAAAGGCCTTGTAAGTTGGCGAGAAAGTGAAAG

CCTGCTTAGATTGTATACATGCATATAAAGGCATTAAGGATAGCCACATTGTGTAAAAGG
CAGCAAAAGCCTAGAAGACCCACAATTCTCTGATATACTTTCCAATCAATAGGTTCATTT
ACAGGCAGTTTTCAGAAAAACATTGTTGATTTATACAATATTCTATGGTAAAGTACCCCA
ACATCCAATTACATATCCCATGAAATTAAGGGAGTAGCCCCAACGTTGGTTTTATTAGGG
TTCAATGTATACTCCAAAAGACAAAAGAAAATTGGTAATAGAGGCAAAAAGGGACTCCTA
AAGATGTTGTACAGACTTGGCCCCCAATACCACATCATCCACATATAACTGAAAGGCAGA
CAGTGGGAAAGTCTACGAACCACTGAACAAATGGCACTAGTAAACTGAGCCAGGAGAAAT
GGACTGAGGCCCAATTCCCATAGAATCTTATGCAGCTCCAGGATGATGGGATGGGAATAC
AAGTGCAGTTTCCTGTCCGAAGGTTTGGGTACAGCAACAAGAGGGAAACATAGAGGTGTT
CCTCCTTTGAGCAGGAGTCGCAGGCTTTGCATGGTCCCAGCTGGTAGTTGATGTTCCTGT
TGCAGGACAAACGGGCAACATACCTGGTGTCCAGAAGAACCAACAAGAAGACATGAGGCA
TAGCAGTGGATGAAGAGGAATATGATAAAACGTGCCGCAGACACATCCAGCGATAGCCAG
GACAAATTGGAGGATGCATGAGGTTAAGTATGATTGAGGTTGGGACCGCGGACGGCTAGA
CACGCGGTGCTCCCTAGAAATTAGAGAAGTCCAATTAATGAGTCTAGACTCTGTGGTATT
GTGAGGATTCTGTCGTACTCAAGAAACGCCTGTAACCCGAGCAGGGTCCTAGGAACTCTG
ATGTTGTGTTCTCCATGTGCCTGCAGGGTCCCCAGTCCCTCAGAGAAGATTGGACGATAT
GGTGTAGAGTAGTCGGAATTGTGGCTTCTATTGTTCCGGGAACTGGAGCCACCTCCAGCA
GGAAGTATTTGGTGAGTTGTGGAGTTCCCACTGCATGGCCTGAGGATGACTGTCTCTTTT
TTAGAGGAGATGGGAGTAGGCTGTCTCTGACTGCCGATTGGTGGAGGCAGGAGGAGGTGC
TACTGGCACTGTTGTCAATATGCCCTGAGCCTGAGGGCTCCACCCCAAAAGACTGCCGTG
GTGGGGTGAACCCTGGCCCGAATGCTCCCTGGGGTTCTACCTGATTGCCTCTGGCCAGTG
ATCCTTGCTAGGCTGTGTCCCAATTGGATTGTTTGAGTTGGCTCCGGAACGCAGGTCCAA
CTGTGGTGATCGGGAAAGAATCCCTAGAGGATTGGGAACAGAAAGATCTGTCCCATGCCT
TTCGAGGTCGTCTGGAAGACCAACCCTCCCATGCTGTAGCTCTTGTTCCCAAGAATATAT
GGACCCAACAAAATGAGGTAAGGCACAGTGTGTAGTTTCTCTTATATAGGAATGCCCGCC
TTCCACAGAGTATGTAAATAATGCCTAGTTTTGAAGTAATGATTAACTGCATGTTCAGGA
TAATATGGCTTAATGCCTAGATATTTGGTAAGGTTAGATAGAAACTCTACAGGCATAATT
AATTTTAATTTTTTTCATTAACTGTAAGAGGGCCCACATATTGCGACTATTAACAATAAT
CCTCCGTAAATGAATGAGGAAAGGAGGGAGTTTGCCACTCAGATTGTAAAGACAGGTACG
GTAGTTAACAAAGCCCAGAAAAGTTTCCCACCTTATGAGTCCAAGGGATACTAACATTAA
GATTCCCGAGAGTGAGATCTTCTGCGACGCGGCATTGAGACCTCGTCTGCGAGGCGGCGA
GGGAGCTCCCTCCTTCTAGGGGACCTGCATCGTCGTTCAACAGTAGTTTCCGGAAGTGTT
GATAAGATAGCATTTTTGTGGTCTGTAAGCAGGGAGGAGCGCGAATCCACACTCCAAAAG
ACACCAAATACTCAAGAACAGTTTCTCTTCTTCCAGGAACATCAACTACCAGCACGGGAC
CATGCAAGACCTGCACGACTCCTGCTCAAGGAACCTCTATGTTTCCCTCTTGTTGCTGTA
CAAAACCTTCGACTTTGAAACTGCACTTGTATTCCCATCCCATCATCCTGGCTGCAAGAT
TCCTATGGGAGTGGGCCTCAGTCCGTTTCTCCTGGCTCAGTTTAATAGTGCCATTTGTTC
AGTGGTTCGTAGGGCTTTCCCCCCACTGTCTGGCTTTCAGTTATATGGATGAGGGTATTG
GGGGCCAAGTCTGTACAACATCTTGAGTCCCTTTACCTCTATTACCAATTTTCTTTTGTC
TTGCATACATTTGAACCCTAATAAAACCAAACGTTGGGGCTACTCCCTTTACCATGGGAT
ATGTAATTGGATGTTGGGGTACTTACCGCAAGAACATATTGTACTAAAAATCAAGCAATG
TTTTCGAAAACTGCCTGTAAATAGACCTATTGATTGGAAAGTATGTCGAGAATTGTGGGT
CTTCTGGGCTTTGCTGCCCCTTTTACACAATGTGGCTATCCTGCCTTAATGCCTTTATAT
GCATGTATACACCCAAGCAGGCTTTCACTTTCTCGCCAACTTACAAGGCCTTTCTGTGTG

TCGGGGCCATCTGAACCTTACCCGTTGCCCGGCAACGGTCAGGTCTCTGCCAAGTGTTTA
AAAGCTGACGCAACCCCCACTGGATGGGCTTGGCTATTGGCCATCGCCGCATGCGTGGAA
CCTTGTGGCTCCTCTACCGATCCATACTGCGGAACTCCTAGCAGCTTGTTTTGCTCGCAG
CCGGTCTGGAGCGAAACTATCGGAACCGACAACCTGTTGTCCTCTCTCGAAATACACCCT
TTCCATGGTTGCCAGGGTGTGCTGCCAACTGGATCCTGCGCGGAACGTCTTATGTCTACG
TCCCGTCGGCGCTGAATCCTGCGGACGACCCGTCTCGGGGGCCGTTTGGGGCTTCACTTC
GTCCCCTTCTTCATCTGCCGTCTCGGGCCGACCACGGGGCGCACTCCTTTCTACGCGGGG
TCTCCCCGTCTGTGCCTTCTCATTCGCCGGACCGTGCACTTCGCTTCACCTCTGCACGTC
GCATGGCGACCACCAGGGAACGCCCACCAGGTCTTGCCCAAGGTCTTACATAAGAGGACT
CTTGGACTCGCAGCAATGTCAACGACCGACCTTGAGGCATACTCAAAGACTGTTTGTTTA
AGGACTGGGAGGAGTTGGGGAGGAGACTCAGGCACGATCTTTGGTACCAGGAGGCTGTAG
GCATAAATGGTCTGTTCACCAGCACTATGCAACTTTGGGGCCCCTCGCCATGTCCTGCAC
CCTGGGAGCGAGCGGCGCGCGGGCGGGGAAGCGCGCCCGACTTCGGGTCCGCCCGGAGCA
GCTGCGCTGTCGGCCAGGCCGGCCCCAGTGGATCTGCGGGCACAGATTTTTCCGTGGACC
GCGCTCCCCACGTGGCGGAGGGACTGGGACCCGGGCACCCGTCCGCTCCTTCACCTTCCA
GCTCCGCTCCTCCGCGCGGACCCCGCCCTGTCCCGACCCTCCCGGGTCCCCGGCCCAGCC
TCCGGGCCCTCCCAGCCCCTCCCCTTCCTTCTGCGGCCCGCCTCCTCGCGGCGCGAGTTT
CAGGCAGCGCTGCGTCCTGCTGCGCACGTGGGAAGCCCTGGCTCCCGGCCACCCCGCGAT
GCCGCGCGCTCCCGCTGCTGAGCCGTAAGCTCCTTGCTGCGCAGCCACTACCGCGAGGTG
CTGCCGCTGGCCACGCCTCGTGCGGCGCCTGGGGCCCCAGGGCTGGCGGCTGGTGCAGCG
CGGACCCGGCGGCTTTCCGCGCGCTGGTGGCCCAGTGCCTGGTGTGCCCTGGGATGCACG
TCGCCCCCGCCGCCCTCCTTCCGCCAGGTGGCCTCCCGGGGTCGCCGTCCGGCTGGGGTT
GAGGGCGGCCGGGGGGAACCAGCGACATGGAGAGCAGCGCAGTGACTCAGGCGCTTCCGC
AGGTGTCCTGCCTGGAAGGGAGCTGGTGGCCCGAGTGCTGCAGAGGCTGTGCAGCGCAGC
GAAGAACGTGCTGGCCTTCGGCTTCGCGCGCTGCTGGATTGGACTCGCGGGGGCCCCTCG
AGGCCTTCACCACCAGCGTGCGCAGCTACCTTCGCCCAATACGGTGACCGGTGACACTGC
GGGGGAAAGCGGGGCGGGGGCTGCCGCTGCGCCGCGTGGGCGACGACGCTGGTCACCTCG
CTGGTACGCTGCGCGCTCTTGTGCTGGTGGCCCCCAGCTGCGCCTACCAGGTGCGGGCCG
CCGCTGTACCAAGCAATACGTAACTGAACGAAGTACAT

**Read ID B Sequence:**
TGTACTTCGTTCAGTTACGTATTTACTGGTATACAAAATTCACTTTGAACATTGAGTGGATTTCCA
GGGTGTGGAAGTGGGGCATATGGAATGAGAGCACCTGGAGCCCTGTTGCAGTATCGTTTCCTG
CAGTCTGGAAGTGTGCTCCTTCCCACTTGGCCTCCATGCTTGCAAACCATTGGGCACAATCATAT
CCCATGCTTGACAAGCCAAGAAGAAAGAGGCGAGATCAACTAATGATGGACTTTAGTTTTCAGG
GAAACATAATCCCTGACCCTAAATTGTGGCATCTTCATGGGGCTCCTGATCACATTGCCTTCACA
GCACCCAGCCTCAGAGGCCCATTCTTGCCTCCACTACAGGGATGGGTGTCAGATTCACAGAATG
ATGCATCAAATGGCAACAACAACCATAAAAAGTGTAAAACAAAAGCAGGTTCCAAACATGGGC
AGCTCCTGTTCTGACACAGATAGTATTCTTGCTGTAGTAAGTATCCTCTTGGTCAAGTGAGTTAA
CCTCTTGTAGTGATGGTCAGGGCTTGTTGCTCTTACTTTTACAAACCTATAAGAGGAAAAGTAAA
CTCACCATATCAGCAGGGCAGGACTGTCAGATGGTACATTATACCCACATTATCTGGGCCGTGA
ACACACACAGCCAGGGACAGGGAGTGGGCCCCACAGGTCACCAATAAGTGCCATTTTCACCTA
GGTCTGTTGATCTGGCCTATGGTTACTTATTACAAATATGAGCCTAGCACTTTCTTTAGAGGATCT
GAGTGTCTGGATTGTAATCTGATACTACAACCCGCAGCCTCAACCCCATACATCCACGTGGCAC

TCACAGAAAGGGACACTCAATCTTCCTCCCTTCCTCACCTCACTTCATGCCTGAGCTGACAGCA
GGCATCCCATGGCCCATCCTAATGTGTCCCCTTTTGCCATCTGCCATGCCTGGAGATCGCTCTCT
GGGAACATCTGACTTTGGGACAGGACAGAACATCAGCAGGACACTAGGTGTGAACCCATGGGC
TTTATTGCTGCTTTAACTCTCTATCTTCCTTGTTCTTGGCCTACCTGGTTCTGAGTGTGTCTAGGCA
GGGTTGGAAGATGCCATCAGACTCAAAATGGATTAGATTGCTCTCTCTGTCTGGATTTCCCTCTT
CTCAAAATCCAGCTGCTCACTGGAGGGTTCCATCCAGTCTGCCCGGTTGATGTACAAGATCCCT
GTGAGCACAGAGAATACAGTTTAGTGAATATTCCATATACATCATTATATTTTTCACCCCAGGGA
GTTATTTTCTCTGTTTTGGAAAATGTACCTGAAGACTGGACTGAAAGAAAAGGAGATTTGGCCAA
GATGTGAGAGGTAAATGGGTTTGCTGTCGGACCAACTCCAGCAGACTGATGAGTAGATGGATG
GGAGCAGCTTTGAGAATCCTGAGAGATTCTCAAAAGCTGAGAGAATACCTAATTTATGCCTACA
GCCTCCTAGTACAAAGACCTTTAACCTAATCTCCTCCCCCAGCTCCTCCCAGTCTTTAAACACAC
AGTCTTTGAAGTACGCCTCAAGGTCGGTCGTTGACATTGCTGAGAGTCCAGAGTCCTCTTATGTA
AGACCTTAGGCAAGACCTGATTGGCGTTCACGGTGTCTCAGCCGACGTGCAGGAGTGAAGCGA
AGTGCACACGGTCCGGCAGATGAGAAGGCACGAACGGAGACCGCGTAAAAGAGAGTGCGCCC
CGTGGTCGGCCGGAACGGCAGACAGAGAAAGGGGACAGTAGATTCCCAAGCGACCCCGAGAT
GGGTCGTCCGCGGGATTCAGCGCCGACGGGACGTAGACAAAGGATGTCCGTGCAGGATCCAG
TTGGCAGCACAGCCGAGCAGCCATGGAAACGAGGTGTATTTCAGGAGAGAACAGGATTGTCGG
TTCCGATGAGTTTTGCTCCAGACCGGCTGCGAGCAAAACAAGCTGCAGGATTCCGCAGTATGGA
TCGGCAGAGGAGCCACAAGGTTCACGCATGCGCCGATGGCCCTTCTTCAGCCCCAACCAGTGG
GGGTTGCGTCAGCAAACACTTGGCATAGACCTGGCCGTTGCCTGGCAACGGGGTAAAGAGGTC
AGATATTGTTTACGAAAGGTCTTGTAAGTTGGCGAGAAGTGAAAGCCTGCTTGGCCCGTATACA
GCCATACAAAGGCAGTAAGGCAGGATAACCACATTGTGTAAAGGGGCAGCAAAGCCCAAAAA
AGACCCACAATTCTTTGAGCATACTTTCCAATCAATAGGCCCCTATTACAGGCAGTTTTCGAAAA
CACTGCTTGAGTTCATTACAATATGTTCCTGTGGTAGGTACTGGCAATTACATACATGAAGTTCG
GGAGTAGCCCCAACGCTTGGTTTTATTAGGGTTCAAATGGATACCCCAAAAGATAAAAAAGAAA
ATTGGTAACAGAGGTAAAGGGATTCAAGATGTTGTACAGACTTGGCCCCAATACCACATCATCC
ATATAACTGAAAGCCAAACAGTGGGGAAAGCCCTACGAACCACTGAACAAATGGCGCTAGTAA
ACTGAGCCAGGAGAAACGGACTGAGGCCCACTCCCATAGGAATTTTGCGAAAGCCCAGGATGA
TGGGATGGGAATACAGGTGCAGTTTCCGTCGAAGGTTTTATGCAGCAACAGAGGGAAACATAG
GATGCCTTGAGCAGTCGTGCAGGTCTTGCATGGTCCGTGCTTGTTGTTGATGTTCCTGGAATTAG
AGGACAAACAGACGCTATACCTTGGTAGTCCAGAAGAACCAACAAGAAGATGAAACAACAGC
AGGATGAAGATATGATAAAACGCCGCAGACACATCAAGCGTGTCCAGGACAAATTGGAGGACA
AGAGGTTGGTGAGTGATTGGAGGTTGGGGACTGCGAATTTTGGCCAGGACATGGGTGCTCCCT
AGAAAATTGAAAGTCCACCACAGTCTAGAACTCTGCGGTATTGGCGAGATTCTTGTCAACAAGA
AAAACCCCGCCTGTAACACGAGCAGGGGTCCTAGGAATCCTGATGTTGTGTTCTCCATGTTCAG
TGCAGGGTCCCCAATCCTCGAGAAGATTGACGATATGGGAGAGGCAGTAGTTGGAACAGGGTT
TGCTGTTCGAACTGGAAACCACCAACCCCCTTAGGAGAAATGTGTTTCCTCTGGGGATCTAGCG
GAAGCAGTGAAGGTTGTGGAGTTCCATTGCATGGCCTAGAGGATGACTGTCTCTCAAGAGGTG
GAGAGATGGGAGTAGGCTGCCTTTCTGACTGCCGATTGGTGGAGGCAAGGAGGAGGAGTTGCT
GGCACTGTTGTGTATGCCGCAGGCCTGAGGCTCCACCCCAAAGACCGCCGTGCGGGTGGGGTG
AACCCCTGACCCAGTGCTCCCACTCCTACCTGCCAGGCCTCGTAGGGGTCCTTGTTGGGGTTGC
CCTTTCTGACTTCTTTCCTTCTATTCGAGATCTCCTCAGCACCATACCTCCTGCTCTGTAGGAGGC
CTTAGAGTCTCCGGAACATTGTTCACCTCACCATACACAGCACTAAGGCAAGCTATTCTGTGTTG
GGGTGAGTTAATCCATAGAGAGGAAGTAATTTGGAAGATCCAGCATCCAGGGGTGGTAGTCAG

CTATGTCAAGTATTGTATGGGCCTAAGATCAGACAACTATTGTGGTTTCACATTTCTTGTCTTACT
TTTGGAAAGAGAAACGGTTCTTGAGTATTTGGCGTTATCCTTGGCAGGGTGTGGATTCGCACAC
CCCCGCCTGAGCCGCCAGATACCCCTATCTTATCAACACTTCGGAGACTACTGTTGTTAGACGA
CGCGAGGCAGGTCCCTAGAAGAAGGGAACTCCTCGCCTCGCAGACAGGTCTCAATCCTTGTAA
TCCGCCAGAAGATCTCAATCTCGGGAATCTGGCATTAGTATCCCTTGGACTCATAAGGTGGGAG
GAAACTTTGCAGGCTTTATTCTTCTACTGTGCAATCTTCCAATCACGAGATGGCAAACTCCCTCTT
TTCACAGTATACATTTGCAGGAAGACATTGGTAATAGATGTCCAACAATTTGTGGGCCCTCTAAA
TGCAAATGAAAAATTAAAGCTAATTATGCCACTTGATTTTTCCTAGCCTTACCAAATGTTGCCATT
GGATAAAGGTATTAAACCTTATTATCCTGAACATTCAGTTAATCATTTATTCAAAACTAGGCATTA
TTTGCATACCCTTTGGAAGGCTGGCATTCTATATAGAAACTACACGCAGTGCATCATTTTGTGGG
TCACCATATTCTTGGGAACAAGAGCTACAGCATGGGAGGTTATTCCTCAAAACCTCGAAAAGGC
ATGGGGACGAATCTTTCTGTCCCAATCCTCTGGGATTCCTGCCCGACCATCAGTTGGACCCGGC
TTTCGGGCAAATTCAAACAATCCAGATTGGGACTTCAACCCCAACAAGGACCCTTGGCCAGAG
GCCTGGCAGGTAGAATCAGGAGCATTCGGGTCAGGGTTCACCCCACCGCACGGCGGTCTTTTG
GGAGGTGGAGCCCTCCAGGCTGGGGCATATTGACAACAGTGCCAGCAACTCCTCCTCTGCCTC
CACACCAATCAACAGTCAGAAAATGGCCTCTCCCATCTCTCCACCTCTGAGAGACAGTCATCCT
CAGGCCATGCAATGCGTCCACAACCTTTCATCAGAAAGCTCTGCTAGATCCCAGTGAGGGCTAT
CTGCTCCTGCAGTGGCTCCAGTTCCGGAACAGCAAACCCTGTTCCAACTACTGCCTCTCCCATAT
CATGGTAATCTTCTCGAGGATTGGGGACCTACCGAACATGGAGAACACAACATCAGAGTTCTAG
GACCCCTGCTCGTGTTACAGGCGGGGTTTTTCAGCATTGACAAAGATCCTCCACAGCTCCGCAG
AGTCTGGGCTCGTGGTGGGACTTCTCTCACAGTTTCACAGGGAGCACCCACGTGTCACAGCCCA
AAATTCGCAGTCCCCAACCTCCAATCATCACCAACCTCTTGTCCTCCAATTTGTCCTGGCTATCG
CTGGATGTGTCTGCGGCGTTTTATCATATTCCTCTTCATCCTCTTTCTGTACCTCATCTTCTTGTTT
GGTTCTTCTGGACTACCAAGGTATCTGTTGCCCGTTTATCCTCTAATTCCAGAACATCAACAACA
AGCACGGGACCATGCAAGACCTGCGACTCAATAGGCACCTCTATATTTTCCCTCTTGTTGCTGTA
CAAAACCTTCGGACGGAAACTGCACCTGTATTCATCCCATCATCTGTTTCGCAAAATTCCTATGG
GAGTGGGCCTCAGTCCGTTTTCTCCTGGCTCAGTTTACTAGCGCCATTTGTTCAGTGGTTGTAGG
GCTTTCCCACTGTTTGGCTTTCAGTTATATGGATGATGTGGTATTGGGGGCCAAGTCATGCAACT
CTTGAATCCCTTTTACCTCTGTTACCCAATTTTCTTTTATCTTTTTGGGTATCCATTTGGAGCCTAAT
AAAACAAAGCGTTAGGGCTACTCCTGAACTTCCATGGGATATGTAATTGGAAGTTGGGGTACCT
TACCACAGGAACATATTGTAATGAAAATCAAGCAGTCTTTTCGAAAACTGCCTGTAAATAGGCC
TATTGATTGGAAAGTATGTCAAAGAATTGTGGGTCTTTGGGCTTTGCTGCCTTTTACACAATGTG
GTTATCCTGCCTTACTGCCTTTATGCATGTATACAAGCTAAGCGAGCTTTCACTTTCTCGCCAACT
TACAAGACACTTATATTATTAAACAATATCTGACCCGCTACCCCGTTGCCAGGCAACGCTCAGG
TCTATGCCAAGTGTTTACTGACATAACCCCCACTGGTTGGAAGCGGCCACTTGACCATCAGCAC
CGTGCGATGGAACCTTTGTGGCTCTCTGCCGATCCATACTGCGGAACTCCTTGCAGCTTGTTTTG
CTCGCAGCCGGTCTGGGGCAAAACTCATCGGAACCGACAACTCTGTTGTTCTCGCAAAATACAC
CTCGTTTCCATGGCTGCTCGGCTGTGCTGCCAACTGGATCCTGCGCGGGACATCCTTTGTCTACG
TCCCGTCGGCGCTGAATCCGCGGACGACCCATCTCCGGGGTCGCTTGAGATCTACCGTCCCCTT
CTCCGTCTGCCGTTCCGGCCGACCACGGGACACCTCTCTTTACGCGGTCTCCCCGTCTGTGCCTT
CTCATCTGCCGGACCGTGTGCCGCCCAGCTTCACCTCTGCGTCGCATGGAGACCACCATGAACG
CCAATCAGGTGCCAGATCTGCATAAGAGGGACTCTTGGACTCTCAGCAATGTCAACGACCGAC
CTTGAGGCGTACTTCAAAGACTGTGTGTTTAGAACTGGGAGGAGGTTGGGGGAGGGAATTAGG
TTAAAGGTCTTTGTACTAGGAGGCTGTAGGCATAAATTAGGGCATTCTCCACAAGCTTTGAGAAT

CTCTCTAGGATTCTCAGCTGCTCCCCATCCATCTATCATCAAGTCCGCTGGAGTTGGTCCTCAAT
AGCAAACCCATTTACCCTCTCACATCTTGGCCAAATCTCCTTTTCTTTTCAGTCCAGTCTTCTGTG
TACATTTTCCAAACAGAGAAAATAACTCCTGGGGTGAAAAATATAATGATGTATATGGAATATTC
ACTAAACTGTATTCTCTGTGCTCACAGGGATCTTGTACATCAACCCGGCAGACTTGGGATGGAA
CCCTCCAGTAAGGCAGCTGGATTGAGAAGAGGAAATCCAAGACAGAGAGAGCCAACAACTCAT
TTTGTTCGACAAGTATCTTCCAACCTGCCTAGACACATCAGAACCAGGTAGGCCAAGAAACAAG
GAAGATAGAGAGTTAAAGCAGCAATAAAAAGCCCCATGGGTTCACACCTAGTGTCCTGCTGAT
GTTCTGTCCTGTCCCAAAGTCAGATGTTCCCAGAGCGATCTCCTCCAGGCATGGCAGATGGCAA
AGAGGACACACATTAGGATGGCCATGGGATGCCTGTTGTCAGCTCAGGCATGAAGTGAAGACG
AGTGAAGGGGAGGAAGATTGAAGTCCCTTTCGTGAGTGCCACGTGGATGTATGTGAAGGAGGC
TCAAGGTTGTAGTATCAGATTA

**Read ID B-reverse complement:**
TAATCTGATACTACAACCTTGAGCCTCCTTCACATACATCCACGTGGCACTCACGAAAGG
GACTTCAATCTTCCTCCCCTTCACTCGTCTTCACTTCATGCCTGAGCTGACAACAGGCAT
CCCATGGCCATCCTAATGTGTGTCCTCTTTGCCATCTGCCATGCCTGGAGGAGATCGCTC
TGGGAACATCTGACTTTGGGACAGGACAGAACATCAGCAGGACACTAGGTGTGAACCCAT
GGGGCTTTTTATTGCTGCTTTAACTCTCTATCTTCCTTGTTTCTTGGCCTACCTGGTTCT
GATGTGTCTAGGCAGGTTGGAAGATACTTGTCGAACAAAATGAGTTGTTGGCTCTCTCTG
TCTTGGATTTCCTCTTCTCAATCCAGCTGCCTTACTGGAGGGTTCCATCCCAAGTCTGCC
GGGTTGATGTACAAGATCCCTGTGAGCACAGAGAATACAGTTTAGTGAATATTCCATATA
CATCATTATATTTTTCACCCCAGGAGTTATTTTCTCTGTTTGGAAAATGTACACAGAAGA
CTGGACTGAAAAGAAAAGGAGATTTGGCCAAGATGTGAGAGGGTAAATGGGTTTGCTATT
GAGGACCAACTCCAGCGGACTTGATGATAGATGGATGGGGAGCAGCTGAGAATCCTAGAG
AGATTCTCAAAGCTTGTGGAGAATGCCCTAATTTATGCCTACAGCCTCCTAGTACAAAGA
CCTTTAACCTAATTCCCTCCCCCAACCTCCTCCCAGTTCTAAACACACAGTCTTTGAAGT
ACGCCTCAAGGTCGGTCGTTGACATTGCTGAGAGTCCAAGAGTCCCTCTTATGCAGATCT
GGCACCTGATTGGCGTTCATGGTGGTCTCCATGCGACGCAGAGGTGAAGCTGGGCGGCAC
ACGGTCCGGCAGATGAGAAGGCACAGACGGGGAGACCGCGTAAAGAGAGGTGTCCCGTGG
TCGGCCGGAACGGCAGACGGAGAAGGGGACGGTAGATCTCAAGCGACCCCGGAGATGGGT
CGTCCGCGGATTCAGCGCCGACGGGACGTAGACAAAGGATGTCCCGCGCAGGATCCAGTT
GGCAGCACAGCCGAGCAGCCATGGAAACGAGGTGTATTTTGCGAGAACAACAGAGTTGTC
GGTTCCGATGAGTTTTGCCCCAGACCGGCTGCGAGCAAAACAAGCTGCAAGGAGTTCCGC
AGTATGGATCGGCAGAGAGCCACAAAGGTTCCATCGCACGGTGCTGATGGTCAAGTGGCC
GCTTCCAACCAGTGGGGGTTATGTCAGTAAACACTTGGCATAGACCTGAGCGTTGCCTGG
CAACGGGGTAGCGGGTCAGATATTGTTTAATAATATAAGTGTCTTGTAAGTTGGCGAGAA
AGTGAAAGCTCGCTTAGCTTGTATACATGCATAAAGGCAGTAAGGCAGGATAACCACATT
GTGTAAAAGGCAGCAAAGCCCAAAGACCCACAATTCTTTGACATACTTTCCAATCAATAG
GCCTATTTACAGGCAGTTTTCGAAAAGACTGCTTGATTTTCATTACAATATGTTCCTGTG
GTAAGGTACCCCAACTTCCAATTACATATCCCATGGAAGTTCAGGAGTAGCCCTAACGCT
TTGTTTTATTAGGCTCCAAATGGATACCCAAAAAGATAAAAGAAAATTGGGTAACAGAGG
TAAAAGGGATTCAAGAGTTGCATGACTTGGCCCCCAATACCACATCATCCATATAACTGA
AAGCCAAACAGTGGGAAAGCCCTACAACCACTGAACAAATGGCGCTAGTAAACTGAGCCA
GGAGAAAACGGACTGAGGCCCACTCCCATAGGAATTTTGCGAAACAGATGATGGGATGAA

TACAGGTGCAGTTTCCGTCCGAAGGTTTTGTACAGCAACAAGAGGGAAAATATAGAGGTG
CCTATTGAGTCGCAGGTCTTGCATGGTCCCGTGCTTGTTGTTGATGTTCTGGAATTAGAG
GATAAACGGGCAACAGATACCTTGGTAGTCCAGAAGAACCAAACAAGAAGATGAGGTACA
GAAAGAGGATGAAGAGGAATATGATAAAACGCCGCAGACACATCCAGCGATAGCCAGGAC
AAATTGGAGGACAAGAGGTTGGTGATGATTGGAGGTTGGGGACTGCGAATTTTGGGCTGT
GACACGTGGGTGCTCCCTGTGAAACTGTGAGAGAAGTCCCACCACGAGCCCAGACTCTGC
GGAGCTGTGGAGGATCTTTGTCAATGCTGAAAAACCCCGCCTGTAACACGAGCAGGGGTC
CTAGAACTCTGATGTTGTGTTCTCCATGTTCGGTAGGTCCCCAATCCTCGAGAAGATTAC
CATGATATGGGAGAGGCAGTAGTTGGAACAGGGTTTGCTGTTCCGGAACTGGAGCCACTG
CAGGAGCAGATAGCCCTCACTGGGATCTAGCAGAGCTTTCTGATGAAAGGTTGTGGACGC
ATTGCATGGCCTGAGGATGACTGTCTCTCAGAGGTGGAGAGATGGGAGAGGCCATTTTCT
GACTGTTGATTGGTGTGGAGGCAGAGGAGGAGTTGCTGGCACTGTTGTCAATATGCCCCA
GCCTGGAGGGCTCCACCTCCCAAAAGACCGCCGTGCGGTGGGGTGAACCCTGACCCGAAT
GCTCCTGATTCTACCTGCCAGGCCTCTGGCCAAGGGTCCTTGTTGGGGTTGAAGTCCCAA
TCTGGATTGTTTGAATTTGCCCGAAAGCCGGGTCCAACTGATGGTCGGGCAGGAATCCCA
GAGGATTGGGACAGAAAGATTCGTCCCCATGCCTTTTCGAGGTTTTGAGGAATAACCTCC
CATGCTGTAGCTCTTGTTCCCAAGAATATGGTGACCCACAAAATGATGCACTGCGTGTAG
TTTCTATATAGAATGCCAGCCTTCCAAAGGGTATGCAAATAATGCCTAGTTTTGAATAAA
TGATTAACTGAATGTTCAGGATAATAAGGTTTAATACCTTTATCCAATGGCAACATTTGG
TAAGGCTAGGAAAAATCAAGTGGCATAATTAGCTTTAATTTTTCATTTGCATTTAGAGGG
CCCACAAATTGTTGGACATCTATTACCAATGTCTTCCTGCAAATGTATACTGTGAAAAGA
GGGAGTTTGCCATCTCGTGATTGGAAGATTGCACAGTAGAAGAATAAAGCCTGCAAAGTT
TCCTCCCACCTTATGAGTCCAAGGGATACTAATGCCAGATTCCCGAGATTGAGATCTTCT
GGCGGATTACAAGGATTGAGACCTGTCTGCGAGGCGAGGAGTTCCCTTCTTCTAGGGACC
TGCCTCGCGTCGTCTAACAACAGTAGTCTCCGAAGTGTTGATAAGATAGGGGTATCTGGC
GGCTCAGGCGGGGGTGTGCGAATCCACACCCTGCCAAGGATAACGCCAAATACTCAAGAA
CCGTTTCTCTTTCCAAAAGTAAGACAAGAAATGTGAAACCACAATAGTTGTCTGATCTTA
GGCCCATACAATACTTGACATAGCTGACTACCACCCCTGGATGCTGGATCTTCCAAATTA
CTTCCTCTCTATGGATTAACTCACCCCAACACAGAATAGCTTGCCTTAGTGCTGTGTATG
GTGAGGTGAACAATGTTCCGGAGACTCTAAGGCCTCCTACAGAGCAGGAGGTATGGTGCT
GAGGAGATCTCGAATAGAAGGAAAGAAGTCAGAAAGGGCAACCCCAACAAGGACCCCTAC
GAGGCCTGGCAGGTAGGAGTGGGAGCACTGGGTCAGGGGTTCACCCCACCCGCACGGCGG
TCTTTGGGGTGGAGCCTCAGGCCTGCGGCATACACAACAGTGCCAGCAACTCCTCCTCCT
TGCCTCCACCAATCGGCAGTCAGAAAGGCAGCCTACTCCCATCTCTCCACCTCTTGAGAG
ACAGTCATCCTCTAGGCCATGCAATGGAACTCCACAACCTTCACTGCTTCCGCTAGATCC
CCAGAGGAAACACATTTCTCCTAAGGGGGTTGGTGGTTTCCAGTTCGAACAGCAAACCCT
GTTCCAACTACTGCCTCTCCCATATCGTCAATCTTCTCGAGGATTGGGGACCCTGCACTG
AACATGGAGAACACAACATCAGGATTCCTAGGACCCCTGCTCGTGTTACAGGCGGGGTTT
TTCTTGTTGACAAGAATCTCGCCAATACCGCAGAGTTCTAGACTGTGGTGGACTTTCAAT
TTTCTAGGGAGCACCCATGTCCTGGCCAAAATTCGCAGTCCCCAACCTCCAATCACTCAC
CAACCTCTTGTCCTCCAATTTGTCCTGGACACGCTTGATGTGTCTGCGGCGTTTTATCAT
ATCTTCATCCTGCTGTTGTTTCATCTTCTTGTTGGTTCTTCTGGACTACCAAGGTATAGC
GTCTGTTTGTCCTCTAATTCCAGGAACATCAACAACAAGCACGGACCATGCAAGACCTGC
ACGACTGCTCAAGGCATCCTATGTTTCCCTCTGTTGCTGCATAAAACCTTCGACGGAAAC

TGCACCTGTATTCCCATCCCATCATCCTGGGCTTTCGCAAAATTCCTATGGGAGTGGGCC
TCAGTCCGTTTCTCCTGGCTCAGTTTACTAGCGCCATTTGTTCAGTGGTTCGTAGGGCTT
TCCCCACTGTTTGGCTTTCAGTTATATGGATGATGTGGTATTGGGGCCAAGTCTGTACAA
CATCTTGAATCCCTTTACCTCTGTTACCAATTTTCTTTTTTATCTTTTGGGGTATCCATT
TGAACCCTAATAAAACCAAGCGTTGGGGCTACTCCCGAACTTCATGTATGTAATTGCCAG
TACCTACCACAGGAACATATTGTAATGAACTCAAGCAGTGTTTTCGAAAACTGCCTGTAA
TAGGGGCCTATTGATTGGAAAGTATGCTCAAAGAATTGTGGGTCTTTTTTGGGCTTTGCT
GCCCCTTTACACAATGTGGTTATCCTGCCTTACTGCCTTTGTATGGCTGTATACGGGCCA
AGCAGGCTTTCACTTCTCGCCAACTTACAAGACCTTTCGTAAACAATATCTGACCTCTTT
ACCCCGTTGCCAGGCAACGGCCAGGTCTATGCCAAGTGTTTGCTGACGCAACCCCCACTG
GTTGGGGCTGAAGAAGGGCCATCGGCGCATGCGTGAACCTTGTGGCTCCTCTGCCGATCC
ATACTGCGGAATCCTGCAGCTTGTTTTGCTCGCAGCCGGTCTGGAGCAAAACTCATCGGA
ACCGACAATCCTGTTCTCTCCTGAAATACACCTCGTTTCCATGGCTGCTCGGCTGTGCTG
CCAACTGGATCCTGCACGGACATCCTTTGTCTACGTCCCGTCGGCGCTGAATCCCGCGGA
CGACCCATCTCGGGGTCGCTTGGGAATCTACTGTCCCCTTTCTCTGTCTGCCGTTCCGGC
CGACCACGGGGCGCACTCTCTTTTACGCGGTCTCCGTTCGTGCCTTCTCATCTGCCGGAC
CGTGTGCACTTCGCTTCACTCCTGCACGTCGGCTGAGACACCGTGAACGCCAATCAGGTC
TTGCCTAAGGTCTTACATAAGAGGACTCTGGACTCTCAGCAATGTCAACGACCGACCTTG
AGGCGTACTTCAAAGACTGTGTGTTTAAAGACTGGGAGGAGCTGGGGGAGGAGATTAGGT
TAAAGGTCTTTGTACTAGGAGGCTGTAGGCATAAATTAGGTATTCTCTCAGCTTTTGAGA
ATCTCTCAGGATTCTCAAAGCTGCTCCCATCCATCTACTCATCAGTCTGCTGGAGTTGGT
CCGACAGCAAACCCATTTACCTCTCACATCTTGGCCAAATCTCCTTTTCTTTCAGTCCAG
TCTTCAGGTACATTTTCCAAAACAGAGAAAATAACTCCCTGGGGTGAAAAATATAATGAT
GTATATGGAATATTCACTAAACTGTATTCTCTGTGCTCACAGGGATCTTGTACATCAACC
GGGCAGACTGGATGGAACCCTCCAGTGAGCAGCTGGATTTTGAGAAGAGGGAAATCCAGA
CAGAGAGAGCAATCTAATCCATTTTGAGTCTGATGGCATCTTCCAACCCTGCCTAGACAC
ACTCAGAACCAGGTAGGCCAAGAACAAGGAAGATAGAGAGTTAAAGCAGCAATAAAGCCC
ATGGGTTCACACCTAGTGTCCTGCTGATGTTCTGTCCTGTCCCAAAGTCAGATGTTCCCA
GAGAGCGATCTCCAGGCATGGCAGATGGCAAAAGGGGACACATTAGGATGGGCCATGGGA
TGCCTGCTGTCAGCTCAGGCATGAAGTGAGGTGAGGAAGGGAGGAAGATTGAGTGTCCCT
TTCTGTGAGTGCCACGTGGATGTATGGGGTTGAGGCTGCGGGTTGTAGTATCAGATTACA
ATCCAGACACTCAGATCCTCTAAAGAAAGTGCTAGGCTCATATTTGTAATAAGTAACCAT
AGGCCAGATCAACAGACCTAGGTGAAAATGGCACTTATTGGTGACCTGTGGGGCCCACTC
CCTGTCCCTGGCTGTGTGTGTTCACGGCCCAGATAATGTGGGTATAATGTACCATCTGAC
AGTCCTGCCCTGCTGATATGGTGAGTTTACTTTTCCTCTTATAGGTTTGTAAAAGTAAGA
GCAACAAGCCCTGACCATCACTACAAGAGGTTAACTCACTTGACCAAGAGGATACTTACT
ACAGCAAGAATACTATCTGTGTCAGAACAGGAGCTGCCCATGTTTGGAACCTGCTTTTGT
TTTACACTTTTTATGGTTGTTGTTGCCATTTGATGCATCATTCTGTGAATCTGACACCCA
TCCCTGTAGTGGAGGCAAGAATGGGCCTCTGAGGCTGGGTGCTGTGAAGGCAATGTGATC
AGGAGCCCCATGAAGATGCCACAATTTAGGGTCAGGGATTATGTTTCCCTGAAAACTAAA
GTCCATCATTAGTTGATCTCGCCTCTTTCTTCTTGGCTTGTCAAGCATGGGATATGATTG
TGCCCAATGGTTTGCAAGCATGGAGGCCAAGTGGGAAGGAGCACACTTCCAGACTGCAGG
AAACGATACTGCAACAGGGCTCCAGGTGCTCTCATTCCATATGCCCCACTTCCACACCCT
GGAAATCCACTCAATGTTCAAAGTGAATTTTGTATACCAGTAAATACGTAACTGAACGAA

GTACA

Supplementary Table 9 Breakpoints of adjacent tissues

| ID | Sample | Viral_Transcript | Host_Gene | Total support-read | Chromoso | Position 1 | Strand 1 | Chromoso | Position 2 | Strand 2 | CytoBand |
|---|---|---|---|---|---|---|---|---|---|---|---|
| 1N | 20C227770_R303_CapNGS | HBVCgp3_X;HBVCgp4_PREC/C | OTOS(dist=29390),GPC1(dist=265615)//intergenic | 803 | chr2 | 241109465 | - | AY123041 | 1692 | + | 2q37.3 |
| 1N | 20C227770_R303_CapNGS | HBVCgp4_PREC/C | OTOS(dist=29378),GPC1(dist=265627)//intergenic | 778 | chr2 | 241109453 | - | AY123041 | 2307 | + | 2q37.3 |
| 1N | 20C227770_R303_CapNGS | HBVCgp4_PREC/C | ESPNL//intronic | 109 | chr2 | 239032139 | + | AY123041 | 1886 | - | 2q37.3 |
| 1N | 20C227770_R303_CapNGS | HBVCgp3_X//upstream | EPRS1//intronic | 7 | chr1 | 220149974 | + | AY123041 | 396 | - | 1q41 |
| 1N | 20C227770_R303_CapNGS | HBVCgp2_PRES1/PRES2/S;HBVCgp3_X; HBVCgp4_PREC/C//upstream;downstre am | FN1//intronic | 1082 | chr2 | 216296034 | - | AY123041 | 2660 | + | 2q35 |
| 1N | 20C227770_R303_CapNGS | HBVCgp3_X | FN1//intronic | 598 | chr2 | 216260792 | + | AY123041 | 1685 | - | 2q35 |
| 1N | 20C227770_R303_CapNGS | HBVCgp3_X;HBVCgp4_PREC/C | PROX1-AS1//ncRNA_intronic | 1206 | chr1 | 214071456 | + | AY123041 | 1699 | - | 1q32.3 |
| 1N | 20C227770_R303_CapNGS | HBVCgp4_PREC/C | RAPH1(dist=51102),CD28(dist=120072)//intergenic | 64 | chr2 | 204451126 | + | AY123041 | 1792 | - | 2q33.2 |
| 1N | 20C227770_R303_CapNGS | HBVCgp4_PREC/C | RAPH1//intronic | 1369 | chr2 | 204337794 | + | AY123041 | 2149 | + | 2q33.2 |
| 1N | 20C227770_R303_CapNGS | HBVCgp4_PREC/C | RAPH1//intronic | 1374 | chr2 | 204337760 | + | AY123041 | 2174 | + | 2q33.2 |
| 1N | 20C227770_R303_CapNGS | HBVCgp4_PREC/C//downstream | EDNRA(dist=8396),TMEM184C(dist=64046)//intergenic | 6 | chr4 | 148474502 | + | AY123041 | 2807 | + | 4q31.22 |
| 1N | 20C227770_R303_CapNGS | HBVCgp4_PREC/C | ZC3HAV1//UTR3 | 60 | chr7 | 138730649 | + | AY123041 | 1726 | - | 7q34 |
| 1N | 20C227770_R303_CapNGS | HBVCgp4_PREC/C | ZC3HAV1//UTR3 | 60 | chr7 | 138730647 | + | AY123041 | 1726 | - | 7q34 |
| 1N | 20C227770_R303_CapNGS | HBVCgp3_X;HBVCgp4_PREC/C | LOC101927822(dist=293313),ZFAT(dist=282068)//intergenic | 6634 | chr8 | 135207963 | + | AY123041 | 1692 | + | 8q24.22 |
| 1N | 20C227770_R303_CapNGS | HBVCgp3_X | NCOR2(dist=118172),SCARB1(dist=91073)//intergenic | 53 | chr12 | 125170330 | + | AY123041 | 1373 | + | 12q24.31 |
| 1N | 20C227770_R303_CapNGS | HBVCgp3_X;HBVCgp4_PREC/C | TLR4(dist=789709),LINC02578(dist=165227)//intergenic | 60 | chr9 | 121276722 | + | AY123041 | 1689 | + | 9q33.1 |
| 1N | 20C227770_R303_CapNGS | HBVCgp3_X | NUS1(dist=77608),SLC35F1(dist=118933)//intergenic | 29 | chr6 | 118109494 | + | AY123041 | 1617 | + | 6q22.1 |
| 1N | 20C227770_R303_CapNGS | HBVCgp3_X;HBVCgp4_PREC/C//upstrea m | PCCA//intronic | 55 | chr13 | 101035376 | + | AY123041 | 1049 | + | 13q32.3 |
| 1N | 20C227770_R303_CapNGS | HBVCgp4_PREC/C | CYP3A4//intronic | 1575 | chr7 | 99359257 | + | AY123041 | 2194 | - | 7q22.1 |
| 1N | 20C227770_R303_CapNGS | HBVCgp2_PRES1/PRES2/S;HBVCgp1_P; HBVCgp3_X;HBVCgp4_PREC/C//upstrea m;downstream | OXGR1(dist=158803),LINC00456(dist=18849)//intergenic | 2690 | chr13 | 97805787 | + | AY123041 | 2340 | + | 13q32.1 |
| 1N | 20C227770_R303_CapNGS | HBVCgp4_PREC/C | OXGR1(dist=158559),LINC00456(dist=19093)//intergenic | 2672 | chr13 | 97805543 | + | AY123041 | 1748 | + | 13q32.1 |
| 1N | 20C227770_R303_CapNGS | HBVCgp4_PREC/C//downstream | HS6ST3//intronic | 1799 | chr13 | 97127281 | - | AY123041 | 2826 | + | 13q32.1 |
| 1N | 20C227770_R303_CapNGS | HBVCgp3_X;HBVCgp4_PREC/C | TSG1(dist=1471),MANEA-DT(dist=1520163)//intergenic | 49 | chr6 | 94487809 | + | AY123041 | 1699 | - | 6q16.1 |
| 1N | 20C227770_R303_CapNGS | HBVCgp4_PREC/C//downstream | CA13//intronic | 6 | chr8 | 86163924 | + | AY123041 | 3086 | - | 8q21.2 |
| 1N | 20C227770_R303_CapNGS | HBVCgp3_X;HBVCgp4_PREC/C | ALX1(dist=54741),RASSF9(dist=444179)//intergenic | 13122 | chr12 | 85750302 | - | AY123041 | 1700 | + | 12q21.31 |
| 1N | 20C227770_R303_CapNGS | HBVCgp3_X;HBVCgp4_PREC/C | ALX1(dist=54735),RASSF9(dist=444185)//intergenic | 13240 | chr12 | 85750296 | + | AY123041 | 1700 | - | 12q21.31 |
| 1N | 20C227770_R303_CapNGS | HBVCgp3_X;HBVCgp4_PREC/C | ALX1(dist=54581),RASSF9(dist=444339)//intergenic | 13156 | chr12 | 85750142 | + | AY123041 | 1693 | - | 12q21.31 |
| 1N | 20C227770_R303_CapNGS | HBVCgp4_PREC/C | LINC01419(dist=339520),RALYL(dist=434370)//intergenic | 6 | chr8 | 84660652 | + | AY123041 | 1949 | + | 8q21.2 |
| 1N | 20C227770_R303_CapNGS | HBVCgp4_PREC/C | PHIP(dist=58459),HMGN3(dist=64532)//intergenic | 7396 | chr6 | 79846430 | + | AY123041 | 2210 | + | 6q14.1 |
| 1N | 20C227770_R303_CapNGS | HBVCgp4_PREC/C | PHIP(dist=58444),HMGN3(dist=64547)//intergenic | 7464 | chr6 | 79846415 | + | AY123041 | 2105 | + | 6q14.1 |
| 1N | 20C227770_R303_CapNGS | HBVCgp4_PREC/C | PPEF2//intronic | 267 | chr4 | 76807186 | + | AY123041 | 2149 | + | 4q21.1 |
| 1N | 20C227770_R303_CapNGS | HBVCgp3_X//upstream | IQGAP2//intronic | 7025 | chr5 | 75890202 | + | AY123041 | 683 | + | 5q13.3 |
| 1N | 20C227770_R303_CapNGS | HBVCgp3_X//upstream | IQGAP2//intronic | 7077 | chr5 | 75890198 | + | AY123041 | 683 | + | 5q13.3 |
| 1N | 20C227770_R303_CapNGS | HBVCgp3_X//upstream | ZDHHC15//UTR3 | 104 | chrX | 74592532 | + | AY123041 | 599 | + | Xq13.3 |
| 1N | 20C227770_R303_CapNGS | HBVCgp3_X//upstream | LOC101926943//ncRNA_intronic | 2953 | chr7 | 74139427 | + | AY123041 | 631 | + | 7q11.23 |
| 1N | 20C227770_R303_CapNGS | HBVCgp3_X//upstream | LOC101926943//ncRNA_intronic | 2953 | chr7 | 74139425 | + | AY123041 | 631 | + | 7q11.23 |
| 1N | 20C227770_R303_CapNGS | HBVCgp3_X//upstream | LOC101926943//ncRNA_intronic | 2953 | chr7 | 74139419 | + | AY123041 | 631 | + | 7q11.23 |
| 1N | 20C227770_R303_CapNGS | HBVCgp3_X//upstream | LOC101926943//ncRNA_intronic | 2913 | chr7 | 74139417 | + | AY123041 | 631 | + | 7q11.23 |
| 1N | 20C227770_R303_CapNGS | HBVCgp3_X//upstream | MDFIC2(dist=484030),FOXP1(dist=156149)//intergenic | 53 | chr3 | 70845819 | + | AY123041 | 372 | - | 3p13 |
| 1N | 20C227770_R303_CapNGS | HBVCgp3_X;HBVCgp4_PREC/C | C5orf64-AS1(dist=392641),KIF2A(dist=177823)//intergenic | 411 | chr5 | 61424166 | + | AY123041 | 1691 | - | 5q12.1 |
| 1N | 20C227770_R303_CapNGS | HBVCgp4_PREC/C | IL17RD(dist=1630),HESX1(dist=25891)//intergenic | 23 | chr3 | 57205975 | + | AY123041 | 1726 | - | 3p14.3 |
| 1N | 20C227770_R303_CapNGS | HBVCgp4_PREC/C | ATP6V1H//intronic | 1726 | chr8 | 54664868 | + | AY123041 | 1718 | + | 8q11.23 |
| 1N | 20C227770_R303_CapNGS | HBVCgp4_PREC/C | THSD7A//exonic//THSD7A:NM_015204:exon3:c.1271 | 3622 | chr7 | 11632881 | + | AY123041 | 1713 | - | 7p21.3 |
| 1N | 20C227770_R303_CapNGS | HBVCgp3_X//upstream | TPO(dist=17867),PXDN(dist=70349)//intergenic | 5 | chr2 | 1565310 | + | AY123041 | 598 | - | 2p25.3 |
| 4N | 20C227772_R303_CapNGS | HBVBgp3_X | WDFY1//intronic | 1436 | chr2 | 224765705 | + | AF100309 | 1819 | + | 2q36.1 |
| 4N | 20C227772_R303_CapNGS | HBVBgp3_X;HBVBgp4_C//downstream | DPP6//intronic | 4345 | chr7 | 154267486 | + | AF100309 | 2703 | - | 7q36.2 |
| 4N | 20C227772_R303_CapNGS | HBVBgp4_C | CIZ1//intronic | 14 | chr9 | 130951594 | + | AF100309 | 2339 | + | 9q34.11 |
| 4N | 20C227772_R303_CapNGS | HBVBgp4_C | CIZ1//intronic | 15 | chr9 | 130951592 | + | AF100309 | 2339 | + | 9q34.11 |
| 4N | 20C227772_R303_CapNGS | HBVBgp4_C | CIZ1//intronic | 7 | chr9 | 130951591 | + | AF100309 | 2339 | + | 9q34.11 |
| 4N | 20C227772_R303_CapNGS | HBVBgp3_X | LINC02627(dist=141926),LINC02624(dist=177906)//intergenic | 1828 | chr10 | 107722017 | + | AF100309 | 1654 | + | 10q25.1 |
| 4N | 20C227772_R303_CapNGS | HBVBgp4_C | GPC6//intronic | 5509 | chr13 | 93958662 | + | AF100309 | 1961 | + | 13q31.3 |
| 4N | 20C227772_R303_CapNGS | HBVBgp3_X;HBVBgp4_C//downstream | FAM133A(dist=547342),MIR548M(dist=803525)//intergenic | 2284 | chrX | 93514615 | + | AF100309 | 2627 | - | Xq21.33 |
| 4N | 20C227772_R303_CapNGS | HBVBgp4_C | LOC643339//ncRNA_intronic | 127 | chr12 | 93507980 | - | AF100309 | 2039 | + | 12q22 |
| 4N | 20C227772_R303_CapNGS | HBVBgp4_C | TLN2(dist=86110),TPM1(dist=112007)//intergenic | 37 | chr15 | 63222939 | + | AF100309 | 1979 | + | 15q22.2 |
| 4N | 20C227772_R303_CapNGS | HBVBgp4_C | HERPUD1//upstream | 2869 | chr16 | 56965174 | + | AF100309 | 2434 | - | 16q13 |
| 4N | 20C227772_R303_CapNGS | HBVBgp4_C | LINC01441(dist=362630),CBLN4(dist=166048)//intergenic | 95 | chr20 | 54406365 | + | AF100309 | 2025 | + | 20q13.2 |
| 4N | 20C227772_R303_CapNGS | HBVBgp3_X | TBC1D22A(dist=131622),LOC339685(dist=38357)//intergenic | 4291 | chr22 | 47702964 | + | AF100309 | 1785 | + | 22q13.31 |
| 5N | 20C227773_R303_CapNGS | HBVCgp4_PREC/C | RFTN2//intronic | 6 | chr2 | 198466966 | - | AY123041 | 1808 | + | 2q33.1 |
| 5N | 20C227773_R303_CapNGS | HBVCgp4_PREC/C | KCNT2//intronic | 598 | chr1 | 196465039 | + | AY123041 | 2219 | + | 1q31.3 |

| | | | | | | | | | | | |
|---|---|---|---|---|---|---|---|---|---|---|---|
| 5N | 20C227773_R303_CapNGS | HBVCgp4_PREC/C | DBET(dist=54817),NONE(dist=NONE)//intergenic | 7280 | chr4 | 191043836 | + | AY123041 | 1735 | + | 4q35.2 |
| 5N | 20C227773_R303_CapNGS | HBVCgp4_PREC/C | DBET(dist=54812),NONE(dist=NONE)//intergenic | 7044 | chr4 | 191043831 | + | AY123041 | 1735 | + | 4q35.2 |
| 5N | 20C227773_R303_CapNGS | HBVCgp3_X;HBVCgp4_PREC/C | LINC01037(dist=950873),BRINP3(dist=1669562)//intergenic | 230 | chr1 | 188397227 | + | AY123041 | 1699 | + | 1q31.1 |
| 5N | 20C227773_R303_CapNGS | HBVCgp4_PREC/C | KNG1//exonic//KNG1:NM_000893:exon3:c.321,KNG1:NM_001102416:exon3:c.321,KNG1:NM_001166451:exon3:c.321 | 14 | chr3 | 186440240 | - | AY123041 | 1864 | + | 3q27.3 |
| 5N | 20C227773_R303_CapNGS | HBVCgp3_X;HBVCgp4_PREC/C | KNG1//intronic | 20 | chr3 | 186440208 | + | AY123041 | 1692 | + | 3q27.3 |
| 5N | 20C227773_R303_CapNGS | HBVCgp4_PREC/C | ABCC5//intronic | 7 | chr3 | 183712035 | - | AY123041 | 1841 | + | 3q27.1 |
| 5N | 20C227773_R303_CapNGS | NONE(dist=NONE),HBVCgp3_X(dist=1153)//intergenic | FGF18(dist=54308),SMIM23(dist=273883)//intergenic | 63 | chr5 | 170938938 | + | AY123041 | 95 | - | 5q35.1 |
| 5N | 20C227773_R303_CapNGS | HBVCgp3_X;HBVCgp4_PREC/C | SERPINI1(dist=30650),LRRC77P(dist=39729)//intergenic | 8 | chr3 | 167574007 | - | AY123041 | 1691 | + | 3q26.1 |
| 5N | 20C227773_R303_CapNGS | HBVCgp3_X//upstream | GALNT13//intronic | 6 | chr2 | 155111625 | + | AY123041 | 426 | - | 2q24.1 |
| 5N | 20C227773_R303_CapNGS | HBVCgp3_X//upstream | RNF13//intronic | 6500 | chr3 | 149671888 | - | AY123041 | 381 | + | 3q25.1 |
| 5N | 20C227773_R303_CapNGS | HBVCgp3_X//upstream | RNF13//intronic | 6582 | chr3 | 149671887 | + | AY123041 | 381 | - | 3q25.1 |
| 5N | 20C227773_R303_CapNGS | NONE(dist=NONE),HBVCgp3_X(dist=1064)//intergenic | LINC02479(dist=695022),SNHG27(dist=681745)//intergenic | 21 | chr4 | 132004248 | + | AY123041 | 184 | + | 4q28.3 |
| 5N | 20C227773_R303_CapNGS | HBVCgp2_PRES1/PRES2/S;HBVCgp1_P;HBVCgp3_X;HBVCgp4_PREC/C//upstream;downstream | CCDC192//intronic | 15 | chr5 | 127092382 | + | AY123041 | 2330 | + | 5q23.2 |
| 5N | 20C227773_R303_CapNGS | HBVCgp3_X//upstream | BRINP1(dist=116682),LINC01613(dist=448943)//intergenic | 111 | chr9 | 122248395 | + | AY123041 | 490 | + | 9q33.1 |
| 5N | 20C227773_R303_CapNGS | HBVCgp4_PREC/C//downstream | LAPTM4B//intronic | 10 | chr8 | 98808980 | + | AY123041 | 2798 | - | 8q22.1 |
| 5N | 20C227773_R303_CapNGS | HBVCgp3_X//upstream | PANK1//intronic | 46 | chr10 | 91403691 | + | AY123041 | 452 | + | 10q23.31 |
| 5N | 20C227773_R303_CapNGS | HBVCgp4_PREC/C//downstream | CRTC3-AS1//ncRNA_intronic | 61 | chr15 | 91170945 | + | AY123041 | 2994 | + | 15q26.1 |
| 5N | 20C227773_R303_CapNGS | HBVCgp3_X | CCDC90B(dist=43838),DLG2(dist=124768)//intergenic | 29 | chr11 | 83041288 | + | AY123041 | 1499 | + | 11q14.1 |
| 5N | 20C227773_R303_CapNGS | HBVCgp4_PREC/C | IRAK1BP1//intronic | 9 | chr6 | 79578336 | + | AY123041 | 1867 | + | 6q14.1 |
| 5N | 20C227773_R303_CapNGS | HBVCgp3_X//upstream | ATXN7L3B(dist=352572),KCNC2(dist=142076)//intergenic | 8896 | chr12 | 75291782 | + | AY123041 | 668 | + | 12q21.1 |
| 5N | 20C227773_R303_CapNGS | HBVCgp3_X;HBVCgp4_PREC/C | SMOC1//intronic | 171 | chr14 | 70485249 | + | AY123041 | 1691 | - | 14q24.2 |
| 5N | 20C227773_R303_CapNGS | HBVCgp3_X//upstream | SLC10A1(dist=9057),LOC100506358(dist=5462)//intergenic | 427 | chr14 | 70273015 | + | AY123041 | 610 | - | 14q24.2 |
| 5N | 20C227773_R303_CapNGS | HBVCgp3_X//upstream | ETAA1(dist=314348),LINC01812(dist=69617)//intergenic | 13 | chr2 | 67953569 | + | AY123041 | 431 | - | 2p14 |
| 5N | 20C227773_R303_CapNGS | HBVCgp4_PREC/C | FUT8(dist=509782),CCDC196(dist=232468)//intergenic | 6 | chr14 | 66720621 | + | AY123041 | 1932 | - | 14q23.3 |
| 5N | 20C227773_R303_CapNGS | HBVCgp2_PRES1/PRES2/S;HBVCgp1_P;HBVCgp3_X;HBVCgp4_PREC/C//upstream;downstream | SMURF2//intronic | 107 | chr17 | 62637774 | + | AY123041 | 2403 | - | 17q24.1 |
| 5N | 20C227773_R303_CapNGS | HBVCgp3_X;HBVCgp4_PREC/C | MIR4712(dist=26474),USP8(dist=37521)//intergenic | 6 | chr15 | 50679081 | + | AY123041 | 1697 | + | 15q21.2 |
| 5N | 20C227773_R303_CapNGS | HBVCgp2_PRES1/PRES2/S;HBVCgp3_X;HBVCgp4_PREC/C//upstream;downstre[illegible] | KLHL18(dist=32602),PTPN23(dist=1603)//intergenic | 12 | chr3 | 47420908 | + | AY123041 | 2657 | - | 3p21.31 |
| 5N | 20C227773_R303_CapNGS | HBVCgp4_PREC/C | ABCG8//UTR3 | 700 | chr2 | 44110009 | + | AY123041 | 2063 | - | 2p21 |
| 5N | 20C227773_R303_CapNGS | HBVCgp3_X | POTEA(dist=604063),NONE(dist=NONE)//intergenic | 74 | chr8 | 43822391 | + | AY123041 | 1682 | - | 8p11.1 |
| 5N | 20C227773_R303_CapNGS | HBVCgp4_PREC/C | NAA16//intronic | 10 | chr13 | 41943657 | + | AY123041 | 2237 | + | 13q14.11 |
| 5N | 20C227773_R303_CapNGS | NONE(dist=NONE),HBVCgp3_X(dist=1109)//intergenic | CNTNAP3//intronic | 234 | chr9 | 39134146 | - | AY123041 | 139 | + | 9p13.1 |
| 5N | 20C227773_R303_CapNGS | HBVCgp3_X//upstream | LINC02278//ncRNA_intronic | 18 | chr4 | 38569196 | + | AY123041 | 621 | + | 4p14 |
| 5N | 20C227773_R303_CapNGS | NONE(dist=NONE),HBVCgp3_X(dist=1077)//intergenic | SIPA1L3//intronic | 2171 | chr19 | 38429625 | + | AY123041 | 171 | + | 19q13.13 |
| 5N | 20C227773_R303_CapNGS | HBVCgp3_X | DYNLT3(dist=33512),H2AP(dist=109696)//intergenic | 19969 | chrX | 37740336 | + | AY123041 | 1681 | + | Xp11.4 |
| 5N | 20C227773_R303_CapNGS | HBVCgp3_X | DYNLT3(dist=33511),H2AP(dist=109697)//intergenic | 20041 | chrX | 37740335 | + | AY123041 | 1681 | + | Xp11.4 |
| 5N | 20C227773_R303_CapNGS | HBVCgp3_X | DYNLT3(dist=33509),H2AP(dist=109699)//intergenic | 19969 | chrX | 37740333 | + | AY123041 | 1681 | + | Xp11.4 |
| 5N | 20C227773_R303_CapNGS | HBVCgp3_X | DYNLT3(dist=33507),H2AP(dist=109701)//intergenic | 19969 | chrX | 37740331 | + | AY123041 | 1681 | + | Xp11.4 |
| 5N | 20C227773_R303_CapNGS | HBVCgp3_X | DYNLT3(dist=33496),H2AP(dist=109712)//intergenic | 19969 | chrX | 37740320 | + | AY123041 | 1681 | + | Xp11.4 |
| 5N | 20C227773_R303_CapNGS | HBVCgp3_X;HBVCgp4_PREC/C | SRCIN1//intronic | 194 | chr17 | 36746437 | + | AY123041 | 1690 | + | 17q12 |
| 5N | 20C227773_R303_CapNGS | HBVCgp3_X//upstream | LARGE1(dist=326304),ISX-AS1(dist=507476)//intergenic | 45 | chr22 | 34645180 | - | AY123041 | 274 | + | 22q12.3 |
| 5N | 20C227773_R303_CapNGS | HBVCgp3_X;HBVCgp4_PREC/C | SLCO1B3-SLCO1B7;SLCO1B3//intronic | 1214 | chr12 | 21043718 | + | AY123041 | 1705 | + | 12p12.2 |
| 5N | 20C227773_R303_CapNGS | HBVCgp4_PREC/C | SGCZ//intronic | 88 | chr8 | 14406029 | + | AY123041 | 1768 | - | 8p22 |
| 5N | 20C227773_R303_CapNGS | HBVCgp4_PREC/C | CACNA1A(dist=143718),CCDC130(dist=81555)//intergenic | 11 | chr19 | 13761011 | - | AY123041 | 1759 | + | 19p13.2 |
| 5N | 20C227773_R303_CapNGS | HBVCgp3_X;HBVCgp4_PREC/C | NEDD9(dist=77877),TMEM170B(dist=77524)//intergenic | 2071 | chr6 | 11460458 | + | AY123041 | 1705 | + | 6p24.2 |
| 5N | 20C227773_R303_CapNGS | HBVCgp3_X;HBVCgp4_PREC/C | NEDD9(dist=77815),TMEM170B(dist=77586)//intergenic | 2093 | chr6 | 11460396 | + | AY123041 | 1700 | - | 6p24.2 |
| 5N | 20C227773_R303_CapNGS | HBVCgp4_PREC/C//downstream | CLNK(dist=418207),MIR572(dist=265852)//intergenic | 6 | chr4 | 11104599 | + | AY123041 | 2777 | + | 4p16.1 |
| 5N | 20C227773_R303_CapNGS | HBVCgp4_PREC/C | CARHSP1(dist=21199),USP7(dist=1883)//intergenic | 7 | chr16 | 8984068 | + | AY123041 | 2245 | + | 16p13.2 |
| 5N | 20C227773_R303_CapNGS | HBVCgp4_PREC/C | SNTG2//intronic | 7 | chr2 | 959300 | - | AY123041 | 2086 | + | 2p25.3 |
| 6N | 20C227774_R303_CapNGS | HBVCgp3_X//upstream | MIR4300HG//ncRNA_intronic | 45 | chr11 | 82039926 | + | AY123041 | 550 | - | 11q14.1 |
| 6N | 20C227774_R303_CapNGS | HBVCgp3_X//upstream | DEDD2//intronic | 22 | chr19 | 42709538 | + | AY123041 | 639 | + | 19q13.2 |
| 6N | 20C227774_R303_CapNGS | HBVCgp2_PRES1/PRES2/S;HBVCgp3_X;HBVCgp4_PREC/C//upstream;downstre[illegible] | BNC2(dist=195135),CNTLN(dist=69235)//intergenic | 14 | chr9 | 17065803 | + | AY123041 | 2693 | + | 9p22.2 |
| 6N | 20C227774_R303_CapNGS | HBVCgp4_PREC/C | NONE(dist=NONE),GYG2P1(dist=697722)//intergenic | 83308 | chrY | 13820193 | - | AY123041 | 2214 | + | Yq11.21 |
| 6N | 20C227774_R303_CapNGS | HBVCgp4_PREC/C | NONE(dist=NONE),GYG2P1(dist=697728)//intergenic | 83308 | chrY | 13820187 | - | AY123041 | 2214 | + | Yq11.21 |
| 6N | 20C227774_R303_CapNGS | HBVCgp4_PREC/C | NONE(dist=NONE),GYG2P1(dist=697729)//intergenic | 83059 | chrY | 13820186 | + | AY123041 | 2213 | - | Yq11.21 |

| | | | | | | | | | | | |
|---|---|---|---|---|---|---|---|---|---|---|---|
| 8N | 20C227775_R303_CapNGS | HBVCgp4_PREC/C | FN1//intronic | 13371 | chr2 | 216254888 | - | AY123041 | 2054 | + | 2q35 |
| 8N | 20C227775_R303_CapNGS | HBVCgp4_PREC/C | FN1//intronic | 13276 | chr2 | 216254887 | - | AY123041 | 2054 | + | 2q35 |
| 8N | 20C227775_R303_CapNGS | HBVCgp3_X;HBVCgp4_PREC/C | CPS1(dist=42800),ERBB4(dist=653811)//intergenic | 807 | chr2 | 211586631 | + | AY123041 | 1690 | + | 2q34 |
| 8N | 20C227775_R303_CapNGS | NONE(dist=NONE),HBVCgp3_X(dist=1101)//intergenic | CEP63//intronic | 6 | chr3 | 134225713 | + | AY123041 | 147 | + | 3q22.2 |
| 8N | 20C227775_R303_CapNGS | HBVCgp3_X | MIR2054(dist=1815607),INTU(dist=310043)//intergenic | 6343 | chr4 | 128244069 | + | AY123041 | 1496 | + | 4q28.1 |
| 8N | 20C227775_R303_CapNGS | NONE(dist=NONE),HBVCgp3_X(dist=1144)//intergenic | LIN52(dist=3412),VSX2(dist=34544)//intergenic | 1301 | chr14 | 74671608 | + | AY123041 | 104 | - | 14q24.3 |
| 8N | 20C227775_R303_CapNGS | HBVCgp2_PRES1/PRES2/S,HBVCgp3_X,HBVCgp4_PREC/C//upstream;downstream | LINC01626(dist=361883),RIMS1(dist=65948)//intergenic | 42 | chr6 | 72530458 | + | AY123041 | 2604 | + | 6q13 |
| 8N | 20C227775_R303_CapNGS | HBVCgp4_PREC/C | ARID5B//intronic | 5 | chr10 | 63766170 | + | AY123041 | 2319 | - | 10q21.2 |
| 8N | 20C227775_R303_CapNGS | HBVCgp3_X;HBVCgp4_PREC/C//upstream | PCDH15//intronic | 10 | chr10 | 56979171 | + | AY123041 | 744 | - | 10q21.1 |
| 8N | 20C227775_R303_CapNGS | HBVCgp4_PREC/C | GHR//intronic | 13115 | chr5 | 42552293 | + | AY123041 | 1739 | + | 5p12 |
| 8N | 20C227775_R303_CapNGS | HBVCgp3_X//upstream | LINC02104(dist=175625),LINC00603(dist=351958)//intergenic | 515 | chr5 | 39700435 | + | AY123041 | 509 | + | 5p13.1 |
| 9N | 20C227776_R303_CapNGS | HBVCgp4_PREC/C | PDE1A//UTR3 | 21 | chr2 | 183004868 | - | AY123041 | 1906 | + | 2q32.1 |
| 9N | 20C227776_R303_CapNGS | HBVCgp4_PREC/C//downstream | LINC01934//ncRNA_intronic | 119 | chr2 | 182257352 | + | AY123041 | 2923 | + | 2q31.3 |
| 9N | 20C227776_R303_CapNGS | HBVCgp3_X//upstream | LOC102546299//ncRNA_intronic | 528 | chr5 | 163922977 | - | AY123041 | 324 | + | 5q34 |
| 9N | 20C227776_R303_CapNGS | HBVCgp4_PREC/C | TPK1(dist=1243723),CNTNAP2(dist=694494)//intergenic | 196 | chr7 | 145776869 | + | AY123041 | 2086 | + | 7q35 |
| 9N | 20C227776_R303_CapNGS | HBVCgp4_PREC/C//downstream | LINC01087//ncRNA_intronic | 210 | chr2 | 132401830 | + | AY123041 | 3062 | + | 2q21.1 |
| 9N | 20C227776_R303_CapNGS | HBVCgp3_X;HBVCgp4_PREC/C | SNCAIP//intronic | 1116 | chr5 | 121770344 | + | AY123041 | 1707 | - | 5q23.2 |
| 9N | 20C227776_R303_CapNGS | HBVCgp3_X;HBVCgp4_PREC/C | SNCAIP//intronic | 1107 | chr5 | 121767938 | + | AY123041 | 1699 | - | 5q23.2 |
| 9N | 20C227776_R303_CapNGS | HBVCgp3_X;HBVCgp4_PREC/C | HSD17B4//intronic | 176 | chr5 | 118844416 | + | AY123041 | 1700 | + | 5q23.1 |
| 9N | 20C227776_R303_CapNGS | HBVCgp3_X;HBVCgp4_PREC/C | HSD17B4//intronic | 175 | chr5 | 118844415 | + | AY123041 | 1700 | + | 5q23.1 |
| 9N | 20C227776_R303_CapNGS | HBVCgp3_X;HBVCgp4_PREC/C | HSD17B4//intronic | 304 | chr5 | 118844412 | + | AY123041 | 1700 | + | 5q23.1 |
| 9N | 20C227776_R303_CapNGS | HBVCgp3_X;HBVCgp4_PREC/C | HSD17B4//intronic | 175 | chr5 | 118844411 | + | AY123041 | 1700 | + | 5q23.1 |
| 9N | 20C227776_R303_CapNGS | HBVCgp3_X//upstream | DPP10(dist=1611534),DDX18(dist=357379)//intergenic | 455 | chr2 | 118214888 | + | AY123041 | 658 | + | 2q14.1 |
| 9N | 20C227776_R303_CapNGS | HBVCgp2_PRES1/PRES2/S,HBVCgp3_X,HBVCgp4_PREC/C//upstream;downstream | LOC101928358(dist=315695),GUCY2F(dist=318307)//intergenic | 93 | chrX | 108297828 | - | AY123041 | 2632 | + | Xq22.3 |
| 9N | 20C227776_R303_CapNGS | HBVCgp3_X | ATXN7L1//intronic | 2750 | chr7 | 105324004 | + | AY123041 | 1591 | - | 7q22.3 |
| 9N | 20C227776_R303_CapNGS | HBVCgp3_X//upstream | NUDT12(dist=405562),RAB9BP1(dist=1131123)//intergenic | 330 | chr5 | 103304052 | - | AY123041 | 613 | + | 5q21.2 |
| 9N | 20C227776_R303_CapNGS | HBVCgp3_X;HBVCgp4_PREC/C | OLFM3(dist=486860),COL11A1(dist=392373)//intergenic | 7 | chr1 | 102949650 | + | AY123041 | 1695 | - | 1p21.1 |
| 9N | 20C227776_R303_CapNGS | HBVCgp3_X//upstream | ZKSCAN1//intronic | 10 | chr7 | 99642241 | + | AY123041 | 497 | + | 7q22.1 |
| 9N | 20C227776_R303_CapNGS | HBVCgp3_X | AOPEP//intronic | 20 | chr9 | 97682456 | + | AY123041 | 1677 | + | 9q22.32 |
| 9N | 20C227776_R303_CapNGS | HBVCgp2_PRES1/PRES2/S,HBVCgp3_X,HBVCgp4_PREC/C//upstream;downstream | DIAPH2(dist=469574),NONE(dist=NONE)//intergenic | 259 | chrX | 97329569 | + | AY123041 | 2606 | - | Xq21.33 |
| 9N | 20C227776_R303_CapNGS | HBVCgp4_PREC/C//downstream | DIAPH2(dist=424903),NONE(dist=NONE)//intergenic | 13 | chrX | 97284898 | + | AY123041 | 2855 | + | Xq21.33 |
| 9N | 20C227776_R303_CapNGS | HBVCgp4_PREC/C | MAP3K7(dist=31500),MIR4643(dist=903087)//intergenic | 144 | chr6 | 91328291 | + | AY123041 | 1921 | - | 6q15 |
| 9N | 20C227776_R303_CapNGS | NONE(dist=NONE),HBVCgp3_X(dist=1085)//intergenic | ZMIZ1//intronic | 8 | chr10 | 80849962 | + | AY123041 | 163 | + | 10q22.3 |
| 9N | 20C227776_R303_CapNGS | HBVCgp3_X | TENM4(dist=1025263),LINC02720(dist=284907)//intergenic | 14 | chr11 | 80177337 | + | AY123041 | 1686 | + | 11q14.1 |
| 9N | 20C227776_R303_CapNGS | HBVCgp3_X | SDAD1//intronic | 8 | chr4 | 76880249 | - | AY123041 | 1625 | + | 4q21.1 |
| 9N | 20C227776_R303_CapNGS | HBVCgp4_PREC/C | NCOA2//exonic//NCOA2:NM_001321711:exon13:c.2646,NCOA2:NM_001321712:exon13:c.2646,NCOA2:NM_001321713:exon13:c.2391,NCOA2:NM_001321703:exon14:c.2853,NCOA2:NM_001321707:exon14:c.2853,NCOA2:NM_006540:exon14:c.2853 | 9 | chr8 | 71053594 | + | AY123041 | 1723 | - | 8q13.3 |
| 9N | 20C227776_R303_CapNGS | HBVCgp3_X | ADGRL3-AS1(dist=1989587),TECRL(dist=124948)//intergenic | 6 | chr4 | 65017070 | + | AY123041 | 1619 | + | 4q13.1 |
| 9N | 20C227776_R303_CapNGS | HBVCgp3_X//upstream | SLC38A6(dist=48589),TMEM30B(dist=145049)//intergenic | 423 | chr14 | 61599040 | - | AY123041 | 640 | + | 14q23.1 |
| 9N | 20C227776_R303_CapNGS | HBVCgp3_X | PDE4D//intronic | 409 | chr5 | 59710503 | - | AY123041 | 1685 | + | 5q12.1 |
| 9N | 20C227776_R303_CapNGS | HBVCgp4_PREC/C | LOC101927620(dist=436312),MIR5580(dist=354587)//intergenic | 6005 | chr14 | 54060558 | + | AY123041 | 1794 | + | 14q22.1 |
| 9N | 20C227776_R303_CapNGS | HBVCgp3_X | VCPKMT;SOS2//upstream;downstream | 18 | chr14 | 50583299 | + | AY123041 | 1587 | - | 14q21.3 |
| 9N | 20C227776_R303_CapNGS | HBVCgp3_X;HBVCgp4_PREC/C | DAG1(dist=8235),BSN-DT(dist=5453)//intergenic | 26 | chr3 | 49581286 | + | AY123041 | 1700 | + | 3p21.31 |
| 9N | 20C227776_R303_CapNGS | HBVCgp3_X;HBVCgp4_PREC/C | TRABD2B//intronic | 538 | chr1 | 48407648 | + | AY123041 | 1700 | - | 1p33 |
| 9N | 20C227776_R303_CapNGS | HBVCgp3_X;HBVCgp4_PREC/C | LRFN5//intronic | 626 | chr14 | 42311494 | - | AY123041 | 1710 | + | 14q21.1 |
| 9N | 20C227776_R303_CapNGS | HBVCgp3_X;HBVCgp4_PREC/C | ULK4(dist=19486),TRAK1(dist=105611)//intergenic | 28 | chr3 | 42023081 | + | AY123041 | 1688 | - | 3p22.1 |
| 9N | 20C227776_R303_CapNGS | HBVCgp3_X | CTDSPL//exonic//CTDSPL:NM_001008392:exon2:c.90,CTDSPL:NM_005808:exon2:c.90 | 186 | chr3 | 37988558 | - | AY123041 | 1671 | + | 3p22.2 |
| 9N | 20C227776_R303_CapNGS | HBVCgp3_X;HBVCgp4_PREC/C | ARAP2//intronic | 316 | chr4 | 36154104 | + | AY123041 | 1698 | - | 4p14 |
| 9N | 20C227776_R303_CapNGS | HBVCgp3_X;HBVCgp4_PREC/C | LINC00457(dist=59698),NBEA(dist=241904)//intergenic | 65 | chr13 | 35274520 | + | AY123041 | 1702 | + | 13q13.2 |
| 9N | 20C227776_R303_CapNGS | HBVCgp4_PREC/C | CHMP4B(dist=90355),RALY-AS1(dist=47767)//intergenic | 11 | chr20 | 32532527 | + | AY123041 | 1838 | + | 20q11.22 |
| 9N | 20C227776_R303_CapNGS | HBVCgp4_PREC/C//downstream | ELAVL2(dist=276247),IZUMO3(dist=416104)//intergenic | 7 | chr9 | 24126846 | + | AY123041 | 3006 | - | 9p21.3 |
| 9N | 20C227776_R303_CapNGS | HBVCgp4_PREC/C//downstream | RALGAPA2(dist=239279),LINC00237(dist=135679)//intergenic | 5 | chr20 | 20932566 | + | AY123041 | 2759 | + | 20p11.23 |
| 9N | 20C227776_R303_CapNGS | HBVCgp3_X | MAU2//UTR3 | 5 | chr19 | 19468714 | + | AY123041 | 1555 | + | 19p13.11 |
| 9N | 20C227776_R303_CapNGS | NONE(dist=NONE),HBVCgp3_X(dist=1035)//intergenic | IGSF21(dist=48971),KLHDC7A(dist=53476)//intergenic | 15 | chr1 | 18753948 | + | AY123041 | 213 | - | 1p36.13 |

| | | | | | | | | | | | |
|---|---|---|---|---|---|---|---|---|---|---|---|
| 9N | 20C227776_R303_CapNGS | HBVCgp4_PREC/C//downstream | MYO15A//intronic | 179 | chr17 | 18080584 | + | AY123041 | 2729 | - | 17p11.2 |
| 9N | 20C227776_R303_CapNGS | HBVCgp3_X | PARN//intronic | 10412 | chr16 | 14651472 | + | AY123041 | 1681 | + | 16p13.12 |
| 9N | 20C227776_R303_CapNGS | HBVCgp3_X | PARN//intronic | 10418 | chr16 | 14651467 | + | AY123041 | 1681 | + | 16p13.12 |
| 9N | 20C227776_R303_CapNGS | HBVCgp3_X | PARN//intronic | 11245 | chr16 | 14651436 | + | AY123041 | 1501 | + | 16p13.12 |
| 9N | 20C227776_R303_CapNGS | HBVCgp3_X | PARN//intronic | 11276 | chr16 | 14651433 | + | AY123041 | 1501 | + | 16p13.12 |
| 9N | 20C227776_R303_CapNGS | HBVCgp3_X | PARN//intronic | 11249 | chr16 | 14651432 | + | AY123041 | 1501 | + | 16p13.12 |
| 9N | 20C227776_R303_CapNGS | HBVCgp3_X | PARN//intronic | 11247 | chr16 | 14651431 | + | AY123041 | 1501 | + | 16p13.12 |
| 9N | 20C227776_R303_CapNGS | HBVCgp3_X | PARN//intronic | 11242 | chr16 | 14651430 | + | AY123041 | 1501 | + | 16p13.12 |
| 9N | 20C227776_R303_CapNGS | HBVCgp3_X | PARN//intronic | 11242 | chr16 | 14651428 | + | AY123041 | 1501 | + | 16p13.12 |
| 9N | 20C227776_R303_CapNGS | HBVCgp3_X | PARN//intronic | 11237 | chr16 | 14651427 | + | AY123041 | 1501 | + | 16p13.12 |
| 9N | 20C227776_R303_CapNGS | HBVCgp3_X | PARN//intronic | 11236 | chr16 | 14651426 | + | AY123041 | 1501 | + | 16p13.12 |
| 9N | 20C227776_R303_CapNGS | HBVCgp3_X | MACROD2//intronic | 1816 | chr20 | 14285671 | - | AY123041 | 1586 | + | 20p12.1 |
| 9N | 20C227776_R303_CapNGS | HBVCgp3_X;HBVCgp4_PREC/C | CIITA//intronic | 105 | chr16 | 10989654 | - | AY123041 | 1695 | + | 16p13.13 |
| 9N | 20C227776_R303_CapNGS | HBVCgp4_PREC/C | MIR10396A(dist=11466),TEKT4P2(dist=69225)//intergenic | 13 | chr21 | 9837964 | + | AY123041 | 2073 | - | 21p11.2 |
| 9N | 20C227776_R303_CapNGS | HBVCgp3_X//upstream | PAK5(dist=10716),PARAL1(dist=136541)//intergenic | 153 | chr20 | 9830440 | + | AY123041 | 565 | - | 20p12.2 |
| 9N | 20C227776_R303_CapNGS | HBVCgp3_X | LINC02645(dist=170095),LOC101927824(dist=380942)//interge | 8 | chr10 | 2713749 | + | AY123041 | 1402 | + | 10p15.3 |
| 9N | 20C227776_R303_CapNGS | HBVCgp3_X;HBVCgp4_PREC/C | CTSD(dist=46626),SYT8(dist=23874)//intergenic | 7 | chr11 | 1831783 | + | AY123041 | 1706 | - | 11p15.5 |
| 9N | 20C227776_R303_CapNGS | HBVCgp3_X;HBVCgp4_PREC/C | DNAAF5(dist=9425),SUN1(dist=19657)//intergenic | 821 | chr7 | 835537 | + | AY123041 | 1688 | + | 7p22.3 |
| 9N | 20C227776_R303_CapNGS | HBVCgp3_X//upstream | GZMM(dist=11023),BSG(dist=10338)//intergenic | 5 | chr19 | 560945 | + | AY123041 | 585 | - | 19p13.3 |
| 10N | 20C227777_R303_CapNGS | HBVBgp2_S | XXYLT1//intronic | 53 | chr3 | 194865300 | + | AF100309 | 270 | + | 3q29 |
| 10N | 20C227777_R303_CapNGS | HBVBgp3_X | LYPD6(dist=7442),MMADHC(dist=88050)//intergenic | 649 | chr2 | 150338101 | - | AF100309 | 1815 | + | 2q23.2 |
| 10N | 20C227777_R303_CapNGS | HBVBgp2_S | LINC02432(dist=267347),IL15(dist=36618)//intergenic | 32 | chr4 | 142521118 | + | AF100309 | 186 | + | 4q31.21 |
| 10N | 20C227777_R303_CapNGS | HBVBgp4_C | WSPAR(dist=15380),C5orf15(dist=22861)//intergenic | 9983 | chr5 | 133268340 | + | AF100309 | 2362 | + | 5q31.1 |
| 10N | 20C227777_R303_CapNGS | HBVBgp3_X | WSPAR(dist=15360),C5orf15(dist=22881)//intergenic | 9949 | chr5 | 133268320 | + | AF100309 | 1818 | + | 5q31.1 |
| 10N | 20C227777_R303_CapNGS | HBVBgp4_C | LINC02615(dist=147709),JADE1(dist=142595)//intergenic | 24 | chr4 | 129588260 | - | AF100309 | 2400 | + | 4q28.2 |
| 10N | 20C227777_R303_CapNGS | HBVBgp3_X | KCND2//intronic | 9260 | chr7 | 120276867 | + | AF100309 | 1826 | - | 7q31.31 |
| 10N | 20C227777_R303_CapNGS | HBVBgp4_C | KCND2//intronic | 9267 | chr7 | 120276864 | - | AF100309 | 2163 | + | 7q31.31 |
| 10N | 20C227777_R303_CapNGS | HBVBgp4_C | KCND2//intronic | 9289 | chr7 | 120276863 | + | AF100309 | 2163 | - | 7q31.31 |
| 10N | 20C227777_R303_CapNGS | HBVBgp3_X | LOC101927190(dist=27546),SEMA6A(dist=41438)//intergenic | 2219 | chr5 | 115737813 | + | AF100309 | 1821 | - | 5q23.1 |
| 10N | 20C227777_R303_CapNGS | HBVBgp3_X | LOC101927190(dist=27535),SEMA6A(dist=41449)//intergenic | 2201 | chr5 | 115737802 | + | AF100309 | 1800 | - | 5q23.1 |
| 10N | 20C227777_R303_CapNGS | HBVBgp4_C | ADD3-AS1//ncRNA_intronic | 1962 | chr10 | 111714993 | - | AF100309 | 2195 | + | 10q25.1 |
| 10N | 20C227777_R303_CapNGS | HBVBgp3_X | TRHR(dist=61027),NUDCD1(dist=58327)//intergenic | 4360 | chr8 | 110194821 | + | AF100309 | 1809 | + | 8q23.1 |
| 10N | 20C227777_R303_CapNGS | HBVBgp3_X | TRHR(dist=60931),NUDCD1(dist=58423)//intergenic | 4380 | chr8 | 110194725 | + | AF100309 | 1821 | + | 8q23.1 |
| 10N | 20C227777_R303_CapNGS | HBVBgp2_S | GRIK2(dist=1826296),HACE1(dist=831715)//intergenic | 2560 | chr6 | 104344254 | + | AF100309 | 169 | - | 6q16.3 |
| 10N | 20C227777_R303_CapNGS | HBVBgp2_S | GRIK2(dist=1826295),HACE1(dist=831716)//intergenic | 2556 | chr6 | 104344253 | - | AF100309 | 169 | + | 6q16.3 |
| 10N | 20C227777_R303_CapNGS | HBVBgp2_S | GRIK2(dist=1826293),HACE1(dist=831718)//intergenic | 2556 | chr6 | 104344251 | - | AF100309 | 169 | + | 6q16.3 |
| 10N | 20C227777_R303_CapNGS | HBVBgp3_X | SUFU//intronic | 7 | chr10 | 104270635 | + | AF100309 | 1747 | + | 10q24.32 |
| 10N | 20C227777_R303_CapNGS | HBVBgp3_X | KIAA0825//intronic | 4518 | chr5 | 93596102 | - | AF100309 | 1779 | + | 5q15 |
| 10N | 20C227777_R303_CapNGS | HBVBgp2_S//upstream | HFM1//intronic | 2256 | chr1 | 91853147 | + | AF100309 | 71 | - | 1p22.2 |
| 10N | 20C227777_R303_CapNGS | HBVBgp2_S | ABCB4//intronic | 1263 | chr7 | 87091355 | + | AF100309 | 287 | + | 7q21.12 |
| 10N | 20C227777_R303_CapNGS | HBVBgp3_X | ABCB4//intronic | 1265 | chr7 | 87091348 | + | AF100309 | 1549 | + | 7q21.12 |
| 10N | 20C227777_R303_CapNGS | HBVBgp4_C | BORA//intronic | 9 | chr13 | 73307870 | + | AF100309 | 2004 | + | 13q22.1 |
| 10N | 20C227777_R303_CapNGS | HBVBgp3_X | NUPR2(dist=210673),LOC650226(dist=96614)//intergenic | 420 | chr7 | 56394783 | + | AF100309 | 1825 | + | 7p11.2 |
| 10N | 20C227777_R303_CapNGS | HBVBgp3_X;HBVBgp4_C;HBVBgp2_S//upstream;downstream | MIR5007(dist=293445),PRR20B(dist=1672924)//intergenic | 8 | chr13 | 56042128 | + | AF100309 | 1249 | + | 13q21.1 |
| 10N | 20C227777_R303_CapNGS | HBVBgp3_X | MIR7975(dist=2470),TNNT1(dist=6936)//intergenic | 36 | chr19 | 55637130 | + | AF100309 | 1746 | + | 19q13.42 |
| 10N | 20C227777_R303_CapNGS | HBVBgp3_X | PFKFB1//intronic | 25 | chrX | 54988404 | + | AF100309 | 1826 | + | Xp11.21 |
| 10N | 20C227777_R303_CapNGS | HBVBgp4_C//downstream | LOC257396(dist=359729),FST(dist=5774)//intergenic | 34 | chr5 | 52770685 | + | AF100309 | 2849 | + | 5q11.2 |
| 10N | 20C227777_R303_CapNGS | HBVBgp3_X;HBVBgp4_C//downstream | NRXN1//intronic | 12 | chr2 | 50689714 | - | AF100309 | 2776 | + | 2p16.3 |
| 10N | 20C227777_R303_CapNGS | HBVBgp3_X | LINC00648(dist=1694442),RPS29(dist=79047)//intergenic | 3367 | chr14 | 49958659 | + | AF100309 | 1823 | - | 14q21.3 |
| 10N | 20C227777_R303_CapNGS | HBVBgp3_X | LINC00648(dist=1694439),RPS29(dist=79050)//intergenic | 3383 | chr14 | 49958656 | + | AF100309 | 1821 | - | 14q21.3 |
| 10N | 20C227777_R303_CapNGS | HBVBgp3_X | LINC00648(dist=1694429),RPS29(dist=79060)//intergenic | 3356 | chr14 | 49958646 | - | AF100309 | 1821 | + | 14q21.3 |
| 10N | 20C227777_R303_CapNGS | HBVBgp3_X | PKD1L1//intronic | 634 | chr7 | 47919450 | + | AF100309 | 1746 | + | 7p12.3 |
| 10N | 20C227777_R303_CapNGS | HBVBgp4_C | RAMP3(dist=167666),ADCY1(dist=222224)//intergenic | 5 | chr7 | 45391515 | - | AF100309 | 2405 | + | 7p13 |
| 10N | 20C227777_R303_CapNGS | HBVBgp3_X | ATP6V0A1//intronic | 43 | chr17 | 40643425 | + | AF100309 | 1746 | - | 17q21.2 |
| 10N | 20C227777_R303_CapNGS | HBVBgp2_S | RALA//intronic | 7 | chr7 | 39697225 | + | AF100309 | 601 | + | 7p14.1 |
| 10N | 20C227777_R303_CapNGS | HBVBgp3_X | LINC01399//ncRNA_intronic | 41 | chr22 | 35559994 | - | AF100309 | 1832 | + | 22q12.3 |
| 10N | 20C227777_R303_CapNGS | HBVBgp4_C | DCTN6(dist=148879),RBPMS-AS1(dist=49601)//intergenic | 1691 | chr8 | 30190034 | + | AF100309 | 2409 | + | 8p12 |
| 10N | 20C227777_R303_CapNGS | HBVBgp4_C//downstream | FAR2//intronic | 1245 | chr12 | 29405294 | + | AF100309 | 2923 | - | 12p11.22 |
| 10N | 20C227777_R303_CapNGS | HBVBgp2_S | OCA2//intronic | 30 | chr15 | 28235864 | + | AF100309 | 740 | + | 15q13.1 |
| 10N | 20C227777_R303_CapNGS | HBVBgp3_X | LINC02211(dist=1356929),CDH9(dist=221388)//intergenic | 3714 | chr5 | 26659318 | + | AF100309 | 1827 | + | 5p14.1 |
| 10N | 20C227777_R303_CapNGS | HBVBgp1_P;HBVBgp3_X;HBVBgp4_C//downstream | LINC02211(dist=1356907),CDH9(dist=221410)//intergenic | 3714 | chr5 | 26659296 | + | AF100309 | 2478 | + | 5p14.1 |
| 10N | 20C227777_R303_CapNGS | HBVBgp4_C | LINC01692(dist=65231),LINC00158(dist=262846)//intergenic | 2202 | chr21 | 26495287 | + | AF100309 | 2107 | + | 21q21.2 |
| 10N | 20C227777_R303_CapNGS | HBVBgp4_C | NLGN4Y(dist=1958279),FAM41AY1(dist=697026)//intergenic | 1121 | chrY | 18915812 | - | AF100309 | 1941 | + | Yq11.221 |

| | | | | | | | | | | | |
|---|---|---|---|---|---|---|---|---|---|---|---|
| 10N | 20C227777_R303_CapNGS | HBVBgp3_X | PLEKHA7//intronic | 13 | chr11 | 17018402 | + | AF100309 | 1746 | + | 11p15.1 |
| 10N | 20C227777_R303_CapNGS | HBVBgp4_C | MSR1(dist=77636),FGF20(dist=721754)//intergenic | 1414 | chr8 | 16127936 | + | AF100309 | 1932 | + | 8p22 |
| 10N | 20C227777_R303_CapNGS | HBVBgp1_P;HBVBgp4_C;HBVBgp3_X//upstream;downstream | MSR1(dist=77610),FGF20(dist=721780)//intergenic | 1416 | chr8 | 16127910 | + | AF100309 | 1842 | + | 8p22 |
| 10N | 20C227777_R303_CapNGS | HBVBgp3_X | ZNF286A(dist=2284),TBC1D26(dist=9207)//intergenic | 16 | chr17 | 15626384 | + | AF100309 | 1746 | + | 17p12 |
| 10N | 20C227777_R303_CapNGS | HBVBgp1_P;HBVBgp4_C;HBVBgp3_X//upstream;downstream | FREM1(dist=65673),LOC389705(dist=16659)//intergenic | 3165 | chr9 | 14976666 | - | AF100309 | 1858 | + | 9p22.3 |
| 10N | 20C227777_R303_CapNGS | HBVBgp3_X | FREM1(dist=65644),LOC389705(dist=16688)//intergenic | 3249 | chr9 | 14976637 | + | AF100309 | 1827 | + | 9p22.3 |
| 10N | 20C227777_R303_CapNGS | HBVBgp3_X | LRATD1(dist=27265),NBAS(dist=499610)//intergenic | 2527 | chr2 | 14807430 | - | AF100309 | 1791 | + | 2p24.3 |
| 10N | 20C227777_R303_CapNGS | HBVBgp4_C | SSU72//intronic | 1500 | chr1 | 1487609 | - | AF100309 | 2335 | + | 1p36.33 |
| 12N | 20C227778_R303_CapNGS | HBVCgp4_PREC/C | LINC01206(dist=251922),LINC01994(dist=184372)//intergenic | 1465 | chr3 | 181980386 | - | AY123041 | 2238 | + | 3q26.33 |
| 12N | 20C227778_R303_CapNGS | HBVCgp3_X;HBVCgp4_PREC/C//upstream | CTU2//intronic | 23 | chr16 | 88779454 | + | AY123041 | 720 | + | 16q24.3 |
| 12N | 20C227778_R303_CapNGS | HBVCgp2_PRES1/PRES2/S;HBVCgp3_X; HBVCgp4_PREC/C//upstream;downstream | CILP(dist=1040),PARP16(dist=45586)//intergenic | 12 | chr15 | 65504851 | + | AY123041 | 2678 | + | 15q22.31 |
| 12N | 20C227778_R303_CapNGS | HBVCgp4_PREC/C | GRIK1(dist=186910),CLDN17(dist=38605)//intergenic | 643 | chr21 | 31499280 | - | AY123041 | 1841 | + | 21q21.3 |
| 12N | 20C227778_R303_CapNGS | HBVCgp4_PREC/C | GRIK1(dist=186905),CLDN17(dist=38610)//intergenic | 642 | chr21 | 31499275 | - | AY123041 | 1841 | + | 21q21.3 |
| 12N | 20C227778_R303_CapNGS | HBVCgp3_X//upstream | SCNN1G//intronic | 1188 | chr16 | 23200105 | + | AY123041 | 289 | - | 16p12.2 |
| 12N | 20C227778_R303_CapNGS | HBVCgp4_PREC/C | GNG7//intronic | 14 | chr19 | 2667038 | + | AY123041 | 1738 | - | 19p13.3 |
| 12N | 20C227778_R303_CapNGS | HBVCgp4_PREC/C//downstream | GMDS-DT//downstream | 5 | chr6 | 2414738 | + | AY123041 | 2757 | + | 6p25.2 |
| 13N | 20C227779_R303_CapNGS | HBVCgp3_X | BCHE//intronic | 13 | chr3 | 165494730 | + | AY123041 | 1429 | + | 3q26.1 |
| 13N | 20C227779_R303_CapNGS | HBVCgp4_PREC/C | C3orf52//intronic | 6 | chr3 | 111834635 | + | AY123041 | 2279 | - | 3q13.2 |
| 13N | 20C227779_R303_CapNGS | HBVCgp4_PREC/C | CDK14//intronic | 9 | chr7 | 90561778 | + | AY123041 | 2163 | - | 7q21.13 |
| 13N | 20C227779_R303_CapNGS | HBVCgp4_PREC/C//downstream | MIR5708(dist=208685),ZBTB10(dist=35435)//intergenic | 5 | chr8 | 81362393 | + | AY123041 | 3073 | + | 8q21.13 |
| 13N | 20C227779_R303_CapNGS | HBVCgp4_PREC/C | HTR1B(dist=137331),MEI4(dist=49452)//intergenic | 860 | chr6 | 78310539 | + | AY123041 | 2299 | + | 6q14.1 |
| 13N | 20C227779_R303_CapNGS | HBVCgp3_X//upstream | CTCFL(dist=1427),PCK1(dist=34031)//intergenic | 824 | chr20 | 56102135 | - | AY123041 | 395 | + | 20q13.31 |
| 13N | 20C227779_R303_CapNGS | HBVCgp3_X;HBVCgp4_PREC/C//upstream | CELF4//intronic | 6 | chr18 | 35012464 | + | AY123041 | 924 | + | 18q12.2 |
| 13N | 20C227779_R303_CapNGS | NONE(dist=NONE),HBVCgp3_X(dist=1058)//intergenic | NBPF3//intronic | 5 | chr1 | 21782039 | + | AY123041 | 190 | - | 1p36.12 |
| 14N | 20C227780_R303_CapNGS | HBVCgp3_X | SMYD3//intronic | 7 | chr1 | 246648459 | - | AY123041 | 1248 | + | 1q44 |
| 14N | 20C227780_R303_CapNGS | HBVCgp3_X//upstream | SP100(dist=115529),LINC01907(dist=29798)//intergenic | 143 | chr2 | 231525848 | + | AY123041 | 558 | - | 2q37.1 |
| 14N | 20C227780_R303_CapNGS | HBVCgp4_PREC/C | FN1//intronic | 14776 | chr2 | 216270181 | - | AY123041 | 2267 | + | 2q35 |
| 14N | 20C227780_R303_CapNGS | HBVCgp4_PREC/C | FN1//intronic | 14776 | chr2 | 216270175 | - | AY123041 | 2267 | + | 2q35 |
| 14N | 20C227780_R303_CapNGS | HBVCgp4_PREC/C | FN1//intronic | 2218 | chr2 | 216264667 | + | AY123041 | 2286 | - | 2q35 |
| 14N | 20C227780_R303_CapNGS | HBVCgp3_X | FN1//intronic | 2245 | chr2 | 216264666 | + | AY123041 | 1686 | - | 2q35 |
| 14N | 20C227780_R303_CapNGS | HBVCgp3_X;HBVCgp4_PREC/C//upstream | CPS1//intronic | 23 | chr2 | 211463210 | + | AY123041 | 706 | + | 2q34 |
| 14N | 20C227780_R303_CapNGS | HBVCgp3_X | CPS1//intronic | 20 | chr2 | 211463111 | - | AY123041 | 1499 | + | 2q34 |
| 14N | 20C227780_R303_CapNGS | HBVCgp3_X | LRCH3//intronic | 8 | chr3 | 197568840 | - | AY123041 | 1499 | + | 3q29 |
| 14N | 20C227780_R303_CapNGS | HBVCgp3_X;HBVCgp4_PREC/C | COL5A2(dist=126113),WDR75(dist=135559)//intergenic | 434 | chr2 | 190170600 | + | AY123041 | 1712 | + | 2q32.2 |
| 14N | 20C227780_R303_CapNGS | HBVCgp4_PREC/C | C5orf47(dist=5289),NSG2(dist=34277)//intergenic | 3238 | chr5 | 173438432 | - | AY123041 | 1731 | + | 5q35.2 |
| 14N | 20C227780_R303_CapNGS | HBVCgp4_PREC/C | C5orf47(dist=5287),NSG2(dist=34279)//intergenic | 3099 | chr5 | 173438430 | - | AY123041 | 1731 | + | 5q35.2 |
| 14N | 20C227780_R303_CapNGS | HBVCgp3_X//upstream | SARDH(dist=10200),VAV2(dist=11739)//intergenic | 8 | chr9 | 136615277 | + | AY123041 | 577 | - | 9q34.2 |
| 14N | 20C227780_R303_CapNGS | HBVCgp4_PREC/C | WDR36(dist=26518),CAMK4(dist=66563)//intergenic | 9 | chr5 | 110492718 | + | AY123041 | 1821 | + | 5q22.1 |
| 14N | 20C227780_R303_CapNGS | HBVCgp3_X;HBVCgp4_PREC/C | CCNJ//exonic//CCNJ:NM_001134375:exon6:c.1098,CCNJ:NM_001134376:exon6:c.1062,CCNJ:NM_019084:exon6:c.1065 | 550 | chr10 | 97817944 | + | AY123041 | 1696 | - | 10q24.1 |
| 14N | 20C227780_R303_CapNGS | HBVCgp2_PRES1/PRES2/S;HBVCgp1_P; HBVCgp3_X;HBVCgp4_PREC/C//upstream;downstream | LINC02008(dist=1772838),LINC00971(dist=401892)//intergenic | 60 | chr3 | 84285664 | + | AY123041 | 2351 | - | 3p12.1 |
| 14N | 20C227780_R303_CapNGS | HBVCgp4_PREC/C | TENM4(dist=928324),LINC02720(dist=381846)//intergenic | 2603 | chr11 | 80080398 | + | AY123041 | 2305 | - | 11q14.1 |
| 14N | 20C227780_R303_CapNGS | HBVCgp3_X;HBVCgp4_PREC/C | FBXO22//intronic | 1421 | chr15 | 76206162 | + | AY123041 | 1692 | - | 15q24.2 |
| 14N | 20C227780_R303_CapNGS | HBVCgp3_X | RFWD3//intronic | 8 | chr16 | 74663110 | + | AY123041 | 1684 | + | 16q23.1 |
| 14N | 20C227780_R303_CapNGS | HBVCgp4_PREC/C | RFWD3//intronic | 11 | chr16 | 74663077 | - | AY123041 | 2081 | + | 16q23.1 |
| 14N | 20C227780_R303_CapNGS | HBVCgp4_PREC/C | PRPSAP1//intronic | 1096 | chr17 | 74318644 | + | AY123041 | 1810 | + | 17q25.1 |
| 14N | 20C227780_R303_CapNGS | HBVCgp3_X | PDZRN3-AS1(dist=61742),LINC02005(dist=119603)//intergenic | 15 | chr3 | 73738792 | + | AY123041 | 1667 | + | 3p13 |
| 14N | 20C227780_R303_CapNGS | HBVCgp3_X//upstream | SLC4A4//intronic | 28 | chr4 | 72147735 | + | AY123041 | 410 | + | 4q13.3 |
| 14N | 20C227780_R303_CapNGS | HBVCgp4_PREC/C | SERBP1(dist=81664),GADD45A(dist=173135)//intergenic | 1200 | chr1 | 67977749 | + | AY123041 | 2060 | - | 1p31.3 |
| 14N | 20C227780_R303_CapNGS | HBVCgp3_X//upstream | ROR1//intronic | 2208 | chr1 | 64251113 | + | AY123041 | 664 | + | 1p31.3 |
| 14N | 20C227780_R303_CapNGS | HBVCgp4_PREC/C | ROR1//intronic | 2232 | chr1 | 64251099 | + | AY123041 | 1942 | + | 1p31.3 |
| 14N | 20C227780_R303_CapNGS | HBVCgp4_PREC/C | C5orf64-AS1(dist=129349),KIF2A(dist=441115)//intergenic | 5 | chr5 | 61160874 | + | AY123041 | 2167 | + | 5q12.1 |
| 14N | 20C227780_R303_CapNGS | HBVCgp4_PREC/C | LINC02735(dist=46498),OR5AK2(dist=64337)//intergenic | 159 | chr11 | 56692052 | + | AY123041 | 2192 | - | 11q12.1 |
| 14N | 20C227780_R303_CapNGS | HBVCgp3_X | ATP8B4//intronic | 34 | chr15 | 50155556 | - | AY123041 | 1455 | + | 15q21.2 |
| 14N | 20C227780_R303_CapNGS | HBVCgp4_PREC/C | CWH43(dist=87716),NONE(dist=NONE)//intergenic | 640 | chr4 | 49151811 | + | AY123041 | 1862 | + | 4p11 |
| 14N | 20C227780_R303_CapNGS | HBVCgp4_PREC/C | KRBOX1(dist=12914),GASK1A(dist=23606)//intergenic | 28 | chr3 | 42997197 | + | AY123041 | 2187 | - | 3p22.1 |

| | | | | | | | | | | | |
|---|---|---|---|---|---|---|---|---|---|---|---|
| 14N | 20C227780_R303_CapNGS | NONE(dist=NONE),HBVCgp3_X(dist=1086)//intergenic | LRRFIP2(dist=54297),LOC152048(dist=11135)//intergenic | 8 | chr3 | 37272148 | + | AY123041 | 162 | - | 3p22.2 |
| 14N | 20C227780_R303_CapNGS | HBVCgp4_PREC/C | LINC00113(dist=155874),LINC00314(dist=106256)//intergenic | 412 | chr21 | 29279426 | + | AY123041 | 1927 | - | 21q21.3 |
| 14N | 20C227780_R303_CapNGS | HBVCgp3_X | EPHX2//intronic | 75 | chr8 | 27387620 | - | AY123041 | 1472 | + | 8p21.2 |
| 14N | 20C227780_R303_CapNGS | NONE(dist=NONE),HBVCgp3_X(dist=1024)//intergenic | LINC02573(dist=48007),LINC00320(dist=435903)//intergenic | 714 | chr21 | 21679005 | - | AY123041 | 224 | + | 21q21.1 |
| 14N | 20C227780_R303_CapNGS | HBVCgp3_X;HBVCgp4_PREC/C | APOB//exonic//APOB:NM_000384:exon26:c.6557 | 204 | chr2 | 21233183 | + | AY123041 | 1700 | - | 2p24.1 |
| 14N | 20C227780_R303_CapNGS | HBVCgp4_PREC/C | OR4N2(dist=30920),OR4K3(dist=7024)//intergenic | 3583 | chr14 | 20329332 | + | AY123041 | 1765 | + | 14q11.2 |
| 14N | 20C227780_R303_CapNGS | HBVCgp4_PREC/C | OR4N2(dist=30919),OR4K3(dist=7025)//intergenic | 3525 | chr14 | 20329331 | + | AY123041 | 1765 | + | 14q11.2 |
| 14N | 20C227780_R303_CapNGS | HBVCgp4_PREC/C//downstream | MIR5689HG(dist=48716),GCNT2(dist=15840)//intergenic | 19 | chr6 | 10505728 | + | AY123041 | 2947 | + | 6p24.3 |
| 14N | 20C227780_R303_CapNGS | HBVCgp2_PRES1/PRES2/S;HBVCgp1_P;HBVCgp3_X;HBVCgp4_PREC/C//upstream;downstream | LOC105374620(dist=59085),LINC01377(dist=198222)//intergenic | 591 | chr5 | 2979727 | - | AY123041 | 2338 | + | 5p15.33 |
| 14N | 20C227780_R303_CapNGS | HBVCgp4_PREC/C | LOC105374620(dist=59024),LINC01377(dist=198283)//intergeni | 597 | chr5 | 2979666 | + | AY123041 | 2032 | - | 5p15.33 |
| 15N | 20C227781_R303_CapNGS | HBVCgp4_PREC/C | TNIK//intronic | 6732 | chr3 | 170853534 | + | AY123041 | 1730 | - | 3q26.2 |
| 15N | 20C227781_R303_CapNGS | HBVCgp3_X;HBVCgp4_PREC/C | EIF5A2(dist=14020),SLC2A2(dist=73690)//intergenic | 6285 | chr3 | 170640446 | + | AY123041 | 1691 | - | 3q26.2 |
| 15N | 20C227781_R303_CapNGS | HBVCgp3_X;HBVCgp4_PREC/C | LINC01845(dist=187623),LINC01847(dist=122875)//intergenic | 1040 | chr5 | 159080907 | + | AY123041 | 1699 | - | 5q33.3 |
| 15N | 20C227781_R303_CapNGS | HBVCgp3_X;HBVCgp4_PREC/C | MIR4465(dist=580890),NMBR(dist=809711)//intergenic | 14015 | chr6 | 141585910 | + | AY123041 | 1697 | + | 6q24.1 |
| 15N | 20C227781_R303_CapNGS | HBVCgp4_PREC/C | OPCML(dist=233332),LINC02743(dist=17838)//intergenic | 20 | chr11 | 133635728 | + | AY123041 | 2309 | - | 11q25 |
| 15N | 20C227781_R303_CapNGS | HBVCgp3_X | MIR4445(dist=1324337),NECTIN3-AS1(dist=118084)//intergenic | 1548 | chr3 | 110646081 | + | AY123041 | 1667 | + | 3q13.13 |
| 15N | 20C227781_R303_CapNGS | HBVCgp4_PREC/C//downstream | ABCA1//intronic | 173 | chr9 | 107683068 | + | AY123041 | 2761 | + | 9q31.1 |
| 15N | 20C227781_R303_CapNGS | HBVCgp3_X;HBVCgp4_PREC/C | EPO//downstream | 411 | chr7 | 100322035 | + | AY123041 | 1700 | - | 7q22.1 |
| 15N | 20C227781_R303_CapNGS | HBVCgp4_PREC/C | C8orf34-AS1//ncRNA_intronic | 354 | chr8 | 69240985 | + | AY123041 | 1864 | + | 8q13.2 |
| 15N | 20C227781_R303_CapNGS | HBVCgp3_X | PPP6R2//intronic | 1571 | chr22 | 50850264 | + | AY123041 | 1668 | + | 22q13.33 |
| 15N | 20C227781_R303_CapNGS | HBVCgp4_PREC/C | PPP6R2//intronic | 1548 | chr22 | 50850256 | + | AY123041 | 1868 | + | 22q13.33 |
| 15N | 20C227781_R303_CapNGS | HBVCgp3_X | LINC01491(dist=103840),SLC24A5(dist=170914)//intergenic | 40 | chr15 | 48242273 | + | AY123041 | 1676 | + | 15q21.1 |
| 15N | 20C227781_R303_CapNGS | HBVCgp4_PREC/C | PRSS36(dist=21833),FUS(dist=8211)//intergenic | 12 | chr16 | 31183220 | - | AY123041 | 1914 | + | 16p11.2 |
| 15N | 20C227781_R303_CapNGS | HBVCgp3_X;HBVCgp4_PREC/C | LINC00929//ncRNA_intronic | 6 | chr15 | 26377136 | - | AY123041 | 1699 | + | 15q12 |
| 15N | 20C227781_R303_CapNGS | HBVCgp4_PREC/C | IQSEC1//UTR3 | 1361 | chr3 | 12940367 | + | AY123041 | 1958 | + | 3p25.2 |
| 15N | 20C227781_R303_CapNGS | HBVCgp3_X;HBVCgp4_PREC/C | PRAMEF12(dist=8518),PRAMEF1(dist=4980)//intergenic | 29 | chr1 | 12846566 | + | AY123041 | 1696 | - | 1p36.21 |
| 15N | 20C227781_R303_CapNGS | HBVCgp3_X;HBVCgp4_PREC/C | PRAMEF12(dist=8517),PRAMEF1(dist=4981)//intergenic | 22 | chr1 | 12846565 | - | AY123041 | 1696 | + | 1p36.21 |
| 16N | 20C227782_R303_CapNGS | HBVCgp4_PREC/C | SMYD3//intronic | 902 | chr1 | 246032989 | - | AY123041 | 1985 | + | 1q44 |
| 16N | 20C227782_R303_CapNGS | HBVCgp4_PREC/C | SMYD3//intronic | 906 | chr1 | 246032987 | + | AY123041 | 1985 | - | 1q44 |
| 16N | 20C227782_R303_CapNGS | HBVCgp4_PREC/C | FN1//exonic//FN1:NM_001306129:exon1:c.105,FN1:NM_001306130:exon1:c.105,FN1:NM_001306131:exon1:c.105,FN1:NM_001306132:exon1:c.105,FN1:NM_001365517:exon1:c.105,FN1:NM_001365518:exon1:c.105,FN1:NM_001365519:exon1:c.105,FN1:NM_001365520:exon1:c.105,FN1:NM_001365521:exon1:c.105,FN1:NM_001365522:exon1:c.105,FN1:NM_001365523:exon1:c.105,FN1:NM_001365524:exon1:c.105,FN1:NM_002026:exon1:c.105,FN1:NM_054034:exon1:c.105,FN1:NM_212474:exon1:c.105,FN1:NM_212476:exon1:c.105,FN1:NM_212478:exon1:c.105,FN1:NM_2124 | 14 | chr2 | 216300421 | - | AY123041 | 2292 | + | 2q35 |
| 16N | 20C227782_R303_CapNGS | HBVCgp3_X//upstream | FN1//exonic//FN1:NM_001306129:exon1:c.124,FN1:NM_001306130:exon1:c.124,FN1:NM_001306131:exon1:c.124,FN1:NM_001306132:exon1:c.124,FN1:NM_001365517:exon1:c.124,FN1:NM_001365518:exon1:c.124,FN1:NM_001365519:exon1:c.124,FN1:NM_001365520:exon1:c.124,FN1:NM_001365521:exon1:c.124,FN1:NM_001365522:exon1:c.124,FN1:NM_001365523:exon1:c.124,FN1:NM_001365524:exon1:c.124,FN1:NM_002026:exon1:c.124,FN1:NM_054034:exon1:c.124,FN1:NM_212474:exon1:c.124,FN1:NM_212476:exon1:c.124,FN1:NM_212478:exon1:c.124,FN1:NM_2124 | 17 | chr2 | 216300402 | + | AY123041 | 414 | + | 2q35 |
| 16N | 20C227782_R303_CapNGS | HBVCgp3_X//upstream | FN1//intronic | 18 | chr2 | 216291809 | - | AY123041 | 587 | + | 2q35 |
| 16N | 20C227782_R303_CapNGS | HBVCgp3_X//upstream | FN1//intronic | 237 | chr2 | 216256668 | + | AY123041 | 347 | - | 2q35 |
| 16N | 20C227782_R303_CapNGS | HBVCgp4_PREC/C | PLXNA2(dist=178738),LINC01717(dist=305566)//intergenic | 1405 | chr1 | 208596467 | - | AY123041 | 2024 | + | 1q32.2 |
| 16N | 20C227782_R303_CapNGS | HBVCgp4_PREC/C | PLXNA2(dist=178734),LINC01717(dist=305570)//intergenic | 1408 | chr1 | 208596463 | + | AY123041 | 2024 | - | 1q32.2 |
| 16N | 20C227782_R303_CapNGS | HBVCgp4_PREC/C//downstream | RALGPS2(dist=73294),FAM20B(dist=30668)//intergenic | 6 | chr1 | 178964271 | + | AY123041 | 3057 | - | 1q25.2 |
| 16N | 20C227782_R303_CapNGS | HBVCgp3_X;HBVCgp4_PREC/C | FNIP2//exonic//FNIP2:NM_001346043:exon14:c.1949,FNIP2:NM_001323916:exon15:c.3080,FNIP2:NM_020840:exon15:c.3011,FNIP2:NM_001366843:exon16:c.3170 | 13961 | chr4 | 159812659 | + | AY123041 | 1689 | - | 4q32.1 |
| 16N | 20C227782_R303_CapNGS | HBVCgp3_X;HBVCgp4_PREC/C | FNIP2//exonic//FNIP2:NM_001346043:exon14:c.1948,FNIP2:NM_001323916:exon15:c.3079,FNIP2:NM_020840:exon15:c.3010,FNIP2:NM_001366843:exon16:c.3169 | 14018 | chr4 | 159812658 | + | AY123041 | 1691 | - | 4q32.1 |
| 16N | 20C227782_R303_CapNGS | HBVCgp3_X;HBVCgp4_PREC/C | NTM//intronic | 792 | chr11 | 131438008 | + | AY123041 | 1699 | + | 11q25 |
| 16N | 20C227782_R303_CapNGS | HBVCgp4_PREC/C | LINC01950(dist=39877),EFNA5(dist=326003)//intergenic | 16 | chr5 | 106386592 | - | AY123041 | 1785 | + | 5q21.3 |
| 16N | 20C227782_R303_CapNGS | HBVCgp4_PREC/C | GPRIN3(dist=119028),SNCA(dist=297271)//intergenic | 66 | chr4 | 90347979 | - | AY123041 | 2120 | + | 4q22.1 |
| 16N | 20C227782_R303_CapNGS | HBVCgp3_X;HBVCgp4_PREC/C | GPRIN3(dist=119010),SNCA(dist=297289)//intergenic | 18 | chr4 | 90347961 | + | AY123041 | 1701 | + | 4q22.1 |
| 16N | 20C227782_R303_CapNGS | HBVCgp4_PREC/C | PKN2-AS1//ncRNA_intronic | 184 | chr1 | 89089231 | + | AY123041 | 1858 | + | 1p22.2 |

| | | | | | | | | | | | |
|---|---|---|---|---|---|---|---|---|---|---|---|
| 16N | 20C227782_R303_CapNGS | HBVCgp4_PREC/C | SLAIN1(dist=38379),EDNRB-AS1(dist=16316)//intergenic | 7 | chr13 | 78376756 | + | AY123041 | 1974 | + | 13q22.3 |
| 16N | 20C227782_R303_CapNGS | HBVCgp3_X;HBVCgp4_PREC/C | LINC01360(dist=76391),LINC02238(dist=259411)//intergenic | 1087 | chr1 | 73880951 | + | AY123041 | 1691 | - | 1p31.1 |
| 16N | 20C227782_R303_CapNGS | HBVCgp3_X;HBVCgp4_PREC/C | LINC01360(dist=76355),LINC02238(dist=259447)//intergenic | 1084 | chr1 | 73880915 | + | AY123041 | 1694 | - | 1p31.1 |
| 16N | 20C227782_R303_CapNGS | HBVCgp4_PREC/C | KRTAP5-11(dist=201477),FAM86C1(dist=3159)//intergenic | 8 | chr11 | 71495398 | + | AY123041 | 1773 | + | 11q13.4 |
| 16N | 20C227782_R303_CapNGS | HBVCgp4_PREC/C | WNK3(dist=57047),TSR2(dist=25282)//intergenic | 212 | chrX | 54441526 | + | AY123041 | 1941 | - | Xp11.22 |
| 16N | 20C227782_R303_CapNGS | HBVCgp3_X//upstream | LINC01478//ncRNA_intronic | 70 | chr18 | 42010851 | + | AY123041 | 352 | + | 18q12.3 |
| 16N | 20C227782_R303_CapNGS | HBVCgp4_PREC/C//downstream | ULK4//intronic | 5 | chr3 | 41434130 | + | AY123041 | 3006 | - | 3p22.1 |
| 16N | 20C227782_R303_CapNGS | HBVCgp4_PREC/C | SULT1A1//exonic//SULT1A1:NM_177534:exon3:c.358,SULT1A1:NM_001055:exon4:c.358,SULT1A1:NM_177529:exon4:c.358,SULT1A1:NM_177530:exon4:c.358 | 80 | chr16 | 28619626 | - | AY123041 | 2240 | + | 16p11.2 |
| 16N | 20C227782_R303_CapNGS | HBVCgp4_PREC/C | YPEL1//intronic | 3138 | chr22 | 22068201 | - | AY123041 | 1777 | + | 22q11.21 |
| 16N | 20C227782_R303_CapNGS | HBVCgp4_PREC/C | YPEL1//intronic | 3149 | chr22 | 22068197 | + | AY123041 | 1777 | - | 22q11.21 |
| 16N | 20C227782_R303_CapNGS | HBVCgp4_PREC/C//downstream | OR11H4(dist=19526),TTC5(dist=22935)//intergenic | 5 | chr14 | 20731451 | + | AY123041 | 3006 | + | 14q11.2 |
| 16N | 20C227782_R303_CapNGS | HBVCgp4_PREC/C//downstream | SATB1-AS1(dist=421724),KCNH8(dist=196672)//intergenic | 8 | chr3 | 18993330 | + | AY123041 | 2798 | - | 3p24.3 |
| 16N | 20C227782_R303_CapNGS | HBVCgp2_PRES1/PRES2/S;HBVCgp1_P;HBVCgp3_X;HBVCgp4_PREC/C//upstream;downstream | LPIN1//intronic | 47 | chr2 | 11945701 | - | AY123041 | 2376 | + | 2p25.1 |
| 16N | 20C227782_R303_CapNGS | NONE(dist=NONE),HBVCgp3_X(dist=1145)//intergenic | PIEZO2(dist=167561),LINC01928(dist=138155)//intergenic | 520 | chr18 | 11316322 | - | AY123041 | 103 | + | 18p11.21 |
| 17N | 20C227783_R303_CapNGS | HBVCgp4_PREC/C | LINC01921(dist=65025),DIRC3-AS1(dist=346069)//intergenic | 5 | chr2 | 217801387 | + | AY123041 | 1847 | - | 2q35 |
| 17N | 20C227783_R303_CapNGS | HBVCgp4_PREC/C | FN1//intronic | 705 | chr2 | 216268775 | + | AY123041 | 1743 | - | 2q35 |
| 17N | 20C227783_R303_CapNGS | HBVCgp3_X;HBVCgp4_PREC/C//upstream | FN1//intronic | 16 | chr2 | 216265222 | + | AY123041 | 841 | - | 2q35 |
| 17N | 20C227783_R303_CapNGS | HBVCgp3_X//upstream | FN1//exonic//FN1:NM_001306131:exon25:c.3968,FN1:NM_001306132:exon25:c.3968,FN1:NM_001365518:exon25:c.3968,FN1:NM_001365520:exon25:c.3968,FN1:NM_001365523:exon25:c.3968,FN1:NM_001365524:exon25:c.3968,FN1:NM_002026:exon25:c.3968,FN1:NM_212474:exon25:c.3968,FN1:NM_212476:exon25:c.3968,FN1:NM_212478:exon25:c.3968,FN1:NM_001306129:exon26:c.4241,FN1:NM_001306130:exon26:c.4241,FN1:NM_001365517:exon26:c.4241,FN1:NM_001365519:exon26:c.4241,FN1:NM_001365521:exon26:c.4241,FN1:NM_001365522:exon26:c.4241,FN1:N[illegible] | 355 | chr2 | 216256366 | + | AY123041 | 625 | - | 2q35 |
| 17N | 20C227783_R303_CapNGS | HBVCgp3_X//upstream | NLGN1//intronic | 181 | chr3 | 173407110 | + | AY123041 | 577 | + | 3q26.31 |
| 17N | 20C227783_R303_CapNGS | HBVCgp3_X;HBVCgp4_PREC/C | GPR149(dist=397960),MME(dist=195974)//intergenic | 962 | chr3 | 154545939 | + | AY123041 | 1696 | - | 3q25.2 |
| 17N | 20C227783_R303_CapNGS | HBVCgp3_X//upstream | LINC01817(dist=183723),RND3(dist=27463)//intergenic | 9 | chr2 | 151297244 | + | AY123041 | 511 | - | 2q23.3 |
| 17N | 20C227783_R303_CapNGS | HBVCgp4_PREC/C//downstream | LINC02712(dist=1655),LINC02098(dist=870078)//intergenic | 9 | chr11 | 127208583 | - | AY123041 | 2763 | + | 11q24.2 |
| 17N | 20C227783_R303_CapNGS | HBVCgp3_X;HBVCgp4_PREC/C//upstream | DENND1A//intronic | 19 | chr9 | 126248611 | - | AY123041 | 781 | + | 9q33.3 |
| 17N | 20C227783_R303_CapNGS | HBVCgp4_PREC/C | SUDS3(dist=313287),LINC02423(dist=27434)//intergenic | 110 | chr12 | 119169125 | - | AY123041 | 1950 | + | 12q24.23 |
| 17N | 20C227783_R303_CapNGS | HBVCgp4_PREC/C | LOC285638//ncRNA_intronic | 257 | chr5 | 108638258 | - | AY123041 | 1743 | + | 5q21.3 |
| 17N | 20C227783_R303_CapNGS | HBVCgp3_X//upstream | BTBD11//intronic | 10 | chr12 | 107925979 | - | AY123041 | 398 | + | 12q23.3 |
| 17N | 20C227783_R303_CapNGS | NONE(dist=NONE),HBVCgp3_X(dist=1153)//intergenic | CERS3-AS1//ncRNA_intronic | 8 | chr15 | 100969421 | + | AY123041 | 95 | - | 15q26.3 |
| 17N | 20C227783_R303_CapNGS | HBVCgp3_X | BCL11B//intronic | 7 | chr14 | 99646808 | - | AY123041 | 1584 | + | 14q32.2 |
| 17N | 20C227783_R303_CapNGS | HBVCgp4_PREC/C | DZIP1//intronic | 1246 | chr13 | 96258921 | - | AY123041 | 2245 | + | 13q32.1 |
| 17N | 20C227783_R303_CapNGS | HBVCgp2_PRES1/PRES2/S;HBVCgp1_P;HBVCgp3_X;HBVCgp4_PREC/C//upstream;downstream | MIR4436A(dist=795958),LOC107985911(dist=1897257)//intergenic | 92 | chr2 | 89907926 | - | AY123041 | 2477 | + | 2p11.2 |
| 17N | 20C227783_R303_CapNGS | HBVCgp4_PREC/C//downstream | MIR4436A(dist=795847),LOC107985911(dist=1897368)//interge[illegible] | 39 | chr2 | 89907815 | + | AY123041 | 2735 | + | 2p11.2 |
| 17N | 20C227783_R303_CapNGS | HBVCgp3_X;HBVCgp4_PREC/C//upstream | EPHA3(dist=280686),NONE(dist=NONE)//intergenic | 7 | chr3 | 89811970 | - | AY123041 | 774 | + | 3p11.1 |
| 17N | 20C227783_R303_CapNGS | HBVCgp3_X | DUSP6(dist=63737),POC1B(dist=3483)//intergenic | 9742 | chr12 | 89810015 | + | AY123041 | 1675 | + | 12q21.33 |
| 17N | 20C227783_R303_CapNGS | HBVCgp3_X;HBVCgp4_PREC/C | RHBDF2(dist=24344),CYGB(dist=1585)//intergenic | 4206 | chr17 | 74521853 | + | AY123041 | 1702 | - | 17q25.1 |
| 17N | 20C227783_R303_CapNGS | HBVCgp3_X//upstream | MIDEAS//intronic | 15 | chr14 | 74234926 | - | AY123041 | 648 | + | 14q24.3 |
| 17N | 20C227783_R303_CapNGS | HBVCgp4_PREC/C | CD276//intronic | 154 | chr15 | 73991363 | + | AY123041 | 2063 | - | 15q24.1 |
| 17N | 20C227783_R303_CapNGS | HBVCgp3_X;HBVCgp4_PREC/C//upstream | FTX//ncRNA_intronic | 8 | chrX | 73486199 | + | AY123041 | 794 | + | Xq13.2 |
| 17N | 20C227783_R303_CapNGS | HBVCgp4_PREC/C//downstream | LOC101928343(dist=37926),RAB37(dist=7209)//intergenic | 96 | chr17 | 72660061 | + | AY123041 | 2926 | - | 17q25.1 |
| 17N | 20C227783_R303_CapNGS | HBVCgp2_PRES1/PRES2/S;HBVCgp3_X;HBVCgp4_PREC/C//upstream;downstre[illegible] | LOC101928343//ncRNA_intronic | 337 | chr17 | 72604773 | + | AY123041 | 2683 | - | 17q25.1 |
| 17N | 20C227783_R303_CapNGS | HBVCgp3_X//upstream | PDXDC2P-NPIPB14P//ncRNA_intronic | 1762 | chr16 | 70058888 | - | AY123041 | 493 | + | 16q22.1 |
| 17N | 20C227783_R303_CapNGS | HBVCgp3_X//upstream | PDXDC2P-NPIPB14P//ncRNA_intronic | 1861 | chr16 | 70058887 | + | AY123041 | 493 | - | 16q22.1 |
| 17N | 20C227783_R303_CapNGS | HBVCgp2_PRES1/PRES2/S;HBVCgp3_X;HBVCgp4_PREC/C//upstream;downstre[illegible] | VKORC1L1//upstream | 1917 | chr7 | 65337815 | + | AY123041 | 2589 | - | 7q11.21 |
| 17N | 20C227783_R303_CapNGS | HBVCgp4_PREC/C//downstream | PRKCA//intronic | 11 | chr17 | 64549051 | + | AY123041 | 2727 | + | 17q24.2 |
| 17N | 20C227783_R303_CapNGS | HBVCgp3_X | MIR1-1HG-AS1//ncRNA_intronic | 281 | chr20 | 60959461 | + | AY123041 | 1377 | - | 20q13.33 |

| | | | | | | | | | | | |
|---|---|---|---|---|---|---|---|---|---|---|---|
| 17N | 20C227783_R303_CapNGS | HBVCgp3_X;HBVCgp4_PREC/C//upstream | LOC101927620(dist=437203),MIR5580(dist=353696)//intergenic | 9 | chr14 | 54061449 | - | AY123041 | 1012 | + | 14q22.1 |
| 17N | 20C227783_R303_CapNGS | HBVCgp3_X | CACNA1D//intronic | 50 | chr3 | 53740185 | + | AY123041 | 1483 | + | 3p21.1 |
| 17N | 20C227783_R303_CapNGS | HBVCgp3_X//upstream | TOX2(dist=4437),JPH2(dist=37646)//intergenic | 99 | chr20 | 42702691 | + | AY123041 | 511 | - | 20q13.12 |
| 17N | 20C227783_R303_CapNGS | HBVCgp3_X;HBVCgp4_PREC/C//upstream | LINC02484(dist=1389256),ARAP2(dist=407001)//intergenic | 8 | chr4 | 35660625 | + | AY123041 | 697 | - | 4p15.1 |
| 17N | 20C227783_R303_CapNGS | HBVCgp4_PREC/C | LOC101929172(dist=81743),GFRA2(dist=299207)//intergenic | 8 | chr8 | 21248703 | + | AY123041 | 2300 | - | 8p21.3 |
| 17N | 20C227783_R303_CapNGS | HBVCgp2_PRES1/PRES2/S;HBVCgp1_P;HBVCgp3_X;HBVCgp4_PREC/C//upstream;downstream | NONE(dist=NONE),CHEK2P2(dist=77049)//intergenic | 10 | chr15 | 20410948 | - | AY123041 | 2332 | + | 15q11.1 |
| 17N | 20C227783_R303_CapNGS | HBVCgp3_X//upstream | CTAGE1(dist=120239),LOC101927571(dist=185337)//intergenic | 2754 | chr18 | 20118117 | + | AY123041 | 542 | + | 18q11.2 |
| 17N | 20C227783_R303_CapNGS | HBVCgp3_X//upstream | CTAGE1(dist=120238),LOC101927571(dist=185338)//intergenic | 2659 | chr18 | 20118116 | + | AY123041 | 542 | + | 18q11.2 |
| 17N | 20C227783_R303_CapNGS | HBVCgp2_PRES1/PRES2/S;HBVCgp1_P;HBVCgp3_X;HBVCgp4_PREC/C//upstream;downstream | GATA6(dist=146238),CTAGE1(dist=64833)//intergenic | 3186 | chr18 | 19928729 | + | AY123041 | 2473 | + | 18q11.2 |
| 17N | 20C227783_R303_CapNGS | HBVCgp2_PRES1/PRES2/S;HBVCgp1_P;HBVCgp3_X;HBVCgp4_PREC/C//upstream;downstream | GATA6(dist=146232),CTAGE1(dist=64839)//intergenic | 3181 | chr18 | 19928723 | + | AY123041 | 2473 | + | 18q11.2 |
| 17N | 20C227783_R303_CapNGS | HBVCgp4_PREC/C//downstream | ICA1//intronic | 176 | chr7 | 8211722 | - | AY123041 | 2872 | + | 7p21.3 |
| 17N | 20C227783_R303_CapNGS | HBVCgp4_PREC/C//downstream | ICA1//intronic | 176 | chr7 | 8211717 | - | AY123041 | 2872 | + | 7p21.3 |
| 17N | 20C227783_R303_CapNGS | HBVCgp3_X;HBVCgp4_PREC/C | TNFAIP8L1//intronic | 63 | chr19 | 4645205 | + | AY123041 | 1690 | - | 19p13.3 |
| 19N | 20C227785_R303_CapNGS | HBVCgp3_X;HBVCgp4_PREC/C | ASTN1//intronic | 10 | chr1 | 177127954 | + | AY123041 | 1699 | - | 1q25.2 |
| 19N | 20C227785_R303_CapNGS | HBVCgp4_PREC/C//downstream | TENM2//intronic | 46 | chr5 | 166550541 | - | AY123041 | 2861 | + | 5q34 |
| 19N | 20C227785_R303_CapNGS | NONE(dist=NONE),HBVCgp3_X(dist=1134)//intergenic | OR10J1(dist=46753),OR10J5(dist=47358)//intergenic | 9 | chr1 | 159457510 | + | AY123041 | 114 | - | 1q23.2 |
| 19N | 20C227785_R303_CapNGS | HBVCgp4_PREC/C | TPK1(dist=1697519),CNTNAP2(dist=240698)//intergenic | 7 | chr7 | 146230665 | + | AY123041 | 1806 | + | 7q35 |
| 19N | 20C227785_R303_CapNGS | HBVCgp4_PREC/C//downstream | UTRN(dist=145825),EPM2A(dist=626446)//intergenic | 6 | chr6 | 145319995 | + | AY123041 | 2723 | + | 6q24.2 |
| 19N | 20C227785_R303_CapNGS | HBVCgp4_PREC/C//downstream | NOTCH1//intronic | 114 | chr9 | 139438788 | + | AY123041 | 2939 | + | 9q34.3 |
| 19N | 20C227785_R303_CapNGS | HBVCgp3_X//upstream | NCKAP5//intronic | 6 | chr2 | 133928302 | + | AY123041 | 513 | + | 2q21.2 |
| 19N | 20C227785_R303_CapNGS | HBVCgp3_X//upstream | CNTNAP5(dist=46549),LINC01941(dist=1142333)//intergenic | 11 | chr2 | 125725344 | + | AY123041 | 510 | - | 2q14.3 |
| 19N | 20C227785_R303_CapNGS | HBVCgp4_PREC/C//downstream | CCDC138//intronic | 9 | chr2 | 109450013 | - | AY123041 | 2742 | + | 2q12.3 |
| 19N | 20C227785_R303_CapNGS | HBVCgp4_PREC/C | FAM155A//intronic | 14528 | chr13 | 108436442 | + | AY123041 | 2153 | - | 13q33.3 |
| 19N | 20C227785_R303_CapNGS | HBVCgp3_X | FAM155A//intronic | 14324 | chr13 | 108436433 | + | AY123041 | 1683 | - | 13q33.3 |
| 19N | 20C227785_R303_CapNGS | NONE(dist=NONE),HBVCgp3_X(dist=1119)//intergenic | IL1RAPL2//intronic | 30 | chrX | 104891249 | + | AY123041 | 129 | + | Xq22.3 |
| 19N | 20C227785_R303_CapNGS | HBVCgp3_X//upstream | LOC101929353(dist=29566),SNORA101A(dist=150056)//interge | 9 | chr4 | 101166228 | + | AY123041 | 303 | + | 4q24 |
| 19N | 20C227785_R303_CapNGS | NONE(dist=NONE),HBVCgp3_X(dist=1220)//intergenic | CNTN5//intronic | 5 | chr11 | 99115579 | - | AY123041 | 28 | + | 11q22.1 |
| 19N | 20C227785_R303_CapNGS | NONE(dist=NONE),HBVCgp3_X(dist=1134)//intergenic | MAML2//intronic | 6 | chr11 | 95938942 | + | AY123041 | 114 | - | 11q21 |
| 19N | 20C227785_R303_CapNGS | HBVCgp3_X | SMCO4(dist=46772),CEP295(dist=71519)//intergenic | 274 | chr11 | 93323329 | + | AY123041 | 1418 | + | 11q21 |
| 19N | 20C227785_R303_CapNGS | HBVCgp4_PREC/C | LINC01049(dist=222680),LINC00410(dist=132933)//intergenic | 8 | chr13 | 91410275 | + | AY123041 | 1744 | - | 13q31.3 |
| 19N | 20C227785_R303_CapNGS | HBVCgp3_X;HBVCgp4_PREC/C//upstream | DOP1A//intronic | 1559 | chr6 | 83854127 | + | AY123041 | 800 | + | 6q14.1 |
| 19N | 20C227785_R303_CapNGS | NONE(dist=NONE),HBVCgp3_X(dist=1131)//intergenic | DOP1A//intronic | 1563 | chr6 | 83854122 | + | AY123041 | 117 | + | 6q14.1 |
| 19N | 20C227785_R303_CapNGS | HBVCgp3_X//upstream | RASGEF1B(dist=88288),HNRNPD(dist=792302)//intergenic | 181 | chr4 | 82481349 | + | AY123041 | 339 | + | 4q21.22 |
| 19N | 20C227785_R303_CapNGS | HBVCgp3_X//upstream | LIN7A(dist=12748),ACSS3(dist=127416)//intergenic | 11 | chr12 | 81344442 | + | AY123041 | 288 | + | 12q21.31 |
| 19N | 20C227785_R303_CapNGS | HBVCgp4_PREC/C | LINC01088//ncRNA_intronic | 252 | chr4 | 80028618 | - | AY123041 | 2168 | + | 4q21.21 |
| 19N | 20C227785_R303_CapNGS | HBVCgp4_PREC/C//downstream | WWOX//intronic | 229 | chr16 | 78737273 | + | AY123041 | 3036 | - | 16q23.1 |
| 19N | 20C227785_R303_CapNGS | HBVCgp3_X;HBVCgp4_PREC/C//upstream | SOX9(dist=109339),LINC02003(dist=107059)//intergenic | 263 | chr17 | 70231893 | + | AY123041 | 998 | + | 17q24.3 |
| 19N | 20C227785_R303_CapNGS | HBVCgp4_PREC/C//downstream | ZWILCH//intronic | 441 | chr15 | 66817332 | + | AY123041 | 2798 | + | 15q22.31 |
| 19N | 20C227785_R303_CapNGS | HBVCgp3_X | CDH8//intronic | 505 | chr16 | 61880487 | + | AY123041 | 1625 | - | 16q21 |
| 19N | 20C227785_R303_CapNGS | HBVCgp4_PREC/C | LINC00520(dist=65755),PELI2(dist=255982)//intergenic | 15 | chr14 | 56329147 | - | AY123041 | 2152 | + | 14q22.3 |
| 19N | 20C227785_R303_CapNGS | HBVCgp3_X//upstream | LINC00458(dist=17786),MIR1297(dist=161315)//intergenic | 5 | chr13 | 54724792 | - | AY123041 | 308 | + | 13q14.3 |
| 19N | 20C227785_R303_CapNGS | HBVCgp3_X//upstream | LINC00648(dist=1451891),RPS29(dist=321598)//intergenic | 248 | chr14 | 49716108 | + | AY123041 | 369 | - | 14q21.3 |
| 19N | 20C227785_R303_CapNGS | HBVCgp4_PREC/C | LINC01310(dist=63470),NONE(dist=NONE)//intergenic | 328 | chr22 | 49357668 | + | AY123041 | 1842 | + | 22q13.32 |
| 19N | 20C227785_R303_CapNGS | HBVCgp4_PREC/C | ALG10B(dist=217613),CPNE8(dist=104869)//intergenic | 246 | chr12 | 38941136 | + | AY123041 | 2117 | + | 12q12 |
| 19N | 20C227785_R303_CapNGS | HBVCgp3_X | MID1IP1(dist=245400),LINC01281(dist=253027)//intergenic | 16 | chrX | 38911183 | + | AY123041 | 1373 | - | Xp11.4 |
| 19N | 20C227785_R303_CapNGS | HBVCgp4_PREC/C//downstream | LINC02472(dist=992669),PCDH7(dist=506539)//intergenic | 214 | chr4 | 30215452 | + | AY123041 | 2940 | + | 4p15.1 |
| 19N | 20C227785_R303_CapNGS | HBVCgp3_X//upstream | SEL1L3//intronic | 395 | chr4 | 25825348 | + | AY123041 | 484 | + | 4p15.2 |
| 22N | 20C227787_R303_CapNGS | HBVCgp3_X;HBVCgp4_PREC/C | DGKI//intronic | 3382 | chr7 | 137463351 | - | AY123041 | 1694 | + | 7q33 |
| 22N | 20C227787_R303_CapNGS | HBVCgp3_X;HBVCgp4_PREC/C | DGKI//intronic | 3384 | chr7 | 137463350 | + | AY123041 | 1694 | - | 7q33 |
| 22N | 20C227787_R303_CapNGS | HBVCgp4_PREC/C | FAM170A(dist=56696),PRR16(dist=771760)//intergenic | 5 | chr5 | 119028213 | + | AY123041 | 1946 | + | 5q23.1 |

| | | | | | | | | | | | |
|---|---|---|---|---|---|---|---|---|---|---|---|
| 22N | 20C227787_R303_CapNGS | HBVCgp3_X//upstream | LOC388813(dist=68592),LINC02246(dist=105139)//intergenic | 8 | chr21 | 16084020 | + | AY123041 | 558 | - | 21q11.2 |
| 22N | 20C227787_R303_CapNGS | HBVCgp3_X | PRKCZ//intronic | 30806 | chr1 | 1982503 | - | AY123041 | 1647 | + | 1p36.33 |
| 22N | 20C227787_R303_CapNGS | HBVCgp3_X | PRKCZ//intronic | 30806 | chr1 | 1982499 | - | AY123041 | 1647 | + | 1p36.33 |
| 23N | 20C227788_R303_CapNGS | HBVBgp4_C | TLL1(dist=60918),SPOCK3(dist=568009)//intergenic | 6 | chr4 | 167086527 | - | AF100309 | 2391 | + | 4q32.3 |
| 23N | 20C227788_R303_CapNGS | HBVBgp4_C | NMI//downstream | 847 | chr2 | 152126629 | + | AF100309 | 2034 | + | 2q23.3 |
| 23N | 20C227788_R303_CapNGS | HBVBgp3_X | LOC102724804(dist=19288),SDC2(dist=87087)//intergenic | 1326 | chr8 | 97418954 | + | AF100309 | 1820 | + | 8q22.1 |
| 23N | 20C227788_R303_CapNGS | HBVBgp4_C//downstream | CNBD1(dist=8050),DCAF4L2(dist=479839)//intergenic | 21 | chr8 | 88403137 | + | AF100309 | 2853 | - | 8q21.3 |
| 23N | 20C227788_R303_CapNGS | HBVBgp2_S | ACTG1P17//ncRNA_intronic | 177 | chr15 | 83398823 | + | AF100309 | 729 | - | 15q25.2 |
| 23N | 20C227788_R303_CapNGS | HBVBgp2_S | RASGRF2//intronic | 9863 | chr5 | 80302661 | + | AF100309 | 592 | + | 5q14.1 |
| 23N | 20C227788_R303_CapNGS | HBVBgp3_X | ADCY1//intronic | 5700 | chr7 | 45704302 | + | AF100309 | 1828 | - | 7p12.3 |
| 23N | 20C227788_R303_CapNGS | HBVBgp3_X | ADCY1//intronic | 5692 | chr7 | 45704301 | + | AF100309 | 1828 | - | 7p12.3 |
| 23N | 20C227788_R303_CapNGS | HBVBgp3_X | ADCY1//intronic | 5692 | chr7 | 45704300 | + | AF100309 | 1828 | - | 7p12.3 |
| 23N | 20C227788_R303_CapNGS | HBVBgp1_P;HBVBgp4_C;HBVBgp3_X//upstream;downstream | ADCY1//UTR3 | 5693 | chr7 | 45704244 | + | AF100309 | 1879 | + | 7p12.3 |
| 23N | 20C227788_R303_CapNGS | HBVBgp1_P;HBVBgp4_C;HBVBgp3_X//upstream;downstream | ADCY1//UTR3 | 5700 | chr7 | 45704241 | + | AF100309 | 1879 | + | 7p12.3 |
| 23N | 20C227788_R303_CapNGS | HBVBgp3_X;HBVBgp2_S//upstream;downstream | PBK(dist=27301),SCARA5(dist=4765)//intergenic | 12 | chr8 | 27722635 | + | AF100309 | 863 | - | 8p21.1 |
| 23N | 20C227788_R303_CapNGS | HBVBgp2_S | FAM126A(dist=28758),KLHL7-DT(dist=58340)//intergenic | 284 | chr7 | 23082507 | + | AF100309 | 410 | - | 7p15.3 |
| 23N | 20C227788_R303_CapNGS | HBVBgp4_C | ZNF709(dist=8979),ZNF564(dist=31578)//intergenic | 6 | chr19 | 12604609 | + | AF100309 | 2380 | - | 19p13.2 |
| 24N | 20C227789_R303_CapNGS | HBVBgp4_C | LOC105379514//ncRNA_intronic | 3429 | chr4 | 190600354 | + | AF100309 | 2157 | + | 4q35.2 |
| 24N | 20C227789_R303_CapNGS | HBVBgp2_S//upstream | NONE(dist=NONE),LINC00290(dist=883366)//intergenic | 3037 | chr4 | 181101877 | + | AF100309 | 138 | - | 4q34.3 |
| 24N | 20C227789_R303_CapNGS | HBVBgp3_X | PLD1//intronic | 13 | chr3 | 171491295 | + | AF100309 | 1819 | - | 3q26.31 |
| 24N | 20C227789_R303_CapNGS | HBVBgp4_C | LINC00880(dist=20641),CCNL1(dist=2859)//intergenic | 6460 | chr3 | 156861432 | - | AF100309 | 2450 | + | 3q25.31 |
| 24N | 20C227789_R303_CapNGS | HBVBgp4_C | LINC00880(dist=20640),CCNL1(dist=2860)//intergenic | 6489 | chr3 | 156861431 | + | AF100309 | 2450 | - | 3q25.31 |
| 24N | 20C227789_R303_CapNGS | HBVBgp3_X | LINC00880(dist=20592),CCNL1(dist=2908)//intergenic | 6478 | chr3 | 156861383 | + | AF100309 | 1801 | - | 3q25.31 |
| 24N | 20C227789_R303_CapNGS | HBVBgp3_X | SH2D2A//intronic | 17 | chr1 | 156784490 | + | AF100309 | 1504 | + | 1q23.1 |
| 24N | 20C227789_R303_CapNGS | HBVBgp2_S | TSC22D2//intronic | 11 | chr3 | 150153125 | + | AF100309 | 740 | - | 3q25.1 |
| 24N | 20C227789_R303_CapNGS | HBVBgp3_X | ARHGAP15//intronic | 1661 | chr2 | 144115801 | + | AF100309 | 1789 | + | 2q22.3 |
| 24N | 20C227789_R303_CapNGS | HBVBgp4_C | R3HDM1//intronic | 23736 | chr2 | 136453079 | - | AF100309 | 2380 | + | 2q21.3 |
| 24N | 20C227789_R303_CapNGS | HBVBgp4_C | R3HDM1//intronic | 23738 | chr2 | 136453078 | - | AF100309 | 2380 | + | 2q21.3 |
| 24N | 20C227789_R303_CapNGS | HBVBgp4_C | R3HDM1//intronic | 23803 | chr2 | 136453077 | - | AF100309 | 2379 | + | 2q21.3 |
| 24N | 20C227789_R303_CapNGS | HBVBgp4_C | R3HDM1//intronic | 23803 | chr2 | 136453075 | - | AF100309 | 2379 | + | 2q21.3 |
| 24N | 20C227789_R303_CapNGS | HBVBgp4_C | R3HDM1//intronic | 23803 | chr2 | 136453073 | - | AF100309 | 2379 | + | 2q21.3 |
| 24N | 20C227789_R303_CapNGS | HBVBgp3_X | ANHX//intronic | 11933 | chr12 | 133802478 | + | AF100309 | 1819 | + | 12q24.33 |
| 24N | 20C227789_R303_CapNGS | HBVBgp3_X | ANHX//intronic | 11816 | chr12 | 133802477 | + | AF100309 | 1822 | + | 12q24.33 |
| 24N | 20C227789_R303_CapNGS | HBVBgp2_S | MGMT//intronic | 316 | chr10 | 131536234 | + | AF100309 | 748 | + | 10q26.3 |
| 24N | 20C227789_R303_CapNGS | HBVBgp4_C | SPAG17//intronic | 5 | chr1 | 118675763 | + | AF100309 | 1946 | + | 1p12 |
| 24N | 20C227789_R303_CapNGS | HBVBgp4_C | RASA3(dist=18030),CDC16(dist=84192)//intergenic | 14783 | chr13 | 114916128 | + | AF100309 | 2224 | + | 13q34 |
| 24N | 20C227789_R303_CapNGS | HBVBgp3_X | ELOVL6(dist=273221),ENPEP(dist=4319)//intergenic | 686 | chr4 | 111392992 | + | AF100309 | 1803 | - | 4q25 |
| 24N | 20C227789_R303_CapNGS | HBVBgp3_X | SYBU//intronic | 2212 | chr8 | 110648208 | + | AF100309 | 1828 | + | 8q23.2 |
| 24N | 20C227789_R303_CapNGS | HBVBgp3_X | TMEM131//intronic | 6210 | chr2 | 98489156 | - | AF100309 | 1809 | + | 2q11.2 |
| 24N | 20C227789_R303_CapNGS | HBVBgp1_P;HBVBgp4_C;HBVBgp3_X//upstream;downstream | TMEM131//intronic | 6229 | chr2 | 98489135 | + | AF100309 | 1876 | - | 2q11.2 |
| 24N | 20C227789_R303_CapNGS | HBVBgp3_X | LINC02267//ncRNA_intronic | 1237 | chr4 | 97283361 | + | AF100309 | 1826 | + | 4q22.3 |
| 24N | 20C227789_R303_CapNGS | HBVBgp3_X | MGAT4C(dist=60859),LOC105369879(dist=430880)//intergenic | 6302 | chr12 | 87293636 | + | AF100309 | 1827 | + | 12q21.32 |
| 24N | 20C227789_R303_CapNGS | HBVBgp2_S | GRM3//downstream | 633 | chr7 | 86494982 | + | AF100309 | 358 | - | 7q21.12 |
| 24N | 20C227789_R303_CapNGS | HBVBgp1_P;HBVBgp4_C;HBVBgp3_X//upstream;downstream | PCLO//intronic | 3018 | chr7 | 82698485 | + | AF100309 | 1841 | - | 7q21.11 |
| 24N | 20C227789_R303_CapNGS | HBVBgp3_X | PCLO//intronic | 3009 | chr7 | 82698448 | + | AF100309 | 1827 | - | 7q21.11 |
| 24N | 20C227789_R303_CapNGS | HBVBgp3_X | LINC02720(dist=658000),MIR4300HG(dist=459047)//intergenic | 15 | chr11 | 81131846 | - | AF100309 | 1566 | + | 11q14.1 |
| 24N | 20C227789_R303_CapNGS | HBVBgp3_X | LINC02720(dist=657996),MIR4300HG(dist=459051)//intergenic | 15 | chr11 | 81131842 | - | AF100309 | 1566 | + | 11q14.1 |
| 24N | 20C227789_R303_CapNGS | HBVBgp1_P;HBVBgp4_C;HBVBgp3_X//upstream;downstream | LOC102724084//ncRNA_intronic | 264 | chr16 | 80546994 | + | AF100309 | 1877 | - | 16q23.2 |
| 24N | 20C227789_R303_CapNGS | HBVBgp3_X;HBVBgp4_C;HBVBgp2_S//upstream;downstream | DPF3//intronic | 131 | chr14 | 73255370 | - | AF100309 | 1076 | + | 14q24.2 |
| 24N | 20C227789_R303_CapNGS | HBVBgp2_S | LINC00967(dist=26601),RRS1-AS1(dist=195667)//intergenic | 37 | chr8 | 67136155 | + | AF100309 | 404 | - | 8q13.1 |
| 24N | 20C227789_R303_CapNGS | HBVBgp4_C | EPHA5//intronic | 3164 | chr4 | 66487847 | - | AF100309 | 2238 | + | 4q13.1 |
| 24N | 20C227789_R303_CapNGS | HBVBgp4_C | EPHA5//intronic | 3179 | chr4 | 66487842 | + | AF100309 | 2238 | - | 4q13.1 |
| 24N | 20C227789_R303_CapNGS | HBVBgp3_X;HBVBgp4_C;HBVBgp2_S//upstream;downstream | EPHA5//intronic | 3183 | chr4 | 66487840 | + | AF100309 | 1202 | - | 4q13.1 |
| 24N | 20C227789_R303_CapNGS | HBVBgp2_S | EYS//intronic | 9079 | chr6 | 66370609 | + | AF100309 | 622 | + | 6q12 |
| 24N | 20C227789_R303_CapNGS | HBVBgp3_X | ADGRL3-AS1(dist=1573131),TECRL(dist=541404)//intergenic | 8971 | chr4 | 64600614 | + | AF100309 | 1803 | + | 4q13.1 |
| 24N | 20C227789_R303_CapNGS | HBVBgp4_C | ADGRL3-AS1(dist=1573129),TECRL(dist=541406)//intergenic | 8998 | chr4 | 64600612 | + | AF100309 | 2144 | + | 4q13.1 |
| 24N | 20C227789_R303_CapNGS | HBVBgp4_C | ADGRL3-AS1(dist=1573127),TECRL(dist=541408)//intergenic | 8948 | chr4 | 64600610 | + | AF100309 | 2144 | + | 4q13.1 |

| | | | | | | | | | | | |
|---|---|---|---|---|---|---|---|---|---|---|---|
| 24N | 20C227789_R303_CapNGS | HBVBgp4_C | LINC01135(dist=60422),LINC01358(dist=60342)//intergenic | 26 | chr1 | 59425806 | + | AF100309 | 2276 | + | 1p32.1 |
| 24N | 20C227789_R303_CapNGS | HBVBgp4_C | IRX6(dist=88838),MMP2-AS1(dist=7203)//intergenic | 4739 | chr16 | 55453506 | + | AF100309 | 2104 | - | 16q12.2 |
| 24N | 20C227789_R303_CapNGS | HBVBgp3_X | IRX6(dist=88818),MMP2-AS1(dist=7223)//intergenic | 4719 | chr16 | 55453486 | + | AF100309 | 1807 | - | 16q12.2 |
| 24N | 20C227789_R303_CapNGS | HBVBgp3_X;HBVBgp4_C;HBVBgp2_S//upstream;downstream | MIR4431(dist=800120),ASB3(dist=167244)//intergenic | 5 | chr2 | 53729873 | - | AF100309 | 1203 | + | 2p16.2 |
| 24N | 20C227789_R303_CapNGS | HBVBgp3_X | ONECUT1(dist=14468),LINC02490(dist=311561)//intergenic | 3365 | chr15 | 53097001 | + | AF100309 | 1771 | + | 15q21.3 |
| 24N | 20C227789_R303_CapNGS | HBVBgp4_C | NONE(dist=NONE),ASNSP1(dist=648560)//intergenic | 10 | chr8 | 46842277 | + | AF100309 | 2299 | + | 8q11.1 |
| 24N | 20C227789_R303_CapNGS | HBVBgp4_C | CNTNAP3P2(dist=147676),XLOC_007697(dist=109778)//interge | 6 | chr9 | 44071733 | + | AF100309 | 2048 | - | 9p11.2 |
| 24N | 20C227789_R303_CapNGS | HBVBgp3_X | C15orf54(dist=227423),THBS1(dist=98814)//intergenic | 4577 | chr15 | 39774466 | + | AF100309 | 1794 | + | 15q14 |
| 24N | 20C227789_R303_CapNGS | HBVBgp1_P;HBVBgp4_C;HBVBgp3_X//upstream;downstream | C15orf54(dist=227397),THBS1(dist=98840)//intergenic | 4456 | chr15 | 39774440 | + | AF100309 | 1885 | + | 15q14 |
| 24N | 20C227789_R303_CapNGS | HBVBgp2_S | ADAM18//intronic | 7512 | chr8 | 39559306 | + | AF100309 | 180 | + | 8p11.22 |
| 24N | 20C227789_R303_CapNGS | HBVBgp3_X | MICALL1//upstream | 97 | chr22 | 38302201 | + | AF100309 | 1804 | - | 22q13.1 |
| 24N | 20C227789_R303_CapNGS | HBVBgp3_X | TRIOBP//intronic | 569 | chr22 | 38109956 | + | AF100309 | 1824 | - | 22q13.1 |
| 24N | 20C227789_R303_CapNGS | HBVBgp4_C | UNC5D//intronic | 7039 | chr8 | 35264704 | + | AF100309 | 2395 | - | 8p12 |
| 24N | 20C227789_R303_CapNGS | HBVBgp3_X | UNC5D//intronic | 7001 | chr8 | 35264687 | + | AF100309 | 1793 | - | 8p12 |
| 24N | 20C227789_R303_CapNGS | HBVBgp3_X | DROSHA//intronic | 1467 | chr5 | 31403428 | + | AF100309 | 1827 | + | 5p13.3 |
| 24N | 20C227789_R303_CapNGS | HBVBgp3_X | PPP1CB(dist=5032),SPDYA(dist=2862)//intergenic | 1545 | chr2 | 29030838 | + | AF100309 | 1778 | + | 2p23.2 |
| 24N | 20C227789_R303_CapNGS | HBVBgp3_X | PPP1CB(dist=5003),SPDYA(dist=2891)//intergenic | 1549 | chr2 | 29030809 | + | AF100309 | 1818 | + | 2p23.2 |
| 24N | 20C227789_R303_CapNGS | HBVBgp3_X | SOX5//intronic | 1802 | chr12 | 23728447 | + | AF100309 | 1820 | - | 12p12.1 |
| 24N | 20C227789_R303_CapNGS | HBVBgp3_X | TMEM196(dist=71404),LOC101927668(dist=73960)//intergenic | 7711 | chr7 | 19884644 | + | AF100309 | 1736 | - | 7p21.1 |
| 24N | 20C227789_R303_CapNGS | HBVBgp4_C | LOC101929066(dist=40596),NAT1(dist=32220)//intergenic | 45 | chr8 | 17995756 | + | AF100309 | 2145 | - | 8p22 |
| 24N | 20C227789_R303_CapNGS | HBVBgp2_S | DMRT1//intronic | 8 | chr9 | 926894 | - | AF100309 | 194 | + | 9p24.3 |
| 25N | 20C227790_R303_CapNGS | HBVCgp3_X//upstream | PLA2G4A//intronic | 176 | chr1 | 186885758 | + | AY123041 | 418 | - | 1q31.1 |
| 25N | 20C227790_R303_CapNGS | HBVCgp4_PREC/C | FBXW7//intronic | 3047 | chr4 | 153378151 | + | AY123041 | 1723 | + | 4q31.3 |
| 25N | 20C227790_R303_CapNGS | HBVCgp4_PREC/C | FBXW7//intronic | 3037 | chr4 | 153378146 | + | AY123041 | 1723 | + | 4q31.3 |
| 25N | 20C227790_R303_CapNGS | HBVCgp3_X;HBVCgp4_PREC/C | CLMP(dist=141367),GRAMD1B(dist=21807)//intergenic | 2024 | chr11 | 123207323 | + | AY123041 | 1711 | - | 11q24.1 |
| 25N | 20C227790_R303_CapNGS | HBVCgp4_PREC/C | CLMP(dist=141279),GRAMD1B(dist=21895)//intergenic | 2018 | chr11 | 123207235 | + | AY123041 | 2209 | - | 11q24.1 |
| 25N | 20C227790_R303_CapNGS | HBVCgp4_PREC/C | MCM9//intronic | 1592 | chr6 | 119203273 | + | AY123041 | 2266 | - | 6q22.31 |
| 25N | 20C227790_R303_CapNGS | HBVCgp4_PREC/C | MCM9//intronic | 443 | chr6 | 119203263 | + | AY123041 | 2176 | + | 6q22.31 |
| 25N | 20C227790_R303_CapNGS | HBVCgp3_X | LINC02264//ncRNA_intronic | 1180 | chr4 | 118764891 | + | AY123041 | 1664 | + | 4q26 |
| 25N | 20C227790_R303_CapNGS | HBVCgp4_PREC/C | CSMD3(dist=1138378),TRPS1(dist=833178)//intergenic | 2486 | chr8 | 115587546 | + | AY123041 | 2276 | - | 8q23.3 |
| 25N | 20C227790_R303_CapNGS | HBVCgp3_X | POR//intronic | 60966 | chr7 | 75563203 | + | AY123041 | 1674 | + | 7q11.23 |
| 25N | 20C227790_R303_CapNGS | HBVCgp3_X;HBVCgp4_PREC/C | POR//intronic | 61275 | chr7 | 75563202 | + | AY123041 | 1692 | + | 7q11.23 |
| 25N | 20C227790_R303_CapNGS | HBVCgp3_X | POR//intronic | 61451 | chr7 | 75563200 | + | AY123041 | 1669 | + | 7q11.23 |
| 25N | 20C227790_R303_CapNGS | HBVCgp3_X//upstream | CENPC//intronic | 6 | chr4 | 68346639 | + | AY123041 | 445 | + | 4q13.2 |
| 25N | 20C227790_R303_CapNGS | HBVCgp3_X//upstream | NUSAP1//intronic | 42 | chr15 | 41646975 | + | AY123041 | 383 | + | 15q15.1 |
| 25N | 20C227790_R303_CapNGS | HBVCgp3_X | LINC00692(dist=389775),LRRC3B(dist=359336)//intergenic | 2094 | chr3 | 26304961 | + | AY123041 | 1668 | + | 3p24.2 |
| 25N | 20C227790_R303_CapNGS | HBVCgp3_X;HBVCgp4_PREC/C | ADGRL1(dist=27350),LINC01841(dist=71939)//intergenic | 18 | chr19 | 14344331 | + | AY123041 | 1695 | - | 19p13.12 |
| 25N | 20C227790_R303_CapNGS | HBVCgp4_PREC/C | ANOS1//intronic | 9 | chrX | 8622812 | + | AY123041 | 1811 | + | Xp22.31 |
| 26N | 20C227791_R303_CapNGS | HBVCgp3_X;HBVCgp4_PREC/C | FN1//intronic | 1469 | chr2 | 216283720 | + | AY123041 | 1697 | - | 2q35 |
| 26N | 20C227791_R303_CapNGS | HBVCgp3_X;HBVCgp4_PREC/C | FN1//intronic | 928 | chr2 | 216271736 | - | AY123041 | 1697 | + | 2q35 |
| 26N | 20C227791_R303_CapNGS | HBVCgp4_PREC/C | FN1//intronic | 73 | chr2 | 216251004 | + | AY123041 | 1929 | + | 2q35 |
| 26N | 20C227791_R303_CapNGS | HBVCgp4_PREC/C | FN1//intronic | 73 | chr2 | 216251001 | + | AY123041 | 1929 | + | 2q35 |
| 26N | 20C227791_R303_CapNGS | HBVCgp4_PREC/C | FN1//intronic | 688 | chr2 | 216245523 | - | AY123041 | 2101 | + | 2q35 |
| 26N | 20C227791_R303_CapNGS | HBVCgp4_PREC/C | LINC01934//ncRNA_intronic | 24 | chr2 | 182140518 | - | AY123041 | 1899 | + | 2q31.3 |
| 26N | 20C227791_R303_CapNGS | HBVCgp3_X;HBVCgp4_PREC/C | CACNB4//intronic | 1571 | chr2 | 152822443 | - | AY123041 | 1689 | + | 2q23.3 |
| 26N | 20C227791_R303_CapNGS | HBVCgp4_PREC/C | CXorf51B(dist=25912),MIR513C(dist=349048)//intergenic | 5 | chrX | 145922174 | + | AY123041 | 2093 | + | Xq27.3 |
| 26N | 20C227791_R303_CapNGS | HBVCgp3_X | CXorf51B(dist=25896),MIR513C(dist=349064)//intergenic | 36 | chrX | 145922158 | + | AY123041 | 1682 | - | Xq27.3 |
| 26N | 20C227791_R303_CapNGS | HBVCgp3_X;HBVCgp4_PREC/C//upstream | WNT2B//intronic | 20 | chr1 | 113061328 | + | AY123041 | 1049 | + | 1p13.2 |
| 26N | 20C227791_R303_CapNGS | HBVCgp4_PREC/C | LINC01492(dist=583135),LOC101928523(dist=91540)//intergeni | 3189 | chr9 | 106670450 | - | AY123041 | 2053 | + | 9q31.1 |
| 26N | 20C227791_R303_CapNGS | HBVCgp3_X;HBVCgp4_PREC/C | PCSK6//intronic | 927 | chr15 | 101950332 | + | AY123041 | 1691 | - | 15q26.3 |
| 26N | 20C227791_R303_CapNGS | HBVCgp3_X//upstream | SLC25A13//intronic | 14 | chr7 | 95888761 | + | AY123041 | 256 | - | 7q21.3 |
| 26N | 20C227791_R303_CapNGS | HBVCgp3_X;HBVCgp4_PREC/C | TSG1(dist=3001),MANEA-DT(dist=1518633)//intergenic | 266 | chr6 | 94489339 | + | AY123041 | 1695 | - | 6q16.1 |
| 26N | 20C227791_R303_CapNGS | HBVCgp4_PREC/C//downstream | ROBO1(dist=270652),LINC02050(dist=726248)//intergenic | 20 | chr3 | 80087800 | + | AY123041 | 2815 | + | 3p12.2 |
| 26N | 20C227791_R303_CapNGS | NONE(dist=NONE),HBVCgp3_X(dist=1079)//intergenic | ALB//exonic//ALB:NM_000477:exon13:c.1740 | 920 | chr4 | 74285311 | + | AY123041 | 169 | + | 4q13.3 |
| 26N | 20C227791_R303_CapNGS | HBVCgp3_X | RLIM(dist=62129),NEXMIF(dist=56103)//intergenic | 1122 | chrX | 73896588 | + | AY123041 | 1674 | - | Xq13.2 |
| 26N | 20C227791_R303_CapNGS | HBVCgp3_X//upstream | GRSF1(dist=4405),MOB1B(dist=58074)//intergenic | 173 | chr4 | 71710012 | + | AY123041 | 322 | + | 4q13.3 |
| 26N | 20C227791_R303_CapNGS | HBVCgp3_X//upstream | PPP6R3//intronic | 4603 | chr11 | 68278705 | + | AY123041 | 605 | - | 11q13.2 |
| 26N | 20C227791_R303_CapNGS | HBVCgp3_X;HBVCgp4_PREC/C | LINC01793(dist=740977),MIR4432HG(dist=338839)//intergenic | 8377 | chr2 | 60247512 | + | AY123041 | 1694 | - | 2p16.1 |
| 26N | 20C227791_R303_CapNGS | HBVCgp3_X//upstream | GLIS1//intronic | 688 | chr1 | 53991879 | + | AY123041 | 385 | + | 1p32.3 |
| 26N | 20C227791_R303_CapNGS | HBVCgp3_X//upstream | LINC02480//upstream | 4359 | chr4 | 52910487 | + | AY123041 | 422 | - | 4q12 |
| 26N | 20C227791_R303_CapNGS | HBVCgp4_PREC/C | SLC4A8//intronic | 9025 | chr12 | 51876787 | + | AY123041 | 1808 | - | 12q13.13 |

| | | | | | | | | | | | |
|---|---|---|---|---|---|---|---|---|---|---|---|
| 26N | 20C227791_R303_CapNGS | HBVCgp3_X//upstream | VDR(dist=17063),TMEM106C(dist=41453)//intergenic | 5 | chr12 | 48315877 | + | AY123041 | 572 | + | 12q13.11 |
| 26N | 20C227791_R303_CapNGS | HBVCgp4_PREC/C | TMEM63B//intronic | 1811 | chr6 | 44111900 | + | AY123041 | 2189 | - | 6p21.1 |
| 26N | 20C227791_R303_CapNGS | NONE(dist=NONE),HBVCgp3_X(dist=1208)//intergenic | LIPE-AS1//ncRNA_intronic | 12 | chr19 | 43020678 | - | AY123041 | 40 | + | 19q13.2 |
| 26N | 20C227791_R303_CapNGS | NONE(dist=NONE),HBVCgp3_X(dist=1208)//intergenic | LIPE-AS1//ncRNA_intronic | 8 | chr19 | 43020672 | + | AY123041 | 40 | - | 19q13.2 |
| 26N | 20C227791_R303_CapNGS | HBVCgp4_PREC/C | LINC01343(dist=207127),LINC01685(dist=53302)//intergenic | 13 | chr1 | 38887566 | + | AY123041 | 2173 | - | 1p34.3 |
| 26N | 20C227791_R303_CapNGS | HBVCgp4_PREC/C | LINC01343(dist=207126),LINC01685(dist=53303)//intergenic | 13 | chr1 | 38887565 | - | AY123041 | 2176 | + | 1p34.3 |
| 26N | 20C227791_R303_CapNGS | HBVCgp4_PREC/C | HEATR5A//intronic | 915 | chr14 | 31767002 | + | AY123041 | 2219 | + | 14q12 |
| 26N | 20C227791_R303_CapNGS | HBVCgp4_PREC/C | KLF13(dist=38542),OTUD7A(dist=1191)//intergenic | 50 | chr15 | 31766410 | - | AY123041 | 1837 | + | 15q13.3 |
| 26N | 20C227791_R303_CapNGS | NONE(dist=NONE),HBVCgp3_X(dist=1093)//intergenic | MIR548H4(dist=140151),STMN4(dist=46194)//intergenic | 969 | chr8 | 27046631 | - | AY123041 | 155 | + | 8p21.2 |
| 26N | 20C227791_R303_CapNGS | HBVCgp3_X;HBVCgp4_PREC/C | SKP1P2(dist=206231),LINC02378(dist=384964)//intergenic | 5718 | chr12 | 17349793 | + | AY123041 | 1694 | + | 12p12.3 |
| 26N | 20C227791_R303_CapNGS | HBVCgp3_X | SKP1P2(dist=206227),LINC02378(dist=384968)//intergenic | 5713 | chr12 | 17349789 | + | AY123041 | 1618 | + | 12p12.3 |
| 26N | 20C227791_R303_CapNGS | HBVCgp3_X | SKP1P2(dist=206219),LINC02378(dist=384976)//intergenic | 5696 | chr12 | 17349781 | + | AY123041 | 1618 | + | 12p12.3 |
| 26N | 20C227791_R303_CapNGS | HBVCgp3_X;HBVCgp4_PREC/C | NAA38//intronic | 105 | chr17 | 7786138 | + | AY123041 | 1697 | + | 17p13.1 |
| 26N | 20C227791_R303_CapNGS | HBVCgp4_PREC/C | PRMT8//intronic | 31 | chr12 | 3513872 | + | AY123041 | 2046 | + | 12p13.32 |
| 26N | 20C227791_R303_CapNGS | HBVCgp4_PREC/C | WASH8P(dist=4276),IQSEC3(dist=80394)//intergenic | 1356 | chr12 | 95539 | + | AY123041 | 1894 | + | 12p13.33 |
| 27N | 20C227792_R303_CapNGS | HBVCgp4_PREC/C | CPS1//intronic | 2181 | chr2 | 211433531 | + | AY123041 | 2210 | + | 2q34 |
| 27N | 20C227792_R303_CapNGS | HBVCgp3_X;HBVCgp4_PREC/C//upstream | TNR//intronic | 13 | chr1 | 175308480 | + | AY123041 | 706 | + | 1q25.1 |
| 27N | 20C227792_R303_CapNGS | HBVCgp3_X//upstream | SNX19(dist=284718),NTM(dist=169410)//intergenic | 9 | chr11 | 131071100 | - | AY123041 | 571 | + | 11q25 |
| 27N | 20C227792_R303_CapNGS | HBVCgp3_X//upstream | MIR3144(dist=293149),TBC1D32(dist=771088)//intergenic | 5 | chr6 | 120629552 | - | AY123041 | 643 | + | 6q22.31 |
| 27N | 20C227792_R303_CapNGS | NONE(dist=NONE),HBVCgp3_X(dist=1069)//intergenic | CCDC168//exonic//CCDC168:NM_001146197:exon4:c.7468 | 1760 | chr13 | 103395579 | + | AY123041 | 179 | + | 13q33.1 |
| 27N | 20C227792_R303_CapNGS | HBVCgp3_X | CCDC168//exonic//CCDC168:NM_001146197:exon4:c.7481 | 1755 | chr13 | 103395566 | + | AY123041 | 1524 | + | 13q33.1 |
| 27N | 20C227792_R303_CapNGS | HBVCgp3_X | MCHR2-AS1(dist=51932),SIM1(dist=260523)//intergenic | 20 | chr6 | 100576227 | + | AY123041 | 1581 | + | 6q16.2 |
| 27N | 20C227792_R303_CapNGS | HBVCgp3_X//upstream | SEC11A//intronic | 1254 | chr15 | 85239332 | + | AY123041 | 371 | - | 15q25.3 |
| 27N | 20C227792_R303_CapNGS | HBVCgp3_X;HBVCgp4_PREC/C | LOC101928978//ncRNA_intronic | 7 | chr4 | 84938098 | + | AY123041 | 1694 | + | 4q21.23 |
| 27N | 20C227792_R303_CapNGS | HBVCgp3_X | LRP1//intronic | 1226 | chr12 | 57551379 | + | AY123041 | 1518 | - | 12q13.3 |
| 27N | 20C227792_R303_CapNGS | HBVCgp2_PRES1/PRES2/S;HBVCgp3_X;HBVCgp4_PREC/C//upstream;downstre | LOC107986794(dist=1440317),POM121L12(dist=202056)//intergenic | 6 | chr7 | 52901270 | - | AY123041 | 2606 | + | 7p12.1 |
| 27N | 20C227792_R303_CapNGS | HBVCgp4_PREC/C | FBN1//intronic | 381 | chr15 | 48926345 | + | AY123041 | 1793 | + | 15q21.1 |
| 27N | 20C227792_R303_CapNGS | HBVCgp2_PRES1/PRES2/S;HBVCgp3_X;HBVCgp4_PREC/C//upstream;downstre | EIF4EBP1//intronic | 8 | chr8 | 37891222 | + | AY123041 | 2636 | - | 8p11.23 |
| 27N | 20C227792_R303_CapNGS | HBVCgp3_X//upstream | LRRFIP2(dist=43591),LOC152048(dist=21841)//intergenic | 11 | chr3 | 37261442 | - | AY123041 | 473 | + | 3p22.2 |
| 27N | 20C227792_R303_CapNGS | HBVCgp3_X;HBVCgp4_PREC/C//upstream | PHACTR4//intronic | 58 | chr1 | 28697179 | + | AY123041 | 1072 | - | 1p35.3 |
| 27N | 20C227792_R303_CapNGS | HBVCgp4_PREC/C//downstream | RERE//intronic | 8 | chr1 | 8540225 | - | AY123041 | 2998 | + | 1p36.23 |
| 27N | 20C227792_R303_CapNGS | HBVCgp4_PREC/C | AP2A2//intronic | 462 | chr11 | 980453 | - | AY123041 | 1765 | + | 11p15.5 |
| 18N | LD20C2277840102 | HBVCgp1_P;HBVCgp2_S | MIR4426//downstream | 205 | chr1 | 192685988 | + | X04615 | 821 | - | 1q31.2 |
| 18N | LD20C2277840102 | HBVCgp4_C;HBVCgp5_P | RFPL4B(dist=916693),LINC02518(dist=160553)//intergenic | 15466 | chr6 | 113589189 | + | X04615 | 2389 | - | 6q21 |
| 18N | LD20C2277840102 | HBVCgp4_C;HBVCgp5_P | RFPL4B(dist=916689),LINC02518(dist=160557)//intergenic | 15321 | chr6 | 113589185 | - | X04615 | 2389 | + | 6q21 |
| 18N | LD20C2277840102 | HBVCgp5_P | PPA2//intronic | 21 | chr4 | 106297142 | + | X04615 | 2871 | + | 4q24 |
| 18N | LD20C2277840102 | HBVCgp4_C | LINC01650(dist=5208),LINC01761(dist=117422)//intergenic | 199 | chr1 | 95822871 | + | X04615 | 2128 | + | 1p21.3 |
| 18N | LD20C2277840102 | HBVCgp4_C;HBVCgp5_P | ACAN//intronic | 5 | chr15 | 89348747 | + | X04615 | 2418 | + | 15q26.1 |
| 18N | LD20C2277840102 | HBVCgp5_P | SNHG5(dist=1071521),HTR1E(dist=187274)//intergenic | 33 | chr6 | 87459972 | - | X04615 | 2749 | + | 6q14.3 |
| 18N | LD20C2277840102 | HBVCgp1_P;HBVCgp2_S | NONE(dist=NONE),SLITRK1(dist=45551)//intergenic | 90 | chr13 | 84405789 | + | X04615 | 379 | + | 13q31.1 |
| 18N | LD20C2277840102 | HBVCgp5_P | IMPG1(dist=34954),LINC02540(dist=414817)//intergenic | 196 | chr6 | 76817349 | + | X04615 | 2765 | - | 6q14.1 |
| 18N | LD20C2277840102 | HBVCgp1_P;HBVCgp2_S | STK4//intronic | 10 | chr20 | 43661058 | + | X04615 | 282 | + | 20q13.12 |
| 18N | LD20C2277840102 | HBVCgp5_P | FOXK1//intronic | 1363 | chr7 | 4782203 | + | X04615 | 2964 | + | 7p22.1 |
| 18N | LD20C2277840102 | HBVCgp5_P | FOXK1//intronic | 1379 | chr7 | 4782198 | + | X04615 | 2964 | + | 7p22.1 |
| 18N | LD20C2277840102 | HBVCgp4_C | FOXK1//intronic | 2501 | chr7 | 4768220 | + | X04615 | 1993 | + | 7p22.1 |
| 18N | LD20C2277840102 | HBVCgp5_P | DLGAP2//intronic | 6 | chr8 | 1252095 | + | X04615 | 2957 | - | 8p23.3 |
| 21N | LD20C2277860101 | HBVBgp3_X | LINC01107(dist=167789),LOC100287387(dist=121258)//interge | 4887 | chr2 | 239631929 | - | AF100309 | 1822 | + | 2q37.3 |
| 21N | LD20C2277860101 | HBVBgp3_X | TRAF3IP1(dist=18236),ASB1(dist=7791)//intergenic | 83 | chr2 | 239327777 | - | AF100309 | 1815 | + | 2q37.3 |
| 21N | LD20C2277860101 | HBVBgp3_X | ECHS1//intronic | 45 | chr10 | 135186565 | - | AF100309 | 1778 | + | 10q26.3 |
| 21N | LD20C2277860101 | HBVBgp3_X | ECHS1//intronic | 45 | chr10 | 135186561 | - | AF100309 | 1778 | + | 10q26.3 |
| 21N | LD20C2277860101 | HBVBgp1_P;HBVBgp3_X;HBVBgp4_C//downstream | ZPLD1(dist=856381),MIR548AB(dist=187811)//intergenic | 2478 | chr3 | 103055066 | - | AF100309 | 2593 | + | 3q13.11 |
| 21N | LD20C2277860101 | HBVBgp4_C | CORO1B//upstream | 1792 | chr11 | 67212012 | + | AF100309 | 2069 | + | 11q13.2 |
| 21N | LD20C2277860101 | HBVBgp4_C | KCTD20//intronic | 26829 | chr6 | 36436632 | + | AF100309 | 2116 | - | 6p21.31 |
| 21N | LD20C2277860101 | HBVBgp3_X | KCTD20//intronic | 26838 | chr6 | 36436631 | - | AF100309 | 1643 | + | 6p21.31 |
| 21N | LD20C2277860101 | HBVBgp3_X;HBVBgp4_C;HBVBgp2_S//upstream;downstream | TMEM201(dist=30569),PIK3CD(dist=6285)//intergenic | 6 | chr1 | 9705504 | + | AF100309 | 1147 | + | 1p36.22 |
